# Expanding the Role of FurA as Essential Global Regulator in Cyanobacteria

**DOI:** 10.1371/journal.pone.0151384

**Published:** 2016-03-11

**Authors:** Andrés González, M. Teresa Bes, M. Luisa Peleato, María F. Fillat

**Affiliations:** Departamento de Bioquímica y Biología Molecular y Celular, Instituto de Biocomputación y Física de Sistemas Complejos (BIFI), Universidad de Zaragoza, Zaragoza, Spain; University of Freiburg, GERMANY

## Abstract

In the nitrogen-fixing heterocyst-forming cyanobacterium *Anabaena* sp. PCC 7120, the ferric uptake regulator FurA plays a global regulatory role. Failures to eliminate wild-type copies of *furA* gene from the polyploid genome suggest essential functions. In the present study, we developed a selectively regulated *furA* expression system by the replacement of *furA* promoter in the *Anabaena* sp. chromosomes with the Co^2+^/Zn^2+^ inducible *coaT* promoter from *Synechocystis* sp. PCC 6803. By removing Co^2+^ and Zn^2+^ from the medium and shutting off *furA* expression, we showed that FurA was absolutely required for cyanobacterial growth. RNA-seq based comparative transcriptome analyses of the *furA*-turning off strain and its parental wild-type in conjunction with subsequent electrophoretic mobility shift assays and semi-quantitative RT-PCR were carried out in order to identify direct transcriptional targets and unravel new biological roles of FurA. The results of such approaches led us to identify 15 novel direct iron-dependent transcriptional targets belonging to different functional categories including detoxification and defences against oxidative stress, phycobilisome degradation, chlorophyll catabolism and programmed cell death, light sensing and response, heterocyst differentiation, exopolysaccharide biosynthesis, among others. Our analyses evidence novel interactions in the complex regulatory network orchestrated by FurA in cyanobacteria.

## Introduction

With only few exceptions, iron is absolutely essential for life of all forms since this metal participates as protein cofactor in major biological processes, such as photosynthesis, respiration, nitrogen fixation, and nucleic acids biosynthesis. Aerobic organisms must face a survival paradox: iron is required for growth, but on the other hand, it is also potentially toxic due to its ability to catalyze the formation of reactive oxygen species (ROS) by Fenton reactions [1]. Furthermore, iron became scarce and growth-limiting in most ecological niches, since the predominant form of this micronutrient in nature is extremely insoluble at neutral pH. To cope with this limitation, bacteria have evolved strategies to efficiently scavenge iron from the environment and thereby ensure its physiological demands, but at the same time they have developed molecular mechanisms to tightly regulate iron homeostasis in order to maintain its intracellular concentration within non-toxic levels [2].

In most bacterial species, the concerted expression of genes involved in iron scavenge, uptake, storage, and metabolism is transcriptionally regulated by the ferric uptake regulator (Fur) protein in response to iron availability [2, 3]. Fur is a homodimeric metalloprotein that complexes with ferrous iron under high intracellular Fe^2+^ concentrations and typically acts as a transcriptional repressor by binding to *cis*-acting regulatory elements known as Fur boxes, which are located in the promoters of target genes [4]. When intracellular iron is depleted, the Fur-Fe^2+^ complexes dissociate from the Fur boxes, allowing transcription of target genes. In less frequent cases, Fur has been reported to act positively rather than negatively on the expression of certain genes by the occurrence of different mechanisms of regulation [5–8]. To date, apo-regulation of Fur has been observed only in epsilon-proteobacteria like *Helicobacter pylori* [9] and *Campylobacter jejuni* [10].

Cyanobacteria constitute an abundant, widespread and diverse group of photoautotrophic microorganisms which possesses the unique ability among prokaryotes to perform oxygenic photosynthesis. In addition, many species of this group are able to fix atmospheric dinitrogen when combined nitrogen sources are shortly supplied, thereby contributing significantly to the Earth’s nitrogen cycle [11]. Since iron is an essential redox component for both, the photosynthetic electron transfer chain and the nitrogenase enzymatic complex, the requirements of iron in cyanobacteria far exceeds those of heterotrophic, non-diazotrophic bacteria [12, 13]. On the other hand, in addition to ROS produced by the respiratory machinery, cyanobacteria are overexposed to those ROS unavoidably generated during the photosynthetic electron flow [14]. Therefore, the challenge of balancing iron homeostasis and oxidative stress become particularly acute in these organisms.

In the filamentous, nitrogen-fixing, heterocyst forming cyanobacterium *Anabaena* sp. PCC 7120, iron homeostasis is orchestrated by the ferric uptake regulator FurA, which modulates the expression of siderophore biosynthesis machineries, TonB-dependent siderophore outer membrane transporters, siderophore periplasmic binding proteins, ABC inner membrane permeases, ferritin Dps family proteins, as well as several enzymes involved in the tetrapyrrole biosynthesis pathway [15–17]. FurA is a constitutive protein whose expression increases under iron limitation [18] and oxidative stress conditions [19]. This metalloregulator associates with DNA in an iron dependent manner, at a 19- to 23-bp consensus binding site with the sequence AAATAAATTCTCAATAAAT [20], which shares 58% homology to the canonical *Escherichia coli* Fur box consensus sequence [21]. Previous analyses have shown that FurA is a global transcriptional regulator, modulating not only the iron assimilation genes, but also a plethora of genes and operons involved in a variety of physiological processes including photosynthesis, respiration, oxidative stress defences, heterocyst differentiation, light-dependent signal transduction mechanisms, among others [17, 20, 22–24]. As other Fur orthologues, FurA displays a dual role as transcriptional modulator, acting both as repressor and as activator of gene expression [15, 20].

Although comparative global analyses of transcriptomes and proteomes for *fur* deletion mutants have been traditionally used to characterize Fur regulons [25–28], attempts to inactivate *furA* from the polyploid genome of *Anabaena* sp. have resulted in only a partial segregation of the mutated chromosomes, suggesting a putative essential role for this protein under standard culture conditions [29]. Similar results had been previously obtained with Fur from *Synechococcus elongatus* [30], which share 75% identity with FurA. Hence, most *in vivo* analyses of FurA regulatory roles have been carried out on *furA*-overexpressing backgrounds [15, 17, 20, 22, 24, 31]. To conclusively establish the essentiality of *furA* and go in-depth on the knowledge of its function, we developed a selectively regulating *furA* expression system by the replacement of *furA* promoter in the *Anabaena* sp. chromosomes with the Co^2+^ and Zn^2+^ inducible *coaT* promoter [32]. This approach allowed us to grow the cells with Co^2+^/Zn^2+^ so that *furA* was expressed and subsequently to remove Co^2+^/Zn^2+^ from the medium to shut off *furA* expression, mimicking the *furA* null phenotype. We showed that FurA was absolutely required for cyanobacterial growth. Comparative transcriptome analysis of the *furA*-turning off phenotype led us to identify 15 novel FurA direct targets involved in different physiological processes.

## Results

### FurA is an essential, well-conserved protein among cyanobacteria

**Fig 1:**
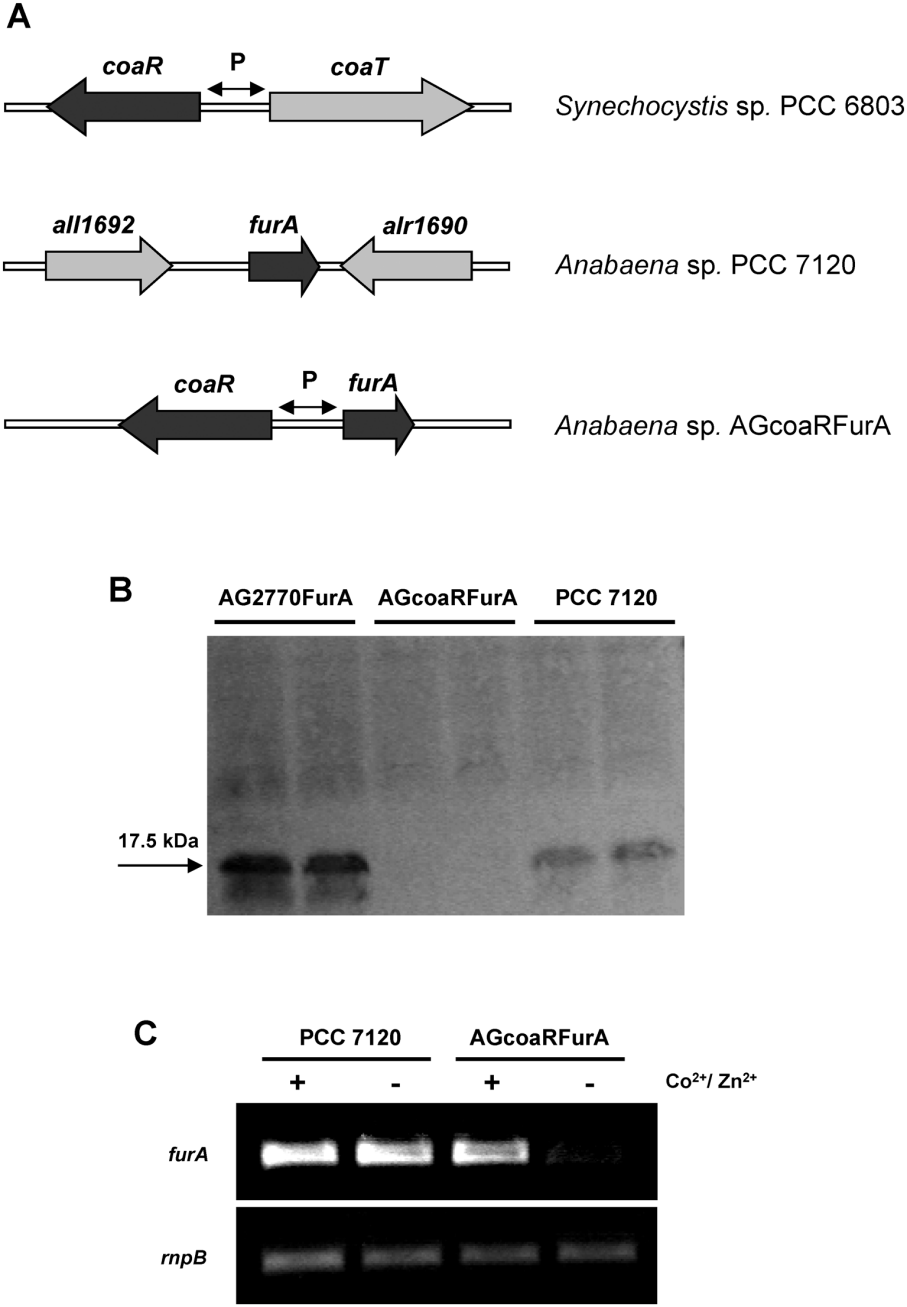
Construction of the *coaR*-P_*coaT*_::*furA* fusion strain AGcoaRFurA. (A) The normal promoter region of the *furA* gene in the *Anabaena* sp. PCC 7120 chromosome was replaced with the divergent cobalt/zinc-inducible *coaT* promoter (P_*coaT*_) and the coding region of the transcriptional activator CoaR from *Synechocystis* sp. PCC 6803. (B) Levels of FurA protein in wild-type *Anabaena* sp. strain PCC 7120, the *furA*-overexpressing strain AG2770FurA and the *coaR*-P_*coaT*_::*furA* fusion strain AGcoaRFurA, revealed by Western blotting. Total cell extracts from filaments grown in Co^2+^/Zn^2+^ deprived medium (BG-11_-Co/Zn_) were separated in duplicate by SDS-PAGE, electrotransferred, and challenged with anti-FurA antiserum. Molecular weight is indicated. (C) *furA* expression in mid-log phase cultures of wild-type and AGcoaRFurA strains growing with and without Co^2+^/Zn^2+^, according to sqRT-PCR. Expression of the housekeeping gene *rnpB* is also shown.

The native promoter region of the *furA* gene was replaced with the cobalt/zinc-inducible *coaT* promoter (P_*coaT*_) to allow regulation of *furA* by controlling the level of Co^2+^ and Zn^2+^ in the medium. The divergently transcribed *coaT* and *coaR* genes (Fig 1A) mediate inducible resistance to Co^2+^ in *Synechocystis* sp. PCC 6803 [32]. In the *coa* operon, *coaT* encodes a Co^2+^ efflux P-type ATPase similarly induced by both Co^2+^ and Zn^2+^, while *coaR* encodes a transcriptional activator of *coaT* whose carboxyl-terminal Cys-His-Cys motif is required for cobalt sensing. Previous bioluminescence assays have shown that gene expression under the control of *coaR*-P_*coaT*_ responded to Co^2+^ and Zn^2+^ with a detection range of 0.3–6 μM and 1–3 μM, respectively. In mixed samples of Co^2+^ and Zn^2+^, the bioluminescence responded in an additive manner [32]. In the strain *Anabaena* sp. AGcoaRFurA, *coaR*-P_*coaT*_ and the coding region of *furA* were fused at their respective ATG translational start sites and were located immediately downstream of the predicted transcriptional terminator of *all1692*, the gene upstream of *furA* on the chromosome (Fig 1A). The region between *furA* and alr1690 remained the same as in wild-type *Anabaena* sp. PCC 7120.

To test the *coaR*-P_*coaT*_::*furA* fusion strain, we grew it in standard BG-11 medium to mid-log phase (3–5 μg Chl/ml), the filaments were subsequently washed with BG-11 medium without Co^2+^/Zn^2+^ (BG-11_-Co/Zn_), and re-suspended in the same Co^2+^/Zn^2+^-free medium with further incubation for 48 h. Under those conditions, the *furA* expression in the AGcoaRFurA strain was completely suppressed according to western blotting (Fig 1B). Expression of *furA* by the strain AGcoaRFurA when grown in the standard BG-11 medium, which contains 0.4 μM of cobalt and 0.8 μM of zinc [33], was quite similar to the wild-type *Anabaena* sp. PCC 7120 (Fig 1C).

**Fig 2:**
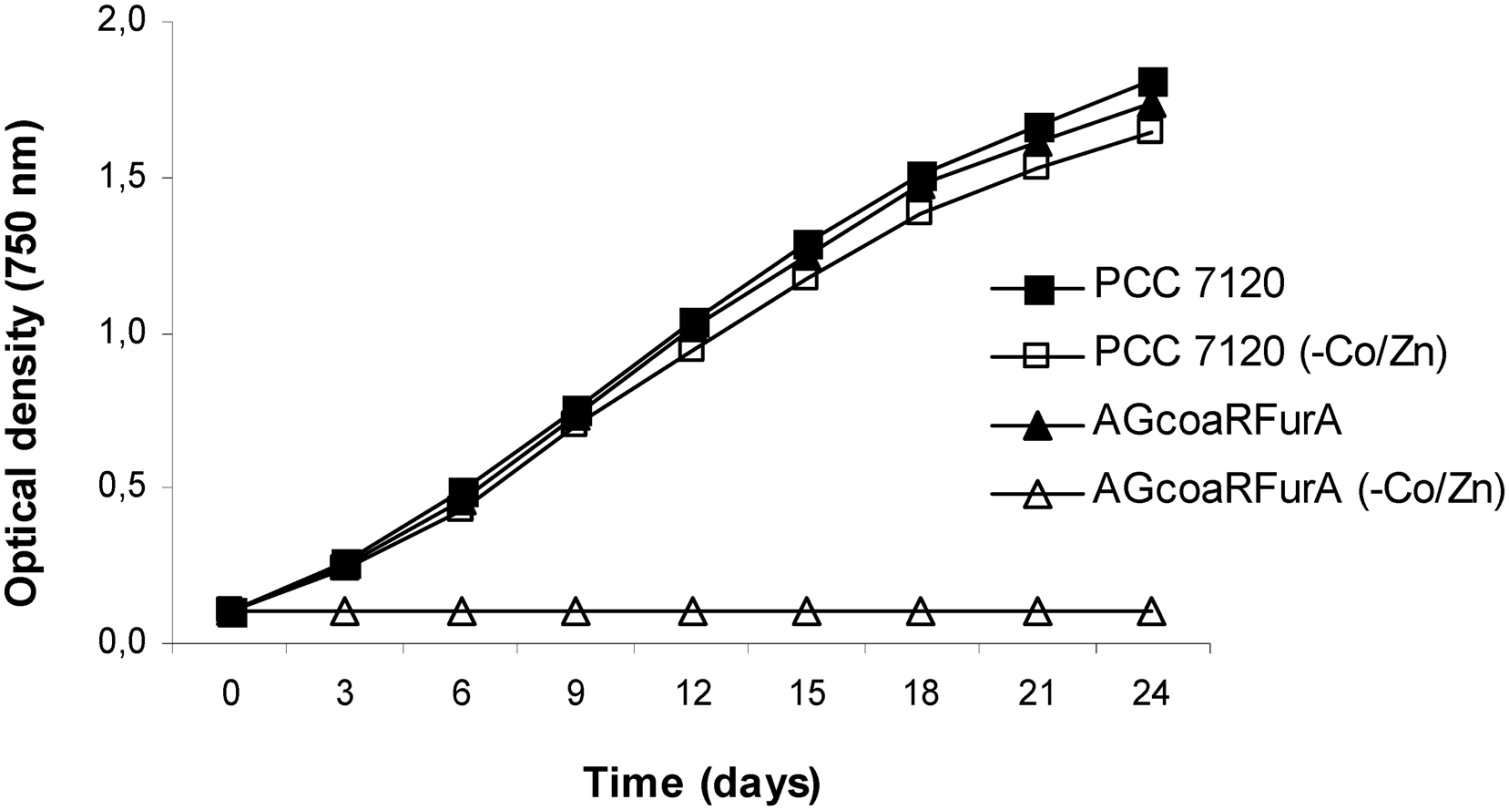
Expression of FurA results essential to the growth of *Anabaena* sp. under standard culture conditions. Growth curves of the *coaR*-P_*coaT*_::*furA* fusion strain *Anabaena* sp. AGcoaRFurA and the wild-type strain *Anabaena* sp. PCC 7120 in the presence or absence of Co^2+^/Zn^2+^.

To evaluate the essentiality of FurA for the *Anabaena* sp. physiology, the strains wild-type *Anabaena* sp. PCC 7120 and *Anabaena* sp. AGcoaRFurA were photoautotrophically grown in BG-11 medium solidified with 1% Noble agar (Difco). Cultures were incubated under continuous white light illumination (20 μE/m^2^s) and controlled temperature of 30°C. Under these conditions, both strains grew abundantly, forming a confluent grow on the agar in 10–14 days (Figures A1 and A3 in S1 File). Both strains were subsequently subcultured in solidified BG-11 medium without Co^2+^ and Zn^2+^. As shown in Figures A2 and A4 in S1 File, elimination of both metals to the medium impaired growth of the *coaR*-P_*coaT*_::*furA* fusion strain, suggesting that normal expression of FurA results essential for the growth of *Anabaena* sp. under standard culture conditions. Similar results were observed in liquid BG-11 medium (Fig 2). Since *furA* appears to have orthologs sharing ≥ 80% protein sequence identity in more than 10 cyanobacterial families and 25 genera (Table A in S1 File), this metalloregulator constitutes an essential well-conserved protein in cyanobacteria.

### Turning off *furA* induces substantial changes in the *Anabaena* sp. transcriptome

To examine the global impact of FurA in the *Anabaena* sp. physiology, comparative transcriptomic analysis was performed for the AGcoaRFurA and wild-type PCC 7120 strains in Co^2+^/Zn^2+^ restricted cultures. In total, 2089 genes (approximately 33% of the PCC 7120 genome) exhibited differential expression after turning off *furA* (at least 2-fold difference). The *coaR*-P_*coaT*_::*furA* fusion strain showed a 4.4-fold decrease in expression of *furA* comparing with wild-type strain under the same culture conditions. Depletion of FurA levels resulted in the increased expression of 494 genes and the decreased expression of 1595 genes (Table B in S1 File). RPKM values and fold change for other ORF of the chromosome and six natural plasmids of *Anabaena* sp. are presented in an additional file (Table C in S1 File).

**Fig 3:**
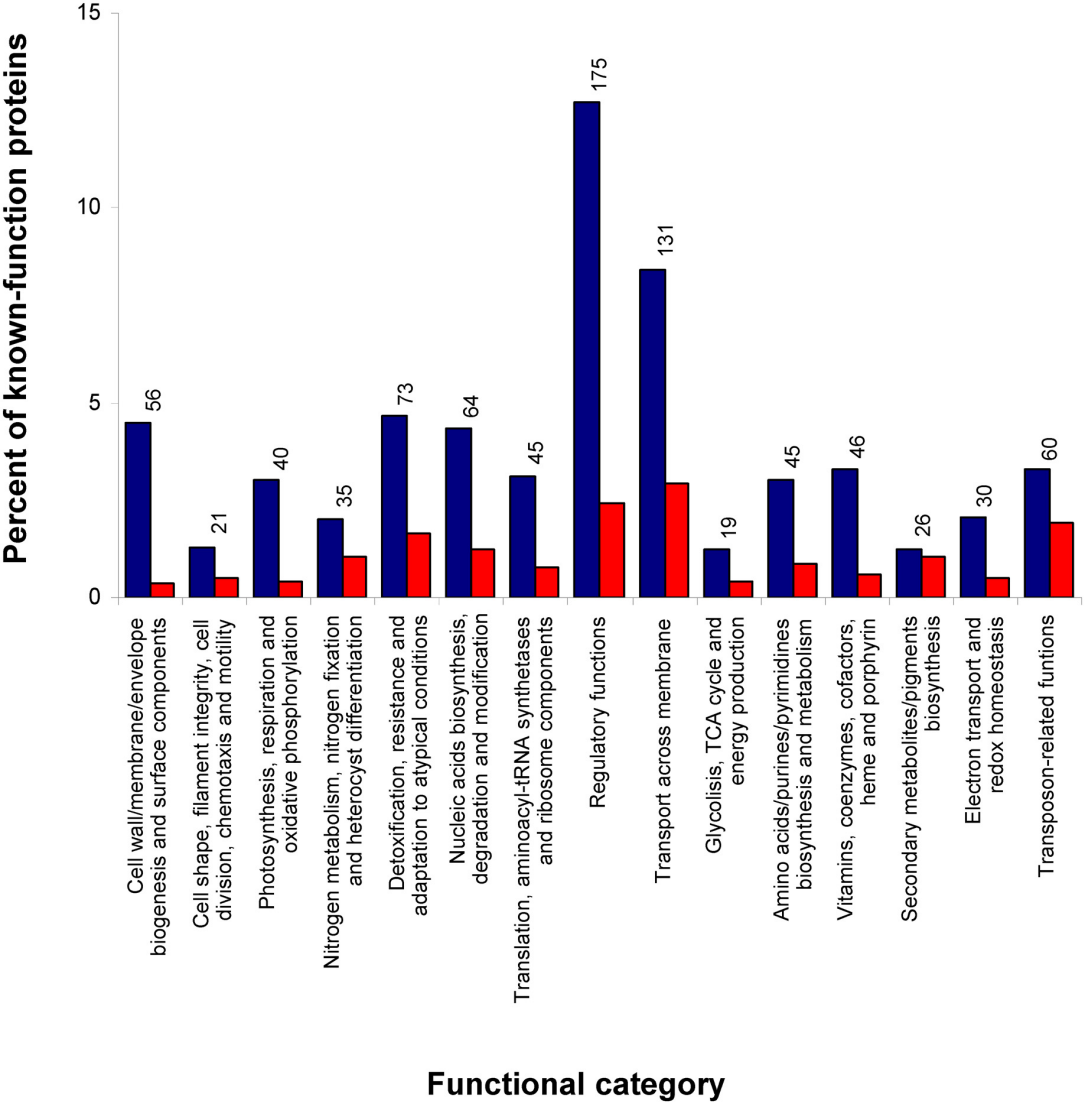
Distribution of known-function genes which exhibited differential expression after turning off *furA* in the *coaR*-P_*coaT*_::*furA* fusion strain AGcoaRFurA. Blue columns represent the percent of genes down-regulated of the total (n = 1155) genes with defined function, distributed according to their functional category. Red columns represent the percent of genes up-regulated. The numbers over the columns indicate the total amount of genes by functional category showing differential expression.

Among the 1155 genes with defined functions, two functional categories were highly enriched with differentially expressed products: (1) genes involved in regulatory functions, and (2) genes related to transport across membrane (Fig 3). Beyond those well-recognized FurA targets belonging to iron homeostasis, several genes involved in less studied Fur coordinated processes such as photosynthesis, detoxification, light sensing and response, exopolysaccharide biosynthesis, chlorophyll catabolism or transposon related functions appeared differentially expressed after depletion of FurA levels.

### FurA controls the expression of novel direct targets

To determine whether the expression of genes identified by RNA-seq was directly regulated by FurA, we investigate the ability of recombinant FurA to bind *in vitro* the promoter regions of selected 30 up-regulated (Table 1) and 30 down-regulated genes (Table 2) in the *coaR*-P_*coaT*_::*furA* fusion strain, belonging to more than 20 different functional categories. Since the analysis of those well-recognized FurA putative targets involved in iron homeostasis has been extensively studied in previous works [15, 17, 20], we made special emphasis in the identification of putative novel targets involved in distinctive processes of cyanobacteria as microbial group, such as oxygenic photosynthesis, biosynthesis and catabolism of chlorophyll as well as mechanisms of light sensing and response. We also investigate the implication of FurA in less studied cyanobacterial processes such as exopolysaccharide biosynthesis, transposon related function, biosynthesis of the Fe-S cluster, among others. Electrophoretic mobility shift assays (EMSA) were carried out using 300- to 400-bp-DNA fragments located immediately upstream of the translational start point, corresponding to the promoter regions of each selected gene. In the case of large promoters, the entire promoter region was analyzed as several fragments of 300- to 400-bp in independent experiments. To confirm the specificity of bindings, all assays included the promoter region of the *nifJ* gene as non-specific competitor DNA [23]. The impact of metal co-repressor and reducing conditions on the *in vitro* affinity of recombinant FurA to its putative targets was evaluated in all assays. The specific binding of FurA to the promoter region of its own gene was used as positive control [34], while promoters of *Anabaena* sp. superoxide dismutases genes *sodA* and *sodB* were included as negative controls [17].

**Table 1:** Subset of selected genes showing a ≥2-fold change increase of expression in the *furA*-turning off strain AGcoaRFurA.

ORF^a^	Symbol^b^	Protein description^b^	Functional category	Fold change	EMSA^a^
*all1847*		CheB methylesterase	chemotaxis, cell motility	6.31	-
***all7348***		lethal leaf-spot 1 homolog	chlorophyll catabolism, developmentally programmed death	4.73	**+**
*alr5007*		similar to cell death suppressor protein, Lls1	chlorophyll catabolism, developmentally programmed death	3.15	-
*all2097*		cell death suppressor protein	chlorophyll catabolism, developmentally programmed death	3.15	-
*alr3195*		glutathione S-transferase	detoxification, adaptations and atypical conditions	6.31	-
***alr0799***		glutaredoxin-related protein	detoxification, adaptations and atypical conditions	3.94	**+**
*alr7354*		glutathione S-transferase	detoxification, adaptations and atypical conditions	2.60	-
*all0177*	*flv1b*	heterocyst-specific flavodiiron protein, Flv1	detoxification, adaptations and atypical conditions	2.37	-
***asl4146***	***srxA***	sulfiredoxin	detoxification, adaptations and atypical conditions	4.18	**+**
***all5185***		mercuric reductase	detoxification, adaptations and atypical conditions	3.35	**+**
***alr3090***	***katB***	Mn-catalase	detoxification, adaptations and atypical conditions	2.37	**+**
*alr3100*		holliday junction resolvase, YqgF	DNA replication, recombination and repair	4.73	-
***all3135***	***exoV***	succinoglycan biosynthesis ketolase, ExoV	exopolysaccharide biosynthesis	6.31	**+**
*alr3513*		Fe-S metabolism associated, SufE	Fe-S cluster biosynthesis	7.88	-
*all2566*	*gap1*	glyceraldehyde-3-phosphate dehydrogenase	glycolisis	4.73	-
***alr3356***		similar to phytochrome	light sensing and response	3.94	**+**
*alr0034*	*menC*	O-succinylbenzoic acid synthase, MenC	menaquinone biosynthesis	6.31	-
*asr0941*	*psbX*	photosystem II protein, PsbX	photosynthesis	4.60	-
***asr4517***	***nblA***	phycobilisome degradation protein, NblA	phycobilisome and phycobiliproteins	2.37	**+**
*alr3707*	*pcyA*	phycocyanobilin:ferredoxin oxidoreductase	phytochromobilin biosynthesis	2.01	-
*alr2945*		probable orotate phosphoribosyltransferase	pyrimidine biosynthesis	3.68	-
*all1681*		aspartate carbamoyltransferase	pyrimidine biosynthesis	6.31	-
***alr1010***	***ccbP***	calcium-binding protein, CcbP	regulatory functions	2.37	**+**
*all1649*		similar to polyketide synthase	siderophore/cyanotoxin biosynthesis	9.46	-
*all1647*		peptide synthetase	siderophore/cyanotoxin biosynthesis	3.94	-
***alr2679***		polyketide synthase	siderophore/cyanotoxin biosynthesis	3.15	**+**
***alr2680***		polyketide synthase	siderophore/cyanotoxin biosynthesis	2.15	**+**
*all4026*	*iacT*	TonB-dependent receptor	transport across membrane	3.15	-
***asr7385***		transposase	transposon related function	3.17	**+**
***alr7386***		transposase	transposon related function	3.15	**+**

^***a***^ FurA direct target genes according to EMSA results are indicated in bold letters.

^***b***^ Gene symbol and protein description according to the cyanobacteria genome database CyanoBase (http://genome.microbedb.jp/cyanobase).

**Table 2:** Subset of selected genes showing a ≥2-fold change decrease of expression in the *furA*-turning off strain AGcoaRFurA.

ORF^a^	Symbol^b^	Protein description^b^	Functional category	Fold change	EMSA^a^
***alr2495***	***sufS***	cysteine desulphurase, SufS	biosyntesis of Fe-S cluster	-2.78	**+**
*alr1358*		Mg-protoporphyrin IX monomethyl ester cyclase	chlorophyll biosynthesis	-2.22	-
*alr1105*		arsenate reductase	detoxification, adaptations and atypical conditions	-7.08	-
*all3033*		arsenical pump membrane protein	detoxification, adaptations and atypical conditions	-2.38	-
*asr1102*		arsenical-resistance protein ACR3, efflux transporter	detoxification, adaptations and atypical conditions	-2.35	-
*all1291*	*cynS*	cyanate lyase, CynS	detoxification, adaptations and atypical conditions	-5.56	-
*all4148*	*petF*	ferredoxin I	electron transfer agents in biological redox reactions	-2.87	-
*asl0884*		probable ferredoxin [2Fe-2S]	electron transfer agents in biological redox reactions	-5.90	-
*asl2914*		similar to ferredoxin	electron transfer agents in biological redox reactions	-6.23	-
*all3735*		fructose-bisphosphate aldolase class I	glycolisis, gluconeogenesis and the Calvin cycle	-4.15	-
*alr2392*	*fraC*	filament integrity proteína, FraC	integrity of cell junctions	-2.75	-
*asr3846*	*psbF*	cytochrome b559 beta subunit	photosynthesis	-2.54	-
*alr3422*	*petD*	cytochrome b6/f-complex, apocytochrome subunit 4, PetD	photosynthesis	-4.60	-
*all1512*	*petC*	cytochrome b6/f-complex, iron-sulfur proteína, PetC	photosynthesis	-3.17	-
*all1365*	*cytM*	cytochrome, CytM	photosynthesis	-4.67	-
*asr1283*	*psaX*	photosystem I 4.8K protein, PsaX	photosynthesis	-2.54	-
*asr3848*	*psbJ*	photosystem II protein, PsbJ	photosynthesis	-2.85	-
*asl0846*	*psbH*	photosystem II protein, PsbH	photosynthesis	-2.83	-
*asl0885*	*psbK*	photosystem II protein, PsbK	photosynthesis	-3.07	-
*asr0847*	*psbN*	photosystem II protein, PsbN	photosynthesis	-5.31	-
*all0801*	*psbW*	photosystem II protein, PsbW	photosynthesis	-7.30	-
*all0450*	*apcA*	allophycocyanin alpha subunit, ApcA	phycobilisomes and phycobiliproteins	-5.08	-
*all3653*	*apcD*	allophycocyanin B alpha chain, ApcD	phycobilisomes and phycobiliproteins	-2.22	-
*asr8504*	*nblA*	phycobilisome degradation protein, NblA plasmidic	phycobilisomes and phycobiliproteins	-2.54	-
*alr3814*	*nblB*	phycocyanin alpha phycocyanobilin lyase, NblB	phycobilisomes and phycobiliproteins	-3.49	-
*all3549*		similar to phycoerythrobilin lyase subunit (cpeF)	phycobilisomes and phycobiliproteins	-2.19	-
*alr3147*		phosphoenolpyruvate synthase	pyruvate metabolism and carboxylate cycle	-6.98	-
*alr3397*		phosphoenolpyruvate synthase	pyruvate metabolism and carboxylate cycle	-5.00	-
*alr4908*	*lexA*	SOS function regulatory protein, LexA	regulatory functions	-2.54	-
***alr4686***		cytochrome P450, germacrene A hydroxylase	sesquiterpene biosynthesis	-4.87	**+**

^***a***^ FurA direct target genes according to EMSA results are indicated in bold letters.

^***b***^ Gene symbol and protein description according to the cyanobacteria genome database CyanoBase (http://genome.microbedb.jp/cyanobase).

**Fig 4:**
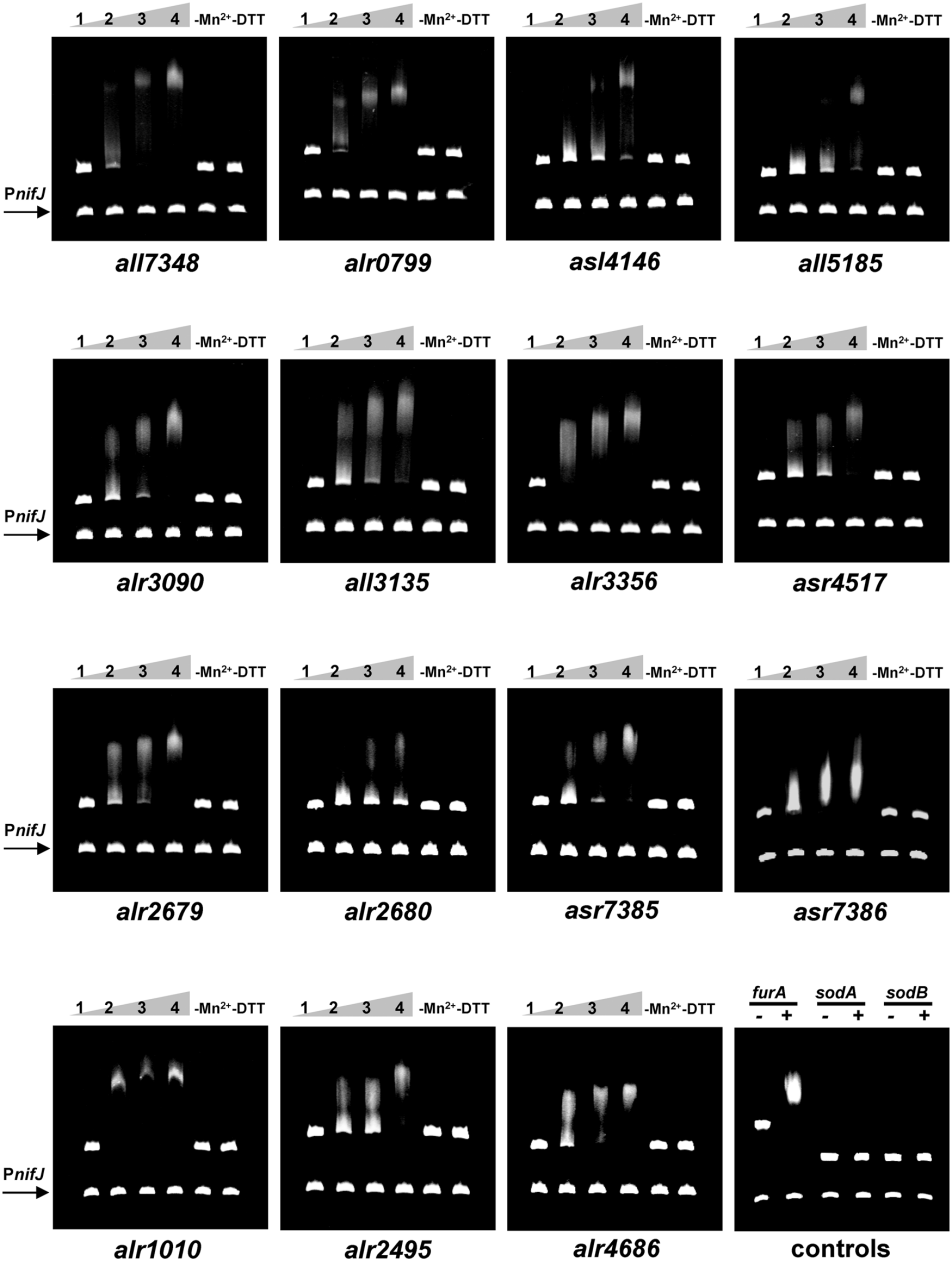
Electrophoretic mobility shift assays showing the ability of FurA to bind *in vitro* the promoter regions of novel direct target genes. DNA fragments free (1) or mixed with recombinant FurA protein at concentration of 300 nM (2), 500 nM (3) and 700 nM (4) in the presence of Mn^2+^ and DTT were separated on a 4% PAGE. The impact of the metal co-regulator (removing Mn^2+^/adding EDTA) and reducing conditions (removing DTT) on the *in vitro* affinity of FurA (700 nM) to each target are also showed. The promoter region of *nifJ* gene was used as non-specific competitor DNA in all assays. Binding of FurA (700 nM) to its own promoter was included as positive controls, while promoter regions of superoxide dismutases genes *sodA* and *sodB* were used as negative controls.

As shown in Fig 4, the EMSA experiments demonstrated that FurA specifically bound *in vitro* to the promoter regions of at least 15 of the selected 60 differently expressed genes. All the tested DNA fragments were shifted in the presence of up to 700 nM FurA in a dose-dependent manner, whereas the same concentrations of the regulator were unable to shift either the non-specific competitor or both negative controls. As occurs with all so far described FurA targets, the *in vitro* specific binding of the regulator to the operator regions of these novel target genes was strongly dependent on the presence of divalent metal ions and reducing conditions.

Among the novel direct FurA targets, we found genes related to detoxification and protection against oxidative stress like the sulfiredoxin SrxA (Asl4146) and the Mn-catalase Alr3090, genes involved in siderophore/cyanotoxin biosynthesis like the polyketide synthases Alr2679 and Alr2680, and genes involved in transposon-related functions such as transposasas Asr7385 and Alr7386. Interestingly, FurA specifically bound to the promoter regions of the calcium-binding protein CcbP (Alr1010), the phycobilisome degradation protein NblA (Asr4517), and the cysteine desulphurase SufS (Alr2495). Thirteen of the 15 novel FurA direct targets corresponding to genes up-regulated in the *coaR*-P_*coaT*_::*furA* fusion strain (Table 1).

### FurA might function not only as a repressor, but also as an activator of gene expression

**Fig 5:**
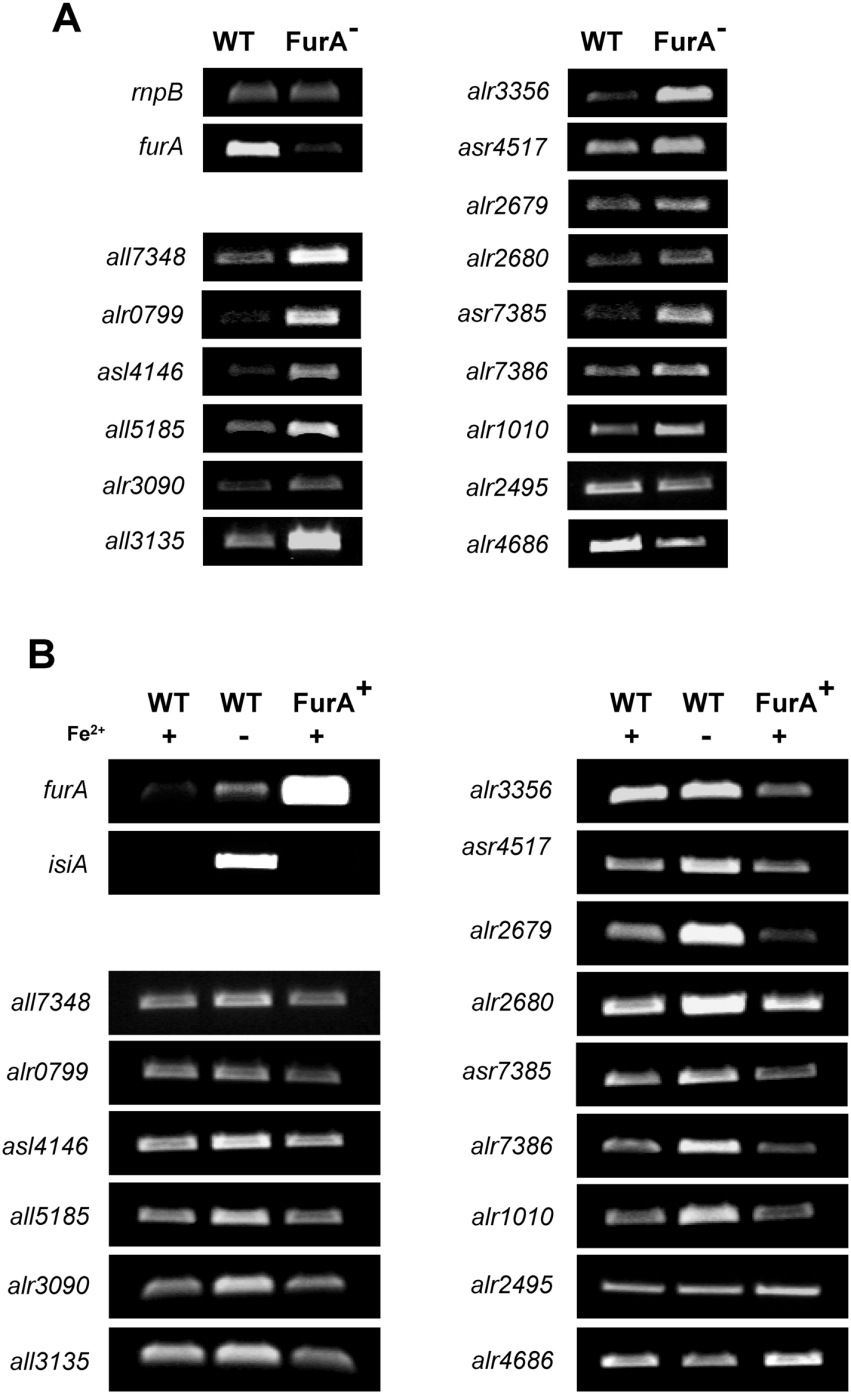
Semi-quantitative RT-PCR analyses showing the impact of FurA depletion, FurA overexpression and iron deprivation on the transcriptional pattern of novel FurA targets. (A) Total RNA from the wild-type strain PCC 7120 (WT) and the *coaR*-P_*coaT*_::*furA* fusion strain AGcoaRFurA (FurA^-^) were isolated from cells grown in Co^2+^/Zn^2+^ deprived medium (BG-11_-Co/Zn_). (B) Total RNA from the wild-type strain PCC 7120 (WT) and the *furA* overexpressing strain AG2770FurA (FurA^+^) were isolated from cells grown in standard BG-11 medium (+Fe^2+^) or iron deprived medium BG-11_-Fe_ (-Fe^2+^). Housekeeping gene *rnpB* was used as control. Determinations for each gene were performed in the early exponential phase of PCR. Expression analyses of genes *furA* and *isiA* were included as controls of experimental conditions. All determinations were performed three times with independent biological samples, and the relevant portion of a representative gel is shown for each gene.

The RNA-seq data corresponding to the 15 novel FurA direct targets were validated in a second experiment by semi-quantitative reverse transcription-PCR (sqRT-PCR). In order to obtain accurate determinations, each measure was performed at the early exponential phase of PCR. The housekeeping gene *rnpB* was included in all RT-PCR analyses to ensure that equivalent amounts of total RNA were being used in all reactions. As shown in Fig 5A and Figure B in S1 File, the levels of transcripts of all selected genes were in correspondence with those previously obtained by RNA-seq. Thus, under a FurA depleted background, most of the novel FurA direct targets appeared up-regulated, suggesting a direct repression of FurA under the expression of those genes. By contrast, the genes *alr2495* and *alr4686* were down-regulated, which could suggest a direct activating role of FurA.

To further investigate the role of FurA on the expression of this novel targets, we analyse the transcriptional patterns observed to all of these genes under a FurA overexpression background. Wild-type strain PCC 7120 and the *furA* overexpressing strain AG2770FurA were grown in BG-11 medium under standard growth conditions to mid-log phase, in triplicate cultures. Total RNA was extracted and analysed by sqRT-PCR. The strain AG2770FurA contains a shuttle vector with a copy of gene *furA* under the control of the *petE* promoter [17]. This system allows increasing the expression of FurA in almost 30 times, respecting to the wild-type parental strain PCC 7120 [20]. As shown in Fig 5B and Figure C in S1 File, under a FurA overexpression background most of genes appeared down-regulated, while genes *alr2495* and *alr4686* showed and increase in transcript levels, corroborating a direct activating role of FurA on the expression of these two genes.

In addition, we evaluate the influence of metal co-regulator in the transcription of all novel direct targets. Mid-log growing filaments of the wild-type strain PCC 7120 were exposed to iron restricted conditions as described in Materials and Methods. Transcriptional patterns were compared with those obtained from both the wild-type and the *furA*-overexpressing strain under iron-replete conditions (Fig 5B and Figures D and E in S1 File). As previously observed in EMSA analyses, metal co-regulator appeared essential for a proper regulation of FurA in all of genes analysed. Under iron-restricted conditions, the regulatory activity of FurA was affected in all cases. In genes down-regulated, the transcript levels appeared slightly increased, while in FurA activated targets like *alr2495* and *alr4686* the restriction of the co-regulator led to a decrease in gene expression. The results demonstrated that the co-regulator appeared to be essential for FurA activity in both cases, as repressor and as activator of gene expression.

## Discussion

FurA is the master regulator of iron homeostasis in *Anabaena* sp. PCC 7120 [15, 17, 20]. The goal of the present study was to discern novel regulatory roles of FurA in cyanobacteria beyond those well-recognized targets involved in iron metabolism. The essential role of FurA in the physiology of *Anabaena* sp. impairs classical genetic approaches to study Fur regulatory functions. Among others, several *in vitro*, *in silico*, and *in vivo* analyses on *Anabaena* sp. *furA*-overexpressing strains have been successfully used to expand our knowledge of the FurA regulon [15–17, 20, 22–24]. To date, the efforts to inactivate *furA* in *Anabaena* sp. and other cyanobacteria species had resulted in the creation of heteroallelic mutants expressing mostly the wild-type *fur* gene [29, 30]. Here, we constructed a *coaR*-P_*coaT*_::*furA* fusion strain by the replacement of natural *furA* promoter in the *Anabaena* sp. chromosomes with the Co^2+^/Zn^2+^ inducible *coaT* promoter from *Synechocystis* sp. PCC 6803 [32]. This approach allowed us to selectively regulate the *furA* expression to effectively test the FurA essentiality. By growing the cells with Co^2+^/Zn^2+^ to allow the synthesis of FurA and subsequently removing Co^2+^/Zn^2+^ from the medium to shut off *furA* expression, we could simulate the *furA* null phenotype, or at least, allow a substantial depletion of FurA expression. Wild-type *Anabaena* sp. PCC 7120 was simultaneously grown under the same Co^2+^/Zn^2+^ restricted conditions in all analyses as control of the natural response of the cyanobacterium to this nutrient limitation.

Depletion of FurA expression in the *coaR*-P_*coaT*_::*furA* fusion strain impaired photoautotrophic growth under standard culture conditions. Similar findings were observed in both solidified and liquid media. As we could note in a subsequent genome-wide transcriptome analysis, the depletion of FurA levels induced substantial changes in the pattern expression of a plethora of genes and operons involved in many essential physiological processes including photosynthesis, respiration, detoxification and gene regulation. The results led to conclude that the proper expression of this highly conserved global regulator appears to be essential for the cyanobacterial physiology under standard culture conditions.

Turning off *furA* induced differential expression in approximately 33% of the *Anabaena* sp. genome, which could indicate the total effect of both direct and indirect regulation of FurA. Curiously, the depletion of FurA levels, which mainly acts as a transcriptional repressor, mostly caused down-regulation of gene expression. This was the case of main genes involved in iron uptake (Table D in S1 File). Previous reports have shown that, as a general trend, the transcription levels of the main components of the iron transport system in *Anabaena* is repressed under iron sufficient condition, while iron limitation causes up-regulation of this machinery [17, 35–37]. In heterotrophic bacteria, *fur* mutants usually exhibit derepression of iron uptake mechanisms, even under iron sufficient condition [38–40]. Our results seem to suggest that in addition to FurA, other yet unidentified iron-responsive regulators are likely modulating iron homeostasis in *Anabaena* sp. PCC 7120, as occurs in *Synechocystis* sp. PCC 6803, where the PfsR regulator plays a crucial role in this process [41]. It is also remarkable that differential expressed products were mainly enriched by genes involved in regulatory functions, including dozens of other transcriptional regulators, two-component signal transduction systems, and other regulatory players. Hence, the effect of FurA depletion observed in the *coaR*-P_*coaT*_::*furA* fusion strain goes beyond the direct transcripcional regulation exerted by FurA and reflects both the range and complexity of the FurA regulatory network.

The RNA-seq data showed differential expression of numerous genes that had not been previously identified as iron-responsive or FurA targets in cyanobacteria, including several genes related to important little studied processes such as chlorophyll catabolism and programmed death, phycobilisome degradation, exopolysaccharide biosynthesis, detoxification, light sensing and response, biosynthesis of Fe-S cluster, among others. Thus, among all differential expressed genes with known function, we selected 60 putative FurA targets and determine whether the expression of these genes was directly regulated by FurA according to EMSA. Fifteen of these further studied genes resulted FurA novel direct targets.

As expected, most of the novel direct targets described here appeared to be up-regulated under a FurA depletion background, while diminished their expression under a *furA*- overexpressing phenotype, suggesting a repressing function of FurA on the transcription of these genes. Among these FurA repressed targets, we found 4 genes directly involved in detoxification and defences against oxidative stress, including the sulfiredoxin SrxA (Asl4146) [42], the Mn-catalase KatB (Alr3090) [43], the glutaredoxin-related protein Alr0799, and the mercuric reductase All5185.

The intimate relationship between iron metabolism and oxidative stress has been extensively recognized in bacteria [2, 44, 45]. In *E*. *coli*, the expression of Fur is modulated by oxidative stress response regulators, and Fur directly or indirectly regulates the expression of antioxidant enzymes. Our findings further support the role of FurA as a key player in the regulation of defences against oxidative stress in *Anabaena* sp. PCC 7120. Previous analyses have identified other FurA targets belonging to the first line of defences against oxidative stress, including the peroxiredoxins Alr4641 and All1541 [24] and the flavodiiron protein Flv3 [20]. To date, all FurA targets involved in the response to oxidative stress appeared repressed by the regulator. In all cases, the modulation of gene expression depended of both, the presence of metal co-regulator and reducing conditions. Taking together, the results suggest that the antioxidant defences regulated by FurA in *Anabaena* sp. PCC 7120 are repressed by the regulator under iron-sufficient conditions, and those become up-regulated under iron deprivation. In fact, iron starvation leads to significant increase in ROS and induces oxidative stress in cyanobacteria [45], and this condition is the most abundant in nature. Other ferric uptake regulator paralogues described in *Anabaena* sp., like FurB or FurC, have also been associated to defences against oxidative stress [19, 36, 46]. Since the three Fur homologues resulted up-regulated in minor or major grade as a response to damage by ROS [19, 36], we could speculate the combined action of these regulators in the generation of a concerted response to oxidative stress in cyanobacteria. The implication of other Fur orthologues in modulation of gene expression as response of oxidative stress signals have been previously documented [47–50].

Our RNA-seq analysis and subsequent EMSA experiments led to identify an important novel FurA direct target, the phycobilisome degradation protein NblA (Asr4517) [51]. Phycobilisomes are the major light-harvesting complexes of the photosynthetic apparatus of cyanobacteria [52]. These multi-protein complexes, which can constitute up to 50% of the total cellular protein, are rapidly degraded when the microorganisms are starved for different nutrients, including combined nitrogen [53], sulphur, phosphorus [54], and iron [55], leading to a dramatic bleaching phenomenon known as chlorosis. NblA, a 59 amino acid polypeptide which is induced upon starvation, results essential for phycobilisome degradation [56]. Previous studies have shown that the *nblA* gene is differentially regulated by iron availability in *Synechocystis* sp. PCC 6803 [57]. Notably, *Anabaena* sp. PCC 7120 has two *nblA* homologues, one on the chromosome (*asr4517*) and one on plasmid delta (*asr8504*). Only the chromosomal *nblA* gene, which is up-regulated upon nitrogen starvation [53], was regulated by FurA.

We have previously documented that FurA influences heterocyst differentiation [20, 22]. Heterocyst development and its pattern formation are developmentally regulated processes, involving the coordinated action of several transcriptional regulators which orchestrated a complex regulatory cascade [58, 59]. Our previous analyses have shown that expression of *furA* is strongly induced by the global regulator of nitrogen metabolism NtcA in proheterocysts during the first 15 h after nitrogen step-down, remaining stably expressed in mature heterocysts [60]. On the other hand, *in vitro*, *in silico* and *in vivo* analyses have shown that FurA acts as a transcriptional modulator of *ntcA* [22], *abp1* [24], *hetC*, *patA*, *alr1728* and *asr1734* [20]. Here, we identify another FurA direct target which plays a central role in the heterocyst development regulation, the calcium-binding protein CcbP [61]. A rapid increase of intracellular Ca^2+^ concentration due to the decreased expression of *ccbP* is observed in proheterocysts as soon as 4 hours after nitrogen deprivation. The *ccbP* message is down-regulated in differentiating cells and absent in mature heterocysts. It has been hypothesized that a regulatory pathway consisting of HetR, CcbP, and NtcA controls intracellular free calcium during heterocyst development [58]. The results presented here could suggest a role of FurA in repressing the *ccbP* expression in differentiating cells and mature heterocysts.

Our results underlined the role of FurA in controlling light-dependent signal transduction mechanisms. Sensing light signals and their subsequent transduction is essential for photosynthetic organisms, since it enables them to adapt to variable environmental conditions [62]. In previous studies [20], predicted and functionally validated FurA binding sites were determined in the promoter regions of the *Anabaena* sensory rhodopsin Asr [63] and the phytochrome-like protein AphC [64]. Here we identified the phytochrome-like protein Alr3356 as a novel FurA direct target. As AphC, the protein Alr3356 is a sensory photoreceptor characterized by the presence of a chromophore-binding GAF domain that is homologous but distinct to the tetrapyrrole-binding GAF domain of the phytochromes, and belongs to the family of cyanobacteriochromes [65, 66]. These findings could indicate that FurA might modulate certain light-dependent regulatory mechanisms based on iron availability or the redox status of cell.

New regulatory roles of FurA were also observed in less studied cyanobacterial processes such as chlorophyll catabolism and exopolysaccharide biosynthesis. Little is known about programmed death in cyanobacteria. In plants, leaf senescence is a genetically programmed process regulated by specific genes [67]. During this process, chlorophyll is degraded to colorless linear tetrapyrroles, termed nonfluorescent chlorophyll catabolites [68]. Exposition to large iron starvation periods could trigger chlorophyll catabolism and programmed death in cyanobacteria via FurA. Further studies will be required to verify this hypothesis.

Similarly, little is known regarding the cyanobacterial exopolysaccharides biosynthetic pathways and regulating factors. Many cyanobacteria strains have polysaccharidic structures surrounding their cells [69]. The ability to synthesize external polysaccharide layers confers the cells protection from unfavourable environmental conditions including dehydration, exposition to high UV radiation and biomineralization. In addition, the presence of negatively charged polysaccharidic layers surrounding cyanobacterial cells may play an important role in the sequestration of metal cations, and in creating a microenvironment enriched in those metals that are essential for cell growth but are present at very low concentrations in some environments. Recent studies demonstrated that cyanobacterial exopolysaccharides play a role in Fe-sorption, which protects *Synechocystis* sp. PCC 6803 against iron starvation [70]. Thus, in iron-limited environment FurA could modulate the expression of cyanobacterial exopolysaccharide in order to enhance metal sequestration.

Two genes, *alr2495* and *alr4686*, decreased their transcript levels under a FurA depletion background, while appeared up-regulated in the *furA*-overexpressing strain. These results could indicate that two novel metabolic pathways appeared to be transcriptionally activated by FurA under iron-replete conditions: (1) the biosynthesis of the Fe-S cluster through the cysteine desulphurase SufS (Alr2495), and (2) the biosynthesis of sesquiterpene through the cytochrome P450 (Alr4686). These evidences further support the role of FurA as a dual regulator, acting both as repressor and activator of gene expression. Previous studies have shown other possible examples of direct positive modulation exerted by FurA [15, 20]. Despite Fur-mediated direct activation as been observed in several heterotrophic bacteria [5, 6, 71, 72], little is known about the mechanism or mechanisms by which Fur proteins function to directly activate gene transcription. In *Neisseria gonorrhoeae* [71], Fur-mediated activation occurs by competition to another repressor for overlapping binding sites, resulting in derepression of transcription. Interestingly, the locations of the Fur boxes in the promoters of these Fur-activated genes were close to the -10 and -35 motifs, which could suggest not only the competition with other repressors, but also the possibility of RNA polymerase recruitment to enhance transcription initiation. When Fur boxes are located far upstream of the transcription start site, Fur might activate gene expression by altering DNA morphology allowing or enhancing RNA polymerase binding [5].

In summary, the results presented here provide new insights into the complex regulatory network orchestrated by the essential cyanobacterial global regulator FurA. RNA-seq based transcriptome analysis employing a selectively regulating *furA* expression system led to define 15 novel direct iron-dependent transcriptional targets belonging to a variety of functional categories including detoxification and defences against oxidative stress, phycobilisome degradation, chlorophyll catabolism and programmed death, light sensing and response, exopolysaccharide biosynthesis, among others. Our analyses unravel the role of FurA as a global transcriptional regulator in cyanobacteria, acting both as repressor and activator of gene expression. In either case, the *in vivo* FurA-mediated regulation seems to be dependent of the environmental iron availability as well as the intracellular redox status.

## Materials and Methods

### Strains and culture conditions

Bacterial strains and plasmids used in this study are described in Table E in S1 File. Wild-type strain *Anabaena* sp. PCC 7120 and its derivatives were routinely grown in BG-11 medium as described previously [20]. Culture medium was supplemented with neomycin 50 μg ml^-1^ (Nm) in the case of strains AG2770FurA and AGcoaRFurA. Chlorophyll a (Chl) was determined in methanol extracts [73].

For cobalt/zinc restricted cultures, *Anabaena* sp. exponentially growing cells (3–5 μg Chl ml^-1^) from standard BG-11 were harvested by filtration, washed three times with BG-11 medium without Co^2+^/Zn^2+^ (BG-11_-Co/Zn_), re-suspended in the same Co^2+^/Zn^2+^-free medium and further grown for 48 h. For iron depletion experiments, exponentially growing filaments from standard BG-11 were collected by filtration, washed three times with BG-11 medium without iron (BG-11_-Fe_), re-suspended in the same iron-free medium and further grown for 72 h. All the glassware used in the experiments of metal restriction growth was soaked with 6M HCl and extensively rinsed with Milli-Q water to remove residual metal ions.

*Escherichia coli* strains carrying plasmids were grown in Luria broth supplemented, as appropriate, with 50 μg ml^-1^ kanamycin (Km), 50 μg ml^-1^ ampicillin (Amp) and/or 34 μg ml^-1^ chloramphenicol (Cm).

### DNA manipulations

Cyanobacterial genomic DNA was isolated as described previously [74]. Plasmid preparations were performed using the GenElute^™^ Plasmid Miniprep Kit (Sigma). DNA purification of PCR products was performed using the GFX^™^ PCR DNA and the Gel Band Purification Kit (GE Healthcare). Standard protocols were used for cloning, *E*. *coli* transformation and PCR [75]. The sequences of all oligonucleotides used in plasmid constructions are described in Table F in S1 File.

The suicide plasmid pCoaRFurA, which contains a copy of the gene *furA* under the control of the *Synechocystis* sp. Co^2+^/Zn^2+^ inducible *coaT* promoter [32], was constructed in several steps (Figure F in S1 File): (i) The native 2-kb sequence from the upstream region of the *furA* gene was amplified from the *Anabaena* sp. PCC 7120 genomic DNA using primers PpetEfurA1 and PpetEfurA2, and cloned into pGEM-T vector (Promega) to generate pCoaR1. (ii) A sequence from upstream of the *coaT* gene containing the *coaT* promoter and the *coaR* regulator gene was amplified from the genomic DNA of *Synechocystis* sp. PCC 6803 using the primers 2770furA-coaR-P_up and 2770furA-coaR-P_dw. The resulting PCR product was digested with *Ava*I and *Nde*I, and inserted between the same restriction sites of vector pAM2770FurA [17] to create pCoaR2. (iii) Plasmid pCoaR3 was generated by moving the *coaR*-P_*coaT*_::*furA* fusion fragment from pCoaR2 to pCoaR1. (iv) The PCR product generated with primers PpetEfurA3 and PpetEfurA4, which contained the native 2-kb sequence located immediately downstream of the *furA* gene in the *Anabaena* sp. PCC 7120 genome, was cloned into pGEM-T (pCoaR4), excised with *BamH*I-*Sac*I and ligated into the same sites of pCoaR3 to create pCoaR5. (v) The Km/Nm-resistance transcription unit was amplified from pAM2770FurA [17] and cloned into the *Ava*I site of pCoaR5 to generate pCoaR6. (vi) Finally, the *Bgl*II-*Sac*I fragment from pCoaR6 was ligated into the same site of pRL278 [76] to create the suicide vector pCoaRFurA. The final vector was partially sequenced to ensure that no modifications in the nucleotide sequence occurred during PCR and cloning.

### Construction of *Anabaena* sp. strain AGcoaRFurA

Plasmid pCoaRFurA was transferred to *Anabaena* sp. PCC 7120 by conjugation according to a previously described method [77]. Triparental mating was carried out using the *E*. *coli* conjugal donor strain ED8654, which contains the plasmid pRL443, and the *E*. *coli* conjugal helper strain CPB1893 carrying the plasmid pRL623. Since replacement of native *furA* promoter by the Co^2+^/Zn^2+^ inducible *coaT* promoter in the *Anabaena* sp. chromosomes occurred by double homologous recombination events, double recombinants were selected by a *sacB*-mediated method of positive selection [74]. Briefly, several exconjugant clones resistant to neomycin were successively grown in liquid culture before the cyanobacterial filaments were fragmented to mostly single cells using a Branson 1200 ultrasonic cleaner. The cells were subsequently plated on BG-11 containing 5% sucrose to select for cells in which resolution of the plasmid resulted in loss of the conditional lethal gene *sacB*, which was part of the plasmid. Complete segregation of *coaR*-P_*coaT*_::*furA* fusion was confirmed by PCR.

### SDS-PAGE and Western blotting

Filaments of *Anabaena* sp. strains from 50 ml mid-log phase cultures were collected by centrifugation, washed with 50 mM Tris-HCl buffer (pH 8.0) and sonicated in an ice-water bath by five 30 s bursts with 30 s cooling intervals. The resulting crude extracts were centrifuged at 12 000 × *g* for 5 min at 4°C to remove cell debris, and protein concentration was determined using the BCA^™^ Protein Assay kit (Thermo Fisher Scientific). For each sample, 30 μg of total protein was loaded and separated by SDS-PAGE 17% polyacrylamide gel, and transferred to a polyvinylidine fluoride membrane (Millipore). Rabbit polyclonal antibodies raised against *Anabaena* sp. FurA recombinant protein were used, and the blot was visualized with an Universal Hood Image Analyser (Bio-Rad).

### RNA sequencing and data analysis

Total RNA from Co^2+^/Zn^2+^ restricted cultures of *Anabaena* sp. wild-type and AGcoaRFurA treated as described previously (see Strains and culture conditions) were extracted in triplicate using the RNeasy Mini Kit (Qiagen). The triplicate samples of each strain were mixed for RNA sequencing. Ribosomal RNA was removed using the RiboMinus Transcriptome Isolation Kit (Thermo Fisher Scientific). RNA sequencing was performed on the Illumina Miseq desktop sequencer (Illumina) at STAB VIDA, Lda (Lisbon, Portugal), following the instructions of the manufacturer.

A total of 11,124,194 reads were obtained for both *Anabaena* sp. strains. The data were deposited in the NCBI Short Read Archive under accession SRP066361. Details about the mapping statistics of the individual libraries are provided in Table G in S1 File. Percents of Q30 bases were used as quality criteria. Short reads were aligned against the complete genome of *Anabaena* sp. PCC 7120 and plasmids pCC7120alpha, pCC7120beta, pCC7120gamma, pCC7120delta, pCC7120epsilon and pCC7120zeta (GenBank accession codes: NC_003272.1, NC_003276.1, NC_003240.1, NC_003267.1, NC_003273.1, NC_003270.1 and NC_003241.1, respectively) using Bow tie [78], allowing a maximum of three mismatches within the first 50 bases. The uniquely mapped reads were collected and analyzed with the DEG-seq package [79] to identify differentially expressed genes. The results of gene transcription were based in the fold-changes of RPKM (Reads Per Kilobase of transcript per Million mapped reads) [80], similarly to other previously published studies [81]. The significance of the expression was calculated using a proportions-based test [82], applicable in situations where the data sample consists of counts of a number of 'types' of data. Expression data were normalized against the housekeeping gene *ilvD* (*alr2771*) [83].

### Electrophoretic mobility shift assays

Recombinant *Anabaena* sp. PCC 7120 FurA protein was expressed in *E*. *coli* BL21 (DE3) (EMD Biosciences) and purified according to previously described methods [84]. The promoter regions of each gene of interest were obtained by PCR using the primers described in Table F in S1 File. Electrophoretic mobility shift assays (EMSA) were performed as described previously [34]. Briefly, 120–140 ng of each DNA fragment were mixed with recombinant FurA protein at concentrations of 300, 500 and 700 nM in a 20 μl reaction volume containing 10 mM bis-Tris (pH 7.5), 40 mM potassium chloride, 100 μg/ml bovine serum albumin, 1 mM DTT, 100 μM manganese chloride and 5% glycerol. In some experiments, EDTA was added to a final concentration of 200 μM. To ensure the specificity of EMSA, the promoter region of *Anabaena* sp. *nifJ* (*alr1911*) gene was included as non-specific competitor DNA in all assays [17] The amount of competitor DNA (90–140 ng) was optimized in previous assays in order to detect unspecific binding of FurA without great molar excess. Mixtures were incubated at room temperature for 20 min, and subsequently separated on 4% non-denaturing polyacrylamide gels in running buffer (25 mM Tris, 190 mM glycine) at 90 V. Gels were stained with SYBR^®^ Safe DNA gel stain (Invitrogen) and processed with a Gel Doc 2000 Image Analyzer (Bio-Rad).

### Semi-quantitative reverse transcription-PCR

Samples of 1 μg RNA were heated at 85°C for 10 min and used as templates for the first-strand cDNA synthesis. Residual DNA in RNA preparations was eliminated by digestion with RNase-free DNase I (Roche). The absence of DNA was checked by PCR. Reverse transcription was carried out using SuperScript retrotranscriptase (Invitrogen) in a 20 μl reaction volume containing 150 ng of random primers (Invitrogen), 1 mM dNTP mix (GE Healthcare) and 10 mM DTT. The sequences of the specific primers used for semi-quantitative reverse transcription-PCR (sqRT-PCR) reactions are defined in Table F in S1 File. Housekeeping gene *rnpB* [17] was used as a control to compensate for variations in the input of RNA amounts and normalize the results. Exponential phase of PCR for each gene was determined by measuring the amount of PCR product at different time intervals. For the final results, 20–23 cycles at the early exponential phase were used in all genes analyzed. The PCR products were resolved by electrophoresis in 1% agarose gels, stained with ethidium bromide and analyzed using a Gel Doc 2000 Image Analyzer (Bio-Rad).

Relative induction ratio for each gene was calculated as the average of ratios between signals observed in two environmental conditions of interest in three independent determinations. Signal assigned to each gene corresponded to the intensity of its DNA band in the agarose gel stained with ethidium bromide normalized to the signal observed for the housekeeping gene *rnpB* in each strain.

## Supporting Information

S1 File Figure A: Expression of FurA results essential to the growth of *Anabaena* sp. under standard culture conditions. Figure B: Fold changes in the expression of FurA direct targets in the *furA*-turning off strain *Anabaena* sp. AGcoaRFurA after cobalt/zinc deprivation as compared with the wild-type PCC 7120 strain under the same growth condition, as result of semi-quantitative RT-PCR analyses. Figure C: Fold changes in the expression of FurA direct targets in the *furA*-overexpressing strain *Anabaena* sp. AG2770FurA under iron-replete condition (BG-11 medium) as compared with the wild-type PCC 7120 strain under the same growth condition, as result of semi-quantitative RT-PCR analyses. Figure D: Fold changes in the expression of FurA direct targets in the wild-type strain *Anabaena* sp. PCC 7120 after iron deprivation as compared with the same strain under iron-replete condition (BG-11 medium), as result of semi-quantitative RT-PCR analyses. Figure E: Fold changes in the expression of FurA direct targets in the *furA*-overexpressing strain *Anabaena* sp. AG2770FurA under iron-replete condition (BG-11 medium) as compared with the wild-type PCC 7120 strain grown under iron deprivation, as result of semi-quantitative RT-PCR analyses. Figure F: Construction of *Anabaena* sp. strain AGcoaRFurA. Table A: Protein sequences producing significant alignments (≥80% identity) with FurA from *Anabaena* sp. PCC 7120. Table B: Complete list of genes showing ≥2-fold change in expression in the *furA*-turning off strain AGcoaRFurA. Table C: Genes showing <2-fold change in expression in the *furA*-turning off strain AGcoaRFurA. Table D: Main genes involved in iron uptake showing ≥2-fold change in expression in the *furA*-turning off strain AGcoaRFurA. Table E: Strains and plasmids used in this study. Table F: Oligonucleotides used in this study. Table G: Summary of sequencing run statistics
(PDF)
