# Supplementary material for: Expanding the Role of FurA as Essential Global Regulator in Cyanobacteria
Source: PLoS One. 2016 Mar 11;11(3):e0151384. doi: 10.1371/journal.pone.0151384 (PMC4788461; doi:10.1371/journal.pone.0151384)
Supplement: S1 File — Figure A: Expression of FurA results essential to the growth of Anabaena sp. under standard culture conditions. Figure B: Fold changes in the expression of FurA direct targets in the furA-turning off strain Anabaena sp. AGcoaRFurA after cobalt/zinc deprivation as compared with the wild-type PCC 7120 strain under the same growth condition, as result of semi-quantitative RT-PCR analyses. Figure C: Fold changes in the expression of FurA direct targets in the furA-overexpressing strain Anabaena sp. AG2770FurA under iron-replete condition (BG-11 medium) as compared with the wild-type PCC 7120 strain under the same growth condition, as result of semi-quantitative RT-PCR analyses. Figure D: Fold changes in the expression of FurA direct targets in the wild-type strain Anabaena sp. PCC 7120 after iron deprivation as compared with the same strain under iron-replete condition (BG-11 medium), as result of semi-quantitative RT-PCR analyses. Figure E: Fold changes in the expression of FurA direct targets in the furA-overexpressing strain Anabaena sp. AG2770FurA under iron-replete condition (BG-11 medium) as compared with the wild-type PCC 7120 strain grown under iron deprivation, as result of semi-quantitative RT-PCR analyses. Figure F: Construction of Anabaena sp. strain AGcoaRFurA. Table A: Protein sequences producing significant alignments (≥80% identity) with FurA from Anabaena sp. PCC 7120. Table B: Complete list of genes showing ≥2-fold change in expression in the furA-turning off strain AGcoaRFurA. Table C: Genes showing <2-fold change in expression in the furA-turning off strain AGcoaRFurA. Table D: Main genes involved in iron uptake showing ≥2-fold change in expression in the furA-turning off strain AGcoaRFurA. Table E: Strains and plasmids used in this study. Table F: Oligonucleotides used in this study. Table G: Summary of sequencing run statistics (PDF) [file pone.0151384.s001.pdf]

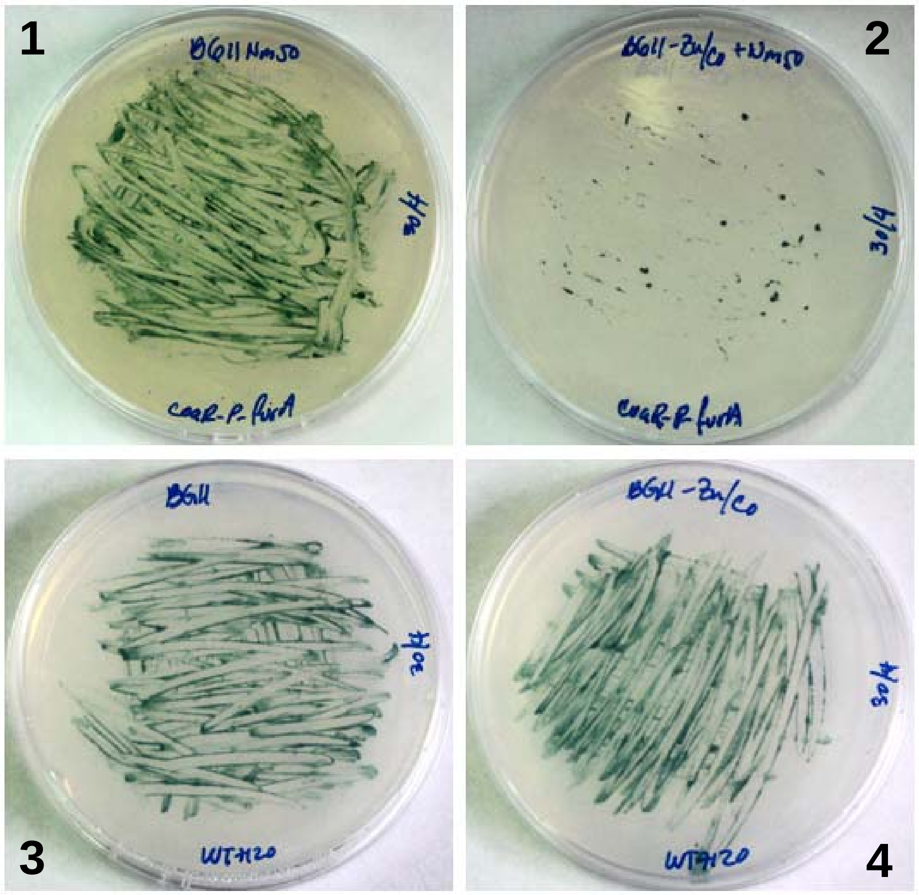

Figure A. Expression of FurA results essential to the growth of *Anabaena* sp. under standard culture conditions.
The *coaR*-P_{coaT}:: *furA* fusion strain *Anabaena* sp. AGcoaRFurA (1) and the wild-type strain *Anabaena* sp. PCC 7120 (3) grew abundantly in standard BG-11 medium solidified with 1% Noble agar, forming a confluent grow on the agar in 10-14 days. The elimination of cobalt and zinc from de culture medium impaired growth of the *coaR*-P_{coaT}:: *furA* fusion strain (2), but not in the case of the wild-type strain (4).

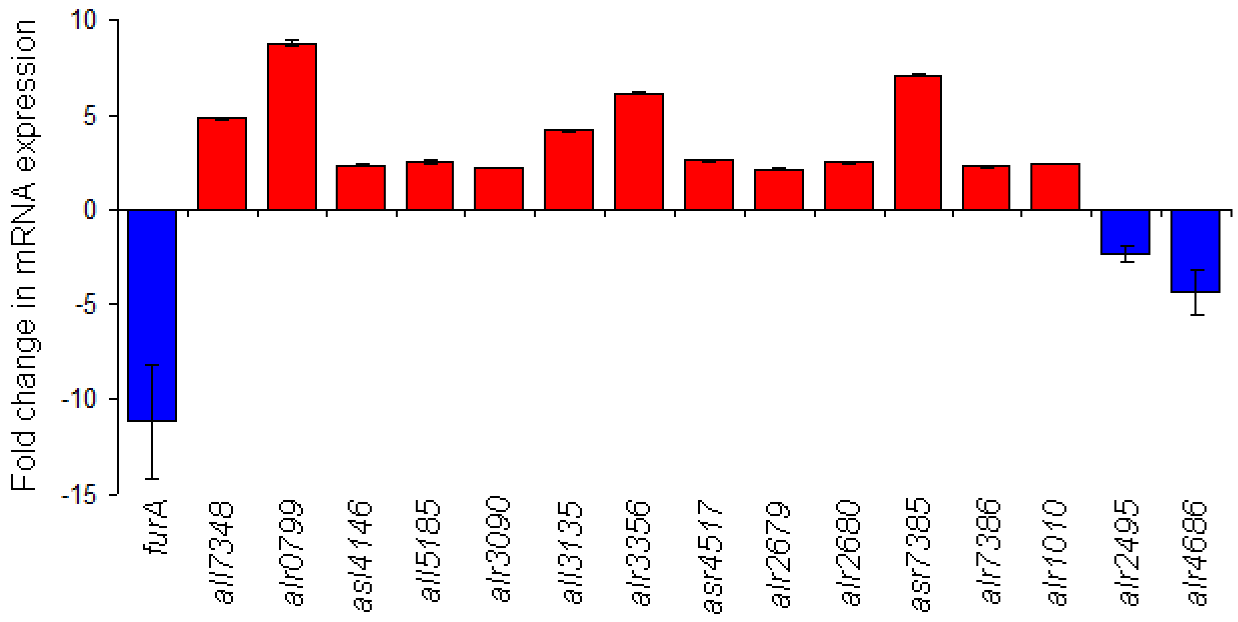

Figure B. Fold changes in the expression of FurA direct targets in the *furA*-turning off strain *Anabaena* sp. AGcoaRFurA after cobalt/zinc deprivation as compared with the wild-type PCC 7120 strain under the same growth condition, as result of semi-quantitative RT-PCR analyses.

Signal assigned to each gene corresponded to the intensity of DNA band in the agarose gel stained with ethidium bromide, normalized to the signal observed for housekeeping gene *rnpB* in each condition. Values are means of three independent determination ± SD.

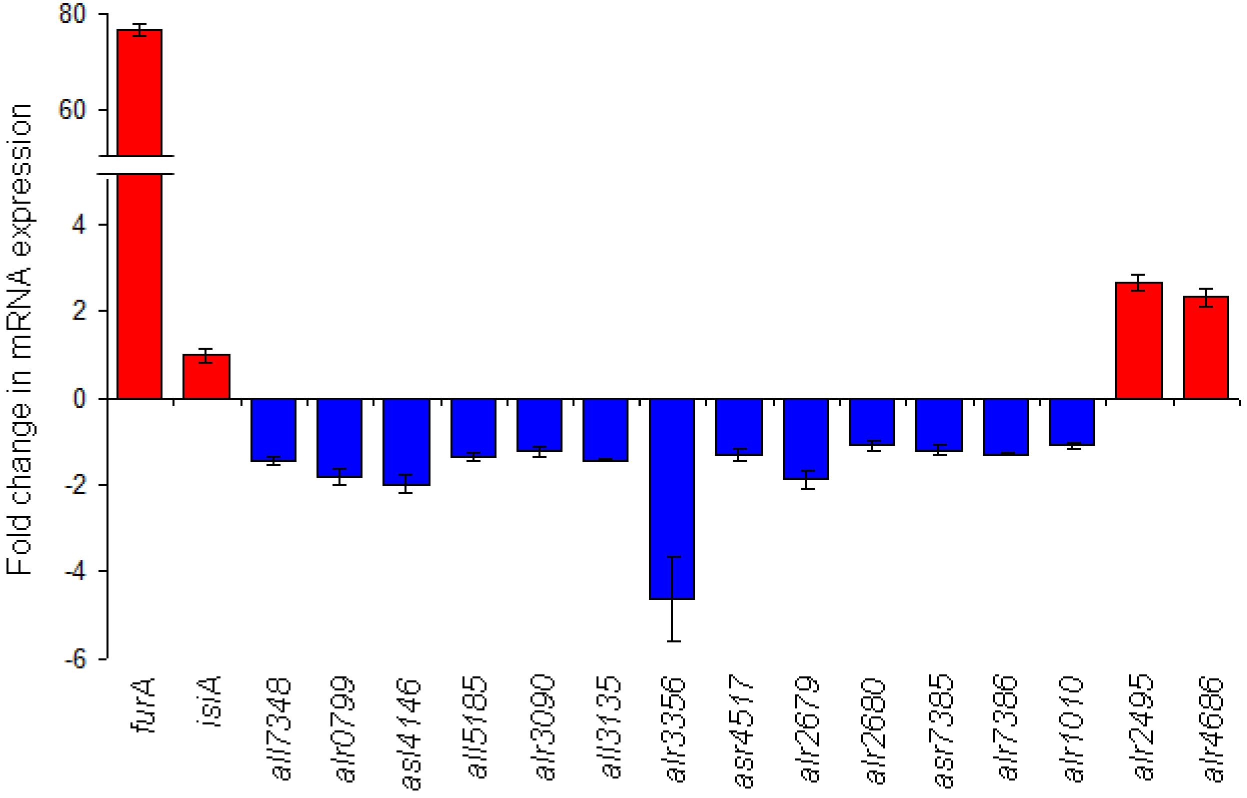

Figure C. Fold changes in the expression of FurA direct targets in the *furA*-overexpressing strain *Anabaena* sp. AG2770FurA under iron-replete condition (BG-11 medium) as compared with the wild-type PCC 7120 strain under the same growth condition, as result of semi-quantitative RT-PCR analyses.

Signal assigned to each gene corresponded to the intensity of DNA band in the agarose gel stained with ethidium bromide, normalized to the signal observed for housekeeping gene *rnpB* in each condition. Values are means of three independent determination ± SD.

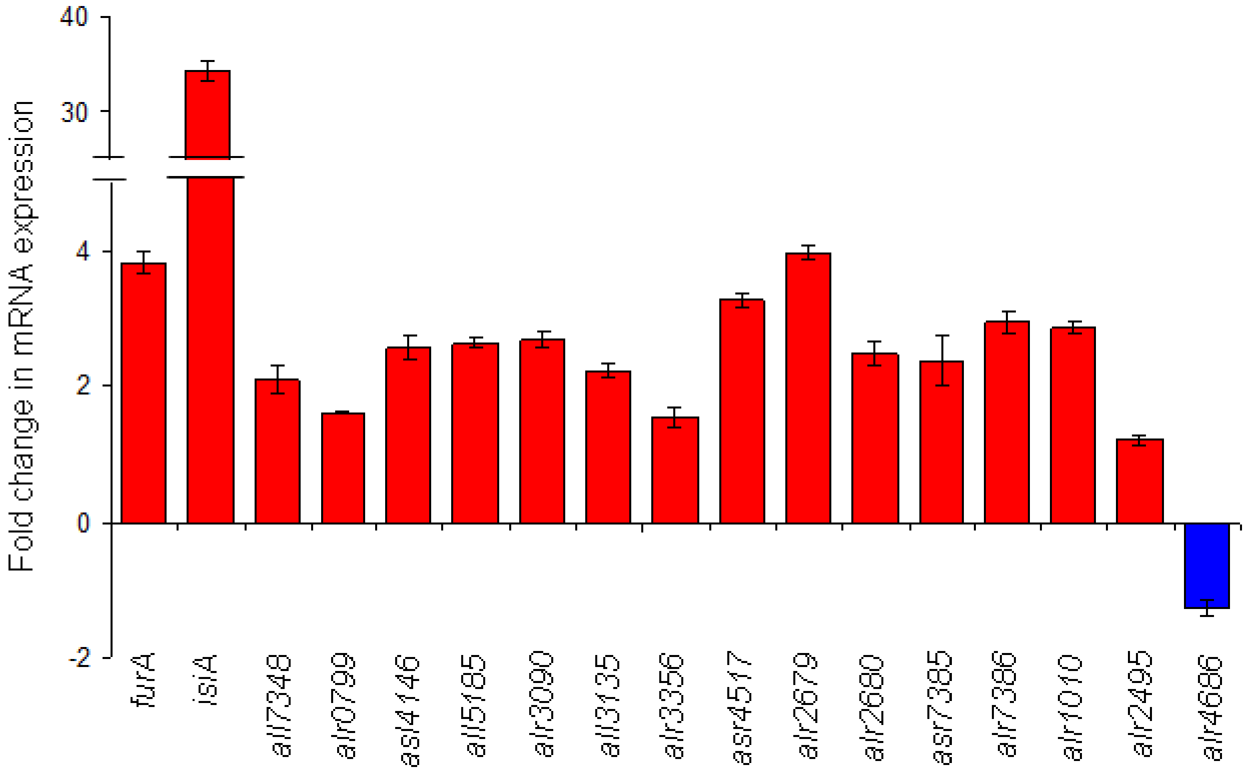

Figure D. Fold changes in the expression of FurA direct targets in the wild-type strain *Anabaena* sp. PCC 7120 after iron deprivation as compared with the same strain under iron-replete condition (BG-11 medium), as result of semi-quantitative RT-PCR analyses.
Signal assigned to each gene corresponded to the intensity of DNA band in the agarose gel stained with ethidium bromide, normalized to the signal observed for housekeeping gene *rnpB* in each condition. Values are means of three independent determination ± SD.

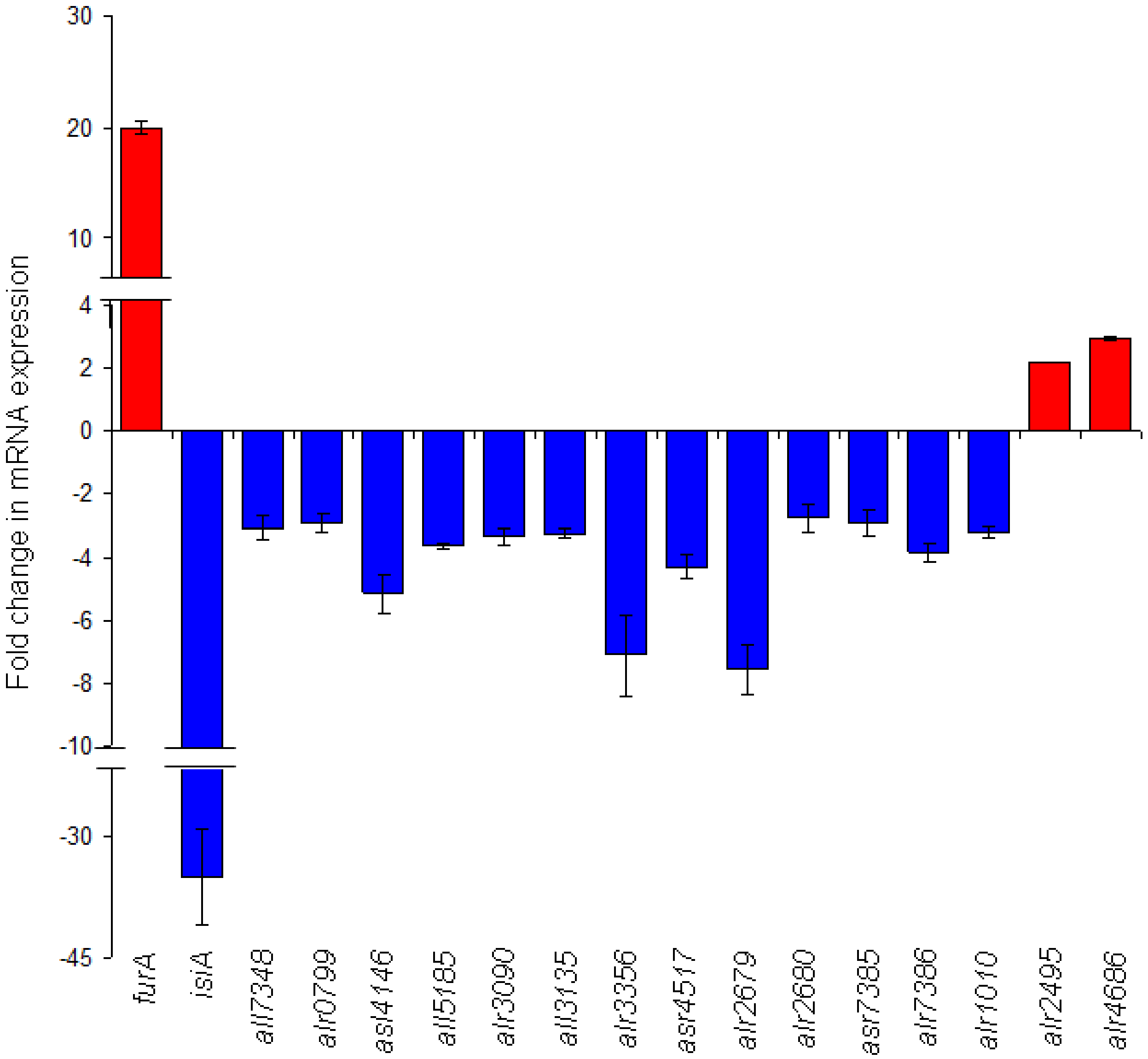

Figure E. Fold changes in the expression of FurA direct targets in the *furA*-overexpressing strain *Anabaena* sp. AG2770FurA under iron-replete condition (BG-11 medium) as compared with the wild-type PCC 7120 strain grown under iron deprivation, as result of semi-quantitative RT-PCR analyses.

Signal assigned to each gene corresponded to the intensity of DNA band in the agarose gel stained with ethidium bromide, normalized to the signal observed for housekeeping gene *rnpB* in each condition. Values are means of three independent determination ± SD.

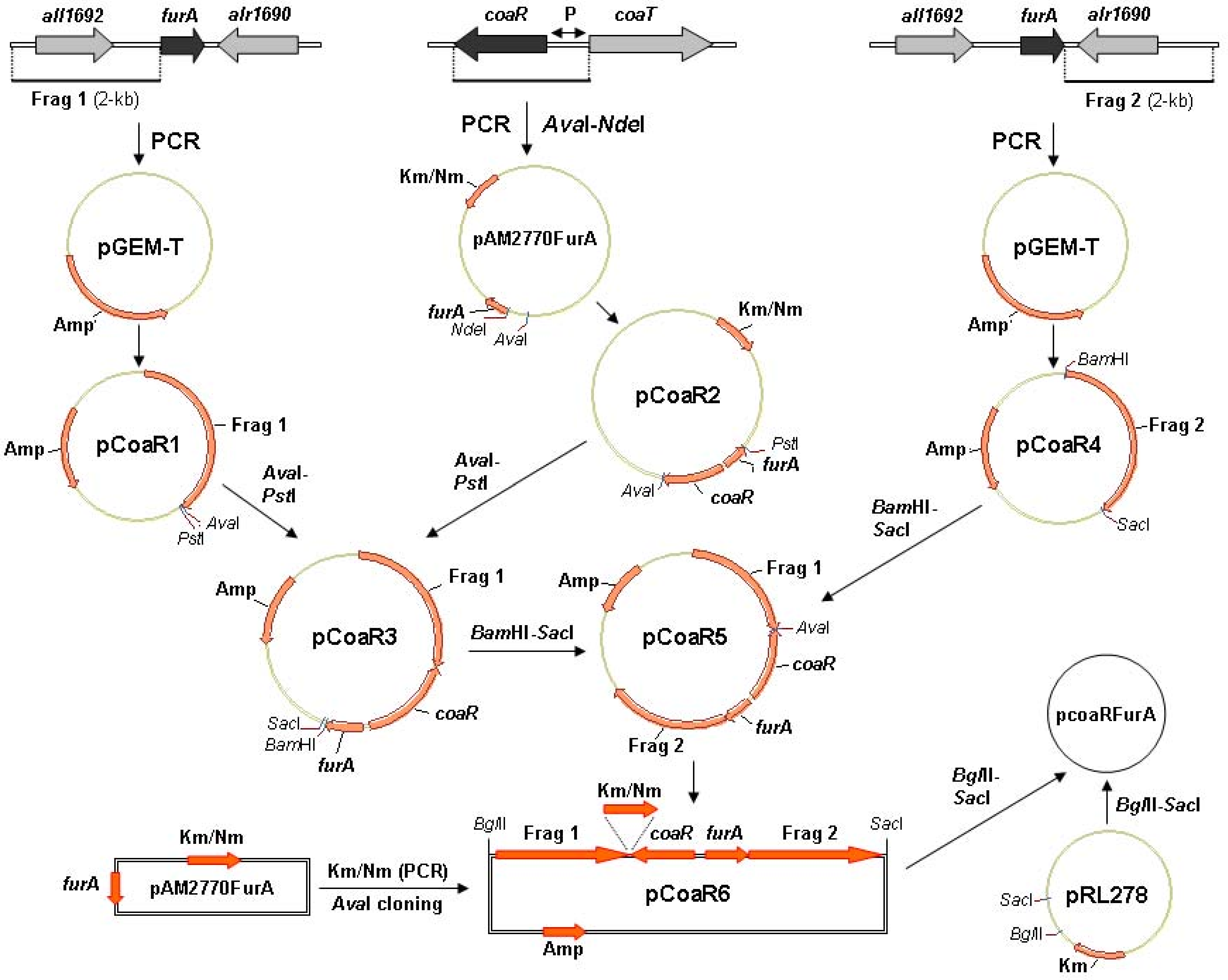

Figure F. Construction of *Anabaena* sp. strain AGcoaRFurA

Table A. Protein sequences producing significant alignments (≥80% identity) with FurA from *Anabaena* sp. PCC 7120[a]

Description	Max score	Total score	Query cover	E value	Identity	NCBI Accession	Cyanobacteria family
MULTISPECIES: Fur family transcriptional regulator [*Nostocaceae*]	314	314	100%	3e-109	100%	WP_010995860.1	*Nostocaceae*
Fur family transcriptional regulator [*Nodularia spumigena*]	310	310	100%	1e-107	98%	WP_006197697.1	*Nostocaceae*
Fur family transcriptional regulator [*Nostoc punctiforme*]	308	308	100%	8e-107	97%	WP_012407750.1	*Nostocaceae*
ferric uptake regulator family protein [*Nostoc* sp. PCC 7107]	307	307	100%	2e-106	97%	WP_015115240.1	*Nostocaceae*
Fe^{2+}/Zn^{2+} uptake regulation protein [*Nostoc* sp. PCC 7524]	305	305	100%	8e-106	97%	WP_015136707.1	*Nostocaceae*
Fe^{2+}/Zn^{2+} uptake regulation protein [*Cylindrospermum stagnale*]	305	305	100%	1e-105	97%	WP_015208849.1	*Nostocaceae*
Fur family transcriptional regulator [*Aphanizomenon flos-aquae*]	302	302	100%	1e-104	95%	WP_027404555.1	*Nostocaceae*
ferric uptake regulator family protein [*Anabaena* sp. 90]	301	301	100%	3e-104	95%	WP_015080256.1	*Nostocaceae*
Fur family transcriptional regulator [*Dolichospermum circinale*]	301	301	100%	3e-104	95%	WP_028084223.1	*Nostocaceae*
ferric uptake regulator family protein [*Calothrix* sp. PCC 7507]	302	302	100%	2e-104	94%	WP_015129343.1	*Rivulariaceae*
Fur family transcriptional regulator [*Microchaete* sp. PCC 7126]	301	301	100%	3e-104	94%	WP_017653552.1	*Microchaetaceae*
ferric uptake regulator family protein [*Calothrix parietina*]	300	300	100%	9e-104	94%	WP_015198567.1	*Rivulariaceae*
Fur family transcriptional regulator [*Anabaena* sp. PCC 7108]	299	299	100%	2e-103	94%	WP_016951304.1	*Nostocaceae*
ferric uptake regulator, Fur family [*Anabaena cylindrica*]	298	298	100%	4e-103	94%	WP_015214948.1	*Nostocaceae*
Fur family transcriptional regulator [*Fischerella* sp. PCC 9605]	301	301	100%	7e-104	93%	WP_026735411.1	*Fischerellaceae*
Fur family transcriptional regulator [*Trichormus azollae*]	296	296	100%	6e-102	93%	WP_013190079.1	*Nostocaceae*
Fur family transcriptional regulator [*Fischerella muscicola*]	298	298	100%	5e-103	93%	WP_016870366.1	*Fischerellaceae*
Fur family transcriptional regulator [*Fischerella* sp. PCC 9431]	297	297	100%	1e-102	92%	WP_026722649.1	*Fischerellaceae*
Fur family transcriptional regulator [*Calothrix* sp. PCC 7103]	295	295	100%	1e-101	92%	WP_019493338.1	*Rivulariaceae*
Fur family transcriptional regulator [*Mastigocladopsis repens*]	293	293	100%	9e-101	92%	WP_017316719.1	*Symphyonemataceae*
Fur family transcriptional regulator [*Calothrix* sp. 336/3]	275	275	100%	1e-93	92%	KFB85589.1	*Rivulariaceae*

Fur family transcriptional regulator [[*Scytonema hofmanni*] UTEX 2349]	297	297	100%	2e-102	91%	WP_029637742.1	*Scytonemataceae*
MULTISPECIES: Fur family transcriptional regulator [*Fischerella*]	295	295	100%	1e-101	91%	WP_009458621.1	*Fischerellaceae*
Fur family transcriptional regulator [*Fischerella* sp. PCC 9339]	296	296	100%	4e-102	91%	WP_017309854.1	*Fischerellaceae*
Fur family transcriptional regulator [*Chlorogloeopsis* sp. PCC 7702]	295	295	100%	1e-101	90%	WP_017319714.1	*Chlorogloeopsidaceae*
Fur family transcriptional regulator [*Chlorogloeopsis fritschii*]	294	294	100%	2e-101	90%	WP_016877311.1	*Chlorogloeopsidaceae*
Ferric uptake regulation protein FUR [*Richelia intracellularis*]	266	266	100%	4e-90	89%	CDN15517.1	*Nostocaceae*
Fur family transcriptional regulator [*Scytonema hofmanni*]	290	290	100%	2e-99	89%	WP_017749066.1	*Scytonemataceae*
Fur family transcriptional regulator [*Raphidiopsis brookii*]	285	285	100%	1e-97	89%	WP_009343575.1	*Nostocaceae*
ferric uptake regulator, Fur family [*Gloeocapsa* sp. PCC 7428]	286	286	100%	2e-98	89%	WP_015188759.1	*Microcystaceae*
Fe^{2+}/Zn^{2+} uptake regulation protein [*Rivularia* sp. PCC 7116]	286	286	100%	4e-98	88%	WP_015117423.1	*Rivulariaceae*
Fur family transcriptional regulator [*Cylindrospermopsis raciborskii*]	283	283	100%	6e-97	88%	WP_006277045.1	*Nostocaceae*
Fur family transcriptional regulator [*Mastigocoleus testarum*]	283	283	100%	4e-97	87%	WP_027843573.1	*Nostochopsidaceae*
ferric uptake regulator family protein [*Chroococcidiopsis thermalis*]	276	276	100%	2e-94	85%	WP_015152359.1	*Xenococcaceae*
Fe^{2+}/Zn^{2+} uptake regulation protein [*Synechocystis* sp. PCC 7509]	273	273	100%	8e-93	84%	WP_009631818.1	*Merismopediaceae*
Fe^{2+}/Zn^{2+} uptake regulation protein [*Oscillatoria acuminata*]	269	269	100%	2e-91	82%	WP_015150141.1	*Oscillatoriaceae*
Fur family transcriptional regulator [*Geitlerinema* sp. PCC 7105]	264	264	99%	1e-89	81%	WP_026097494.1	*Pseudanabaenaceae*
Fur family transcriptional regulator [*Leptolyngbya boryana*]	258	258	98%	3e-87	81%	WP_017285907.1	*Pseudanabaenaceae*
ferric uptake regulator family protein [*Crinalium epipsammum*]	264	264	100%	2e-89	80%	WP_015203706.1	*Gomontiellaceae*

[a]Protein sequences alignments were performed using the Basic Local Alignment Search Tool (BLAST) from NCBI.

Table B. Complete list of genes showing ≥2-fold change in expression in the *furA*-turning off strain AGcoaRFurA

ORF	RPKM (WT)	RPKM ($FurA^-$)	Fold change	Protein description	Functional category
all8559	34,862	0,872	-39,965	transposase	transposon-related functions
asr2389	34,454	0,000	-34,454	unknown	unknown
all4671	19,260	0,949	-20,300	metal dependent phosphohydrolase	other enzymes
all4063	18,223	0,000	-18,223	phosphatidylglycerophosphate synthase	lipid metabolism
all7234	3,831	0,224	-17,128	TPR repeat-containing protein	unknown
asr7638	16,167	0,000	-16,167	heavy metal transport/detoxification protein	transport across membrane
alr2725	6,081	0,383	-15,859	deoxyribopyrimidine photolyase	DNA replication, recombination and repair
asl7327	14,827	0,000	-14,827	unknown	unknown
asr3168	14,780	0,000	-14,780	ribosomal protein PSRP-3/Ycf65	ribosome component
asr7256	14,153	0,000	-14,153	unknown	unknown
alr4311	20,387	1,461	-13,956	ABC transporter, ATP-binding protein	transport across membrane
all4414	3,701	0,265	-13,956	dynamin-like protein (GTPase)	membrane invaginations, membrane fusions
alr4716	5,228	0,392	-13,322	two-component sensor histidine kinase	regulatory functions
all1884	10,754	0,807	-13,322	short-chain dehydrogenase/reductase SDR	redox reactions
all7619	4,090	0,322	-12,687	unknown	unknown
all1802	5,248	0,414	-12,687	Na+-transporting ATP synthase	transport across membrane
asl8028	12,384	0,000	-12,384	unknown	unknown
asl0597	12,219	0,000	-12,219	unknown	unknown
asr2388	37,601	3,120	-12,053	unknown	unknown
alr0556	10,902	0,904	-12,053	DegT/DnrJ/EryC1/StrS aminotransferase	cell envelope
all0378	5,215	0,457	-11,419	sodium-dependent nucleoside transporter	salvage pathways
asr3468	11,120	0,000	-11,120	unknown	unknown
all0539	10,963	0,000	-10,963	unknown	unknown
alr3177	6,321	0,586	-10,784	probable glycosyl transferase	sugars
all4797	11,608	1,076	-10,784	unknown	unknown
alr1347	11,089	1,028	-10,784	unknown	unknown
alr7243	3,078	0,303	-10,150	ankyrin	attachment of integral membrane proteins to the cytoskeleton
alr3138	10,321	1,017	-10,150	peptidase M22, glycoprotease	degradation of proteins, peptides, and glycopeptides
alr0201	16,453	1,673	-9,833	translation factor SUA5	translation
all3976	4,102	0,431	-9,516	seryl-tRNA synthetase; SerS	aminoacyl tRNA synthetases and tRNA modification
all3579	11,227	1,180	-9,516	unknown	unknown
alr1379	6,815	0,716	-9,516	unknown	unknown

all2853	5,197	0,546	-9,516	UDP-glucose 4-epimerase	sugars
alr1298	20,155	2,191	-9,198	pentapeptide repeat-containing protein	unknown
asr7603	9,122	0,000	-9,122	unknown	unknown
all2934	4,880	0,549	-8,881	glutaminase	amino acids biosynthesis and metabolism
alr7241	2,903	0,327	-8,881	reverse transcriptase homolog	transposon-related functions
all3093	3,224	0,363	-8,881	bifunctional purine biosynthesis protein, PurH	purine/pyrimidine biosynthesis and metabolism
all4689	9,616	1,083	-8,881	unknown	unknown
alr3496	14,214	1,600	-8,881	unknown	unknown
asl2779	33,360	3,756	-8,881	unknown	unknown
asr3796	8,812	0,000	-8,812	stress induced hydrophobic peptide	resistance, adaptations to atypical conditions
asr3080	8,585	0,000	-8,585	unknown	unknown
all3876	15,684	1,831	-8,564	precorrin decarbocylase, CbiT subunit	cobalamin, heme, phycobilin and porphyrin
alr8562	12,712	1,484	-8,564	chromosome partitioning protein, ParA family ATPase	nucleoproteins
alr0084	74,134	8,765	-8,458	fdxN element excision controlling factor XisI homolog	heterocyst differentiation
all4699	5,345	0,648	-8,247	prolipoprotein diacylglyceryl transferase	lipoprotein biogenesis
alr5324	2,357	0,286	-8,247	penicillin-binding protein	surface components
all4950	5,750	0,697	-8,247	unknown	unknown
all1380	7,865	0,954	-8,247	unknown	unknown
alr7206	1,615	0,196	-8,247	unknown	unknown
asl1262	7,997	0,000	-7,997	unknown	unknown
alr0296	28,511	3,567	-7,993	unknown	unknown
all4813	391,322	49,235	-7,948	serine/threonine kinase	regulatory functions
all0498	7,371	0,930	-7,930	DegT/DnrJ/EryC1/StrS family	cell envelope
alr4069	7,847	0,990	-7,930	permease YjgP/YjgQ	transport across membrane
alr3304	467,044	59,090	-7,904	unknown	unknown
asr2155	7,889	0,000	-7,889	unknown	unknown
all4960	6,438	0,823	-7,824	ABC-1 domain protein	protein amino acid phosphorylation
asl2942	7,784	0,000	-7,784	unknown	unknown
alr1104	7,733	0,000	-7,733	unknown	unknown
all3963	7,711	0,000	-7,711	unknown	unknown
all1618	66,984	8,719	-7,683	unknown	unknown
all3634	3,969	0,521	-7,612	similar to esterase	lipid metabolism
alr1853	7,374	0,969	-7,612	transposase	transposon-related functions
all4962	3,328	0,437	-7,612	unknown	unknown
asl2451	18,197	2,390	-7,612	unknown	unknown
all3392	4,038	0,530	-7,612	cobalamin synthesis protein CobW	cobalamin, heme, phycobilin and porphyrin
asr4076	19,460	2,556	-7,612	unknown	unknown
alr9026	5,410	0,711	-7,612	chromosome partitioning protein, ParA family ATPase	nucleoproteins
all3836	41,083	5,454	-7,533	glucose 1-dehydrogenase (NAD+)	sugars

all2012	16,333	2,191	-7,454	ABC transporter, permease protein	transport across membrane
all2645	5,553	0,748	-7,422	peptide synthetase	siderophore/cyanotoxin biosyntesis
all1686	13,443	1,816	-7,401	putative purple acid phosphatase precursor	other enzymes
alr3430	7,339	0,000	-7,339	unknown	unknown
all0801	7,298	0,000	-7,298	photosystem II protein W	photosynthesis
alr0117	17,552	2,406	-7,295	two-component sensor histidine kinase	regulatory functions
alr4281	13,563	1,859	-7,295	heterocyst specific ABC-transporter, membrane spanning subunit DevC homolog	transport across membrane, heterocyst
all0495	18,024	2,471	-7,295	polysaccharide export protein	exopolysaccharide biosynthesis
alr4610	24,060	3,346	-7,190	pentapeptide repeat containing protein	unknown
all1848	14,332	1,993	-7,190	similar to methyltransferase	other enzymes
asl2437	7,185	0,000	-7,185	unknown	unknown
asl8515	7,120	0,000	-7,120	putative carbohydrate kinase	sugars
asr0148	7,120	0,000	-7,120	unknown	unknown
alr1105	7,076	0,000	-7,076	arsenate reductase	detoxification
alr3038	4,052	0,581	-6,978	putative methyltransferase	other enzymes
alr5368	4,103	0,588	-6,978	sugar ABC transporter, permease protein	transport across membrane
all7171	4,587	0,657	-6,978	unknown	unknown
alr1392	4,414	0,632	-6,978	unknown	unknown
alr3638	3,293	0,472	-6,978	DNA mismatch repair protein	DNA replication, recombination and repair
alr3147	7,832	1,122	-6,978	phosphoenolpyruvate synthase	pyruvate metabolism and reductive carboxylate cycle (CO2 fixation)
all2367	11,783	1,689	-6,978	thioredoxin	redox homeostasis
all3673	6,520	0,934	-6,978	oxidoreductase, molybdopterin binding	redox reactions
all5343	3,051	0,437	-6,978	glycosyl transferase	sugars
alr0812	5,512	0,790	-6,978	unknown	unknown
alr7656	8,027	1,150	-6,978	unknown	unknown
asl8021	15,663	2,245	-6,978	unknown	unknown
asr5054	35,677	5,113	-6,978	unknown	unknown
all4431	4,039	0,579	-6,978	glycosyl transferase	sugars
all4622	1,281	0,184	-6,978	unknown	unknown
alr7127	1,791	0,257	-6,978	unknown	unknown
alr9012	2,181	0,312	-6,978	unknown	unknown
asl0046	26,212	3,756	-6,978	YiaAB two helix domain-containing protein	unknown
asl3322	6,868	0,000	-6,868	unknown	unknown
all4822	84,892	12,559	-6,759	similar to beta-lactamase	resistance, adaptations to atypical conditions
asr4498	6,710	0,000	-6,710	unknown	unknown
all1101	2,767	0,415	-6,661	ferrichrome iron receptor	transport across membrane
all4430	5,560	0,835	-6,661	probable polysaccharide biosynthesis protein	sugars
alr3920	14,630	2,218	-6,597	two-component response regulator	regulatory functions
alr2411	707,972	107,481	-6,587	serine/threonine kinase	regulatory functions
asr1662	6,560	0,000	-6,560	unknown	unknown

alr2526	10,837	1,653	-6,555	luciferase-alpha subunit	bioluminescence
all3983	9,026	1,377	-6,555	similar to surface layer protein	cell envelope
alr3932	6,543	0,000	-6,543	unknown	unknown
asl3856	6,487	0,000	-6,487	similar to WD repeat protein	regulatory functions
all3169	4,170	0,657	-6,344	WD repeat protein with Ser/Thr protein kinase motif	regulatory functions
all1862	3,496	0,551	-6,344	similar to peptidase	degradation of proteins, peptides, and glycopeptides
alr3425	3,002	0,473	-6,344	probable glycosyl transferase	sugars
all7610	3,828	0,603	-6,344	cation efflux system protein	transport across membrane
alr3287	12,226	1,927	-6,344	nitrate transport protein NrtB	transport across membrane
alr3648	3,002	0,473	-6,344	heterocyst specific ABC-transporter, membrane spanning subunit DevC homolog	transport across membrane, heterocyst
all0605	2,926	0,461	-6,344	PBS lyase HEAT-like repeat	energy production and conversion
all0921	9,980	1,573	-6,344	Rieske (2Fe-2S) domain-containing protein	redox reactions
all3014	9,650	1,521	-6,344	glyoxalase/bleomycin resistance protein/dioxygenase	resistance, adaptations to atypical conditions
alr0585	3,593	0,566	-6,344	probable glycosyl transferase	sugars
all5177	15,673	2,471	-6,344	molybdenum-pterin binding protein homolog	transport across membrane
alr1635	2,056	0,324	-6,344	sulfate permease	transport across membrane
all2320	5,897	0,930	-6,344	unknown	unknown
all3696	3,073	0,484	-6,344	unknown	unknown
all4276	4,615	0,727	-6,344	unknown	unknown
all4790	4,200	0,662	-6,344	unknown	unknown
alr2820	5,482	0,864	-6,344	unknown	unknown
alr7189	2,715	0,428	-6,344	unknown	unknown
alr7358	9,812	1,547	-6,344	unknown	unknown
asr7038	11,794	1,859	-6,344	unknown	unknown
alr4576	5,927	0,934	-6,344	biotin biosynthesis protein BioY	vitamin biosynthesis
alr3386	9,122	1,438	-6,344	two-component response regulator	regulatory functions
alr7643	6,311	0,000	-6,311	unknown	unknown
all0858	6,255	0,000	-6,255	unknown	unknown
all0876	54,674	8,765	-6,238	unknown	unknown
alr1565	6,235	0,000	-6,235	unknown	unknown
asl2914	6,227	0,000	-6,227	similar to ferredoxin	electron transport chain
asr4584	6,211	0,000	-6,211	unknown	unknown
asr0516	6,145	0,000	-6,145	unknown	unknown
all2103	62,508	10,225	-6,113	unknown	unknown
asr1257	6,039	0,000	-6,039	unknown	unknown
alr0698	6,028	1,000	-6,027	hydrogenase expression/formation protein HupE	other enzymes
all3840	75,629	12,549	-6,027	NADH dehydrogenase chain J	respiration, oxidative phosphorylation
asl2332	5,988	0,000	-5,988	unknown	unknown

all0274	6,636	1,113	-5,963	Na-Ca exchanger/integrin-beta4	transport across membrane
alr1286	5,923	0,000	-5,923	ExsB family protein	unknown
all1319	20,433	3,451	-5,921	2-dehydropantoate 2-reductase	coenzymes and cofactors
all5323	5,351	0,904	-5,921	two-component transcription regulator, complementary chromatic adaptation protein RcaC homolog	regulatory functions
asr3266	5,912	0,000	-5,912	unknown	unknown
asl0884	5,897	0,000	-5,897	probable ferredoxin [2Fe-2S]	electron transport chain
rrn5Sa	5,887	0,000	-5,887	5S rRNA	ribosome component
alr4990	5,871	0,000	-5,871	O-6-alkylguanine-DNA/cysteine-protein methyltransferase	DNA degradation and modification
alr2117	7,153	1,219	-5,868	unknown	unknown
all0271	81,417	13,929	-5,845	unknown	unknown
alr0590	5,780	0,000	-5,780	transposase	transposon-related functions
asl3595	5,742	0,000	-5,742	unknown	unknown
all4087	8,543	1,496	-5,709	protein kinase C inhibitor	cell division
alr2169	6,527	1,143	-5,709	similar to D,D-carboxypeptidase	cell envelope
alr1171	2,340	0,410	-5,709	two-component sensor histidine kinase	regulatory functions
all3332	5,077	0,889	-5,709	pentapeptide repeat-containing protein	unknown
alr3912	3,562	0,624	-5,709	aspartoacylase	amino acids biosynthesis and metabolism
all3031	1,751	0,307	-5,709	OmpA/MotB	cell envelope
all1466	2,335	0,409	-5,709	kinesin light chain	cell shape, microtubule motor activity
all0968	3,574	0,626	-5,709	3-ketoacyl-acyl carrier protein reductase; FabG	lipid metabolism
alr3714	7,755	1,358	-5,709	HAD-superfamily hydrolase subfamily IIB	other enzymes
all2094	1,837	0,322	-5,709	two-component hybrid sensor and regulator	regulatory functions
all4924	1,223	0,214	-5,709	ferrichrome iron receptor	transport across membrane
all7106	31,436	5,506	-5,709	transposase	transposon-related functions
all3013	12,712	2,226	-5,709	unknown	unknown
all4265	4,691	0,822	-5,709	unknown	unknown
all5060	5,502	0,964	-5,709	unknown	unknown
alr2131	4,316	0,756	-5,709	unknown	unknown
alr7126	1,612	0,282	-5,709	unknown	unknown
asl2850	39,906	6,990	-5,709	unknown	unknown
alr0728	4,650	0,814	-5,709	HAD family hydrolase	other enzymes
alr3269	4,472	0,783	-5,709	FHA domain containing protein	regulatory functions
all3793	4,472	0,783	-5,709	allergen V5/Tpx-1 family protein	unknown
all7379	4,957	0,868	-5,709	unknown	unknown
alr3491	3,028	0,530	-5,709	unknown	unknown
asl1795	5,627	0,000	-5,627	unknown	unknown
all0345	5,617	0,000	-5,617	transcriptional regulator	regulatory functions
all0966	8,969	1,600	-5,604	unknown	unknown
all1291	5,560	0,000	-5,560	cyanate lyase; CynS	detoxification

asl7266	109,228	19,791	-5,519	unknown	unknown
all2084	9,399	1,710	-5,498	unknown	unknown
alr3610	5,473	0,000	-5,473	transposase	transposon-related functions
alr1370	4,362	0,809	-5,392	unknown	unknown
all0743	3,629	0,673	-5,392	adenylate cyclase; CyaD	regulatory functions
alr3511	6,403	1,187	-5,392	two-component sensor histidine kinase	regulatory functions
all1841	5,389	0,000	-5,389	unknown	unknown
alr4322	5,326	0,000	-5,326	malate dehydrogenase	TCA cycle
asr0847	5,307	0,000	-5,307	photosystem II protein PsbN	photosynthesis
asl2786	5,307	0,000	-5,307	unknown	unknown
all2545	10,811	2,045	-5,286	unknown	unknown
alr1538	9,539	1,804	-5,286	unknown	unknown
all1322	4,026	0,762	-5,286	TPR repeat-containing protein	unknown
all7372	5,239	0,000	-5,239	unknown	unknown
all2314	13,664	2,611	-5,234	unknown	unknown
asr4522	5,228	0,000	-5,228	putative N-acetylmuramoyl-L-alanine amidase	peptidoglycan biosynthesis
all0659	5,206	0,000	-5,206	probable transcription regulator	regulatory functions
all5093	111,093	21,444	-5,181	unknown	unknown
asr4048	5,151	0,000	-5,151	unknown	unknown
alr4170	197,165	38,575	-5,111	unknown	unknown
all4294	47,943	9,402	-5,099	probable N-acetylmuramoyl-L-alanine amidase	peptidoglycan biosynthesis
alr3362	5,077	0,000	-5,077	unknown	unknown
asl3784	5,077	0,000	-5,077	unknown	unknown
all2787	1,010	0,199	-5,075	probable kinesin light chain	cell shape microtubule motor activity
alr0379	3,692	0,727	-5,075	cobalamin synthase	cobalamin, heme, phycobilin and porphyrin
all3286	3,210	0,632	-5,075	N-acetylmuramoyl-L-alanine amidase	peptidoglycan biosynthesis
all0450	5,766	1,136	-5,075	allophycocyanin alpha subunit ApcA	phycobilisomes and phycobiliproteins
alr0904	2,080	0,410	-5,075	small GTP-binding protein domain	regulatory functions
all4896	1,807	0,356	-5,075	putative diguanylate cyclase with PAS/PAC sensor	regulatory functions
alr2190	11,368	2,240	-5,075	alpha-amylase	sugars
alr5237	2,235	0,440	-5,075	probable glycosyl transferase	sugars
all1949	2,504	0,493	-5,075	ABC transporter, permease protein	transport across membrane
all1110	2,318	0,457	-5,075	chromate transport protein	transport across membrane
all0321	3,221	0,635	-5,075	sulfate transport system permease protein	transport across membrane
all0282	5,731	1,129	-5,075	unknown	unknown
all1465	1,411	0,278	-5,075	unknown	unknown
all1958	2,566	0,506	-5,075	unknown	unknown
all3404	7,984	1,573	-5,075	unknown	unknown
all3515	3,063	0,603	-5,075	unknown	unknown
all4425	2,755	0,543	-5,075	unknown	unknown
all4626	9,248	1,822	-5,075	unknown	unknown

all5037	3,593	0,708	-5,075	unknown	unknown
all8022	8,982	1,770	-5,075	unknown	unknown
alr0196	2,104	0,415	-5,075	unknown	unknown
alr1036	2,947	0,581	-5,075	unknown	unknown
alr3950	20,605	4,060	-5,075	unknown	unknown
asr3498	13,737	2,707	-5,075	unknown	unknown
alr2782	18,934	3,731	-5,075	3-dehydroquinate dehydratase	amino acids biosynthesis and metabolism
all0086	6,818	1,343	-5,075	rod shape-determining protein MreC	cell shape
all2847	2,518	0,496	-5,075	cobalamin biosynthesis protein D	cobalamin, heme, phycobilin and porphyrin
alr1264	1,753	0,345	-5,075	DnaK-type molecular chaperone DnaK	heat shock proteins and chaperones
alr3789	1,218	0,240	-5,075	predicted signal transduction protein containing Nacht domain	regulatory functions
alr0582	2,341	0,461	-5,075	glycosyl transferase	sugars
all5163	9,467	1,865	-5,075	peptidoglycan-binding LysM	surface components
all2236	1,087	0,214	-5,075	ferrichrome-iron receptor	transport across membrane
all2067	4,916	0,969	-5,075	transposase	transposon-related functions
alr5340	2,284	0,450	-5,075	TPR repeat-containing protein	unknown
all1317	6,311	1,244	-5,075	unknown	unknown
all3208	2,365	0,466	-5,075	unknown	unknown
alr0162	6,997	1,379	-5,075	unknown	unknown
alr4742	1,766	0,348	-5,075	unknown	unknown
asr0243	9,532	1,878	-5,075	unknown	unknown
alr0348	61,522	12,293	-5,004	NADH dehydrogenase subunit 4; NdhD	respiration, oxidative phosphorylation
alr3397	9,479	1,897	-4,996	phosphoenolpyruvate synthase	pyruvate metabolism and reductive carboxylate cycle (CO2 fixation)
asr3249	4,969	0,000	-4,969	putative ammonia monooxygenase	nitrogen metabolism
all4935	6,879	1,390	-4,948	unknown	unknown
alr9017	25,071	5,081	-4,934	unknown	unknown
alr3506	11,069	2,251	-4,916	phosphoribose diphosphate:decaprenyl-phosphate phosphoribosyltransferase	coenzymes and cofactors
all8023	3,580	0,728	-4,916	pentapeptide repeat-containing protein	unknown
alr4686	59,252	12,166	-4,870	cytochrome P450, germacrene A hydroxylase	sesquiterpene biosynthesis
all3744	4,745	0,976	-4,864	probable phytoene dehydrogenase	carotenoids
alr0189	4,901	1,008	-4,864	ABC-1 domain protein	protein amino acid phosphorylation
all2152	28,773	5,916	-4,864	transposase	transposon-related functions
all7313	7,795	1,603	-4,864	unknown	unknown
all4125	5,204	1,070	-4,864	unknown	unknown
all3893	4,831	0,000	-4,831	unknown	unknown
all4260	30,390	6,303	-4,821	unknown	unknown
alr3982	13,601	2,832	-4,803	two-component sensor histidine kinase	regulatory functions
all5044	16,200	3,373	-4,803	polyamine ABC transporter, ATP-binding protein	transport across membrane
asr2387	75,467	15,712	-4,803	unknown	unknown

all4473	4,790	0,000	-4,790	unknown	unknown
alr4405	58,680	12,270	-4,782	unknown	unknown
all0283	7,548	1,579	-4,779	WD-40 repeat protein	regulatory functions
all1090	10,969	2,306	-4,758	peptidase U62, modulator of DNA gyrase	degradation of proteins, peptides, and glycopeptides
all2761	5,724	1,203	-4,758	alpha/beta hydrolase fold protein	other enzymes
alr2130	14,239	2,993	-4,758	unknown	unknown
alr9015	5,166	1,086	-4,758	unknown	unknown
all3922	5,490	1,154	-4,758	L-asparaginase	amino acids biosynthesis and metabolism
alr0522	11,191	2,352	-4,758	probable pseudouridine synthase	posttranscriptional modifications
all0778	4,670	0,982	-4,758	similar to serine protease inhibitor	protease inhibitor
all5009	6,560	1,379	-4,758	aspartyl/asparaginyl beta-hydroxylase	unknown
all1672	11,676	2,454	-4,758	unknown	unknown
all1865	4,346	0,913	-4,758	similar to xenobiotic reductase	energy production and conversion
all2070	5,799	1,219	-4,758	ribonuclease BN	RNA degradation
alr2324	63,661	13,411	-4,747	unknown	unknown
all2568	4,746	0,000	-4,746	2-phosphosulfolactate phosphatase	coenzymes and cofactors
all1082	4,746	0,000	-4,746	photosystem II protein PsbW, class I	photosynthesis
all0688	4,729	0,000	-4,729	[NiFe] uptake hydrogenase small subunit (hupS)	photosynthesis
alr1385	4,727	0,000	-4,727	unknown	unknown
alr0971	30,468	6,447	-4,726	unknown	unknown
all0703	4,708	0,000	-4,708	unknown	unknown
alr7333	24,546	5,229	-4,694	putative nuclease	DNA degradation and modification
alr2386	1461,582	311,984	-4,685	TPR repeat-containing protein	unknown
all4523	68,207	14,579	-4,679	unknown	unknown
all1365	4,670	0,000	-4,670	cytochrome CytM	photosynthesis
alr2856	3,485	0,749	-4,652	lipopolysaccharide biosynthesis protein	LPS biosynthesis
all3367	65,231	14,086	-4,631	TPR repeat-containing protein	unknown
all5100	3,123	0,676	-4,622	alpha-2-macroglobulin domain-containing protein	unknown
all3894	70,891	15,338	-4,622	unknown	unknown
alr3060	7,204	1,566	-4,599	O-antigen polymerase	cell wall/membrane biogenesis
alr3422	63,094	13,719	-4,599	cytochrome b6/f-complex, apocytochrome subunit 4 PetD	photosynthesis
alr5162	22,062	4,815	-4,582	unknown	unknown
asl0146	45,198	9,896	-4,567	50S ribosomal protein L27	ribosome component
all1830	4,534	0,000	-4,534	unknown	unknown
alr2394	21,784	4,807	-4,531	FraE	filament integrity
alr0702	61,910	13,697	-4,520	serine proteinase	degradation of proteins, peptides, and glycopeptides
asl9502	327,314	72,416	-4,520	unknown	unknown
alr4329	58,897	13,031	-4,520	anti-sigma-factor antagonist	regulatory functions
alr2769	4,513	0,000	-4,513	transcriptional regulator	regulatory functions

asl3518	4,491	0,000	-4,491	putative ABC transporter, transmembrane region	transport across membrane
alr4953	34,102	7,596	-4,489	unknown	unknown
all1673	1,746	0,393	-4,441	similar to heme binding protein CemA	cell envelope
alr5314	1,100	0,248	-4,441	photosystem I P700 chlorophyll a apoprotein A2	photosynthesis
alr2204	4,969	1,119	-4,441	thioredoxin reductase	redox homeostasis
alr4079	4,894	1,102	-4,441	4-hydroxybenzoyl-CoA thioesterase	resistance, adaptations to atypical conditions
alr3062	5,077	1,143	-4,441	probable glycosyl transferase	sugars
all0042	23,278	5,242	-4,441	putative glycosyl transferase	sugars
alr4509	7,006	1,578	-4,441	polysaccharide pyruvyl transferase	surface components
alr2879	1,224	0,276	-4,441	bicarbonate transport ATP-binding protein CmpC	transport across membrane
all2399	9,692	2,182	-4,441	unknown	unknown
alr3008	6,755	1,521	-4,441	unknown	unknown
all4970	2,339	0,527	-4,441	hydantoinase/oxoprolinase	amino acids biosynthesis and metabolism
alr1039	4,097	0,923	-4,441	probable aspartate aminotransferase	amino acids biosynthesis and metabolism
alr1885	2,404	0,541	-4,441	queuine tRNA-ribosyltransferase	aminoacyl tRNA synthetases and tRNA modification
asl4136	10,754	2,422	-4,441	prevent-host-death protein	cell division
all3702	3,027	0,682	-4,441	similar to D,D-carboxypeptidase	cell envelope
alr3345	4,794	1,080	-4,441	curli production assembly/transport component CsgG	cell wall/membrane biogenesis
all4135	6,336	1,427	-4,441	PilT protein-like	chemotaxis and cell motility
all0067	4,566	1,028	-4,441	cobalamin adenosyltransferase	cobalamin, heme, phycobilin and porphyrin
alr0996	1,671	0,376	-4,441	protease	degradation of proteins, peptides, and glycopeptides
all4941	33,225	7,482	-4,441	putative Orange carotenoid protein	detoxification
alr4604	0,848	0,191	-4,441	type I site-specific deoxyribonuclease chain R	DNA degradation and modification
all1427	4,128	0,930	-4,441	CBS domain containing membrane protein	membrane component
all3141	5,461	1,230	-4,441	AAA superfamily ATPase	membrane fusions, proteolysis and DNA replication
all3113	3,802	0,856	-4,441	serine esterase	other enzymes
all0193	13,948	3,141	-4,441	haloalkane dehalogenase	other enzymes
all2470	3,784	0,852	-4,441	protein serine-threonine phosphatase	regulatory functions
alr4954	1,788	0,403	-4,441	serine/threonine kinase	regulatory functions
alr0642	7,773	1,751	-4,441	two-component sensor histidine kinase	regulatory functions
all1495	12,574	2,832	-4,441	virulence associated protein C	resistance, adaptations to atypical conditions
all2641	0,801	0,180	-4,441	polyketide synthase	siderophore/cyanotoxin biosynthesis
all4426	1,955	0,440	-4,441	probable glucosyltransferase	sugars
all5118	14,860	3,346	-4,441	probable glycosyl transferase	sugars
alr4492	2,383	0,537	-4,441	probable glycosyltransferase	sugars
all0916	7,656	1,724	-4,441	ABC transporter ATP binding subunit	transport across membrane
alr2539	3,144	0,708	-4,441	branched-chain amino acid ABC transporter, ATP-binding protein	transport across membrane

alr2209	0,926	0,208	-4,441	ferric aerobactin receptor	transport across membrane
alr1854	4,780	1,076	-4,441	transposase	transposon-related functions
alr3384	2,743	0,618	-4,441	transposase	transposon-related functions
alr1315	3,027	0,682	-4,441	type IV prepilin peptidase	type-IV pilus biogenesis and extracellular protein secretion
all0403	7,430	1,673	-4,441	unknown	unknown
all3852	2,898	0,653	-4,441	unknown	unknown
alr1851	3,434	0,773	-4,441	unknown	unknown
alr5197	2,008	0,452	-4,441	unknown	unknown
alr7074	6,336	1,427	-4,441	unknown	unknown
alr7503	6,436	1,449	-4,441	unknown	unknown
alr7517	4,066	0,916	-4,441	unknown	unknown
alr7613	3,050	0,687	-4,441	unknown	unknown
alr8529	4,235	0,954	-4,441	unknown	unknown
asr1558	11,845	2,667	-4,441	unknown	unknown
asr4560	11,196	2,521	-4,441	unknown	unknown
all0771	2,369	0,533	-4,441	4-hydroxyphenylpyruvate dioxygenase	amino acids biosynthesis and metabolism
asl2686	4,434	0,000	-4,434	unknown	unknown
asl4266	4,434	0,000	-4,434	unknown	unknown
alr2478	4,423	0,000	-4,423	unknown	unknown
all1991	4,414	0,000	-4,414	unknown	unknown
all1691	138,270	31,483	-4,392	ferric uptake regulator FurA	regulatory functions
all1065	24,511	5,620	-4,361	unknown	unknown
all4875	36,554	8,392	-4,356	unknown	unknown
alr0543	57,058	13,104	-4,354	ABC transporter; substrate-binding protein	transport across membrane
all1701	14,371	3,304	-4,350	unknown	unknown
alr7092	4,324	0,000	-4,324	unknown	unknown
all3841	223,080	51,626	-4,321	NADH dehydrogenase chain K	respiration, oxidative phosphorylation
alr4863	16,697	3,865	-4,320	unknown	unknown
all1027	4,314	0,000	-4,314	ABC transporter, periplasmic sugar-binding protein	transport across membrane
alr2581	4,506	1,045	-4,314	similar to ferric aerobactin receptor	transport across membrane
alr0236	4,300	0,000	-4,300	unknown	unknown
all4887	6,447	1,506	-4,282	lysine decarboxylase	amino acids biosynthesis and metabolism
all3722	54,623	12,756	-4,282	precorrin-6y-dependent methyltransferase	cobalamin, heme, phycobilin and porphyrin
all4760	9,821	2,294	-4,282	unknown	unknown
alr5226	35,635	8,366	-4,259	unknown	unknown
alr4153	4,246	0,000	-4,246	sporulation-control protein Spo0M homolog	resistance, adaptations to atypical conditions
alr3150	51,308	12,094	-4,242	glycosyl transferase	sugars
all0405	3,370	0,797	-4,229	unknown	unknown
alr4280	14,161	3,369	-4,203	heterocyst specific ABC-transporter, membrane fusion protein DevB homolog	transport across membrane, heterocyst

all7112	26,446	6,293	-4,203	transposase	transposon-related functions
alr4685	43,017	10,257	-4,194	terpene synthase, metal-binding	terpene biosynthesis
all5221	14,324	3,421	-4,187	unknown	unknown
alr7009	4,170	0,000	-4,170	transposase	transposon-related functions
alr2943	4,170	0,000	-4,170	unknown	unknown
alr3968	209,108	50,197	-4,166	unknown	unknown
alr1659	4,162	0,000	-4,162	surface antigen	surface components
asr0925	71,759	17,255	-4,159	unknown	unknown
all2531	4,145	0,000	-4,145	nitrogen fixation protein NifX homolog	nitrogen fixation
all3735	31,697	7,648	-4,145	fructose-bisphosphate aldolase class I	glycolisis
all0830	43,934	10,607	-4,142	ABC transporter permease protein	transport across membrane
asr3427	22,998	5,577	-4,123	50S ribosomal protein L35	ribosome component
alr0227	4,944	1,199	-4,123	inorganic polyphosphate/ATP-NAD kinase	sugars
all0363	25,298	6,135	-4,123	transposase	transposon-related functions
alr0739	23,473	5,692	-4,123	unknown	unknown
alr0805	41,873	10,155	-4,123	unknown	unknown
alr0644	6,963	1,689	-4,123	biopolymer transport protein ExbD/TolR	intracellular trafficking and secretion
alr1170	38,755	9,399	-4,123	two-component response regulator	regulatory functions
all7161	3,729	0,904	-4,123	transposase	transposon-related functions
all4333	7,744	1,878	-4,123	unknown	unknown
asl3597	87,235	21,156	-4,123	unknown	unknown
asl3966	4,121	0,000	-4,121	unknown	unknown
alr2486	60,126	14,676	-4,097	ABC transporter ATP-binding protein	transport across membrane
asl2354	4,097	0,000	-4,097	CAB/ELIP/HLIP-related protein	resistance, adaptations to atypical conditions
all4236	4,097	0,000	-4,097	unknown	unknown
alr2246	4,097	0,000	-4,097	unknown	unknown
all7172	9,951	2,440	-4,078	putative helicase	DNA replication, recombination and repair
all3021	4,050	0,000	-4,050	unknown	unknown
alr7261	4,040	0,000	-4,040	sugar-non-specific nuclease NucA homolog	DNA degradation and modification
all7110	44,246	10,979	-4,030	unknown	unknown
alr4077	109,654	27,208	-4,030	unknown	unknown
alr3337	4,026	0,000	-4,026	unknown	unknown
alr3088	5,718	1,423	-4,018	class-V aminotransferase; NifS	nitrogen metabolism
alr3385	11,204	2,789	-4,018	GAF domain containing protein	secondary metabolites biosynthesis
asl4098	34,130	8,495	-4,018	unknown	unknown
all2390	14,452	3,597	-4,018	cell division inhibitor	cell division
all2358	6,784	1,689	-4,018	phosphonate ABC transport phosphonate binding component	transport across membrane
alr3647	5,100	1,269	-4,018	heterocyst specific ABC-transporter, membrane fusion protein DevB homolog	transport across membrane, heterocyst
all1764	10,223	2,545	-4,018	unknown	unknown
all4763	10,666	2,655	-4,018	unknown	unknown

asl3517	418,394	104,299	-4,011	unknown	unknown
all1889	30,650	7,669	-3,997	unknown	unknown
alr4628	59,608	14,924	-3,994	transposase	transposon-related functions
alr1856	90,131	22,604	-3,987	unknown	unknown
alr3671	12,592	3,158	-3,987	two-component hybrid sensor and regulator	regulatory functions
alr0083	191,217	48,015	-3,982	fdxN element excision controlling factor XisH homolog	heterocyst differentiation
all2692	3,971	0,000	-3,971	transposase	transposon-related functions
asr9503	42,305	10,670	-3,965	unknown	unknown
asl5085	134,208	33,850	-3,965	unknown	unknown
asr0843	3,958	0,000	-3,958	30S ribosomal protein S21	ribosome component
alr4823	3,958	0,000	-3,958	similar to glucosyl-1-phosphate transferase	sugars
asl1676	3,958	0,000	-3,958	unknown	unknown
asr0100	3,958	0,000	-3,958	unknown	unknown
alr0246	3,948	0,000	-3,948	heat shock protein DnaJ-like	heat shock proteins and chaperones
all4883	96,143	24,376	-3,944	NADH dehydrogenase subunit 2	respiration, oxidative phosphorylation
asr0756	3,936	0,000	-3,936	unknown	unknown
alr4891	34,011	8,654	-3,930	probable chloride channel protein	transport across membrane
alr4310	175,457	44,771	-3,919	unknown	unknown
all3899	3,918	0,000	-3,918	mutator protein MutT	DNA replication, recombination and repair
asl1749	142,423	36,407	-3,912	unknown	unknown
all4799	116,205	29,800	-3,900	DedA protein	membrane component
asr4959	3,892	0,000	-3,892	YciI-like protein	unknown
alr3608	61,864	15,976	-3,872	similar to endoglucanase	sugars
alr1300	3,866	0,000	-3,866	unknown	unknown
alr1794	58,783	15,232	-3,859	unknown	unknown
all0878	3,849	0,000	-3,849	probable acetyltransferase	other enzymes
asl3128	3,849	0,000	-3,849	unknown	unknown
asr1714	3,849	0,000	-3,849	unknown	unknown
asl0206	3,828	0,000	-3,828	unknown	unknown
asr3369	3,828	0,000	-3,828	unknown	unknown
asl4253	3,807	0,000	-3,807	unknown	unknown
all4477	0,983	0,258	-3,806	DnaK-type molecular chaperone DnaK	heat shock proteins and chaperones
alr1809	8,982	2,360	-3,806	heat shock protein, class I	heat shock proteins and chaperones
all4574	4,420	1,161	-3,806	phosphate ABC transporter, permease protein	transport across membrane
all3781	4,579	1,203	-3,806	unknown	unknown
alr3182	2,848	0,748	-3,806	glucose inhibited division protein B	cell division
alr3931	9,383	2,465	-3,806	rhomboid-like protein	degradation of proteins, peptides, and glycopeptides
asr4549	7,453	1,958	-3,806	pterin-4a-carbinolamine dehydratase	other enzymes
alr3095	6,507	1,710	-3,806	putative methyltransferase	other enzymes
all2760	2,091	0,549	-3,806	serine/threonine kinase	regulatory functions

all2699	1,521	0,400	-3,806	two-component sensor histidine kinase	regulatory functions
alr1540	38,015	9,988	-3,806	similar to penicillin-resistant DD-carboxypeptidase	resistance, adaptations to atypical conditions
alr3380	2,073	0,545	-3,806	dolichol-phosphate mannosyltransferase	surface components
all5110	0,880	0,231	-3,806	putative filamentous hemagglutinin family outer membrane protein	surface components
alr2880	2,416	0,635	-3,806	bicarbonate transport ATP-binding protein CmpD	transport across membrane
alr3946	2,548	0,669	-3,806	cobalt transport ATP-binding protein CbiO	transport across membrane
all0388	2,327	0,611	-3,806	iron(III) ABC transporter, periplasmic iron-compound-binding protein	transport across membrane
all4614	4,980	1,308	-3,806	multidrug efflux transporter, MFS family protein	transport across membrane
all3567	1,027	0,270	-3,806	probable Na+/H+-exchanging protein	transport across membrane
alr1398	0,829	0,218	-3,806	TPR repeat-containing protein	unknown
all0712	4,670	1,227	-3,806	unknown	unknown
all0753	5,307	1,394	-3,806	unknown	unknown
all0937	2,407	0,632	-3,806	unknown	unknown
all1509	2,104	0,553	-3,806	unknown	unknown
all4109	2,968	0,780	-3,806	unknown	unknown
alr0647	3,520	0,925	-3,806	unknown	unknown
alr0811	7,006	1,841	-3,806	unknown	unknown
alr2749	4,462	1,172	-3,806	unknown	unknown
alr3715	7,006	1,841	-3,806	unknown	unknown
alr4064	7,222	1,897	-3,806	unknown	unknown
alr4739	9,961	2,617	-3,806	unknown	unknown
alr4952	5,348	1,405	-3,806	unknown	unknown
alr5363	3,726	0,979	-3,806	unknown	unknown
asr0923	30,907	8,120	-3,806	unknown	unknown
asr4951	17,087	4,489	-3,806	unknown	unknown
all0170	1,914	0,503	-3,806	glycolate oxidase	amino acids biosynthesis and metabolism
all4966	3,639	0,956	-3,806	histidinol-phosphate aminotransferase	amino acids biosynthesis and metabolism
all0574	5,575	1,465	-3,806	phospho-2-dehydro-3-deoxyheptonate aldolase; AroF	amino acids biosynthesis and metabolism
all1638	4,220	1,109	-3,806	molybdopterin converting factor, subunit 2	coenzymes and cofactors
alr0312	1,191	0,313	-3,806	2-succinyl-6-hydroxy-2,4-cyclohexadiene-1-carboxylate synthase; MenD	coenzymes and cofactors
all0066	17,514	4,601	-3,806	dCTP deaminase	dNTPs biosynthesis
alr2405	8,194	2,153	-3,806	flavodoxin, IsiB	electron transport chain
all2102	4,755	1,249	-3,806	N-acyl-L-amino acid amidohydrolase	lipid metabolism
all2319	6,200	1,629	-3,806	nitrogen regulatory protein P-II	nitrogen metabolism
all5023	2,013	0,529	-3,806	probable hydrolase	other enzymes
all4106	3,925	1,031	-3,806	probable oxidoreductase	redox reactions
alr2920	4,798	1,261	-3,806	putative short chain oxidoreductase	redox reactions

all4518	1,345	0,353	-3,806	serine/threonine kinase	regulatory functions
all1625	0,392	0,103	-3,806	serine/threonine kinase with two-component sensor domain	regulatory functions
alr4394	4,702	1,235	-3,806	resolvase, RNase H-like fold	RNA synthesis and modification
all0832	2,715	0,713	-3,806	ABC transporter, ATP-binding protein	transport across membrane
alr1382	4,158	1,092	-3,806	ABC transporter, ferric iron-binding periplasmic protein	transport across membrane
alr2588	0,822	0,216	-3,806	ferrichrome-iron receptor	transport across membrane
all5206	1,297	0,341	-3,806	probable reverse transcriptase	transposon-related functions
asr7642	8,757	2,301	-3,806	putative transposase	transposon-related functions
alr0019	5,650	1,484	-3,806	transposase	transposon-related functions
all3083	6,625	1,740	-3,806	TPR repeat-containing protein	unknown
all0112	8,757	2,301	-3,806	unknown	unknown
all0967	1,412	0,371	-3,806	unknown	unknown
all1226	7,677	2,017	-3,806	unknown	unknown
all4297	3,199	0,840	-3,806	unknown	unknown
all4724	1,705	0,448	-3,806	unknown	unknown
all5191	1,376	0,362	-3,806	unknown	unknown
all9020	1,434	0,377	-3,806	unknown	unknown
alr2202	4,003	1,052	-3,806	unknown	unknown
alr3591	2,189	0,575	-3,806	unknown	unknown
alr3990	5,650	1,484	-3,806	unknown	unknown
alr2377	5,673	1,490	-3,806	cobyric acid synthase	cobalamin, heme, phycobilin and porphyrin
all0759	3,160	0,830	-3,806	WD-repeat containing protein	regulatory functions
alr3890	2,836	0,745	-3,806	unknown	unknown
all5274	270,716	71,248	-3,800	unknown	unknown
asl7034	3,766	0,000	-3,766	transcriptional regulator	regulatory functions
alr7384	3,766	0,000	-3,766	unknown	unknown
asr2348	3,766	0,000	-3,766	unknown	unknown
all4796	53,802	14,436	-3,727	unknown	unknown
asr1451	3,726	0,000	-3,726	unknown	unknown
alr7083	110,187	29,607	-3,722	chromosome partitioning protein, ParB family	nucleoproteins
alr9016	33,015	8,886	-3,716	unknown	unknown
alr3602	12,973	3,498	-3,709	probable long-chain-fatty-acid--CoA ligase	lipid metabolism
all2069	3,707	0,000	-3,707	unknown	unknown
alr2203	3,700	0,000	-3,700	unknown	unknown
alr0651	81,399	22,087	-3,685	unknown	unknown
all4085	3,682	0,000	-3,682	endonuclease V	DNA degradation and modification
all4828	138,494	37,618	-3,682	GDP-D-mannose dehydratase	sugars
alr3119	10,003	2,719	-3,679	WD repeat protein with Ser/Thr protein kinase motif	regulatory functions
all1072	8,572	2,330	-3,679	two-component response regulator	regulatory functions

all4855	3,678	0,000	-3,678	transcriptional regulator	regulatory functions
asr2994	252,768	68,769	-3,676	unknown	unknown
all4432	17,474	4,758	-3,673	probable exopolysaccharide biosynthesis protein	exopolysaccharide biosynthesis
alr0541	58,743	16,005	-3,670	GCN5-related N-acetyltransferase	other enzymes
alr2560	3,649	0,000	-3,649	unknown	unknown
alr5051	3,649	0,000	-3,649	unknown	unknown
asr3109	3,649	0,000	-3,649	unknown	unknown
asr4457	3,649	0,000	-3,649	unknown	unknown
all7362	19,531	5,354	-3,648	sugar-non-specific nuclease NucA	DNA degradation and modification
alr4794	3,684	1,010	-3,648	putative twin-arginine translocation pathway signal	transport across membrane
all1781	20,500	5,620	-3,648	unknown	unknown
all2200	14,361	3,937	-3,648	unknown	unknown
alr1537	6,731	1,845	-3,648	GCN5-related N-acetyltransferase	other enzymes
alr4585	8,663	2,375	-3,648	phosphate ABC transporter, periplasmic phosphate-binding protein	transport across membrane
alr4833	33,799	9,284	-3,641	cytochrome P450	electron transport chain
all0133	64,933	17,842	-3,639	unknown	unknown
all2916	3,626	0,000	-3,626	similar to DnaJ protein	resistance, adaptations to atypical conditions
alr2057	3,624	0,000	-3,624	shikimate 5-dehydrogenase; AroK	amino acids biosynthesis and metabolism
alr2481	520,298	143,648	-3,622	two-component sensor histidine kinase	regulatory functions
all0630	3,620	0,000	-3,620	unknown	unknown
alr1613	7,846	2,182	-3,595	PpiC-type peptidyl-prolyl cis-trans isomerase	posttranscriptional modifications
alr3064	4,214	1,172	-3,595	probable glycosyl transferase	sugars
alr4164	5,365	1,492	-3,595	amino acid ABC transporter, periplasmic amino acid-binding protein	transport across membrane
all4868	10,447	2,906	-3,595	transposase	transposon-related functions
asr7037	44,605	12,408	-3,595	unknown	unknown
all2375	13,595	3,782	-3,595	peroxiredoxin GCT3	detoxification
all1776	2,963	0,824	-3,595	unknown	unknown
all1782	19,334	5,378	-3,595	von Willebrand factor, type A	unknown
all2501	185,276	51,752	-3,580	4-hydroxy-3-methylbut-2-en-1-yl diphosphate synthase	steroids biosynthesis
alr0044	326,750	91,315	-3,578	unknown	unknown
asr4133	3,574	0,000	-3,574	unknown	unknown
alr4416	80,835	22,653	-3,568	cystathionine beta-synthase	amino acids biosynthesis and metabolism
all3555	15,554	4,366	-3,562	acetolactate synthase; IlvB	amino acids biosynthesis and metabolism
alr0487	796,791	223,829	-3,560	unknown	unknown
alr4815	23,634	6,653	-3,552	site-specific DNA-methyltransferase	DNA replication, recombination and repair
alr3761	33,726	9,494	-3,552	two-component hybrid sensor and regulator	regulatory functions
all2145	10,313	2,903	-3,552	transposase	transposon-related functions
alr0693	8,361	2,354	-3,552	NHL repeat-containing protein	unknown

alr3175	12,337	3,473	-3,552	unknown	unknown
all2852	389,517	109,841	-3,546	metallophosphoesterase	other enzymes
alr3844	23,009	6,496	-3,542	BNR repeat-containing glycosyl hydrolase	unknown
all1268	3,538	0,000	-3,538	unknown	unknown
asr9028	3,538	0,000	-3,538	unknown	unknown
alr5223	10,119	2,863	-3,534	glycosyl transferase	sugars
alr4491	28,946	8,203	-3,529	glucose-1-phosphate thymidylyltransferase	surface components
alr9018	4,480	1,271	-3,524	unknown	unknown
all1724	27,693	7,866	-3,521	unknown	unknown
alr2412	11,925	3,394	-3,513	serine/threonine kinase	regulatory functions
all2421	69,721	19,862	-3,510	unknown	unknown
alr2892	129,396	36,885	-3,508	unknown	unknown
all5339	3,503	0,000	-3,503	unknown	unknown
all3223	10,615	3,042	-3,489	unknown	unknown
alr2946	3,490	1,000	-3,489	unknown	unknown
alr2042	6,327	1,813	-3,489	von Willebrand factor, type A	unknown
all4390	6,116	1,753	-3,489	imidazoleglycerol-phosphate dehydratase	amino acids biosynthesis and metabolism
alr3307	3,260	0,934	-3,489	ankyrin	attachment of integral membrane proteins to the cytoskeleton
all0948	4,026	1,154	-3,489	heme O synthase	cobalamin, heme, phycobilin and porphyrin
alr1461	18,614	5,335	-3,489	fdxN element excision controlling factor protein XisH	heterocyst differentiation
alr4516	5,329	1,527	-3,489	probable protein phosphatase	other enzymes
alr3814	11,623	3,331	-3,489	phycocyanin alpha phycocyanobilin lyase related protein NblB	phycobilisomes and phycobiliproteins
all1692	12,320	3,531	-3,489	RNA polymerase sigma-subunit; SigC	RNA synthesis and modification
alr4773	1,647	0,472	-3,489	alpha-glucosidase	sugars
alr0584	3,203	0,918	-3,489	probable glycosyl transferase	sugars
alr3528	5,285	1,515	-3,489	putative beta-phosphoglucomutase	sugars
alr4781	5,932	1,700	-3,489	sugar ABC transporter, ATP-binding protein	transport across membrane
all1916	3,065	0,879	-3,489	sugar transport system, sugar-binding protein	transport across membrane
all2262	8,982	2,574	-3,489	unknown	unknown
all2913	24,511	7,025	-3,489	unknown	unknown
all3892	2,936	0,841	-3,489	unknown	unknown
all3941	3,800	1,089	-3,489	unknown	unknown
alr1386	2,564	0,735	-3,489	unknown	unknown
alr1808	4,026	1,154	-3,489	unknown	unknown
alr4876	10,034	2,876	-3,489	unknown	unknown
alr5180	5,489	1,573	-3,489	unknown	unknown
all3740	5,397	1,547	-3,489	heterocyst differentiation protein HetL	heterocyst differentiation
alr4976	2,446	0,701	-3,489	phosphodiesterase/alkaline phosphatase D	regulatory functions
all4713	3,857	1,105	-3,489	UDP-glucose 4-epimerase	sugars

all0995	24,233	6,946	-3,489	unknown	unknown
alr0230	2,396	0,687	-3,489	unknown	unknown
alr1060	8,506	2,438	-3,489	unknown	unknown
alr2696	8,678	2,487	-3,489	unknown	unknown
alr4911	9,294	2,664	-3,489	unknown	unknown
asl0817	3,485	0,000	-3,485	unknown	unknown
alr0451	40,509	11,644	-3,479	secretion protein HlyD	protein and peptide secretion
all0800	3,475	0,000	-3,475	molybdopterin precursor biosynthesis protein	coenzymes and cofactors
all7644	240,274	69,188	-3,473	unknown	unknown
alr1230	3,468	0,000	-3,468	two-component response regulator	regulatory functions
alr0653	230,064	66,663	-3,451	similar to rod shape-determining protein	cell shape
alr4171	59,849	17,364	-3,447	L-asparaginase II	amino acids biosynthesis and metabolism
alr1609	3,443	0,000	-3,443	transposase	transposon-related functions
alr1158	74,237	21,587	-3,439	ribonuclease III	RNA degradation
all2340	7,784	2,272	-3,426	aspartate transaminase	amino acids biosynthesis and metabolism
alr7323	12,661	3,696	-3,426	transposase	transposon-related functions
alr0045	164,839	48,120	-3,426	ferredoxin	electron transport chain
alr0029	6,656	1,948	-3,416	WD-40 repeat protein	regulatory functions
alr4239	29,423	8,616	-3,415	toxin secretion ABC transporter ATP-binding protein	transport across membrane
asr0043	565,457	165,652	-3,414	unknown	unknown
all1651	36,120	10,593	-3,410	transcriptional regulator	regulatory functions
alr3067	36,758	10,793	-3,406	probable glycosyl transferase	sugars
alr1460	403,849	118,594	-3,405	unknown	unknown
all1553	15,657	4,601	-3,403	NADH dehydrogenase	respiration, oxidative phosphorylation
alr2502	12,821	3,773	-3,398	serine/threonine kinase	regulatory functions
all0131	3,384	0,000	-3,384	unknown	unknown
all2371	3,384	0,000	-3,384	unknown	unknown
asr7140	3,384	0,000	-3,384	unknown	unknown
alr5360	6,769	2,001	-3,383	unknown	unknown
all4406	9,435	2,789	-3,383	NB-ARC domain containing protein	resistance, adaptations to atypical conditions
all3927	8,649	2,556	-3,383	unknown	unknown
all7130	2,439	0,721	-3,383	unknown	unknown
asr0654	28,306	8,366	-3,383	unknown	unknown
all2058	127,312	37,698	-3,377	unknown	unknown
all1697	45,086	13,364	-3,374	probable ribonuclease D	RNA synthesis and modification
alr4282	37,937	11,296	-3,358	heterocyst specific ABC-transporter, ATP-binding subunit DevA homolog	transport across membrane, heterocyst
all0747	12,238	3,650	-3,353	carboxysome formation protein CcmA	CO2 concentration mechanisms
alr2361	131,720	39,300	-3,352	mannose-1-phosphate guanyltransferase	sugars
all7012	86,410	25,892	-3,337	unknown	unknown
all1053	39,080	11,716	-3,336	glutamyl-tRNA (Gln) amidotransferase subunit A	aminoacyl tRNA synthetases and tRNA

					modification
alr1539	15,718	4,719	-3,330	cupin domain containing protein	unknown
all1248	45,618	13,697	-3,330	pyridoxamine 5-phosphate oxidase	vitamin biosynthesis
alr4062	80,983	24,316	-3,330	HTTM domain containing protein	unknown
all2995	39,232	11,780	-3,330	undecaprenyl pyrophosphate synthetase	carotenoids
all4307	28,130	8,446	-3,330	radical SAM domain-containing protein	energy production and conversion
alr3706	4,483	1,346	-3,330	serine/threonine kinase	regulatory functions
all4371	18,477	5,577	-3,313	peptidase M48, Ste24p	degradation of proteins, peptides, and glycopeptides
alr0547	8,058	2,432	-3,313	probable protein phosphatase	other enzymes
all3914	13,418	4,050	-3,313	succinyl-CoA synthetase beta chain	TCA cycle
all4335	246,974	74,641	-3,309	peptidase S16, lon domain-containing protein	degradation of proteins, peptides, and glycopeptides
all3733	3,305	0,000	-3,305	unknown	unknown
all2613	9,284	2,814	-3,299	transcriptional regulator	regulatory functions
alr3399	11,039	3,346	-3,299	chromosome segregation and condensation protein ScpA	unknown
all3458	17,548	5,320	-3,299	unknown	unknown
alr2993	1926,556	585,039	-3,293	DnaJ protein	heat shock proteins and chaperones
asr4809	3,289	0,000	-3,289	inorganic carbon transport; IctA	redox reactions
alr3905	3,280	0,000	-3,280	rhodanese-like	detoxification
all4704	3,280	0,000	-3,280	pyridoxamine 5'-phosphate oxidase	vitamin biosynthesis
alr2082	3,274	0,000	-3,274	cobalamin biosynthetic protein CobD	cobalamin, heme, phycobilin and porphyrin
all7137	3,274	0,000	-3,274	unknown	unknown
alr4852	3,274	0,000	-3,274	unknown	unknown
all5218	9,404	2,882	-3,262	putative modulator of DNA gyrase; PmbA	DNA replication, recombination and repair
all7151	25,630	7,856	-3,262	unknown	unknown
alr3213	3,261	0,000	-3,261	unknown	unknown
all3875	73,223	22,461	-3,260	phosphatidate cytidylyltransferase	lipid metabolism
all4442	24,915	7,655	-3,255	unknown	unknown
all2263	16,508	5,077	-3,251	heat shock protein X	heat shock proteins and chaperones
alr3701	124,659	38,359	-3,250	type II site-specific deoxyribonuclease	DNA degradation and modification
alr4448	93,287	28,834	-3,235	endo-1,4-beta-xylanase	sugars
alr2803	23,255	7,209	-3,226	pyruvate-flavodoxin dehydrogenase; nitrogen fixation protein NifJ	nitrogen fixation
alr3455	47,801	14,823	-3,225	septum site-determining protein MinC	cell division
alr3731	8,292	2,571	-3,225	protein serine-threonine phosphatase; PrpA	regulatory functions
all1210	3,218	0,000	-3,218	unknown	unknown
all4510	243,429	75,854	-3,209	FAD-dependent pyridine nucleotide-disulphide oxidoreductase	posttranslational modifications
asl7051	3,199	0,000	-3,199	unknown	unknown
alr0222	44,979	14,084	-3,194	citrate synthase; GltA	TCA cycle

all2931	320,909	100,711	-3,186	glyoxalase/bleomycin resistance protein/dioxygenase	resistance, adaptations to atypical conditions
alr9014	920,866	289,890	-3,177	unknown	unknown
all5327	2,085	0,657	-3,172	two-component sensor histidine kinase	regulatory functions
all8558	4,170	1,315	-3,172	unknown	unknown
asr0755	6,346	2,001	-3,172	unknown	unknown
alr0318	4,947	1,560	-3,172	carbon dioxide concentrating mechanism protein CcmK	CO2 concentration mechanisms
all0245	3,208	1,011	-3,172	tocopherol cyclase	coenzymes and cofactors
alr3815	0,703	0,222	-3,172	peptidase	degradation of proteins, peptides, and glycopeptides
alr0510	0,871	0,275	-3,172	type I site-specific deoxyribonuclease	DNA degradation and modification
all2684	3,243	1,023	-3,172	similar to restriction enzyme, restriction chain	DNA replication, recombination and repair
alr4060	4,885	1,540	-3,172	probable alginate O-acetylation protein	exopolysaccharide biosynthesis
alr4717	2,690	0,848	-3,172	hydrogenase accessory protein HupE	other enzymes
alr0512	2,170	0,684	-3,172	nucleoside triphosphate pyrophosphohydrolase	purine/pyrimidine biosynthesis and metabolism
alr4011	2,272	0,716	-3,172	probable oxidoreductase	redox reactions
alr3252	3,434	1,083	-3,172	short-chain dehydrogenase/reductase	redox reactions
alr1232	8,007	2,524	-3,172	predicted signal transduction protein containing Nacht domain	regulatory functions
alr4141	1,574	0,496	-3,172	serine/threonine kinase	regulatory functions
all0352	11,723	3,696	-3,172	NADPH-dependent carbonyl reductase	sugars
alr1521	4,979	1,570	-3,172	putative invertase	sugars
alr1793	2,006	0,632	-3,172	putative oligosaccharide deacetylase	sugars
all4956	1,471	0,464	-3,172	coproporphyrinogen III oxidase, HemF	tetrapyrrole biosynthesis pathway
all4219	2,115	0,667	-3,172	ABC-2 type transport system permease	transport across membrane
all8070	2,298	0,725	-3,172	transposase	transposon-related functions
all4401	1,574	0,496	-3,172	unknown	unknown
all4982	2,298	0,725	-3,172	unknown	unknown
all5281	5,780	1,822	-3,172	unknown	unknown
alr0381	1,557	0,491	-3,172	unknown	unknown
alr0452	4,314	1,360	-3,172	unknown	unknown
alr1378	2,245	0,708	-3,172	unknown	unknown
alr1790	5,121	1,615	-3,172	unknown	unknown
alr1923	5,809	1,831	-3,172	unknown	unknown
alr3592	0,565	0,178	-3,172	unknown	unknown
alr4505	1,622	0,511	-3,172	unknown	unknown
alr4606	0,756	0,238	-3,172	unknown	unknown
alr4720	5,724	1,804	-3,172	unknown	unknown
alr5271	9,267	2,922	-3,172	unknown	unknown
alr7079	3,057	0,964	-3,172	unknown	unknown
alr9505	5,724	1,804	-3,172	unknown	unknown

asl2557	7,207	2,272	-3,172	unknown	unknown
asl4482	8,982	2,832	-3,172	unknown	unknown
asr0636	13,737	4,331	-3,172	unknown	unknown
asr3881	10,615	3,346	-3,172	unknown	unknown
asr7230	11,447	3,609	-3,172	unknown	unknown
alr3527	3,892	1,227	-3,172	similar to ubiquinone/menaquinone biosynthesis methyltransferase	vitamin biosynthesis
alr2895	22,454	7,079	-3,172	imidazoleglycerol-phosphate synthase, cyclase subunit HisF	amino acids biosynthesis and metabolism
alr4746	5,897	1,859	-3,172	indole-3-glycerol phosphate synthase	amino acids biosynthesis and metabolism
alr3882	2,308	0,727	-3,172	tRNA methyltransferase	aminoacyl tRNA synthetases and tRNA modification
alr2903	2,138	0,674	-3,172	NAD-dependent epimerase/dehydratase	cell wall/membrane biogenesis
all7277	1,364	0,430	-3,172	HNH endonuclease	DNA degradation and modification
all2277	6,211	1,958	-3,172	aldo/keto reductase	other enzymes
alr0234	2,412	0,761	-3,172	phospholipid/glycerol acyltransferase	phospholipid biosynthesis, membrane biogenesis
all1512	3,261	1,028	-3,172	cytochrome b6/f-complex iron-sulfur protein PetC	photosynthesis
all0637	1,330	0,419	-3,172	two-component hybrid sensor and regulator	regulatory functions
all2095	1,411	0,445	-3,172	two-component sensor histidine kinase	regulatory functions
all2284	3,517	1,109	-3,172	sigma-B activity negative regulator; RsbW	regulatory functions
alr5126	4,054	1,278	-3,172	aminoglycoside phosphotransferase	resistance, adaptations to atypical conditions
alr2922	8,585	2,707	-3,172	glyoxalase/bleomycin resistance protein/dioxygenase	resistance, adaptations to atypical conditions
all3791	2,780	0,876	-3,172	ribonuclease D	RNA degradation
alr0075	2,373	0,748	-3,172	surface antigen (D15)	surface components
alr3795	5,033	1,587	-3,172	translation initiation factor Sui1	translation
alr1201	1,615	0,509	-3,172	ABC transporter ATP-binding protein	transport across membrane
all0640	3,202	1,009	-3,172	ABC transporter, ATP-binding protein	transport across membrane
all2904	17,917	5,649	-3,172	major facilitator superfamily transporter	transport across membrane
alr2363	1,240	0,391	-3,172	major facilitator transporter	transport across membrane
all0127	6,828	2,153	-3,172	unknown	unknown
all0231	4,537	1,430	-3,172	unknown	unknown
all0667	1,211	0,382	-3,172	unknown	unknown
all0918	2,422	0,764	-3,172	unknown	unknown
all1326	95,968	30,256	-3,172	unknown	unknown
all2333	1,959	0,618	-3,172	unknown	unknown
all2415	11,492	3,623	-3,172	unknown	unknown
all2788	3,971	1,252	-3,172	unknown	unknown
all3127	3,853	1,215	-3,172	unknown	unknown
all3669	4,457	1,405	-3,172	unknown	unknown
all3839	1,330	0,419	-3,172	unknown	unknown
all4348	1,474	0,465	-3,172	unknown	unknown

all4408	5,560	1,753	-3,172	unknown	unknown
all7197	2,820	0,889	-3,172	unknown	unknown
alr1377	12,388	3,906	-3,172	unknown	unknown
alr1910	1,966	0,620	-3,172	unknown	unknown
alr2889	10,363	3,267	-3,172	unknown	unknown
alr3565	1,659	0,523	-3,172	unknown	unknown
alr3718	2,308	0,727	-3,172	unknown	unknown
alr3729	4,200	1,324	-3,172	unknown	unknown
alr4836	3,672	1,158	-3,172	unknown	unknown
asr1064	95,849	30,218	-3,172	unknown	unknown
asr3218	11,015	3,473	-3,172	unknown	unknown
asr3299	12,163	3,835	-3,172	unknown	unknown
alr4386	6,081	1,917	-3,172	dihydropteroate synthase	vitamin biosynthesis
alr4438	4,561	1,438	-3,172	transposase	transposon-related functions
alr5141	3,156	0,000	-3,156	unknown	unknown
asl0026	3,156	0,000	-3,156	unknown	unknown
asl3973	3,156	0,000	-3,156	unknown	unknown
asr2504	3,156	0,000	-3,156	unknown	unknown
all3837	73,344	23,256	-3,154	unknown	unknown
all2550	94,470	30,118	-3,137	unknown	unknown
alr2465	3,135	0,000	-3,135	thioesterase superfamily protein	other enzymes
alr0946	127,229	40,620	-3,132	transcriptional regulator, AbrB family	regulatory functions
all1747	78,839	25,170	-3,132	unknown	unknown
alr2525	3,128	0,000	-3,128	unknown	unknown
all4196	37,237	11,939	-3,119	adenylate kinase	regulatory functions
all1454C	1,480	0,475	-3,114	nitrogenase molybdenum-iron protein alpha chain, Cend fragment (nifD3')	nitrogen fixation
asr4987	3,114	0,000	-3,114	unknown	unknown
alr3672	23,555	7,586	-3,105	aldehyde dehydrogenase	aldehyde metabolism
alr2385	7972,126	2567,522	-3,105	HesB/YadR/YfhF family protein	Fe-S cluster assembly accessory protein
alr4917	9,125	2,942	-3,101	unknown	unknown
alr1867	3,100	0,000	-3,100	transcriptional regulator	regulatory functions
alr1819	176,499	56,962	-3,099	beta-Ig-H3/fasciclin domain containing protein	unknown
alr4912	3,089	0,000	-3,089	unknown	unknown
all2929	3,084	0,000	-3,084	carbonic anhydrase; EcaA	other enzymes
alr0616	17,488	5,676	-3,081	PBS lyase HEAT domain protein repeat-containing protein	energy production and conversion
all0934	86,472	28,109	-3,076	site-specific DNA-methyltransferase	DNA replication, recombination and repair
all2381	3,073	0,000	-3,073	cyclase/dehydrase	other enzymes
asr3002	3,073	0,000	-3,073	aspartate carbamoyltransferase	purine/pyrimidine biosynthesis and metabolism
asr0890	3,073	0,000	-3,073	unknown	unknown
alr7249	31,844	10,374	-3,070	unknown	unknown

asl0885	220,831	72,022	-3,066	photosystem II protein PsbK	photosynthesis
all4826	10,749	3,506	-3,066	dTDP-glucose dehydratase	surface components
alr2414	29,573	9,645	-3,066	unknown	unknown
alr1343	26,732	8,719	-3,066	thiamin-phosphate pyrophosphorylase	vitamin biosynthesis
all1640	3,053	0,000	-3,053	two-component response regulator	regulatory functions
alr7276	3,053	0,000	-3,053	unknown	unknown
alr1911	9,927	3,254	-3,051	pyruvate flavodoxin dehydrogenase NifJ	nitrogen fixation
all0661	5,924	1,946	-3,045	adenylate cyclase	regulatory functions
alr4849	9,248	3,037	-3,045	methanol dehydrogenase regulatory protein	regulatory functions
alr0963	2,692	0,884	-3,045	TPR repeat-containing protein	unknown
asr4470	3,033	0,000	-3,033	unknown	unknown
all4936	7,772	2,564	-3,031	cell division protein FtsH	degradation of proteins, peptides, and glycopeptides
alr3546	9,281	3,068	-3,025	heterocyst differentiation protein HetF	heterocyst differentiation
all4429	3,023	0,000	-3,023	similar to hemolytic protein HlpA	resistance, adaptations to atypical conditions
alr4165	3,023	0,000	-3,023	amino acid ABC transporter, permease protein	transport across membrane
all3205	4,612	1,531	-3,013	glutamyl-tRNA synthetase; GltX	aminoacyl tRNA synthetases and tRNA modification
all4761	12,463	4,136	-3,013	dual specificity protein phosphatase	regulatory functions
all0443	7,624	2,530	-3,013	TPR repeat-containing protein	unknown
all2487	404,252	134,157	-3,013	TPR repeat-containing protein	unknown
alr0617	46,952	15,582	-3,013	unknown	unknown
alr2712	6,563	2,178	-3,013	unknown	unknown
alr4589	11,148	3,700	-3,013	unknown	unknown
all2410	4,384	1,455	-3,013	putative phenylacetate-CoA ligase	amino acids biosynthesis and metabolism
all0197	7,980	2,648	-3,013	unknown	unknown
all1987	13,050	4,331	-3,013	unknown	unknown
alr2408	8,803	2,922	-3,013	unknown	unknown
all3984	64,420	21,420	-3,007	unknown	unknown
all1124	3,007	0,000	-3,007	probable glutathione S-transferase	detoxification
asl1934	2,994	0,000	-2,994	unknown	unknown
asl4862	2,994	0,000	-2,994	unknown	unknown
asr3960	2,994	0,000	-2,994	unknown	unknown
alr1519	8,450	2,825	-2,991	amino acid transporter	transport across membrane
alr3936	21,831	7,300	-2,991	histidinol-phosphate aminotransferase	amino acids biosynthesis and metabolism
all0293	15,691	5,251	-2,988	unknown	unknown
asl4451	205,953	69,021	-2,984	30S ribosomal protein S18	ribosome component
alr2718	2,983	0,000	-2,983	probable cobyric acid synthase	tetrapyrrole biosynthesis pathway
alr1390	9,406	3,163	-2,974	unknown	unknown
all1480	2,968	0,000	-2,968	unknown	unknown
all2030	2,968	0,000	-2,968	unknown	unknown
all7319	885,564	298,794	-2,964	unknown	unknown

alr3222	2,963	0,000	-2,963	chromosome partitioning protein, ParA family	nucleoproteins
alr0763	2,963	0,000	-2,963	unknown	unknown
all3826	27,019	9,127	-2,960	peptidoglycan-binding domain 1	surface components
all4453	8,741	2,953	-2,960	RDD domain-containing protein	surface components
all0157	7,330	2,476	-2,960	unknown	unknown
alr0335	10,615	3,586	-2,960	threonyl-tRNA synthetase; ThrS	aminoacyl tRNA synthetases and tRNA modification
alr0286	32,477	10,970	-2,960	small heat shock protein	heat shock proteins and chaperones
alr5010	9,867	3,333	-2,960	D-hydantoinase	purine/pyrimidine biosynthesis and metabolism
alr3594	11,760	3,972	-2,960	two-component response regulator	regulatory functions
alr2176	5,061	1,710	-2,960	ABC transporter, iron(III) dicitrate-binding protein	transport across membrane
alr3811	20,692	6,990	-2,960	unknown	unknown
alr4821	9,261	3,128	-2,960	unknown	unknown
all3341	5,413	1,828	-2,960	heterodisulfide reductase, subunit B	vitamin biosynthesis
alr4114	2,956	0,000	-2,956	TspO and MBR related proteins	regulatory functions
asl1778	2,956	0,000	-2,956	unknown	unknown
alr0255	28,716	9,727	-2,952	unknown	unknown
all1738	21,729	7,428	-2,925	carboxyl-terminal protease	degradation of proteins, peptides, and glycopeptides
alr1792	2,919	0,000	-2,919	GDSL family lipase	lipid metabolism
alr7505	2,919	0,000	-2,919	unknown	unknown
all1318	5,292	1,813	-2,918	valyl-tRNA synthetase; ValS	aminoacyl tRNA synthetases and tRNA modification
alr4559	3,412	1,169	-2,918	WD-40 repeat-protein	regulatory functions
alr2450	10,134	3,473	-2,918	glycoside hydrolase	sugars
all2624	9,489	3,252	-2,918	unknown	unknown
alr0518	20,980	7,190	-2,918	unknown	unknown
alr3505	2,066	0,708	-2,918	unknown	unknown
alr2979	2,910	0,000	-2,910	heat shock protein DnaJ-like	heat shock proteins and chaperones
all5052	47,824	16,441	-2,909	phospholipid/glycerol acyltransferase	phospholipid biosynthesis, membrane biogenesis
all7235	7,609	2,617	-2,908	filamentous haemagglutinin-like protein	surface components
asr9501	85,625	29,449	-2,908	unknown	unknown
all4538	27,473	9,474	-2,900	mannose-6-phosphate isomerase	sugars
alr4043	31,136	10,737	-2,900	rhodanese domain-containing protein	detoxification
all3865	383,998	132,588	-2,896	molybdenum cofactor biosynthesis protein A	coenzymes and cofactors
alr4169	776,880	268,267	-2,896	Iojap-related protein	unknown
alr4235	12,338	4,269	-2,890	ornithine acetyl transferase	nitrogen metabolism
all2080	34,690	12,004	-2,890	transcriptional regulator AbrB	regulatory functions
all3785	40,171	13,917	-2,886	abortive infection protein	resistance, adaptations to atypical conditions
alr3955	135,769	47,084	-2,884	thioredoxin	redox homeostasis
asr5142	2,883	0,000	-2,883	unknown	unknown

alr2122	2,876	0,000	-2,876	unknown	unknown
all4687	4,729	1,646	-2,873	serine/threonine kinase with two-component sensor domain	regulatory functions
all4148	100,414	34,971	-2,871	ferredoxin I	electron transport chain
all0813	13,793	4,804	-2,871	heterocyst-specific glycolipids-directing protein HglK	heterocyst differentiation
alr2698	2,869	0,000	-2,869	transposase	transposon-related functions
all7121	17,472	6,091	-2,868	unknown	unknown
alr0096	6,487	2,272	-2,855	solanesyl diphosphate synthase; Sds	carotenoids
alr1894	4,170	1,461	-2,855	3-oxoacyl-[acyl-carrier protein] reductase	lipid metabolism
alr0545	5,126	1,796	-2,855	probable pseudouridine synthase	posttranscriptional modifications
alr0810	5,101	1,787	-2,855	transcriptional regulator	regulatory functions
all1985	3,562	1,248	-2,855	glycyl-tRNA synthetase alpha chain	aminoacyl tRNA synthetases and tRNA modification
all1757	3,491	1,223	-2,855	cell-division protein	cell division
alr1615	0,725	0,254	-2,855	subtilase family protein	degradation of proteins, peptides, and glycopeptides
alr1381	19,020	6,663	-2,855	trypsin; PrcA	degradation of proteins, peptides, and glycopeptides
all4591	2,994	1,049	-2,855	isopentenyl pyrophosphate isomerase	energy production and conversion
alr0540	8,432	2,954	-2,855	1-pyrroline-5 carboxylate dehydrogenase	glutamate metabolism and arginine and proline metabolism
all8020	16,292	5,707	-2,855	similar to 8R-lipoxygenase-allene oxide synthase fusion protein	redox reactions
asr7363	24,438	8,561	-2,855	CopG/Arc/MetJ DNA-binding domain transcriptional regulator	regulatory functions
all4084	5,417	1,897	-2,855	FHA domain containing protein	regulatory functions
alr9013	4,691	1,643	-2,855	two-component response regulator	regulatory functions
all0330	3,132	1,097	-2,855	two-component sensor histidine kinase	regulatory functions
all1145	3,274	1,147	-2,855	two-component sensor histidine kinase	regulatory functions
all4055	3,204	1,122	-2,855	glycine cleavage T-protein; aminomethyltransferase	salvage pathways
alr2434	2,640	0,925	-2,855	major facilitator superfamily MFS_1	transport across membrane
alr3493	5,838	2,045	-2,855	RNA-directed DNA polymerase (Reverse transcriptase)	transposon-related functions
all0479	2,367	0,829	-2,855	unknown	unknown
all0802	2,013	0,705	-2,855	unknown	unknown
all2267	10,104	3,540	-2,855	unknown	unknown
all3398	8,042	2,817	-2,855	unknown	unknown
all5107	1,186	0,415	-2,855	unknown	unknown
all7660	2,660	0,932	-2,855	unknown	unknown
alr1870	3,002	1,052	-2,855	unknown	unknown

alr3596	21,779	7,629	-2,855	unknown	unknown
alr5224	7,247	2,539	-2,855	unknown	unknown
alr7604	29,716	10,409	-2,855	unknown	unknown
alr4036	5,389	1,888	-2,855	pyridoxamine 5'-phosphate oxidase-related, FMN-binding	vitamin biosynthesis
alr5286	4,105	1,438	-2,855	probable short-chain dehydrogenase	other enzymes
all0139	9,730	3,408	-2,855	unknown	unknown
all4006	8,210	2,876	-2,855	unknown	unknown
asr3848	2,848	0,000	-2,848	photosystem II protein J	photosynthesis
asr7087	2,848	0,000	-2,848	unknown	unknown
all0664	11,739	4,134	-2,840	WD-40 repeat protein	regulatory functions
alr3352	64,446	22,708	-2,838	probable rRNA methylase	RNA synthesis and modification
alr3705	74,890	26,402	-2,837	probable sugar transporter	transport across membrane
all1729	41,274	14,553	-2,836	unknown	unknown
alr3265	38,561	13,602	-2,835	glutamate-1-semialdehyde 2,1-aminomutase	tetrapyrrole biosynthesis pathway
alr0221	2,831	0,000	-2,831	phosphohistidine Phosphatase, SixA	regulatory functions
asl0846	181,628	64,274	-2,826	photosystem II protein PsbH	photosynthesis
asl4452	176,040	62,296	-2,826	50S ribosomal protein L33	ribosome component
alr3230	17,991	6,367	-2,826	4-diphosphocytidyl-2C-methyl-D-erythritol kinase	terpenoid biosynthesis
all4497	23,236	8,241	-2,819	unknown	unknown
all3180	5,904	2,094	-2,819	similar to adenylate cyclase	regulatory functions
alr0091	19,220	6,817	-2,819	Na+/H+-exchanging protein	transport across membrane
alr2784	19,715	7,003	-2,815	metal dependent phosphohydrolase	other enzymes
all3220	13,020	4,635	-2,809	beta-lactamase domain-containing protein	resistance, adaptations to atypical conditions
all0592	23,434	8,352	-2,806	ABC-1 domain protein	protein amino acid phosphorylation
asr3137	478,298	170,752	-2,801	unknown	unknown
all7026	2,331	0,835	-2,791	Type III restriction enzyme, res subunit	DNA degradation and modification
alr1543	2,780	0,000	-2,780	tRNA pseudouridine synthase B	aminoacyl tRNA synthetases and tRNA modification
asr4899	2,780	0,000	-2,780	unknown	unknown
alr2495	9,707	3,498	-2,775	cysteine desulphurases, SufS	Fe-S cluster biosynthesis
asl7365	44,908	16,181	-2,775	unknown	unknown
alr1788	8,108	2,922	-2,775	similar to zeta-carotene desaturase	carotenoids
all0687C	5,271	1,902	-2,771	[NiFe] uptake hydrogenase large subunit, Cend fragment (hupL3')	photosynthesis
asr3992	266,882	96,411	-2,768	photosystem II reaction center protein PsbZ	photosynthesis
alr4157	11,187	4,041	-2,768	NADH dehydrogenase subunit 4	respiration, oxidative phosphorylation
alr5154	3762,6	1363,442	-2,760	photosystem I core protein A1	photosynthesis
all3842	456,429	165,804	-2,753	NADH dehydrogenase subunit 3	respiration, oxidative phosphorylation
all1989	1,946	0,708	-2,749	single-strand-DNA-specific exonuclease; RecJ	DNA replication, recombination and repair
alr2392	8,433	3,068	-2,749	filament integrity protein FraC	filament integrity
all4945	23,942	8,709	-2,749	unknown	unknown

alr3379	6,459	2,350	-2,749	NLP/P60 protein	cell wall/membrane biogenesis
all4090	4,048	1,472	-2,749	similar to carboxyl-terminal processing proteinase	degradation of proteins, peptides, and glycopeptides
all4427	4,865	1,770	-2,749	similar to phytanoyl-CoA hydroxylase	lipid metabolism
asr0773	15,811	5,752	-2,749	Mo-dependent nitrogenase-like	nitrogen fixation
all7310	1,941	0,706	-2,749	adenylate cyclase	regulatory functions
alr0068	16,410	5,969	-2,749	adenylate kinase	regulatory functions
all0219	2,590	0,942	-2,749	diguanylate cyclase/phosphodiesterase with PAS/PAC sensor	regulatory functions
all4083	4,649	1,691	-2,749	FHA domain-containing protein	regulatory functions
all5308	3,466	1,261	-2,749	two-component hybrid sensor and regulator	regulatory functions
alr0366	1,316	0,479	-2,749	filamentous haemagglutinin-like protein	surface components
alr0153	2,821	1,026	-2,749	penicillin-binding protein	surface components
all2702	23,967	8,719	-2,749	unknown	unknown
all4050	14,011	5,097	-2,749	unknown	unknown
all4663	11,499	4,183	-2,749	unknown	unknown
alr0436	6,445	2,345	-2,749	unknown	unknown
alr1114	6,000	2,182	-2,749	unknown	unknown
asr3250	21,684	7,888	-2,749	unknown	unknown
asr3831	28,639	10,418	-2,749	unknown	unknown
asr4154	48,964	17,812	-2,749	unknown	unknown
alr3573	2,747	0,000	-2,747	FO synthase subunit 1	coenzymes and cofactors
asr2932	2,747	0,000	-2,747	unknown	unknown
alr0165	49,006	17,913	-2,736	UvrD/REP helicase	DNA replication, recombination and repair
asl2329	96,156	35,187	-2,733	unknown	unknown
alr2393	10,183	3,745	-2,719	FraD	filament integrity
alr2572	6,992	2,572	-2,719	two-component sensor histidine kinase	regulatory functions
all3136	8,281	3,046	-2,719	polyA polymerase	RNA synthesis and modification
alr4030	12,037	4,427	-2,719	unknown	unknown
alr4393	82,461	30,368	-2,715	unknown	unknown
alr9029	2,715	0,000	-2,715	unknown	unknown
all2086	15,913	5,864	-2,714	unknown	unknown
alr3751	80,442	29,809	-2,699	ferrochelatase	tetrapyrrole biosynthesis pathway
alr7646	32,275	11,971	-2,696	MobB protein	plasmid mobilization
alr3730	4,306	1,597	-2,696	unknown	unknown
all2315	17,936	6,653	-2,696	ketol-acid reductoisomerase	amino acids biosynthesis and metabolism
all2564	4,161	1,543	-2,696	pyruvate kinase	glycolisis
asl3117	224,234	83,662	-2,680	unknown	unknown
all1051	11,377	4,247	-2,678	glycerophosphoryl diester phosphodiesterase	lipid metabolism
all2413	11,065	4,131	-2,678	unknown	unknown
all2971	41,858	15,628	-2,678	putative regulator	regulatory functions
alr7514	36,643	13,706	-2,673	unknown	unknown

all1425	16,058	6,007	-2,673	unknown	unknown
asr0064	354,717	132,801	-2,671	unknown	unknown
alr3539	43,630	16,375	-2,664	unknown	unknown
alr0130	20,779	7,799	-2,664	alpha/beta hydrolase fold protein	other enzymes
all2563	22,090	8,291	-2,664	transaldolase; Tal	pentose phosphate pathway
alr2208	7,385	2,772	-2,664	transcriptional regulator	regulatory functions
all5122	20,433	7,669	-2,664	unknown	unknown
alr3251	9,015	3,383	-2,664	unknown	unknown
all1288	283,389	106,408	-2,663	GDSL family lipase	lipid metabolism
asl4195	137,000	51,536	-2,658	translation initiation factor IF-1	translation
all4023	38,023	14,321	-2,655	cytochrome D ubiquinol oxidase chain II	nitrogen metabolism
asr1828	2,654	0,000	-2,654	putative calcium-translocating P-type ATPase	transport across membrane
asl2181	2,654	0,000	-2,654	unknown	unknown
asl4204	45,517	17,158	-2,653	50S ribosomal protein L24	ribosome component
all1939	5,690	2,153	-2,643	processing proteinase	degradation of proteins, peptides, and glycopeptides
all7376	12,474	4,719	-2,643	transposase	transposon-related functions
alr1726	16,776	6,347	-2,643	transposase	transposon-related functions
all2608	8,740	3,306	-2,643	unknown	unknown
all4916	10,953	4,144	-2,643	unknown	unknown
all2655	1,789	0,677	-2,643	unknown	unknown
all0384	2,637	0,000	-2,637	rhodanese domain-containing protein	detoxification
alr0622	23,011	8,733	-2,635	TPR repeat-containing protein	unknown
alr3864	15,882	6,027	-2,635	unknown	unknown
all2196	2,634	0,000	-2,634	PilT protein domain protein	chemotaxis and cell motility
all3520	87,382	33,249	-2,628	cytochrome c biogenesis membrane protein	posttranslational modifications
all4608	103,391	39,340	-2,628	glycine cleavage system protein H	salvage pathways
all2025	3,089	1,176	-2,628	similar to MrsD protein	polysacharides and glycoproteins
asr1049	2,624	0,000	-2,624	unknown	unknown
alr1297	11,189	4,267	-2,622	ABC transporter ATP binding protein	transport across membrane
alr1492	29,791	11,362	-2,622	unknown	unknown
alr3301	13,860	5,297	-2,617	unknown	unknown
alr0986	23,858	9,117	-2,617	unknown	unknown
alr0235	85,147	32,559	-2,615	alpha/beta fold family hydrolase	other enzymes
all2508	19,911	7,618	-2,614	GTP-binding elongation factor LepA	translation
all2056	2,611	0,000	-2,611	alpha/beta fold family hydrolase	other enzymes
alr3219	41,540	15,928	-2,608	unknown	unknown
alr5155	1426,152	549,443	-2,596	photosystem I core protein A2	photosynthesis
alr2936	9,821	3,784	-2,595	pantothenate synthetase PanC	vitamin biosynthesis
all4972	2,595	0,000	-2,595	transcriptional regulator, PadR family	regulatory functions
alr7515	2,595	0,000	-2,595	unknown	unknown
all3275	19,285	7,445	-2,590	two-component hybrid sensor and regulator	regulatory functions

all4212	19,866	7,669	-2,590	50S ribosomal protein L2	ribosome component
all3631	2,586	0,000	-2,586	type II site-specific deoxyribonuclease; AvaIR	DNA degradation and modification
all4736	33,450	12,934	-2,586	pyridoxamine 5'-phosphate oxidase-related, FMN-binding	vitamin biosynthesis
all2906	18,216	7,061	-2,580	molybdopterin biosynthesis protein MoeB	coenzymes and cofactors
asl0272	136,970	53,093	-2,580	unknown	unknown
all9023	15,087	5,860	-2,575	unknown	unknown
all2777	94,576	36,811	-2,569	RNA binding protein; RbpE	nucleoproteins
asl1305	2,566	0,000	-2,566	unknown	unknown
alr1148	2,559	0,000	-2,559	unknown	unknown
all4428	2,551	0,000	-2,551	O-antigen polymerase	cell wall/membrane biogenesis
alr3930	2,549	0,000	-2,549	peptidoglycan binding domain-containing protein	surface components
asr3846	2,538	0,000	-2,538	cytochrome b559 beta subunit	photosynthesis
all7165	2,538	0,000	-2,538	unknown	unknown
all0233	10,555	4,159	-2,537	methionyl-tRNA synthetase	aminoacyl tRNA synthetases and tRNA modification
all1269	1,390	0,548	-2,537	tryptophanyl-tRNA synthetase; TrpS	aminoacyl tRNA synthetases and tRNA modification
alr1108	1,857	0,732	-2,537	3-octaprenyl-4hydroxybenzoate decarboxylase	coenzymes and cofactors
all0961	2,245	0,885	-2,537	molybdopterin-guanine dinucleotide biosynthesis protein A	coenzymes and cofactors
alr1270	5,893	2,322	-2,537	proline iminopeptidase	degradation of proteins, peptides, and glycopeptides
all1940	8,829	3,479	-2,537	protease	degradation of proteins, peptides, and glycopeptides
alr0672	2,152	0,848	-2,537	similar to vanadium chloroperoxidase	detoxification
alr4512	8,609	3,393	-2,537	sulfide-quinone reductase	detoxification
alr3451	2,004	0,790	-2,537	ATP-dependent helicase	DNA replication, recombination and repair
all3284	3,243	1,278	-2,537	cytosine deaminase	DNA replication, recombination and repair
alr7570	1,086	0,428	-2,537	DNA polymerase III gamma and tau subunits	DNA replication, recombination and repair
alr2323	4,961	1,955	-2,537	heat shock protein HtpG	heat shock proteins and chaperones
alr1245	7,836	3,088	-2,537	acetylglutamate kinase; ArgB	lipid metabolism
alr4798	19,800	7,803	-2,537	argininosuccinate synthase	nitrogen metabolism
alr0987	4,491	1,770	-2,537	nitrogen regulatory protein P-II (GlnB, GlnK)	nitrogen metabolism
alr3248	3,073	1,211	-2,537	putative ammonia monooxygenase	nitrogen metabolism
all1235	1,996	0,787	-2,537	probable amidase	other enzymes
alr4542	7,656	3,017	-2,537	probable methyltransferase	other enzymes
alr1362	4,899	1,931	-2,537	putative carboxymethylenebutenolidase	other enzymes
all2561	1,150	0,453	-2,537	acetate kinase; AckA	pyruvate and acetyl-CoA metabolism
asl7641	5,560	2,191	-2,537	thioredoxin	redox homeostasis
alr3655	9,341	3,681	-2,537	photomixotrophic growth related protein, PmgA homolog	regulatory functions

all0192	0,843	0,332	-2,537	serine/threonine kinase	regulatory functions
all3773	0,660	0,260	-2,537	serine/threonine kinase	regulatory functions
all2035	4,969	1,958	-2,537	transcriptional regulator	regulatory functions
all3743	3,860	1,521	-2,537	transcriptional regulator	regulatory functions
all4925	1,370	0,540	-2,537	transcriptional regulator	regulatory functions
all1178	0,506	0,199	-2,537	two-component hybrid sensor and regulator	regulatory functions
all3759	1,176	0,464	-2,537	two-component response regulator	regulatory functions
alr3037	2,353	0,927	-2,537	two-component sensor histidine kinase	regulatory functions
all2352	11,741	4,627	-2,537	WD-40 repeat protein	regulatory functions
all2238	3,876	1,527	-2,537	serine/threonine protein kinase	regulatory functions
all4080	1,698	0,669	-2,537	ribosomal large chain pseudouridine synthase A	RNA synthesis and modification
all5194	1,207	0,476	-2,537	glycosyl transferase	sugars
alr0819	5,790	2,282	-2,537	putative invertase	sugars
alr3576	1,433	0,565	-2,537	adhesin precursor	surface components
all4345	1,526	0,601	-2,537	polysaccharide deacetylase	surface components
alr0058	1,366	0,538	-2,537	D-lactate dehydrogenase	TCA cycle
all5282	5,077	2,001	-2,537	ABC transporter sugar permease	transport across membrane
alr4068	3,844	1,515	-2,537	ABC transporter, ATP-binding protein	transport across membrane
all2147	3,871	1,525	-2,537	ABC transporter, ferrichrome binding protein	transport across membrane
all3334	3,538	1,394	-2,537	ABC transporter, nitrate transport ATP-binding protein NrtC	transport across membrane
alr0299	1,201	0,473	-2,537	ABC transporter, periplasmic polyamine-binding protein	transport across membrane
all2158	0,531	0,209	-2,537	ferrichrome-iron receptor	transport across membrane
alr0656	5,240	2,065	-2,537	Na+/H+ antiporter	transport across membrane
all2357	3,813	1,503	-2,537	phosphonate ABC transport ATP-binding component	transport across membrane
alr2835	0,768	0,303	-2,537	ABC transporter, ATP-binding protein; heterocyst differentiation protein HepA	transport across membrane, heterocyst
all5014	1,650	0,650	-2,537	RNA-directed DNA polymerase (Reverse transcriptase)	transposon-related functions
all1971	2,731	1,076	-2,537	transposase	transposon-related functions
all4867	2,731	1,076	-2,537	transposase	transposon-related functions
all7303	7,984	3,146	-2,537	transposase	transposon-related functions
all4647	3,336	1,315	-2,537	beta-Ig-H3/fasciclin	unknown
all0134	2,407	0,949	-2,537	unknown	unknown
all0307	4,061	1,600	-2,537	unknown	unknown
all0711	0,729	0,287	-2,537	unknown	unknown
all1287	23,159	9,127	-2,537	unknown	unknown
all1479	0,592	0,233	-2,537	unknown	unknown
all2871	2,289	0,902	-2,537	unknown	unknown
all2909	6,802	2,680	-2,537	unknown	unknown

all3049	14,371	5,663	-2,537	unknown	unknown
all3209	3,774	1,487	-2,537	unknown	unknown
all3613	0,720	0,284	-2,537	unknown	unknown
all3675	2,445	0,964	-2,537	unknown	unknown
all4117	55,546	21,890	-2,537	unknown	unknown
all4218	3,053	1,203	-2,537	unknown	unknown
all4381	2,525	0,995	-2,537	unknown	unknown
all5082	4,469	1,761	-2,537	unknown	unknown
all5342	1,139	0,449	-2,537	unknown	unknown
all7625	2,379	0,937	-2,537	unknown	unknown
alr1242	2,104	0,829	-2,537	unknown	unknown
alr1785	2,278	0,898	-2,537	unknown	unknown
alr2562	3,384	1,334	-2,537	unknown	unknown
alr2574	1,594	0,628	-2,537	unknown	unknown
alr3077	2,245	0,885	-2,537	unknown	unknown
alr3106	3,892	1,534	-2,537	unknown	unknown
alr3495	1,668	0,657	-2,537	unknown	unknown
alr4275	3,860	1,521	-2,537	unknown	unknown
alr4532	18,259	7,196	-2,537	unknown	unknown
alr4938	30,727	12,109	-2,537	unknown	unknown
alr8557	3,434	1,353	-2,537	unknown	unknown
asl5079	4,718	1,859	-2,537	unknown	unknown
asr1486	7,656	3,017	-2,537	unknown	unknown
asr4108	7,784	3,068	-2,537	unknown	unknown
asr5004	6,578	2,592	-2,537	unknown	unknown
asr7657	6,145	2,422	-2,537	unknown	unknown
all2109	4,513	1,778	-2,537	von Willebrand factor, type A	unknown
all0395	0,867	0,341	-2,537	L-2,4-diaminobutyrate decarboxylase	amine and polyamine biosynthesis
all1354	1,294	0,510	-2,537	FAD dependent oxidoreductase	amino acids biosynthesis and metabolism
all3432	1,803	0,711	-2,537	undecaprenyl pyrophosphate synthetase	carotenoids
all5305	1,599	0,630	-2,537	3-beta hydroxysteroid dehydrogenase/isomerase	cell wall/membrane biogenesis
all1069	1,270	0,501	-2,537	methyl-accepting chemotaxis protein	chemotaxis and cell motility
alr3448	1,464	0,577	-2,537	tocopherol phytyltransferase	coenzymes and cofactors
alr1779	4,195	1,653	-2,537	similar to cyanophycin synthetase	cyanophycin biosynthesis
all4358	2,113	0,833	-2,537	ATP-dependent Clp protease proteolytic subunit	degradation of proteins, peptides, and glycopeptides
all7071	0,624	0,246	-2,537	exodeoxyribonuclease V, alpha chain	DNA replication, recombination and repair
alr2445	45,016	17,740	-2,537	heat shock protein GrpE	heat shock proteins and chaperones
alr9027	3,033	1,195	-2,537	chromosome partitioning protein, ParB family	nucleoproteins
alr3102	2,595	1,023	-2,537	HAD-superfamily hydrolase subfamily IIIA	other enzymes
alr0694	1,783	0,703	-2,537	hydrogenase maturation protein HypF	other enzymes
asr1283	10,379	4,090	-2,537	photosystem I 4.8K protein PsaX	photosynthesis

alr4592	2,588	1,020	-2,537	photosystem II protein D1	photosynthesis
asr8504	15,066	5,937	-2,537	phycobilisome degradation protein NblA	phycobilisomes and phycobiliproteins
alr1336	2,271	0,895	-2,537	serine/threonine kinase	regulatory functions
alr2682	0,480	0,189	-2,537	serine/threonine kinase with two-component sensor domain	regulatory functions
alr0803	3,274	1,290	-2,537	signal transduction histidine kinase, LytS	regulatory functions
all4279	1,578	0,622	-2,537	transcriptional regulator	regulatory functions
alr1044	2,371	0,934	-2,537	transcriptional regulator	regulatory functions
all2239	2,734	1,078	-2,537	two-component hybrid sensor and regulator	regulatory functions
alr3547	1,018	0,401	-2,537	two-component sensor histidine kinase	regulatory functions
all1964	1,971	0,777	-2,537	two-component system response regulator	regulatory functions
alr4908	6,936	2,734	-2,537	SOS function regulatory protein, LexA repressor	resistance, adaptations to atypical conditions
all1864	1,868	0,736	-2,537	similar to NADH dehydrogenase	respiration, oxidative phosphorylation
all4336	8,812	3,473	-2,537	30S ribosomal protein S10	ribosome component
alr1050	6,180	2,436	-2,537	glucose-6-phosphate isomerase	sugars
all0601	2,616	1,031	-2,537	glycosyl transferase	sugars
alr4347	2,715	1,070	-2,537	glycosyl transferase	sugars
all0915	7,733	3,047	-2,537	probable glycosyl transferase	sugars
alr1668	1,091	0,430	-2,537	putative glycosyl transferase	sugars
alr1384	1,290	0,508	-2,537	ABC transporter ATP-binding protein	transport across membrane
all1948	1,891	0,745	-2,537	ABC transporter, ATP-binding protein	transport across membrane
all2148	1,081	0,426	-2,537	ferrichrome-iron receptor	transport across membrane
all4285	3,941	1,553	-2,537	folate/biopterin transporter	transport across membrane
all2585	1,346	0,530	-2,537	iron(III) dicitrate transport system permease protein	transport across membrane
alr0738	1,511	0,596	-2,537	sugar ABC transporter, permease protein	transport across membrane
all4768	5,431	2,140	-2,537	ErfK/YbiS/YcfS/YnhG family protein	unknown
all4662	22,783	8,978	-2,537	nucleotide-binding protein	unknown
all4659	15,765	6,213	-2,537	TPR repeat-containing protein	unknown
all0406	1,109	0,437	-2,537	unknown	unknown
all0466	1,705	0,672	-2,537	unknown	unknown
all0768	3,156	1,244	-2,537	unknown	unknown
all0861	4,277	1,686	-2,537	unknown	unknown
all1003	3,512	1,384	-2,537	unknown	unknown
all2703	6,894	2,717	-2,537	unknown	unknown
all2756	3,485	1,374	-2,537	unknown	unknown
all3034	3,975	1,566	-2,537	unknown	unknown
all3110	1,730	0,682	-2,537	unknown	unknown
all3433	3,003	1,184	-2,537	unknown	unknown
all3615	0,751	0,296	-2,537	unknown	unknown
all4295	8,703	3,430	-2,537	unknown	unknown
all4407	3,649	1,438	-2,537	unknown	unknown

all7265	14,710	5,797	-2,537	unknown	unknown
alr0053	2,580	1,017	-2,537	unknown	unknown
alr0501	3,409	1,343	-2,537	unknown	unknown
alr0901	1,639	0,646	-2,537	unknown	unknown
alr1085	1,824	0,719	-2,537	unknown	unknown
alr2141	1,424	0,561	-2,537	unknown	unknown
alr2471	3,199	1,261	-2,537	unknown	unknown
alr3825	1,567	0,618	-2,537	unknown	unknown
alr5087	14,945	5,890	-2,537	unknown	unknown
alr5225	1,109	0,437	-2,537	unknown	unknown
alr5239	2,395	0,944	-2,537	unknown	unknown
alr7300	1,730	0,682	-2,537	unknown	unknown
alr7311	6,919	2,727	-2,537	unknown	unknown
alr7341	1,541	0,607	-2,537	unknown	unknown
asr1857	12,131	4,781	-2,537	unknown	unknown
asr3478	5,189	2,045	-2,537	unknown	unknown
alr3381	2,525	0,000	-2,525	putative antibiotic efflux protein	resistance, adaptations to atypical conditions
all1783	101,154	40,092	-2,523	unknown	unknown
all7589	2,516	0,000	-2,516	unknown	unknown
asr2474	2,511	0,000	-2,511	phosphoribosylformylglycinamidine synthase subunit PurS	purine/pyrimidine biosynthesis and metabolism
asr0905	2,511	0,000	-2,511	unknown	unknown
all7623	2,506	0,000	-2,506	phosphoesterase, PA-phosphatase related protein	other enzymes
all1947	2,506	0,000	-2,506	ABC transporter, ATP-binding protein	transport across membrane
all0012	36,780	14,725	-2,498	methyltransferase type 11	other enzymes
alr2313	27,044	10,827	-2,498	unknown	unknown
all7122	2,495	0,000	-2,495	probable transporter	transport across membrane
all4338	123,163	49,401	-2,493	translation elongation factor EF-G	translation
all3969	122,541	49,203	-2,491	30S ribosomal protein S14	ribosome component
all4396	38,812	15,615	-2,485	polyribonucleotide nucleotidyltransferase	RNA synthesis and modification
alr2554	13,255	5,335	-2,485	unknown	unknown
asl3253	2,484	0,000	-2,484	RNA-binding S4 domain-containing protein	nucleoproteins
alr4271	20,164	8,131	-2,480	unknown	unknown
all1717	7,405	2,986	-2,480	DNA ligase	DNA replication, recombination and repair
alr4488	12,738	5,148	-2,474	glycosyl transferase	sugars
all0122	35,954	14,590	-2,464	pyruvate dehydrogenase E1 beta subunit	pyruvate and acetyl-CoA metabolism
all4450	349,773	142,263	-2,459	ribonuclease II	RNA degradation
alr2418	4,109	1,671	-2,458	alanyl-tRNA synthetase	aminoacyl tRNA synthetases and tRNA modification
alr4359	8,639	3,514	-2,458	von Willebrand factor, type A	unknown
asr4471	2,458	0,000	-2,458	plasmid stabilization system	plasmid mobilization
all2416	2,454	0,000	-2,454	putative diguanylate cyclase	regulatory functions

alr5303	24,065	9,835	-2,447	50S ribosomal protein L12	ribosome component
alr4893	4,657	1,903	-2,447	surface antigen (D15)	surface components
all2997	6,780	2,771	-2,447	diaminopimelate decarboxylase	amino acids biosynthesis and metabolism
alr3954	13,191	5,391	-2,447	NnrU family protein	unknown
alr2975	2,445	0,000	-2,445	signal peptidase I	degradation of proteins, peptides, and glycopeptides
all3503	38,625	15,803	-2,444	probable integrase	transposon-related functions
all0118	91,475	37,515	-2,438	GCN5-related N-acetyltransferase	other enzymes
alr1535	2,433	0,000	-2,433	unknown	unknown
asr4227	2,433	0,000	-2,433	unknown	unknown
alr0900	1,491	0,613	-2,432	serine/threonine kinase with two-component sensor domain	regulatory functions
all1750	7,586	3,120	-2,432	similar to WD-repeat containing protein	involved in different biological functions
alr3692	669,020	275,117	-2,432	MscS Mechanosensitive ion channel	transport across membrane
alr4642	12,549	5,160	-2,432	putative thiol-specific antioxidant protein	detoxification
alr3055	4,762	1,958	-2,432	DNA mismatch repair protein MutL	DNA replication, recombination and repair
all0769	2,929	1,204	-2,432	acetyl-CoA synthetase	pyruvate and acetyl-CoA metabolism
all5119	12,152	4,997	-2,432	integral membrane protein, DedA family	unknown
all5020	14,595	6,002	-2,432	unknown	unknown
alr2440	13,245	5,494	-2,411	unknown	unknown
alr0354	20,377	8,464	-2,408	serine/threonine kinase with two-component sensor domain	regulatory functions
asr4600	2,407	0,000	-2,407	unknown	unknown
alr0880	14,450	6,022	-2,400	oligopeptidase A	degradation of proteins, peptides, and glycopeptides
all2422	197,255	82,268	-2,398	unknown	unknown
all0070	14,649	6,113	-2,397	superoxide dismutase	detoxification
asl2061	42,687	17,812	-2,397	unknown	unknown
all1035	2,383	0,000	-2,383	transcriptional regulator	regulatory functions
all4816	2,383	0,000	-2,383	transposase	transposon-related functions
all3502	2,383	0,000	-2,383	unknown	unknown
alr9025	2,383	0,000	-2,383	unknown	unknown
all4499	39,276	16,488	-2,382	S-layer region-like protein	surface components
alr0030	2,963	1,246	-2,379	similar to Mg-protoporphyrin IX monomethyl ester oxidative cyclase	chlorophyll biosynthesis
alr1963	25,788	10,840	-2,379	endopeptidase Clp ATP-binding chain	degradation of proteins, peptides, and glycopeptides
all3033	3,791	1,594	-2,379	arsenical pump membrane protein	detoxification
alr0094	5,978	2,513	-2,379	glutamate racemase	nitrogen metabolism
all4520	4,457	1,873	-2,379	anhydro-N-acetylmuramic acid kinase	peptidoglycan recycling
all1279	3,510	1,475	-2,379	two-component hybrid sensor and regulator	regulatory functions
all1011	45,373	19,073	-2,379	maltose transacetylase	sugars

all1708	8,714	3,663	-2,379	unknown	unknown
all1755	13,169	5,536	-2,379	unknown	unknown
all1974	2,895	1,217	-2,379	unknown	unknown
all3604	9,519	4,001	-2,379	unknown	unknown
all7686	8,890	3,737	-2,379	unknown	unknown
alr2321	11,996	5,043	-2,379	lactoylglutathione lyase	detoxification
all1988	20,131	8,462	-2,379	cyclopropane-fatty-acyl-phospholipid synthase	lipid metabolism
all4355	8,420	3,540	-2,379	similar to DnaJ protein	resistance, adaptations to atypical conditions
alr4755	4,825	2,028	-2,379	pyridoxal phosphate biosynthetic protein PdxA	vitamin biosynthesis
alr1080	60,290	25,372	-2,376	N-acetylornithine aminotransferase; ArgD	nitrogen metabolism
alr4548	63,356	26,667	-2,376	photosystem II protein D2	photosynthesis
all0209	85,229	35,926	-2,372	unknown	unknown
alr3732	8,428	3,564	-2,364	protein serine-threonine kinase; PknE	regulatory functions
all4214	37,076	15,702	-2,361	50S ribosomal protein L4	ribosome component
all4357	55,584	23,558	-2,359	ATP-dependent Clp protease proteolytic subunit	degradation of proteins, peptides, and glycopeptides
alr7526	2,359	0,000	-2,359	transcriptional regulator	regulatory functions
asl5277	2,359	0,000	-2,359	HicA protein	resistance, adaptations to atypical conditions
asl3164	2,359	0,000	-2,359	pentapeptide repeat containing protein	unknown
asl2052	2,359	0,000	-2,359	unknown	unknown
all3521	264,520	112,389	-2,354	probable hydrogenase component	other enzymes
alr1700	104,398	44,390	-2,352	unknown	unknown
alr5065	29,721	12,642	-2,351	UDP-N-acetylmuramoylalanine--D-glutamate ligase	cell envelope
alr5208	67,492	28,726	-2,350	unknown	unknown
asr1102	66,464	28,317	-2,347	arsenical-resistance protein ACR3, efflux transporter	detoxification
all2425	26,910	11,504	-2,339	unknown	unknown
all2637	2,335	0,000	-2,335	unknown	unknown
alr4890	2,939	1,264	-2,326	peptidase U62, modulator of DNA gyrase	degradation of proteins, peptides, and glycopeptides
alr3331	1,776	0,764	-2,326	ATP-dependent helicase	DNA replication, recombination and repair
all0332	10,442	4,489	-2,326	polymorphic membrane protein	membrane component
all7377	2,001	0,860	-2,326	probable acid phosphatase	other enzymes
all4020	3,362	1,445	-2,326	transaldolase	pentose phosphate pathway
alr1883	2,150	0,924	-2,326	two-component hybrid sensor and regulator	regulatory functions
all1280	1,363	0,586	-2,326	two-component sensor histidine kinase	regulatory functions
alr0169	3,995	1,717	-2,326	cyclomaltodextrin glucanotransferase	sugars
all3633	3,701	1,591	-2,326	glucanase	sugars
all0143	6,536	2,810	-2,326	glycosyl transferase	sugars
all4830	6,760	2,906	-2,326	mannosyl transferase	sugars
alr0782	38,096	16,378	-2,326	ribulose-phosphate 3-epimerase	sugars

all1413	2,392	1,028	-2,326	MscS mechanosensitive ion channel	transport across membrane
all8545	3,778	1,624	-2,326	integrase-recombinase protein	transposon-related functions
alr1858	21,406	9,203	-2,326	transposase	transposon-related functions
all0796	4,281	1,841	-2,326	unknown	unknown
all1339	4,491	1,931	-2,326	unknown	unknown
all7227	1,061	0,456	-2,326	unknown	unknown
alr1546	7,511	3,229	-2,326	unknown	unknown
alr2980	11,892	5,113	-2,326	unknown	unknown
alr3411	26,898	11,564	-2,326	unknown	unknown
alr7075	18,614	8,002	-2,326	unknown	unknown
alr7532	1,362	0,586	-2,326	unknown	unknown
alr4957	73,829	31,787	-2,323	PilT domain-containing protein	chemotaxis and cell motility
alr1310	7,993	3,459	-2,311	glycoside hydrolase, family 57	sugars
alr3855	40,235	17,411	-2,311	unknown	unknown
all4116	19,874	8,615	-2,307	peptidase M15D, vanX D-ala-D-ala dipeptidase	degradation of proteins, peptides, and glycopeptides
alr0502	2,304	0,000	-2,304	unknown	unknown
alr4061	262,898	114,303	-2,300	unknown	unknown
all1844	17,364	7,551	-2,300	peptidase M50	degradation of proteins, peptides, and glycopeptides
alr2404	10,784	4,689	-2,300	unknown	unknown
all0328	118,148	51,390	-2,299	anthranilate synthetase alpha-subunit	anthranilate biosynthesis
all4315	37,587	16,389	-2,294	unknown	unknown
alr2359	40,351	17,594	-2,294	unknown	unknown
all0794	7,713	3,377	-2,284	radical SAM domain-containing protein	energy production and conversion
alr2051	3,445	1,509	-2,284	gamma-glutamyltranspeptidase	glutathione metabolism
all3116	8,543	3,741	-2,284	unknown	unknown
alr0947	7,615	3,334	-2,284	abortive infection protein	resistance, adaptations to atypical conditions
alr7612	9,730	4,261	-2,284	DNA-directed RNA polymerase subunit beta	RNA synthesis and modification
alr1960	189,007	82,826	-2,282	unknown	unknown
alr1414	11,786	5,185	-2,273	adenosylhomocysteinase	thioether formation
alr7210	2,272	0,000	-2,272	unknown	unknown
alr1216	86,403	38,039	-2,271	photosystem II 12 kD extrinsic protein PsbU	photosynthesis
alr7278	2,267	0,000	-2,267	unknown	unknown
all1787	28,068	12,388	-2,266	exopolysaccharide synthesis, ExoD	exopolysaccharide biosynthesis
all1760	13,769	6,077	-2,266	transcription termination factor; NusB	RNA synthesis and modification
all1931	2,264	0,000	-2,264	unknown	unknown
all0107	818,002	361,731	-2,261	photosystem I subunit XI; PsaL	photosynthesis
alr4368	6,248	2,770	-2,256	serine/threonine kinase; PknD	regulatory functions
all4931	20,149	8,959	-2,249	unknown	unknown
alr0504	2,245	0,000	-2,245	unknown	unknown
alr7077	2,245	0,000	-2,245	unknown	unknown

asl8512	2,245	0,000	-2,245	unknown	unknown
all2316	18,846	8,396	-2,245	aldo/keto reductase	other enzymes
alr2185	6,289	2,802	-2,245	ferrichrome-iron receptor	transport across membrane
alr1133	38,437	17,148	-2,241	unknown	unknown
alr2957	45,737	20,451	-2,236	putative carbohydrate kinase	sugars
alr0336	2,231	0,000	-2,231	unknown	unknown
all0575	2,224	0,000	-2,224	unknown	unknown
alr1244	4,491	2,023	-2,220	shikimate kinase	amino acids biosynthesis and metabolism
all0375	3,819	1,720	-2,220	holliday junction DNA helicase; RuvA	DNA replication, recombination and repair
alr3988	3,240	1,459	-2,220	exonuclease SbcC	DNA replication, recombination and repair
alr2514	2,492	1,122	-2,220	cytochrome c oxidase subunit II	electron transport chain
all4391	3,084	1,389	-2,220	enoyl-[acyl-carrier-protein] reductase	lipid metabolism
alr0766	5,066	2,282	-2,220	hydrogenase large subunit; HoxH	other enzymes
alr2280	3,874	1,745	-2,220	two-component response regulator	regulatory functions
all4205	39,870	17,957	-2,220	50S ribosomal protein L14	ribosome component
alr1383	1,500	0,675	-2,220	iron(III) ABC transporter, permease protein	transport across membrane
all3531	2,342	1,055	-2,220	unknown	unknown
all3786	4,684	2,110	-2,220	unknown	unknown
all4472	2,818	1,269	-2,220	unknown	unknown
all4479	4,984	2,245	-2,220	unknown	unknown
all7081	2,469	1,112	-2,220	unknown	unknown
alr1358	4,644	2,092	-2,220	Mg-protoporphyrin IX monomethyl ester (oxidative) cyclase	chlorophyll biosynthesis
all0266	1,870	0,842	-2,220	aminopeptidase P	degradation of proteins, peptides, and glycopeptides
alr7199	1,436	0,647	-2,220	plasmid recombinant protein	DNA replication, recombination and repair
alr0952	7,859	3,540	-2,220	cytochrome c oxidase subunit III	electron transport chain
all3305	8,223	3,703	-2,220	PatL	heterocyst differentiation
all1693	2,654	1,195	-2,220	glycerol-3-phosphate dehydrogenase	lipid metabolism
all2810	2,209	0,995	-2,220	glutathione dependent formaldehyde dehydrogenase	methane metabolism
all1058	2,399	1,081	-2,220	HAD-superfamily hydrolase subfamily IA, variant 3	other enzymes
all3891	5,726	2,579	-2,220	probable flavoprotein	photosynthesis
all3653	15,136	6,817	-2,220	allophycocyanin B alpha chain;ApcD	phycobilisomes and phycobiliproteins
all5008	2,289	1,031	-2,220	oxidoreductase	redox reactions
alr1067	11,196	5,043	-2,220	protein-tyrosine-phosphatase	regulatory functions
alr3268	1,557	0,701	-2,220	serine/threonine kinase	regulatory functions
all1639	2,349	1,058	-2,220	two-component hybrid sensor and regulator	regulatory functions
all7605	5,250	2,365	-2,220	two-component sensor histidine kinase	regulatory functions
alr3225	0,828	0,373	-2,220	two-component sensor histidine kinase	regulatory functions
alr2463	3,968	1,787	-2,220	aminoglycoside phosphotransferase	resistance, adaptations to atypical conditions

alr7552	0,901	0,406	-2,220	similar to ATP-dependent RNA helicase	RNA synthesis and modification
all4989	2,151	0,969	-2,220	glycoside hydrolase family 65, central catalytic	sugars
alr5240	3,193	1,438	-2,220	glycosyl transferase	sugars
all0041	5,124	2,308	-2,220	UDP-glucose 6-dehydrogenase	sugars
alr4741	2,940	1,324	-2,220	S-layer region-like protein	surface components
all4242	2,162	0,974	-2,220	potassium-dependent ATPase subunit D'	transport across membrane
all4130	5,413	2,438	-2,220	ABC transporter, binding protein component	transport across membrane
all3747	5,377	2,422	-2,220	TonB box-like	transport across membrane
alr2305	9,395	4,231	-2,220	MOSC domain-containing protein	unknown
all2893	6,077	2,737	-2,220	TPR repeat-containing protein	unknown
all0437	16,026	7,218	-2,220	unknown	unknown
all0969	1,502	0,677	-2,220	unknown	unknown
all1043	3,449	1,553	-2,220	unknown	unknown
all1626	0,920	0,415	-2,220	unknown	unknown
all2038	1,099	0,495	-2,220	unknown	unknown
all2804	5,014	2,258	-2,220	unknown	unknown
all4041	4,128	1,859	-2,220	unknown	unknown
alr0340	2,059	0,927	-2,220	unknown	unknown
alr0857	6,287	2,832	-2,220	unknown	unknown
alr1330	5,377	2,422	-2,220	unknown	unknown
alr1926	4,149	1,869	-2,220	unknown	unknown
alr2558	5,307	2,390	-2,220	unknown	unknown
alr2710	2,919	1,315	-2,220	unknown	unknown
alr3639	3,168	1,427	-2,220	unknown	unknown
alr4684	9,541	4,297	-2,220	unknown	unknown
alr5121	4,007	1,804	-2,220	unknown	unknown
alr7253	6,099	2,747	-2,220	unknown	unknown
alr7502	8,340	3,756	-2,220	unknown	unknown
alr7536	1,342	0,604	-2,220	unknown	unknown
alr7620	5,880	2,648	-2,220	unknown	unknown
asl0060	17,028	7,669	-2,220	unknown	unknown
asl2360	24,767	11,155	-2,220	unknown	unknown
asl8084	8,340	3,756	-2,220	unknown	unknown
asr2781	28,678	12,916	-2,220	unknown	unknown
alr4702	2,599	1,170	-2,220	unknown	unknown
alr2310	2,355	1,061	-2,220	similar to agmatinase	urea cycle and metabolism of amino groups
alr7082	2,220	0,000	-2,220	chromosome partitioning protein, ParA family ATPase	nucleoproteins
alr3185	34,713	15,753	-2,204	lipoate-protein ligase B	posttranslational modifications
asr4449	2,203	0,000	-2,203	unknown	unknown
alr0488	22,404	10,188	-2,199	pyrroline-5-carboxylate reductase	amino acids biosynthesis and metabolism
alr5055	29,617	13,468	-2,199	unknown	unknown

alr1954	37,800	17,214	-2,196	unknown	unknown
alr1107	2,193	0,000	-2,193	phosphoglycerate mutase	glycolisis
alr1815	2,193	0,000	-2,193	unknown	unknown
all4388	8,982	4,098	-2,191	polysaccharide export protein	exopolysaccharide biosynthesis
asr1592	45,275	20,659	-2,191	30S ribosomal protein S20	ribosome component
all3549	2,189	0,000	-2,189	similar to phycoerythrobilin lyase subunit (cpeF)	phycobilisomes and phycobiliproteins
alr3214	2,189	0,000	-2,189	unknown	unknown
all0232	17,236	7,888	-2,185	unknown	unknown
all4081	8,398	3,843	-2,185	unknown	unknown
alr4525	2,182	0,000	-2,182	unknown	unknown
asr3830	307,471	141,111	-2,179	unknown	unknown
alr2009	38,328	17,605	-2,177	chromosomal replication initiator protein DnaA	DNA replication, recombination and repair
all4418	8,052	3,702	-2,175	adenosine/AMP deaminase	nucleotide transport and metabolism
alr2800	2,226	1,023	-2,175	WD-repeat protein	regulatory functions
asr0742	44,480	20,451	-2,175	30S ribosomal protein S21	ribosome component
all2646	1,882	0,865	-2,175	polyketide synthase type I	siderophore/cyanotoxin biosynthesis
all5089	5,850	2,690	-2,175	phosphoglucomutase/phosphomannomutase	sugars
alr5291	5,040	2,317	-2,175	similar to alkaline phosphatase	unknown
all4838	8,705	4,022	-2,164	serine/threonine kinase	regulatory functions
all1813	2,162	0,000	-2,162	unknown	unknown
all3267	2,162	0,000	-2,162	unknown	unknown
all4632	2,162	0,000	-2,162	unknown	unknown
alr1422	2,162	0,000	-2,162	unknown	unknown
alr5053	13,124	6,085	-2,157	prolyl-tRNA synthetase	aminoacyl tRNA synthetases and tRNA modification
all0926	2,001	0,928	-2,157	two-component hybrid sensor and regulator	regulatory functions
all1173	10,502	4,869	-2,157	DNA-binding protein, starvation-inducible	resistance, adaptations to atypical conditions
alr1112	5,423	2,514	-2,157	probable transglycosylase	sugars
alr7014	2,776	1,287	-2,157	ABC transporter ATP-binding protein	transport across membrane
alr2768	15,447	7,162	-2,157	pentapeptide repeat-containing protein	unknown
all4829	15,691	7,275	-2,157	undecaprenyl-phosphate galactosephosphotransferase	cell envelope
alr1956	49,936	23,152	-2,157	phosphate starvation-inducible protein	resistance, adaptations to atypical conditions
alr2046	2,565	1,189	-2,157	putative potassium/proton antiporter	transport across membrane
all3278	7,271	3,371	-2,157	ErfK/YbiS/YcfS/YnhG family protein	unknown
alr2576	6,529	3,027	-2,157	unknown	unknown
alr7667	2,152	0,000	-2,152	unknown	unknown
all7072	5,906	2,747	-2,150	unknown	unknown
all2965	2,149	0,000	-2,149	unknown	unknown
alr1602	21,317	9,928	-2,147	6-phosphogluconolactonase	pentose phosphate pathway
alr4240	9,804	4,566	-2,147	RTX toxin transporter	protein and peptide secretion
alr4119	35,188	16,389	-2,147	unknown	unknown

all0404	2,142	0,000	-2,142	ATP-dependent Clp protease adaptor protein ClpS	degradation of proteins, peptides, and glycopeptides
all1033	2,142	0,000	-2,142	transcriptional regulator, XRE family	regulatory functions
all7223	2,142	0,000	-2,142	unknown	unknown
alr5335	2,142	0,000	-2,142	unknown	unknown
all4501	14,137	6,603	-2,141	phosphate regulon transcriptional regulator	regulatory functions
all4572	11,720	5,474	-2,141	phosphate ABC transporter, ATP-binding protein	transport across membrane
asl2403	340,751	159,642	-2,134	unknown	unknown
alr5284	12,706	5,955	-2,134	unknown	unknown
all1804	3,354	1,572	-2,134	two-component hybrid sensor and regulator	regulatory functions
alr4979	84,625	39,682	-2,133	unknown	unknown
all1944	2,131	0,000	-2,131	phenazine biosynthesis PhzC/PhzF protein	other enzymes
all4376	12,973	6,092	-2,130	probable glycosyl transferase	sugars
all1887	17,700	8,322	-2,127	unknown	unknown
all2580	2,123	0,000	-2,123	transcriptional regulator	regulatory functions
asr0183	2,123	0,000	-2,123	unknown	unknown
asr1373	2,123	0,000	-2,123	unknown	unknown
asr2978	2,123	0,000	-2,123	unknown	unknown
alr3537	28,777	13,557	-2,123	N-acetyl-glutamate semialdehyde dehydrogenase; HisH	urea cycle and metabolism of amino groups
all3794	19,742	9,336	-2,115	tryptophan synthase beta subunit	amino acids biosynthesis and metabolism
alr1952	4,756	2,249	-2,115	signal recognition particle protein	protein and peptide secretion
all1904	1,356	0,641	-2,115	adenylate cyclase; CyaB2	regulatory functions
all1389	1,438	0,680	-2,115	two-component hybrid sensor and regulator	regulatory functions
all1127	10,287	4,865	-2,115	NADH dehydrogenase	respiration, oxidative phosphorylation
alr0972	4,633	2,191	-2,115	ABC transporter ATP-binding protein	transport across membrane
all2050	2,138	1,011	-2,115	unknown	unknown
all4378	4,812	2,275	-2,115	unknown	unknown
all5018	8,523	4,030	-2,115	unknown	unknown
alr2207	4,126	1,951	-2,115	unknown	unknown
alr3925	1,182	0,559	-2,115	unknown	unknown
alr7636	3,841	1,816	-2,115	unknown	unknown
asl4122	21,622	10,225	-2,115	methionyl-tRNA formyltransferase	aminoacyl tRNA synthetases and tRNA modification
all4274	2,780	1,315	-2,115	probable 2-octaprenyl-6-methoxyphenol 4-monoxygenase; UbiH	coenzymes and cofactors
all3970	10,425	4,930	-2,115	endonuclease III	DNA degradation and modification
all1323	1,822	0,861	-2,115	DNA primase	DNA replication, recombination and repair
all1361	2,254	1,066	-2,115	similar to cytochrome P450	electron transport chain
all1876	3,604	1,704	-2,115	diacylglycerol kinase	lipid metabolism
all2928	67,362	31,856	-2,115	RNA-binding protein RbpB	nucleoproteins
all4947	2,398	1,134	-2,115	metallophosphoesterase	other enzymes

asl0108	93,409	44,174	-2,115	photosystem I subunit IX; PsaJ	photosynthesis
alr9001	1,953	0,923	-2,115	similar to mobilization protein	plasmid mobilization
all2772	2,834	1,340	-2,115	two-component sensor histidine kinase	regulatory functions
alr4877	3,899	1,844	-2,115	WD-repeat protein	regulatory functions
all5341	2,767	1,308	-2,115	probable glycosyl transferase	sugars
all1294	10,519	4,975	-2,115	similar to ABC transporter	transport across membrane
alr7329	8,223	3,889	-2,115	transposase	transposon-related functions
all1710	1,290	0,610	-2,115	unknown	unknown
all2318	4,406	2,084	-2,115	unknown	unknown
all3582	17,691	8,366	-2,115	unknown	unknown
all5096	2,672	1,264	-2,115	unknown	unknown
all5108	1,591	0,752	-2,115	unknown	unknown
alr3490	2,734	1,293	-2,115	unknown	unknown
alr7124	1,989	0,941	-2,115	unknown	unknown
all0251	2,108	0,000	-2,108	metallophosphoesterase	other enzymes
alr2269	10,220	4,855	-2,105	chloroplastic outer envelope membrane protein homolog	cell envelope
alr1311	26,777	12,761	-2,098	serine/threonine kinase	regulatory functions
alr2991	2,098	0,000	-2,098	DnaJ protein	heat shock proteins and chaperones
alr4693	2,098	0,000	-2,098	unknown	unknown
all4379	12,086	5,784	-2,090	peptide-chain-release factor 3	regulatory functions
alr3827	33,325	15,954	-2,089	unknown	unknown
alr1462	2,085	0,000	-2,085	fdxN element excision controlling factor protein XisI	heterocyst differentiation
all1843	2,085	0,000	-2,085	putative monovalent cation/H+ antiporter subunit C	transport across membrane
alr7531	2,085	0,000	-2,085	unknown	unknown
asl2397	2,085	0,000	-2,085	unknown	unknown
asl7181	2,085	0,000	-2,085	unknown	unknown
asr3694	2,085	0,000	-2,085	unknown	unknown
all4578	66,145	31,734	-2,084	unknown	unknown
alr4531	2,154	1,033	-2,084	unknown	unknown
all1359	15,215	7,300	-2,084	tRNA (5-methylaminomethyl-2-thiouridylate)-methyltransferase	aminoacyl tRNA synthetases and tRNA modification
alr1903	1,735	0,832	-2,084	unknown	unknown
alr2864	2,080	0,000	-2,080	probable glycosyl transferase	sugars
all7662	2,073	0,000	-2,073	high light inducible protein	electron transport chain
alr5156	2,073	0,000	-2,073	unknown	unknown
alr1742	36,096	17,419	-2,072	DnaK-type molecular chaperone; DnaK	heat shock proteins and chaperones
all2875	7,129	3,442	-2,071	two-component hybrid sensor and regulator	regulatory functions
all1970	2,070	0,000	-2,070	unknown	unknown
alr3758	2,067	0,000	-2,067	probable anti-sigma factor antagonist	regulatory functions

all0004	24,017	11,649	-2,062	ATP synthase subunit gamma; AtpC	energy production and conversion
all0852	7,477	3,627	-2,062	cyclase/dehydrase	other enzymes
all3552	11,019	5,345	-2,062	exopolyphosphatase	purine/pyrimidine biosynthesis and metabolism
alr1575	1,448	0,703	-2,062	possible Sensor with Chase2 domain	regulatory functions
all7606	6,170	2,993	-2,062	two-component response regulator	regulatory functions
all1767	9,035	4,382	-2,062	probable glycosyl transferase	sugars
alr3757	2,187	1,061	-2,062	cellulose synthase catalytic subunit	cell envelope
alr0939	3,388	1,643	-2,062	biotin carboxylase; AccC	lipid metabolism
all1205	4,912	2,383	-2,062	alpha/beta hydrolase fold protein	other enzymes
all3862	11,477	5,567	-2,062	GTP-binding protein	regulatory functions
alr4586	4,406	2,137	-2,062	two-component sensor histidine kinase	regulatory functions
all1126	3,321	1,611	-2,062	NADH dehydrogenase	respiration, oxidative phosphorylation
alr1556	4,069	1,974	-2,062	ABC transporter, permease protein	transport across membrane
alr1620	5,601	2,717	-2,062	ABC transporter, permease protein	transport across membrane
all2778	4,464	2,165	-2,062	unknown	unknown
alr1197	5,861	2,843	-2,062	unknown	unknown
asr1328	58,380	28,317	-2,062	unknown	unknown
alr1690	731,061	354,689	-2,061	cell wall-binding protein	cell envelope
all2898	2,060	0,000	-2,060	two-component system response regulator	regulatory functions
alr3727	26,198	12,746	-2,055	photosystem II protein D1	photosynthesis
alr1229	3,670	1,788	-2,052	two-component sensor histidine kinase	regulatory functions
alr4683	146,214	71,301	-2,051	RNA-binding protein RbpD	nucleoproteins
alr3228	39,708	19,374	-2,050	unknown	unknown
asl4409	2,048	0,000	-2,048	NB-ARC	resistance, adaptations to atypical conditions
asl1098	2,048	0,000	-2,048	unknown	unknown
asl1446	2,048	0,000	-2,048	unknown	unknown
all0129	27,307	13,359	-2,044	two-component response regulator; RpaA	regulatory functions
all3541	6,213	3,039	-2,044	unknown	unknown
alr7073	2,039	0,000	-2,039	HAD family hydrolase	other enzymes
all1557	2,034	0,000	-2,034	unknown	unknown
all0493	10,265	5,057	-2,030	lipopolysaccharide biosynthesis protein	LPS biosynthesis
all4296	3,707	1,826	-2,030	AAA ATPase	posttranslational modifications
alr2336	4,314	2,125	-2,030	phosphate permease	transport across membrane
alr5084	8,939	4,403	-2,030	endopeptidase Clp ATP-binding chain B	degradation of proteins, peptides, and glycopeptides
all0492	8,230	4,054	-2,030	acyl-phosphate glycerol-3-phosphate acyltransferase	lipid metabolism
alr5293	3,958	1,950	-2,030	secretion protein HlyD	protein and peptide secretion
asl2551	21,229	10,458	-2,030	transcriptional regulator	regulatory functions
alr3593	5,070	2,497	-2,030	polyphosphate kinase	respiration, oxidative phosphorylation
alr7312	4,865	2,397	-2,030	unknown	unknown
alr2938	18,589	9,157	-2,030	iron superoxide dismutase; SodB	detoxification

alr0397	22,733	11,239	-2,023	similar to ferric aerobactin receptor	transport across membrane
all4078	14,163	7,009	-2,021	unknown	unknown
all3688	2,019	0,000	-2,019	FAD dependent oxidoreductase	amino acids biosynthesis and metabolism
all1321	2,013	0,000	-2,013	GCN5-related N-acetyltransferase	other enzymes
all4040	2,013	0,000	-2,013	KWG Leptospira repeat protein	unknown
alr3775	2,013	0,000	-2,013	unknown	unknown
alr0791	2,232	1,111	-2,009	outer membrane secretion protein	cell envelope
alr1054	4,919	2,449	-2,009	DNA polymerase III alpha subunit	DNA replication, recombination and repair
all4463	5,359	2,667	-2,009	exonuclease SbcD	DNA replication, recombination and repair
all2115	7,624	3,795	-2,009	unknown	unknown
all4968	4,823	2,401	-2,009	glutathione reductase	detoxification
alr5066	6,682	3,326	-2,009	UDP-N-acetylenolpyruvylglucosamine reductase	peptidoglycan biosynthesis
all3557	1,229	0,612	-2,009	serine/threonine kinase with two-component sensor domain	regulatory functions
all5056	19,291	9,603	-2,009	transcriptional regulator	regulatory functions
all2281	2,004	0,000	-2,004	two-component response regulator	regulatory functions
alr3707	8,543	17,209	2,014	phycocyanobilin:ferredoxin oxidoreductase	phytochromobilin biosynthesis
all2549	2,447	4,960	2,027	putative glycosyl transferase, group 1	sugars
all2842	0,000	2,034	2,034	unknown	unknown
all3032	5,897	12,085	2,049	unknown	unknown
all1772	0,778	1,594	2,049	unknown	unknown
alr3504	4,928	10,159	2,061	putative diguanylate cyclase	regulatory functions
asl3609	0,000	2,068	2,068	unknown	unknown
all7167	0,000	2,068	2,068	unknown	unknown
alr3670	3,283	6,793	2,069	urease alpha subunit	nitrogen metabolism
asl0994	0,000	2,092	2,092	unknown	unknown
alr2092	0,989	2,080	2,102	histidinol phosphate aminotransferase; HisC	amino acids biosynthesis and metabolism
alr4692	2,245	4,719	2,102	transcriptional regulator	regulatory functions
all1355	2,085	4,382	2,102	unknown	unknown
all3643	2,085	4,382	2,102	unknown	unknown
alr5152	2,169	4,559	2,102	ribonuclease Z	aminoacyl tRNA synthetases and tRNA modification
all3075	1,769	3,718	2,102	Maf-like protein	cell division
all2391	0,924	1,943	2,102	putative corrinoid adenosyltransferase BtuR/CobO/CobP	cobalamin, heme, phycobilin and porphyrin
alr5164	1,743	3,663	2,102	serine protease	degradation of proteins, peptides, and glycopeptides
alr3716	1,092	2,296	2,102	exonuclease ABC subunit A	DNA replication, recombination and repair
alr0079	0,850	1,787	2,102	esterase	lipid metabolism
alr4059	5,516	11,594	2,102	phosphopantetheine-binding protein	lipid metabolism
alr2968	2,787	5,859	2,102	homocitrate synthase; NifV2	nitrogen fixation
alr1713	3,020	6,347	2,102	Mo-dependent nitrogenase-like	nitrogen fixation

all3572	0,970	2,039	2,102	photosystem II protein D1	photosynthesis
alr0430	1,212	2,548	2,102	probable short-chain dehydrogenase	redox reactions
alr3009	1,317	2,768	2,102	CBS domain-containing protein	redox reactions
all2821	0,765	1,607	2,102	two-component response regulator	regulatory functions
all0217	1,460	3,068	2,102	patogenesis related protein	resistance, adaptations to atypical conditions
all0745	1,191	2,504	2,102	ribosomal biogenesis GTPase	ribosome biogenesis
asl4211	3,766	7,916	2,102	30S ribosomal protein S19	ribosome component
alr2890	1,347	2,832	2,102	RNA-binding S4 domain-containing protein	translation
all7196	0,569	1,195	2,102	ABC transporter ATP-binding protein	transport across membrane
all4732	0,580	1,219	2,102	ABC transporter, ATP-binding protein	transport across membrane
all2610	0,408	0,858	2,102	ferrichrome-iron receptor	transport across membrane
all3624	2,351	4,941	2,102	transposase	transposon-related functions
all3114	1,214	2,552	2,102	pentapeptide repeat-containing protein	unknown
asl4014	7,076	14,873	2,102	unknown	unknown
asr0098	6,369	13,386	2,102	unknown	unknown
asr0105	3,936	8,272	2,102	unknown	unknown
alr1536	2,895	6,085	2,102	unknown	unknown
alr1622	1,751	3,681	2,102	unknown	unknown
alr3365	1,052	2,211	2,102	unknown	unknown
alr2888	2,780	5,843	2,102	unknown	unknown
alr8543	1,607	3,377	2,102	unknown	unknown
all4679	1,523	3,201	2,102	unknown	unknown
alr0586	1,055	2,218	2,102	unknown	unknown
alr3357	0,865	1,818	2,102	unknown	unknown
alr0132	0,787	1,654	2,102	unknown	unknown
asr1335	6,255	13,147	2,102	unknown	unknown
alr1296	2,520	5,297	2,102	unknown	unknown
alr2967	2,520	5,297	2,102	unknown	unknown
all2704	1,126	2,367	2,102	unknown	unknown
all0911	0,000	2,116	2,116	ABC phosphate transport system phosphate-binding periplasmic protein	transport across membrane
asl2490	0,000	2,116	2,116	unknown	unknown
all3320	3,958	8,423	2,128	unknown	unknown
all2006	6,097	12,975	2,128	unknown	unknown
all0218	8,271	17,639	2,133	pyridoxal phosphate biosynthetic protein PdxJ	vitamin biosynthesis
asl0491	0,000	2,140	2,140	unknown	unknown
asl1938	0,000	2,140	2,140	unknown	unknown
alr5272	1,708	3,671	2,150	two-component sensor histidine kinase	regulatory functions
all4985	1,592	3,421	2,150	sucrose synthase	sugars
alr2680	0,510	1,096	2,150	polyketide synthase	siderophore/cyanotoxin biosynthesis
alr7015	0,000	2,153	2,153	aminoglycoside N6'-acetyltransferase	resistance, adaptations to atypical conditions
asr5146	0,000	2,165	2,165	unknown	unknown

alr4309	6,066	13,147	2,167	unknown	unknown
alr0181	1,958	4,245	2,167	unknown	unknown
all1070	0,000	2,204	2,204	purine-binding chemotaxis protein cheW	chemotaxis and cell motility
alr0877	5,724	12,631	2,207	acylphosphatase	energy production and conversion
all0496	2,402	5,302	2,207	unknown	unknown
all1707	4,543	10,026	2,207	unknown	unknown
alr4806	5,456	12,041	2,207	serine hydroxymethyltransferase	cellular one-carbon cycle
alr4269	3,444	7,601	2,207	radical SAM domain-containing protein	other enzymes
alr0356	4,068	8,978	2,207	two-component response regulator	regulatory functions
all0579	7,631	16,842	2,207	50S ribosomal protein L9	ribosome component
all3575	2,195	4,844	2,207	ABC transporter, ATP-binding protein	transport across membrane
all7111	2,354	5,195	2,207	transposase	transposon-related functions
all0476	4,246	9,370	2,207	unknown	unknown
alr4468	0,000	2,245	2,245	probable phosphinothricin N-acetyltransferase	other enzymes
asl0629	0,000	2,245	2,245	HNH endonuclease	resistance, adaptations to atypical conditions
asr0179	0,000	2,245	2,245	unknown	unknown
asl2194	0,000	2,245	2,245	unknown	unknown
alr8560	1,360	3,063	2,252	reverse transcriptase homolog	transposon-related functions
alr2073	1,974	4,446	2,252	glutamate N-acetyltransferase	urea cycle and metabolism of amino groups
alr0203	0,000	2,258	2,258	unknown	unknown
all8085	20,041	45,328	2,262	unknown	unknown
asr1005	0,000	2,272	2,272	unknown	unknown
asr7548	0,000	2,272	2,272	unknown	unknown
alr1552	1,907	4,343	2,277	ABC transporter, ATP-binding protein	transport across membrane
alr1143	5,417	12,334	2,277	unknown	unknown
alr8073	0,000	2,291	2,291	unknown	unknown
all4609	3,407	7,811	2,293	glycine cleavage system protein T	salvage pathways
asl7281	0,000	2,301	2,301	unknown	unknown
asl2805	0,000	2,330	2,330	unknown	unknown
all7091	0,000	2,345	2,345	unknown	unknown
asl2362	0,000	2,360	2,360	lipoprotein; NlpD	lipid metabolism
asl2540	0,000	2,360	2,360	unknown	unknown
alr5190	0,000	2,360	2,360	unknown	unknown
alr4456	0,871	2,060	2,365	tropinone reductase homolog	alkaloid biosynthesis
alr1965	1,086	2,568	2,365	ATP phosphoribosyltransferase	amino acids biosynthesis and metabolism
all4895	0,987	2,335	2,365	carotenoid oxygenase	carotenoids
alr3857	2,484	5,874	2,365	cell division protein FtsQ	cell division
alr5348	0,521	1,233	2,365	putative ParB family protein	cell division
all2544	1,128	2,667	2,365	precorrin isomerase, CbiC/CobH	cobalamin, heme, phycobilin and porphyrin
all0177	0,409	0,967	2,365	heterocyst-specific flavodiiron protein, Flv1	detoxification
alr1576	0,753	1,781	2,365	dehydrogenase subunit	other enzymes
all4558	0,642	1,517	2,365	Rieske (2Fe-2S) domain-containing protein	redox reactions

alr2505	1,201	2,839	2,365	aminotransferase, class V (cystein desulfurase)	thiamine metabolism
alr3947	0,761	1,799	2,365	cobalt transport protein CbiM	transport across membrane
all7563	1,047	2,476	2,365	transposase	transposon-related functions
all8069	1,150	2,720	2,365	transposase	transposon-related functions
all7173	3,258	7,705	2,365	unknown	unknown
alr7239	1,507	3,562	2,365	unknown	unknown
all3461	1,899	4,489	2,365	unknown	unknown
all4460	1,262	2,985	2,365	unknown	unknown
all0254	1,007	2,380	2,365	unknown	unknown
alr0841	0,567	1,342	2,365	unknown	unknown
alr3090	3,033	7,171	2,365	Mn-catalase	detoxification
all4708	1,102	2,605	2,365	thymidylate kinase	DNA replication, recombination and repair
alr0241	2,193	5,185	2,365	1-acyl-sn-glycerol-3-phosphate acyltransferase	lipid metabolism
alr0038	0,654	1,547	2,365	dTDP-glucose 4-6-dehydratase; RfbB	LPS biosynthesis
asr4517	14,153	33,465	2,365	phycobilisome degradation protein; NblA	phycobilisome and phycobiliproteins
all0792	1,578	3,731	2,365	Streptomyces cyclase/dehydrase	polyketide biosynthesis
alr2242	0,299	0,706	2,365	putative signal transduction protein	regulatory functions
all8086	1,552	3,669	2,365	transcriptional regulator	regulatory functions
alr4992	1,273	3,009	2,365	transcriptional regulator, TetR family	regulatory functions
all0825	0,341	0,807	2,365	two-component sensor histidine kinase	regulatory functions
alr1010	1,839	4,348	2,365	calcium-binding protein, CcbP	regulatory functions
all2918	0,989	2,340	2,365	putative secreted nuclease	resistance, adaptations to atypical conditions
all2964	0,527	1,246	2,365	probable NADH dehydrogenase	respiration, oxidative phosphorylation
all1702	1,633	3,861	2,365	anti-sigma B factor	RNA synthesis and modification
all2037	0,636	1,505	2,365	glycosyltransferase group 1	sugars
all2011	1,487	3,517	2,365	ABC transporter, permease protein	transport across membrane
asl4181	5,988	14,158	2,365	preprotein translocation subunit SecG	transport across membrane
all0119	10,153	24,007	2,365	unknown	unknown
all4767	4,272	10,101	2,365	unknown	unknown
alr5135	2,312	5,467	2,365	unknown	unknown
all1292	1,633	3,861	2,365	unknown	unknown
all0037	1,567	3,706	2,365	unknown	unknown
alr3548	1,536	3,633	2,365	unknown	unknown
alr3828	1,516	3,586	2,365	unknown	unknown
alr4504	1,424	3,367	2,365	unknown	unknown
alr1449	1,210	2,861	2,365	unknown	unknown
all7616	1,020	2,411	2,365	unknown	unknown
all1467	0,942	2,226	2,365	unknown	unknown
alr3215	0,934	2,209	2,365	unknown	unknown
alr1959	0,891	2,108	2,365	unknown	unknown
all2617	0,701	1,658	2,365	unknown	unknown
alr1612	3,512	8,303	2,365	unknown	unknown

all7598	2,715	6,421	2,365	unknown	unknown
alr2462	2,182	5,160	2,365	unknown	unknown
alr2071	1,469	3,473	2,365	unknown	unknown
asl1502	0,000	2,390	2,390	unknown	unknown
asr3389	0,000	2,422	2,422	unknown	unknown
all0936	2,985	7,320	2,452	c-type cytochrome synthesis protein	cytochrome biogenesis
asr3621	0,000	2,454	2,454	unknown	unknown
alr8528	0,000	2,465	2,465	unknown	unknown
all3650	4,324	10,712	2,477	unknown	unknown
asr0081	0,000	2,521	2,521	unknown	unknown
all2128	5,166	13,031	2,522	unknown	unknown
alr4658	4,261	10,748	2,522	unknown	unknown
alr0806	3,695	9,319	2,522	unknown	unknown
alr1822	0,000	2,539	2,539	unknown	unknown
alr4934	3,163	8,160	2,579	N-acyl-L-amino acid amidohydrolase	lipid metabolism
alr7354	0,000	2,592	2,592	glutathione S-transferase	detoxification
alr8074	0,000	2,605	2,605	unknown	unknown
all8048	9,433	24,691	2,617	unknown	unknown
all4845	3,175	8,341	2,627	phenylalanyl-tRNA synthetase alpha chain	aminoacil-tRNA synthesis and tRNA modification
all3587	0,736	1,933	2,627	two-component sensor histidine kinase	regulatory functions
asr1134	5,742	15,087	2,627	putative CsbD-like protein	resistance, adaptations to atypical conditions
alr2074	2,557	6,717	2,627	possible glyoxalase/bleomycin resistance protein/dioxygenase	resistance, adaptations to atypical conditions
alr4082	1,660	4,362	2,627	transposase	transposon-related functions
all0958	0,736	1,933	2,627	pentapeptide repeat containing protein	unknown
alr1821	1,806	4,744	2,627	unknown	unknown
all7633	1,441	3,787	2,627	unknown	unknown
alr1206	1,893	4,975	2,627	unknown	unknown
asl0095	0,000	2,629	2,629	unknown	unknown
alr5251	4,803	12,814	2,668	two-component response regulator	regulatory functions
all4198	7,836	21,000	2,680	50S ribosomal protein L15	ribosome component
asr2565	0,000	2,707	2,707	putative beta-ketoacyl synthase	lipid metabolism
alr2261	0,000	2,720	2,720	unknown	unknown
asl7258	0,000	2,747	2,747	unknown	unknown
alr1709	1,557	4,295	2,759	alpha/beta hydrolase fold protein	other enzymes
all1800	2,445	6,746	2,759	TrkA domain-containing protein	transport across membrane
all4462	0,965	2,662	2,759	similar to light-harvesting 1 (B870) complex assembly protein PucC	bacteriochlorophyll
all5088	2,371	6,540	2,759	putative deoxyrribonucleotide triphosphate pyrorophosphatase	nucleotide transport and metabolism
all3570	2,747	7,579	2,759	inorganic pyrophosphatase; Ppa	phosphorilation
all3310	0,668	1,843	2,759	TonB-dependent receptor	transport across membrane

all2724	2,552	7,040	2,759	unknown	unknown
all8503	0,000	2,832	2,832	unknown	unknown
all1886	2,203	6,251	2,837	unknown	unknown
alr2125	5,242	15,025	2,866	unknown	unknown
asr7257	0,000	2,876	2,876	unknown	unknown
all1164	42,291	121,739	2,879	unknown	unknown
all1456	2,327	6,726	2,890	nitrogen fixation protein NifU	nitrogen fixation
alr1982	0,000	2,894	2,894	glucokinase	glycolisis
all8062	2,856	8,323	2,914	unknown	unknown
alr8006	3,256	9,533	2,928	chromosome partitioning protein, ParA family ATPase	nucleoproteins
alr1722	0,000	2,950	2,950	probable oxidoreductase	redox reactions
all3181	2,892	8,548	2,956	unknown	unknown
asr8042	0,000	2,969	2,969	putative GTP binding protein EngA	other enzymes
all0311	0,000	2,969	2,969	unknown	unknown
all2126	0,000	2,969	2,969	unknown	unknown
all2919	0,000	2,993	2,993	ferredoxin	electron transport chain
all8080	6,749	20,214	2,995	unknown	unknown
alr3512	0,000	3,017	3,017	dihydroneopterin aldolase	vitamin biosynthesis
all3161	2,520	7,680	3,048	cadmium-transporting ATPase	transport across membrane
all2621	0,000	3,137	3,137	transcriptional regulator	regulatory functions
alr3577	0,000	3,146	3,146	unknown	unknown
alr1313	1,608	5,070	3,153	3-isopropylmalate dehydrogenase; LeuB	amino acids biosynthesis and metabolism
all4012	2,538	8,002	3,153	chorismate mutase	amino acids biosynthesis and metabolism
all7099	1,134	3,574	3,153	HNH endonuclease	DNA degradation and modification
all7179	1,105	3,484	3,153	SigG	RNA synthesis and modification
all2638	0,283	0,893	3,153	glutamate-1-semialdehyde aminotransferase	tetrapyrrole biosynthesis pathway
alr3273	0,316	0,998	3,153	twin-arginine translocation pathway signal	transport across membrane
all2767	1,134	3,574	3,153	unknown	unknown
alr4015	0,885	2,789	3,153	unknown	unknown
alr7587	0,257	0,811	3,153	unknown	unknown
alr1018	0,502	1,583	3,153	Glu-tRNA(Gln) amidotransferase subunit A	aminoacyl-tRNA synthetases and tRNA modification
all2097	0,234	0,736	3,153	cell death suppressor protein	chlorophyll catabolism, developmentally programmed death
all0455	0,497	1,566	3,153	cobalamin biosynthesis precorrin-2 methyltransferase	cobalamin, heme, phycobilin and porphyrin
alr4932	0,534	1,683	3,153	DNA polymerase III gamma and tau subunits	DNA replication, recombination and repair
alr2075	0,813	2,562	3,153	recombinase	DNA replication, recombination and repair
all1371	0,487	1,534	3,153	polyphosphate glucokinase	glycolisis
all4871	0,405	1,278	3,153	putative phospholipid/glycerol acyltransferase	lipid metabolism
asr1309	4,549	14,342	3,153	NifU like protein	nitrogen fixation

all1432	0,923	2,910	3,153	putative molybdenum cofactor biosynthesis	nitrogen fixation
all0250	2,115	6,669	3,153	metallophosphoesterase	other enzymes
all4946	0,463	1,461	3,153	posible methyltransferase type 11	other enzymes
alr7101	0,340	1,073	3,153	WD-repeat protein	other enzymes
all2753	0,426	1,343	3,153	possible alpha/beta hydrolase fold protein	other enzymes
alr4993	1,526	4,812	3,153	posssible NUDIX hydrolase	other enzymes
all2221	0,233	0,735	3,153	phosphonate metabolism protein PhnM	phosphonate metabolism
all3776	1,507	4,750	3,153	FHA domain protein	regulatory functions
alr0344	0,293	0,924	3,153	serine/threonine kinase	regulatory functions
asl0125	2,407	7,590	3,153	transcriptional regulator	regulatory functions
all4726	0,314	0,990	3,153	two-component sensor histidine kinase	regulatory functions
asl3726	1,946	6,135	3,153	CAB/ELIP/HLIP superfamily	resistance, adaptations to atypical conditions
all4092	1,007	3,173	3,153	heat shock protein DnaJ-like	resistance, adaptations to atypical conditions
asl0779	1,717	5,413	3,153	proteinase inhibitor I4, serpin	resistance, adaptations to atypical conditions
alr3407	1,156	3,645	3,153	similar to abortive phage resistance protein	resistance, adaptations to atypical conditions
rrn5Sc	2,944	9,280	3,153	5S rRNA	ribosome component
alr1117	1,424	4,489	3,153	glycosyl transferase group 1	sugars
alr3424	2,091	6,593	3,153	GDP-mannose 4,6-dehydratase	sugars
all3396	1,091	3,440	3,153	sulfur-regulated protein SrpB	sulfur-responsive protein
alr1312	1,796	5,663	3,153	tRNA-i(6) A37 modification enzyme MiaB	translation
alr3944	1,156	3,645	3,153	cobalt transport protein CbiN	transport across membrane
all2113	0,225	0,711	3,153	Na+/H+ antiporter	transport across membrane
alr7093	1,112	3,506	3,153	periplasmic divalent cation tolerance protein	transport across membrane
all8089	0,373	1,176	3,153	phosphonate ABC transporter, phosphate-binding protein	transport across membrane
all3151	1,150	3,627	3,153	potassium-transporting ATPase C chain	transport across membrane
all3534	0,407	1,283	3,153	probable potassium efflux transporter	transport across membrane
alr1633	0,741	2,335	3,153	sulfate permease	transport across membrane
alr7386	1,145	3,609	3,153	transposase	transposon-related functions
all2066	1,366	4,305	3,153	transposase	transposon-related functions
all7244	1,366	4,305	3,153	transposase	transposon-related functions
all7115	0,856	2,700	3,153	transposase	transposon-related functions
asr3686	2,994	9,439	3,153	unknown	unknown
all0313	2,104	6,633	3,153	unknown	unknown
alr2872	2,104	6,633	3,153	unknown	unknown
asr4321	1,645	5,185	3,153	unknown	unknown
all4144	1,629	5,136	3,153	unknown	unknown
asr2668	1,622	5,113	3,153	unknown	unknown
all2441	1,262	3,980	3,153	unknown	unknown
asr0461	1,204	3,795	3,153	unknown	unknown
alr2527	1,145	3,609	3,153	unknown	unknown
alr7264	1,052	3,316	3,153	unknown	unknown

all0337	0,957	3,017	3,153	unknown	unknown
alr1917	0,859	2,707	3,153	unknown	unknown
alr1356	0,817	2,574	3,153	unknown	unknown
all3318	0,778	2,454	3,153	unknown	unknown
alr2345	0,628	1,979	3,153	unknown	unknown
all0350	0,624	1,969	3,153	unknown	unknown
alr0194	0,602	1,897	3,153	unknown	unknown
all8538	0,602	1,897	3,153	unknown	unknown
alr5205	0,501	1,580	3,153	unknown	unknown
alr1233	0,422	1,329	3,153	unknown	unknown
alr3472	0,418	1,319	3,153	unknown	unknown
all5213	0,396	1,248	3,153	unknown	unknown
all8554	0,348	1,096	3,153	unknown	unknown
all1477	0,268	0,846	3,153	unknown	unknown
all5114	0,254	0,801	3,153	unknown	unknown
all2793	0,171	0,540	3,153	unknown	unknown
alr3588	0,128	0,405	3,153	unknown	unknown
asl0716	2,162	6,817	3,153	unknown	unknown
all0025	1,589	5,008	3,153	unknown	unknown
all3581	1,424	4,489	3,153	unknown	unknown
alr2537	1,162	3,663	3,153	unknown	unknown
all3616	1,112	3,506	3,153	unknown	unknown
all7193	0,865	2,727	3,153	unknown	unknown
all1123	0,712	2,245	3,153	unknown	unknown
alr0761	0,703	2,218	3,153	unknown	unknown
all1969	0,577	1,818	3,153	unknown	unknown
alr7216	0,446	1,405	3,153	unknown	unknown
all3163	0,336	1,058	3,153	unknown	unknown
alr7543	0,247	0,778	3,153	unknown	unknown
alr5007	0,518	1,632	3,153	similar to cell death suppressor protein Lls1	chlorophyll catabolism, developmentally programmed death
alr3224	0,481	1,518	3,153	site-specific recombinase (xisC)-like	heterocyst differentiation
all5179	0,528	1,666	3,153	O-methyltransferase	other enzymes
alr3742	0,323	1,020	3,153	photosystem II protein D1	photosynthesis
all5288	0,536	1,689	3,153	N-(5'-phosphoribosyl)anthranilate isomerase	purine/pyrimidine biosynthesis and metabolism
alr4551	2,399	7,564	3,153	transcriptional regulator	regulatory functions
alr0820	0,519	1,636	3,153	sun protein	RNA synthesis and modification
alr2679	0,330	1,039	3,153	polyketide synthase	siderophore/cyanotoxin biosynthesis
all4026	0,137	0,431	3,153	TonB-dependent receptor	transport across membrane
all0917	0,414	1,305	3,153	ABC transporter permease protein	transport across membrane
all0371	0,276	0,870	3,153	probable integral membrane efflux protein	transport across membrane
all1837	1,057	3,331	3,153	putative monovalent cation/H+ antiporter subunit	transport across membrane

				B	
alr0190	0,361	1,140	3,153	ABC transporter, ATP-binding protein	transport across membrane
all4303	1,637	5,160	3,153	pentapeptide repeat-containing protein	unknown
alr8005	1,637	5,160	3,153	unknown	unknown
alr2496	1,469	4,630	3,153	unknown	unknown
alr4810	1,102	3,473	3,153	unknown	unknown
alr0519	1,071	3,377	3,153	unknown	unknown
alr4134	0,942	2,969	3,153	unknown	unknown
alr1125	0,919	2,899	3,153	unknown	unknown
alr0892	0,871	2,747	3,153	unknown	unknown
alr7088	0,784	2,471	3,153	unknown	unknown
alr1215	0,548	1,728	3,153	unknown	unknown
alr4151	0,528	1,666	3,153	unknown	unknown
alr3434	0,388	1,223	3,153	unknown	unknown
alr1329	0,248	0,782	3,153	unknown	unknown
asr7385	0,000	3,173	3,173	transposase	transposon-related functions
alr0553	0,000	3,229	3,229	transposase	transposon-related functions
alr3508	0,000	3,258	3,258	unknown	unknown
asr1907	0,000	3,287	3,287	unknown	unknown
all5185	1,832	6,135	3,350	mercuric reductase	detoxification
alr4268	2,492	8,417	3,378	putative dihydroflavonol 4-reductase	anthocyanin biosynthesis
alr1514	0,000	3,408	3,408	unknown	unknown
asr2605	0,000	3,408	3,408	unknown	unknown
asr8053	0,000	3,408	3,408	unknown	unknown
asr3089	7,699	26,294	3,415	transglycosylase-associated protein	membrane component
alr0054	1,327	4,601	3,468	peptidase S13, D-Ala-D-Ala carboxipeptidase C	cell wall/membrane biogenesis
asr3605	0,000	3,473	3,473	trigger factor	ribosome associated molecular chaperone, protein export
all8043	16,471	58,275	3,538	unknown	unknown
asl0294	0,000	3,540	3,540	unknown	unknown
alr2948	1,390	4,930	3,547	zinc-containing alcohol deshydrogenase	energy production and conversion
alr4803	3,620	12,841	3,547	2-hydroxyhepta-2,4-diene-1,7-dioate isomerase	amino acids biosynthesis and metabolism
alr8527	0,000	3,645	3,645	unknown	unknown
alr2927	2,634	9,687	3,678	peptidase	degradation of proteins, peptides, and glycopeptides
all4286	0,900	3,312	3,678	3-oxoacyl-[acyl-carrier-protein] synthase	lipid metabolism
alr2945	0,699	2,572	3,678	probable orotate phosphoribosyltransferase	purine/pyrimidine biosynthesis and metabolism
asl0319	3,936	14,476	3,678	unknown	unknown
alr1654	1,068	3,928	3,678	unknown	unknown
asl1962	0,000	3,718	3,718	unknown	unknown
all2689	0,000	3,782	3,782	type I restriction enzyme, modification chain	DNA replication, recombination and repair
all0050	1,687	6,383	3,783	unknown	unknown

asl0260	15,718	60,173	3,828	unknown	unknown
all1995	1,052	4,145	3,941	phosphoribosyltransferase	other enzymes
alr4582	1,350	5,320	3,941	adenine phosphoribosyltransferase	salvage pathways
all1647	0,236	0,931	3,941	peptide synthetase	siderophore/cyanotoxin biosynthesis
all5184	1,000	3,941	3,941	Mg2+ transport protein	transport across membrane
asr4707	2,883	11,362	3,941	unknown	unknown
all3585	0,837	3,299	3,941	unknown	unknown
all5137	0,647	2,549	3,941	probable peptidase C14, caspase catalytic subunit P20	degradation of proteins, peptides, and glycopeptides
alr0799	2,162	8,521	3,941	glutaredoxin-related protein	detoxification
all1032	1,599	6,303	3,941	putative acetyltransferase	other enzymes
alr3356	1,297	5,113	3,941	similar to phytochrome	regulatory functions
alr3423	4,734	18,654	3,941	similar to anti-sigma-B factor	RNA synthesis and modification
alr8015	0,288	1,133	3,941	probable ATPase	transport across membrane
alr0992	0,496	1,954	3,941	ammonium transporter	transport across membrane
alr5330	0,871	3,434	3,941	unknown	unknown
all1902	1,390	5,478	3,941	unknown	unknown
alr8034	6,694	26,750	3,996	similar to mobilization protein TraI	plasmid mobilization
asl4994	0,000	4,045	4,045	similar to circadian clock protein KaiC	regulatory functions
asl4146	0,000	4,183	4,183	sulfiredoxin	detoxification
asl0814	0,000	4,183	4,183	unknown	unknown
asl4395	0,000	4,183	4,183	unknown	unknown
alr7305	0,861	3,618	4,204	transposase	transposon-related functions
alr2014	2,320	9,751	4,204	unknown	unknown
asl8072	0,000	4,280	4,280	transcriptional regulator	regulatory functions
alr0149	0,000	4,280	4,280	unknown	unknown
asr3657	0,000	4,280	4,280	unknown	unknown
asr3961	0,000	4,331	4,331	unknown	unknown
alr0477	1,783	7,728	4,335	phospho-N-acetylmuramoyl-pentapeptide-transferase	cell wall/membrane biogenesis
alr0301	1,123	4,867	4,335	unknown	unknown
asl4362	0,000	4,435	4,435	tRNA (guanine-N1)-methyltransferase	RNA synthesis and modification
alr3883	0,000	4,435	4,435	2-C-methyl-D-erythritol 2,4-cyclodiphosphate synthase	terpenoid biosynthesis
all7116	0,000	4,526	4,526	unknown	unknown
asl4918	0,000	4,545	4,545	putative transposase	transposon-related functions
asr0941	0,000	4,601	4,601	photosystem II protein PsbX	photosynthesis
asr1131	0,000	4,719	4,719	signal transduction protein	regulatory functions
all7348	0,507	2,396	4,729	lethal leaf-spot 1 homolog	chlorophyll catabolism, developmentally programmed death
alr1450	0,254	1,200	4,729	cytochrome P450	electron transport chain
all2031	0,320	1,513	4,729	cyclic nucleotide binding protein	regulatory functions

alr1629	0,852	4,030	4,729	transcriptional regulator	regulatory functions
alr2731	0,383	1,810	4,729	cytochrome c oxidase subunit II	respiration, oxidative phosphorylation
all7010	0,241	1,138	4,729	similar to hemolysin export system membrane fusion protein HlyD	transport across membrane
alr5362	0,237	1,122	4,729	sugar ABC transporter ATP binding protein	transport across membrane
all3371	0,871	4,121	4,729	transposase	transposon-related functions
alr3388	1,134	5,361	4,729	unknown	unknown
all4508	1,134	5,361	4,729	unknown	unknown
alr2431	0,805	3,808	4,729	unknown	unknown
all2005	0,773	3,657	4,729	unknown	unknown
alr1301	0,531	2,510	4,729	unknown	unknown
all1340	0,531	2,510	4,729	unknown	unknown
all4737	0,521	2,465	4,729	unknown	unknown
all8516	0,514	2,432	4,729	unknown	unknown
asl3923	2,162	10,225	4,729	unknown	unknown
alr4513	0,631	2,985	4,729	unknown	unknown
alr4919	0,176	0,833	4,729	unknown	unknown
all2162	0,155	0,733	4,729	methyl-accepting chemotaxis protein	chemotaxis and cell motility
all5371	0,891	4,215	4,729	alkylhydroperoxidase AhpD	detoxification
alr3100	0,768	3,633	4,729	holliday junction resolvase YqgF	DNA replication, recombination and repair
alr2873	0,348	1,643	4,729	glycerate kinase	glycine/serine/threonine/glyoxylate metabolism
all2566	0,339	1,605	4,729	glyceraldehyde-3-phosphate dehydrogenase	glycolisis
all1455	0,789	3,731	4,729	nitrogenase iron protein NifH	nitrogen fixation
alr2189	0,341	1,615	4,729	regulatory protein	regulatory functions
alr4485	0,868	4,105	4,729	ABC transporter, permease protein	transport across membrane
alr7511	0,323	1,530	4,729	integrase-recombinase protein	transposon-related functions
all3256	0,868	4,105	4,729	pentapepetide-repeat containing protein	unknown
asl7217	2,883	13,634	4,729	unknown	unknown
alr1810	1,021	4,829	4,729	unknown	unknown
alr0896	0,834	3,944	4,729	unknown	unknown
alr2947	0,811	3,835	4,729	unknown	unknown
all5029	0,739	3,495	4,729	unknown	unknown
alr2823	0,447	2,116	4,729	unknown	unknown
asr0581	0,000	4,781	4,781	unknown	unknown
asl2455	0,000	5,185	5,185	protein secretion chaperonin CsaA	heat shock proteins and chaperones
all3319	0,000	5,467	5,467	unknown	unknown
asl1187	0,000	5,494	5,494	surface antigen variable number	membrane component
all0315	1,717	9,474	5,517	transposase	transposon-related functions
alr4567	1,156	6,378	5,517	transcriptional regulator	regulatory functions
all8041	5,560	30,676	5,517	unknown	unknown
asl3656	0,000	5,577	5,577	unknown	unknown
all8067	1,460	8,283	5,675	unknown	unknown

all8037	6,322	36,126	5,714	unknown	unknown
all4750	1,530	8,841	5,780	two-component response regulator	regulatory functions
asr5364	0,000	5,843	5,843	unknown	unknown
asr8054	0,000	6,204	6,204	unknown	unknown
asr4676	0,000	6,239	6,239	unknown	unknown
all3898	0,423	2,667	6,305	2-hydroxy-6-ketonona-2,4-dienoate hydrolase	catabolic pathways
all1681	0,350	2,204	6,305	aspartate carbamoyltransferase	purine/pyrimidine biosynthesis and metabolism
all5120	0,178	1,124	6,305	unknown	unknown
all1847	0,611	3,855	6,305	CheB methylesterase	chemotaxis and cell motility
alr3195	0,475	2,993	6,305	glutathione S-transferase	detoxification
all3135	0,383	2,414	6,305	succinoglycan biosynthesis ketolase ExoV	exopolysaccharide biosynthesis
all3036	1,007	6,347	6,305	Nitrate transport permease	nitrogen metabolism
alr3666	0,417	2,629	6,305	urease accessory protein D	nitrogen metabolism
alr0894	0,482	3,042	6,305	cyclase/dehydrase	polyketide biosynthesis
all0322	0,306	1,927	6,305	sulfate-binding protein SbpA	transport across membrane
alr2168	0,295	1,859	6,305	transposase	transposon-related functions
all4886	0,763	4,812	6,305	unknown	unknown
all0244	0,667	4,207	6,305	unknown	unknown
alr2783	0,374	2,360	6,305	unknown	unknown
alr1733	1,091	6,881	6,305	unknown	unknown
alr4734	0,645	4,068	6,305	unknown	unknown
alr0034	0,364	2,294	6,305	O-succinylbenzoic acid synthase; MenC	vitamin biosynthesis
asl0940	1,191	7,513	6,305	YGGT-containing protein	membrane component
all4400	0,912	5,752	6,305	transposase	transposon-related functions
alr4712	0,510	3,215	6,305	unknown	unknown
all8002	0,410	2,583	6,305	unknown	unknown
alr3382	0,000	6,535	6,535	molybdenum cofactor biosynthesis protein C	coenzymes and cofactors
alr1791	0,000	6,693	6,693	putative transposase	transposon-related functions
all7004	1,412	9,648	6,831	transposase	transposon-related functions
all8046	5,615	38,834	6,917	unknown	unknown
asl4753	0,000	6,946	6,946	unknown	unknown
all8047	9,907	70,276	7,094	unknown	unknown
asl1671	0,000	7,218	7,218	unknown	unknown
asl7509	0,000	7,564	7,564	unknown	unknown
all1474	0,345	2,723	7,882	CRISPR-associated RAMP protein	DNA replication, recombination and repair
alr3513	0,805	6,347	7,882	Fe-S metabolism associated SufE	Fe-S cluster biosynthesis
all3765	0,263	2,073	7,882	two-component hybrid sensor and regulator	regulatory functions
asr4653	1,914	15,087	7,882	unknown	unknown
alr2045	0,460	3,623	7,882	Thioesterase	lipid metabolism
alr3738	0,519	4,090	7,882	Mo-dependent nitrogenase	nitrogen fixation
all2127	0,516	4,063	7,882	radical SAM, Fe-S containing enzyme	other enzymes
all8035	4,246	33,465	7,882	unknown	unknown

alr0804	0,758	5,976	7,882	unknown	unknown
all8032	2,817	23,686	8,407	unknown	unknown
all8040	3,862	33,660	8,716	unknown	unknown
alr4996	0,000	8,765	8,765	unknown	unknown
all1649	0,074	0,703	9,458	similar to polyketide synthase	siderophore/cyanotoxin biosynthesis
all0166	0,235	2,226	9,458	aapha,alpha-trehalase	sugars
asl9022	1,441	13,634	9,458	putative transposase	transposon-related functions
alr1868	1,168	11,043	9,458	unknown	unknown
asl2299	0,000	9,816	9,816	unknown	unknown
asl8049	0,000	10,225	10,225	unknown	unknown
asl1777	0,000	10,827	10,827	putative ATPase	transport across membrane
alr8066	0,417	4,601	11,035	putative N-acetyltransferase	other enzymes
alr8060	1,287	16,232	12,611	unknown	unknown
all8087	0,351	4,422	12,611	glycerate dehydrogenase	glycine/serine/threonine/glyoxylate metabolism
alr3479	0,778	9,816	12,611	similar to nitrogen regulation protein NtrR	nitrogen metabolism
all8039	2,507	33,876	13,512	putative DNA primase	DNA replication, recombination and repair
all8044	1,123	15,928	14,187	unknown	unknown
alr8061	0,416	6,550	15,764	unknown	unknown
alr8058	1,734	30,069	17,340	unknown	unknown
asr8057	0,000	17,529	17,529	unknown	unknown
alr8059	0,000	19,642	19,642	unknown	unknown
all8038	1,219	26,912	22,069	unknown	unknown
alr8052	0,000	22,839	22,839	putative cobalt ABC transporter ATP-binding protein	transport across membrane
asl8050	0,000	23,007	23,007	copy number control protein CopB	nucleoproteins
alr8056	0,878	20,758	23,645	unknown	unknown
alr8051	1,877	49,121	26,168	unknown	unknown
all8036	0,000	41,207	41,207	unknown	unknown

Table C. Genes showing <2-fold change in expression in the *furA*-turning off strain AGcoaRFurA

ORF	RPKM (WT)	RPKM (FurA$^-$)	Fold change
all2409	1,996	0,000	-1,996
all4434	1,996	0,000	-1,996
alr5143	1,996	0,000	-1,996
all7594	1,996	0,000	-1,996
all4364	12,350	6,194	-1,994
all3288	4,884	2,449	-1,994
all3226	37,837	18,978	-1,994
all2192	9,842	4,936	-1,994
alr3406	5,536	2,777	-1,994
psbB	228,027	114,404	-1,993
apcC	345,206	173,387	-1,991
all1426	1,990	0,000	-1,990
all3866	1,990	0,000	-1,990
alr7063	1,987	0,000	-1,987
dnaK_3	47,490	23,922	-1,985
alr3494	23,167	11,686	-1,982
all1541	11,583	5,843	-1,982
all5174	4,849	2,446	-1,982
psaD	3762,206	1898,420	-1,982
psbD_1	247,454	124,971	-1,980
all0195	1,979	0,000	-1,979
asl2383	1,979	0,000	-1,979
alr4783	1,979	0,000	-1,979
all7185	1,979	0,000	-1,979
psbE	74,558	37,698	-1,978
all3054	9,103	4,606	-1,976
alr1890	17,946	9,081	-1,976
alr3400	16,766	8,495	-1,974
alr3414	25,541	12,942	-1,974
alr0740	6,423	3,254	-1,974
alr4404	30,698	15,554	-1,974
all0850	10,379	5,259	-1,974
alr7506	9,006	4,563	-1,974

all1061	4,365	2,212	-1,974
all4042	5,923	3,001	-1,974
alr9024	5,923	3,001	-1,974
alr4596	21,893	11,107	-1,971
alr3561	21,494	10,911	-1,970
all0854	32,318	16,434	1,967
desC_2	12,656	6,436	-1,967
alr5294	3,348	1,703	-1,967
all1103	32,032	16,288	-1,967
alr1728	8,901	4,540	-1,961
all4752	12,066	6,154	-1,961
petC_4	1,957	0,000	-1,957
alr3952	36,001	18,406	-1,956
alr2338	5,745	2,937	-1,956
all7245	32,624	16,691	-1,955
all0887	1,946	0,000	-1,946
asl1739	1,946	0,000	-1,946
alr4649	1,946	0,000	-1,946
asr7145	1,946	0,000	-1,946
all7284	1,946	0,000	-1,946
alr2717	23,978	12,325	-1,945
all4252	46,704	24,050	-1,942
coxA_1	11,757	6,071	-1,937
rbcS	142,236	73,623	-1,932
trpD_2	1,930	0,000	-1,930
tuf	61,513	31,873	-1,930
all1345	21,591	11,196	-1,928
rbpA2	88,693	46,014	-1,928
alr0974	1,927	0,000	-1,927
alr3353	66,129	34,344	-1,925
pecC	197,950	102,914	-1,923
alr0975	1,922	0,000	-1,922
alr4466	1,920	0,000	-1,920
alr5230	1,919	0,000	-1,919
asr1915	1,914	0,000	-1,914
alr4650	1,914	0,000	-1,914
asr7293	1,914	0,000	-1,914
alr4550	114,937	60,074	-1,913
alr2112	1,904	0,000	-1,904
alr1652	4,042	2,124	-1,903
alr3449	2,245	1,180	-1,903
smpB	2,245	1,180	-1,903

alr2536	2,210	1,161	-1,903
alr0353	1,145	0,601	-1,903
psaM	34,174	17,957	-1,903
ndhF_2	24,625	12,939	-1,903
alr4785	14,011	7,362	-1,903
all7005	11,975	6,293	-1,903
asl4369	11,062	5,812	-1,903
alr4417	10,796	5,673	-1,903
rpsQ	8,543	4,489	-1,903
asl8027	8,242	4,331	-1,903
all1972	7,374	3,875	-1,903
fmt	6,127	3,220	-1,903
all2091	6,081	3,195	-1,903
all0047	5,926	3,114	-1,903
alr3363	5,638	2,963	-1,903
all3580	5,417	2,846	-1,903
alr7102	5,417	2,846	-1,903
alr3058	5,139	2,700	-1,903
asl1513	4,934	2,592	-1,903
all2395	4,712	2,476	-1,903
all2096	4,549	2,390	-1,903
alr3453	4,549	2,390	-1,903
hetI	4,351	2,286	-1,903
alr0618	4,324	2,272	-1,903
all0002	4,272	2,245	-1,903
alr3361	3,992	2,098	-1,903
all2976	3,936	2,068	-1,903
asl8537	3,936	2,068	-1,903
alr7192	3,914	2,057	-1,903
all4580	3,903	2,051	-1,903
asl1412	3,807	2,001	-1,903
alr3126	3,799	1,996	-1,903
alr0851	3,726	1,958	-1,903
alr2988	3,707	1,948	-1,903
alr3050	3,611	1,897	-1,903
gatC	3,611	1,897	-1,903
alr7524	3,611	1,897	-1,903
all1641	3,602	1,893	-1,903
alr2629	3,368	1,770	-1,903
alr3452	3,368	1,770	-1,903
all1021	3,333	1,751	-1,903
all2620	3,261	1,714	-1,903

alr2944	2,907	1,527	-1,903
alr0302	2,883	1,515	-1,903
alr7634	2,848	1,496	-1,903
all0439	2,769	1,455	-1,903
alr4066	2,674	1,405	-1,903
all9002	2,416	1,269	-1,903
all1943	2,335	1,227	-1,903
alr1045	2,304	1,211	-1,903
aphA	2,286	1,201	-1,903
all2286	2,060	1,083	-1,903
all0827	1,968	1,034	-1,903
alr1341	1,946	1,023	-1,903
alr0593	1,925	1,011	-1,903
alr3752	1,904	1,000	-1,903
alr1410	1,853	0,974	-1,903
all2905	1,844	0,969	-1,903
alr4536	1,806	0,949	-1,903
all0168	1,692	0,889	-1,903
alr1529	1,692	0,889	-1,903
alr4095	1,614	0,848	-1,903
alr0598	1,510	0,793	-1,903
alr3904	1,497	0,787	-1,903
alr0515	1,453	0,764	-1,903
alr1183	1,342	0,705	-1,903
all4461	1,302	0,684	-1,903
alr5189	1,302	0,684	-1,903
phnE_1	1,274	0,669	-1,903
all2989	1,238	0,650	-1,903
alr2214	1,196	0,628	-1,903
alr1997	1,164	0,611	-1,903
alr3017	1,027	0,540	-1,903
alr4494	0,984	0,517	-1,903
alr3584	0,978	0,514	-1,903
all7335	0,973	0,511	-1,903
alr0838	0,927	0,487	-1,903
alr2241	0,927	0,487	-1,903
all5113	0,857	0,451	-1,903
alr5202	0,815	0,428	-1,903
moeA	0,802	0,421	-1,903
all7583	0,750	0,394	-1,903
alr0557	0,587	0,308	-1,903
alr0428	0,549	0,288	-1,903

all0167	0,380	0,199	-1,903
all0432	0,378	0,199	-1,903
all2647	0,322	0,169	-1,903
all7607	28,109	14,770	-1,903
asr2607	26,537	13,944	-1,903
alr3801	26,189	13,761	-1,903
hoxR	25,020	13,147	-1,903
recA	24,461	12,853	-1,903
typA	21,123	11,099	-1,903
alr4641	20,605	10,827	-1,903
asr2985	19,460	10,225	-1,903
alr2304	16,217	8,521	-1,903
all4145	14,951	7,856	-1,903
all4779	11,485	6,035	-1,903
asl0551	11,120	5,843	-1,903
all4342	9,937	5,221	-1,903
all7355	8,407	4,417	-1,903
all0085	8,194	4,305	-1,903
alr1961	7,961	4,183	-1,903
ribG	7,682	4,036	-1,903
alr2940	7,648	4,019	-1,903
alr1921	7,298	3,835	-1,903
radC	7,178	3,772	-1,903
all4272	6,504	3,418	-1,903
asl1249	6,487	3,408	-1,903
all0544	6,427	3,377	-1,903
alr2954	5,742	3,017	-1,903
asl0873	5,151	2,707	-1,903
alr0821	4,969	2,611	-1,903
asl2047	4,798	2,521	-1,903
alr2049	4,520	2,375	-1,903
asl4137	4,379	2,301	-1,903
alr5352	4,298	2,258	-1,903
alr7504	4,200	2,207	-1,903
alr2774	4,097	2,153	-1,903
alr4788	4,026	2,116	-1,903
all1342	3,942	2,071	-1,903
alr5147	3,908	2,054	-1,903
all5043	3,881	2,039	-1,903
alr0893	3,839	2,017	-1,903
hetN	3,649	1,917	-1,903
all2876	3,604	1,894	-1,903

all4926	3,574	1,878	-1,903
alr2881	3,325	1,747	-1,903
alr4944	3,289	1,728	-1,903
kaiB_1	3,214	1,689	-1,903
all0956	3,156	1,658	-1,903
alr3645	3,046	1,600	-1,903
alr0704	3,028	1,591	-1,903
all0625	2,956	1,553	-1,903
all5215	2,953	1,552	-1,903
alr5260	2,944	1,547	-1,903
all0591	2,825	1,484	-1,903
all3626	2,825	1,484	-1,903
alr1869	2,814	1,478	-1,903
alr4270	2,802	1,472	-1,903
alr3994	2,576	1,353	-1,903
all3918	2,520	1,324	-1,903
alr2694	2,351	1,235	-1,903
alr1715	2,182	1,147	-1,903
alr8001	2,169	1,140	-1,903
all2706	2,123	1,115	-1,903
all4284	2,110	1,109	-1,903
glk	2,042	1,073	-1,903
all3160	2,037	1,070	-1,903
alr7670	2,025	1,064	-1,903
all1357	2,013	1,058	-1,903
alr7326	2,013	1,058	-1,903
alr7512	2,002	1,052	-1,903
alr3026	1,893	0,995	-1,903
all5105	1,891	0,994	-1,903
alr0078	1,883	0,990	-1,903
all0123	1,676	0,881	-1,903
alr0566	1,645	0,864	-1,903
ubiE	1,523	0,800	-1,903
glgA_1	1,421	0,747	-1,903
all4180	1,401	0,736	-1,903
all3007	1,390	0,730	-1,903
fabG_3	1,390	0,730	-1,903
alr1030	1,379	0,725	-1,903
panB	1,358	0,713	-1,903
alr2153	1,309	0,688	-1,903
hemD	1,265	0,664	-1,903
alr0429	1,265	0,664	-1,903

all7366	1,220	0,641	-1,903
alr0931	1,200	0,630	-1,903
all4652	1,187	0,624	-1,903
all4411	1,183	0,622	-1,903
alr2589	1,102	0,579	-1,903
alr3096	1,095	0,575	-1,903
alr2836	1,084	0,570	-1,903
all2237	1,039	0,546	-1,903
all4730	1,021	0,537	-1,903
all4096	0,981	0,516	-1,903
all2134	0,944	0,496	-1,903
all2911	0,907	0,477	-1,903
gvpN	0,852	0,448	-1,903
all4897	0,852	0,448	-1,903
all0930	0,848	0,446	-1,903
all0392	0,818	0,430	-1,903
all4298	0,715	0,376	-1,903
alr2179	0,684	0,359	-1,903
alr1928	0,683	0,359	-1,903
alr3934	0,680	0,357	-1,903
alr7572	0,578	0,304	-1,903
alr1037	0,520	0,273	-1,903
alr2691	0,519	0,273	-1,903
alr1586	0,423	0,222	-1,903
alr3907	16,334	8,583	-1,903
alr2791	5,593	2,939	-1,903
purT	5,361	2,817	-1,903
alr1493	3,305	1,736	-1,903
alr3231	3,305	1,736	-1,903
alr4711	2,478	1,302	-1,903
alr0446	1,652	0,868	-1,903
all7626	1,352	0,711	-1,903
alr0663	1,107	0,582	-1,903
alr3073	1,078	0,566	-1,903
alr1855	0,597	0,314	-1,903
all2961	1,902	0,000	-1,902
alr4318	110,773	58,521	-1,893
ssrA	17975,802	9511,516	-1,890
asl0448	1,883	0,000	-1,883
asr1818	1,883	0,000	-1,883
asl4948	1,883	0,000	-1,883
all1716	6,214	3,311	-1,877

alr2492	15,811	8,436	-1,874
alr0331	9,500	5,072	-1,873
alr1614	7,314	3,912	-1,870
alr3649	1,868	0,000	-1,868
all4666	1,868	0,000	-1,868
all1338	14,595	7,814	-1,868
asl7296	95,706	51,295	-1,866
all4459	1,863	0,000	-1,863
alr0142	16,955	9,112	-1,861
alr5186	1,861	0,000	-1,861
alr1397	15,222	8,190	-1,859
all0853	7,092	3,817	-1,858
all3540	15,734	8,485	-1,854
all4387	15,903	8,576	-1,854
asr1202	1,853	0,000	-1,853
asl2601	1,853	0,000	-1,853
alr4552	12,731	6,881	-1,850
all3916	10,642	5,752	-1,850
alr7297	5,724	3,093	-1,850
rfbB_2	11,975	6,489	-1,845
asr1661	42,862	23,298	-1,840
alr2090	11,231	6,105	-1,840
alr7345	11,138	6,055	-1,840
all3029	1,839	0,000	-1,839
alr3204	1,839	0,000	-1,839
fda	128,761	70,044	-1,838
alr1196	8,480	4,627	-1,833
alr4870	4,312	2,353	-1,833
atpH	37,022	20,201	-1,833
all4422	11,767	6,421	-1,833
all7614	11,080	6,046	-1,833
kaiB_2	1,832	0,000	-1,832
alr0212	19,071	10,430	-1,828
all0016	1,824	0,000	-1,824
asr2896	1,824	0,000	-1,824
asl4356	1,824	0,000	-1,824
asr8523	1,824	0,000	-1,824
all2996	8,340	4,573	-1,824
all0187	26,642	14,608	-1,824
all3420	12,849	7,045	-1,824
ndhA	14,399	7,895	-1,824
alr4398	2,496	1,368	-1,824

alr0991	29,696	16,313	-1,820
alr3415	20,226	11,111	-1,820
alr2851	18,074	9,949	-1,817
all3902	18,148	9,996	-1,816
alr4599	1,815	0,000	-1,815
psaI	49,686	27,413	-1,812
leuA_1	8,570	4,728	-1,812
all2161	2,395	1,321	-1,812
all0949	26,918	14,879	-1,809
alr3280	19,727	10,926	-1,806
all0641	43,202	23,927	-1,806
psbC	190,625	105,633	-1,805
rplC	29,741	16,496	-1,803
all5094	17,227	9,555	-1,803
tsf	32,723	18,171	-1,801
pgk	25,623	14,229	-1,801
psbAI	629,086	349,760	-1,799
alr2592	2,335	1,299	-1,797
all0929	16,270	9,052	-1,797
all0632	6,594	3,669	-1,797
alr3887	4,296	2,390	-1,797
alr0506	1,796	0,000	-1,796
alr1419	1,796	0,000	-1,796
asr7595	1,796	0,000	-1,796
all5241	12,438	6,957	-1,788
alr2464	9,971	5,577	-1,788
all1871	961,033	538,014	-1,786
aphB	6,987	3,916	-1,784
alr1109	1,783	0,000	-1,783
all2955	1,783	0,000	-1,783
alr4995	9,785	5,490	-1,782
alr3589	8,092	4,556	-1,776
all7065	4,894	2,755	-1,776
alr3618	2,104	1,184	-1,776
cbiQ_1	12,245	6,894	-1,776
alr1144	11,235	6,325	-1,776
alr0039	5,598	3,152	-1,776
cysS	3,357	1,890	-1,776
all1696	0,817	0,460	-1,776
asl1496	23,352	13,147	-1,776
asr3134	23,352	13,147	-1,776
all1304	11,391	6,413	-1,776

alr4010	8,298	4,672	-1,776
all4343	7,419	4,177	-1,776
all5309	6,986	3,933	-1,776
crhC	3,828	2,155	-1,776
alr4787	3,199	1,801	-1,776
alr7025	1,776	0,000	-1,776
all0706	1,774	0,000	-1,774
all1951	17,820	10,062	-1,771
asr3834	1,769	0,000	-1,769
asr4352	1,769	0,000	-1,769
all4980	1,769	0,000	-1,769
asr5175	1,769	0,000	-1,769
alr3260	19,053	10,782	-1,767
alr5361	13,048	7,383	-1,767
all1835	27,273	15,450	-1,765
all0626	8,019	4,551	-1,762
all5073	6,132	3,480	-1,762
all3321	12,421	7,049	-1,762
all0497	9,508	5,396	-1,762
all1636	3,042	1,731	-1,757
alr1740	1,756	0,000	-1,756
all2907	1,756	0,000	-1,756
ftsH_1	44,689	25,480	-1,754
alr1346	20,647	11,784	-1,752
all0648	10,442	5,986	-1,745
all0542	4,475	2,565	-1,745
all1699	4,369	2,504	-1,745
all0723	3,528	2,023	-1,745
alr5214	3,528	2,023	-1,745
all2897	1,985	1,138	-1,745
rpoA	28,451	16,309	-1,745
all0270	16,900	9,687	-1,745
asr0460	15,663	8,978	-1,745
alr7100	7,977	4,573	-1,745
all1770	7,533	4,318	-1,745
pecF	7,381	4,231	-1,745
all1503	7,135	4,090	-1,745
all4904	5,465	3,133	-1,745
rph	5,179	2,969	-1,745
hisB_1	4,865	2,789	-1,745
alr5125	3,008	1,724	-1,745
alr3926	2,899	1,662	-1,745

hlyD	2,564	1,470	-1,745
alr1655	2,513	1,441	-1,745
all0635	1,562	0,896	-1,745
alr2366	21,586	12,374	-1,745
alr0445	9,861	5,652	-1,745
all0253	6,030	3,456	-1,745
all4008	4,354	2,496	-1,745
asr4594	1,743	0,000	-1,743
asr4942	1,743	0,000	-1,743
ftsH_2	23,389	13,460	-1,738
alr7301	1,736	0,000	-1,736
all4184	1,734	0,000	-1,734
alr5031	1,734	0,000	-1,734
alr8534	1,732	0,000	-1,732
alr4543	34,681	20,046	-1,730
alr2765	8,670	5,011	-1,730
alr4258	14,790	8,589	-1,722
alr2744	5,183	3,010	-1,722
alr1674	2,966	1,722	-1,722
alr0444	17,607	10,225	-1,722
ribH	11,615	6,746	-1,722
alr4370	8,803	5,113	-1,722
alr4530	5,703	3,312	-1,722
all3652	5,667	3,291	-1,722
alr3690	1,721	0,000	-1,721
thrC_2	55,294	32,157	-1,719
glgB	12,821	7,459	-1,719
asr2755	1,717	0,000	-1,717
asl2844	1,717	0,000	-1,717
alr5233	1,717	0,000	-1,717
rpsI	38,640	22,511	-1,717
all3741	25,020	14,608	-1,713
all4231	1,711	0,000	-1,711
minD_3	15,192	8,895	-1,708
all4183	14,466	8,470	-1,708
alr4533	9,982	5,855	-1,705
alr0627	5,529	3,243	-1,705
all2798	1,705	0,000	-1,705
alr5034	1,705	0,000	-1,705
all8003	1,705	0,000	-1,705
cpcG1	39,198	23,007	-1,704
alr0599	26,987	15,917	-1,696

alr0734	1,692	0,000	-1,692
all7131	1,692	0,000	-1,692
alr7298	1,692	0,000	-1,692
asr0785	15,313	9,052	-1,692
all2874	11,288	6,673	-1,692
all3872	6,997	4,136	-1,692
alr5338	6,997	4,136	-1,692
alr4554	6,420	3,795	-1,692
alr5367	2,661	1,573	-1,692
alr3059	2,566	1,517	-1,692
alr0564	2,347	1,387	-1,692
asl4138	18,870	11,155	-1,692
alr7342	18,436	10,898	-1,692
asl3261	14,153	8,366	-1,692
all1908	9,781	5,782	-1,692
alr2139	9,248	5,467	-1,692
all5063	8,939	5,284	-1,692
all0538	6,894	4,075	-1,692
all4762	6,509	3,848	-1,692
all3171	6,085	3,597	-1,692
alr7233	5,463	3,229	-1,692
all4163	4,670	2,761	-1,692
alr2735	4,427	2,617	-1,692
alr0490	4,133	2,443	-1,692
all4245	3,992	2,360	-1,692
all1106	3,925	2,320	-1,692
all4969	3,600	2,128	-1,692
alr4691	3,447	2,038	-1,692
alr3358	3,188	1,885	-1,692
alr0999	3,135	1,853	-1,692
alr3884	3,135	1,853	-1,692
all2260	3,103	1,834	-1,692
alr3120	2,903	1,716	-1,692
all2908	2,538	1,500	-1,692
alr0871	2,478	1,465	-1,692
alr1627	2,478	1,465	-1,692
all2748	2,188	1,293	-1,692
all7382	2,071	1,224	-1,692
coxA_2	1,668	0,986	-1,692
alr0205	1,299	0,768	-1,692
alr1184	1,098	0,649	-1,692
alr0774	7,241	4,280	-1,692

alr4016	7,241	4,280	-1,692
all4256	7,023	4,152	-1,692
coaD	4,865	2,876	-1,692
all0160	2,426	1,434	-1,692
alr1142	1,789	1,058	-1,692
alr0242	1,687	0,000	-1,687
alr7304	2,956	1,752	-1,687
alr3808	53,647	31,837	-1,685
all2156	1,680	0,000	-1,680
allrt43	180,385	107,459	-1,679
alr1403	1,677	0,000	-1,677
all1606	10,000	5,965	-1,677
all7571	1,676	0,000	-1,676
alr0942	11,249	6,726	-1,672
accD	18,417	11,032	-1,669
rpsB	21,948	13,147	-1,669
all3526	21,041	12,613	-1,668
asr3081	1,668	0,000	-1,668
alr7308	1,668	0,000	-1,668
alr2079	8,354	5,017	-1,665
alr0546	5,330	3,201	-1,665
cpcG3	41,210	24,747	-1,665
alr0093	3,904	2,345	-1,665
all3375	9,263	5,572	-1,662
alr0973	1,660	0,000	-1,660
frr	21,693	13,075	-1,659
leuS	10,833	6,536	-1,658
rps1_1	31,906	19,262	-1,656
hupB	1,656	0,000	-1,656
alr4634	1,656	0,000	-1,656
alr4949	30,930	18,690	-1,655
psaK	314,047	190,404	-1,649
asr4313	37,479	22,723	-1,649
all3819	5,383	3,263	-1,649
all2529	3,343	2,027	-1,649
alr3771	3,042	1,844	-1,649
all4902	3,776	2,289	-1,649
all1176	1,747	1,059	-1,649
rnpB	1393,517	845,063	-1,649
alr0683	1,645	0,000	-1,645
all0781	10,816	6,587	-1,642
alr0970	11,418	6,967	-1,639

alr7208	9,756	5,953	-1,639
alr3437	4,570	2,789	-1,639
cpcC	252,236	154,557	-1,632
alr2570	7,426	4,553	-1,631
ftrC	34,454	21,121	-1,631
secY	14,395	8,825	-1,631
ftsZ	9,798	6,007	-1,631
alr4147	3,780	2,317	-1,631
all4261	2,027	1,242	-1,631
alr0141	6,145	3,767	-1,631
alr4075	4,050	2,482	-1,631
all1703	3,766	2,309	-1,631
all4495	2,719	1,667	-1,631
all1881	1,629	0,000	-1,629
all1861	41,083	25,223	-1,629
alr5229	1,627	0,000	-1,627
all4933	10,207	6,279	-1,626
asl1066	1,622	0,000	-1,622
alr2093	1,622	0,000	-1,622
alr2276	1,622	0,000	-1,622
asr3217	1,622	0,000	-1,622
asl3480	1,622	0,000	-1,622
psaF	260,412	160,632	-1,621
alr2722	1,618	0,000	-1,618
all2430	4,502	2,785	-1,617
all1284	1,616	0,000	-1,616
all3550	15,643	9,687	-1,615
rpsP	37,578	23,272	-1,615
alr0202	8,072	4,999	-1,615
alr1753	7,031	4,354	-1,615
pknA	5,786	3,583	-1,615
alr0213	31,638	19,593	-1,615
alr2018	1,614	0,000	-1,614
alr3601	20,825	12,910	-1,613
alr5032	1,613	0,000	-1,613
atpF	20,495	12,727	-1,610
all4967	1,607	0,000	-1,607
ccmM	46,200	28,800	-1,604
alr2935	15,066	9,401	-1,603
all0924	1,599	0,000	-1,599
alr2331	1,599	0,000	-1,599
asr3005	1,599	0,000	-1,599

asl5268	1,599	0,000	-1,599
apcF	53,573	33,563	-1,596
all7182	1,589	0,000	-1,589
all7013	17,341	10,934	-1,586
all3263	10,761	6,786	-1,586
alr4334	10,031	6,325	-1,586
asr5092	9,267	5,843	-1,586
alr4740	8,936	5,634	-1,586
alr2136	7,485	4,719	-1,586
all1849	6,415	4,045	-1,586
all4764	5,838	3,681	-1,586
all1079	5,166	3,258	-1,586
alr3419	5,099	3,215	-1,586
alr0964	5,077	3,201	-1,586
lpxD	5,004	3,155	-1,586
murE	4,699	2,963	-1,586
alr7294	4,597	2,899	-1,586
all0473	4,526	2,854	-1,586
all2456	4,406	2,778	-1,586
all3373	4,230	2,667	-1,586
all4901	3,909	2,465	-1,586
alr3790	3,841	2,422	-1,586
all1227	3,327	2,098	-1,586
alr5249	3,243	2,045	-1,586
all3197	3,122	1,969	-1,586
alr2921	2,919	1,841	-1,586
alr3554	2,905	1,831	-1,586
alr1799	2,897	1,827	-1,586
alr5231	2,538	1,600	-1,586
all4727	2,443	1,540	-1,586
all4013	2,131	1,343	-1,586
all0862	1,902	1,199	-1,586
alr3303	1,774	1,119	-1,586
alr4033	1,748	1,102	-1,586
all0666	1,673	1,055	-1,586
alr1094	1,654	1,043	-1,586
alr5238	1,524	0,961	-1,586
alr4602	1,493	0,941	-1,586
alr0776	1,471	0,927	-1,586
all5106	1,431	0,902	-1,586
all0959	1,242	0,783	-1,586
alr4738	1,189	0,750	-1,586

alr0369	1,163	0,733	-1,586
all4620	1,121	0,707	-1,586
all4446	1,012	0,638	-1,586
alr5067	20,131	12,694	-1,586
alr3426	18,832	11,875	-1,586
hoxU	17,099	10,782	-1,586
purK	13,268	8,366	-1,586
fabZ	10,183	6,421	-1,586
asl0822	8,108	5,113	-1,586
exbB	8,034	5,066	-1,586
ureE	7,889	4,975	-1,586
alr2762	6,710	4,231	-1,586
alr2741	6,380	4,023	-1,586
btpA	6,189	3,902	-1,586
alr4528	5,988	3,776	-1,586
all4778	5,578	3,517	-1,586
all2740	5,456	3,440	-1,586
alr4514	5,260	3,316	-1,586
all4283	4,670	2,945	-1,586
all4782	4,670	2,945	-1,586
pknB	3,791	2,390	-1,586
alr2538	3,779	2,383	-1,586
alr4795	3,695	2,330	-1,586
alr2426	3,355	2,116	-1,586
all5074	3,355	2,116	-1,586
alr1628	3,105	1,958	-1,586
all7648	3,041	1,917	-1,586
all5173	3,022	1,905	-1,586
alr0787	3,009	1,897	-1,586
thrB	2,994	1,888	-1,586
all3010	2,852	1,799	-1,586
alr2118	2,820	1,778	-1,586
all2116	2,800	1,766	-1,586
all4906	2,741	1,728	-1,586
hisD_2	2,703	1,704	-1,586
all2459	2,636	1,662	-1,586
alr0072	2,595	1,636	-1,586
all3767	2,516	1,587	-1,586
alr2307	2,479	1,563	-1,586
alr4837	2,433	1,534	-1,586
alr3910	2,308	1,455	-1,586
all4155	2,092	1,319	-1,586

purU	2,048	1,292	-1,586
alr5028	1,966	1,239	-1,586
all3232	1,959	1,235	-1,586
all2900	1,940	1,223	-1,586
all4458	1,830	1,154	-1,586
alr4674	1,824	1,150	-1,586
mtnA	1,678	1,058	-1,586
all8544	1,668	1,052	-1,586
alr1786	1,631	1,028	-1,586
alr1097	1,549	0,976	-1,586
all0729	1,419	0,895	-1,586
all1191	1,339	0,844	-1,586
all5115	1,277	0,806	-1,586
alr3749	1,250	0,788	-1,586
all5193	0,971	0,613	-1,586
hglC	0,526	0,332	-1,586
rnpA	16,562	10,443	-1,586
all3569	4,561	2,876	-1,586
asl0639	1,578	0,000	-1,578
alr2081	1,578	0,000	-1,578
petA	33,910	21,492	-1,578
sigA	63,606	40,483	-1,571
rpsC	21,026	13,399	-1,569
alr0725	1,567	0,000	-1,567
alr1569	1,567	0,000	-1,567
alr1967	1,567	0,000	-1,567
all3850	10,542	6,731	-1,566
atpI	59,307	37,980	-1,562
all3985	6,075	3,891	-1,562
truA	24,342	15,609	-1,560
guaA	18,561	11,908	-1,559
asr2120	1,557	0,000	-1,557
asl3236	1,557	0,000	-1,557
alr4161	9,693	6,251	-1,551
alr0175	9,375	6,046	-1,551
alr3338	5,708	3,681	-1,551
all0387	7,940	5,121	-1,551
alr2479	5,385	3,473	-1,551
all4874	1,550	0,000	-1,550
all4988	1,550	0,000	-1,550
all2705	41,720	26,924	-1,550
all5090	27,937	18,067	-1,546

cpcG2	36,723	23,749	-1,546
all2198	1,543	0,000	-1,543
alr3959	1,543	0,000	-1,543
pecA	540,109	350,048	-1,543
all3076	8,036	5,216	-1,541
alr7346	7,547	4,899	-1,541
all4744	3,233	2,098	-1,541
all7364	15,629	10,145	-1,541
alr2790	15,387	9,988	-1,541
all1768	6,145	3,989	-1,541
all4384	4,034	2,619	-1,541
all1688	2,932	1,903	-1,541
all2003	19,653	12,756	-1,541
ndhF_1	6,413	4,163	-1,541
all2870	3,092	2,007	-1,541
rbcL	202,190	131,580	-1,537
asl0572	1,536	0,000	-1,536
alr2325	1,536	0,000	-1,536
alr2407	1,536	0,000	-1,536
asr2666	1,536	0,000	-1,536
asr7154	1,536	0,000	-1,536
all4037	21,146	13,768	-1,536
alr2272	13,384	8,730	-1,533
psaE	134,851	88,140	-1,530
alr1554	7,861	5,138	-1,530
atpA	31,090	20,330	-1,529
ycf27	25,466	16,664	-1,528
alr3344	31,844	20,847	-1,528
all2457	108,320	70,962	-1,526
all7170	1,526	0,000	-1,526
asr1156	19,734	12,962	-1,522
alr3203	8,596	5,646	-1,522
alr4814	8,596	5,646	-1,522
all4367	7,413	4,869	-1,522
alr5061	4,208	2,764	-1,522
alr3911	2,034	1,336	-1,522
alr1160	13,873	9,112	-1,522
all7160	11,485	7,543	-1,522
all3447	7,149	4,695	-1,522
pyrE	6,769	4,446	-1,522
all1745	6,369	4,183	-1,522
ndhD_3	5,327	3,499	-1,522

fbp	4,003	2,629	-1,522
all4665	3,839	2,521	-1,522
hisS	3,026	1,988	-1,522
infC_1	102,267	67,276	-1,520
cpcD	113,878	74,986	-1,519
alr0297	1,516	0,000	-1,516
asl0977	1,516	0,000	-1,516
alr1877	1,516	0,000	-1,516
asr2958	1,516	0,000	-1,516
alr5216	11,333	7,479	-1,515
alr4124	32,449	21,422	-1,515
alr4174	15,945	10,541	-1,513
alr7163	14,790	9,816	-1,507
rplM	14,595	9,687	-1,507
alr7232	3,792	2,517	-1,507
all4160	6,302	4,183	-1,507
alr7250	1,507	0,000	-1,507
thiC	30,847	20,495	-1,505
alr4330	11,941	7,948	-1,502
alr2264	1,502	0,000	-1,502
alr2503	18,974	12,654	-1,499
pbpB	3,974	2,650	-1,499
all3194	1,497	0,000	-1,497
alr0658	8,340	5,577	-1,495
secD	8,112	5,425	-1,495
alr3092	2,985	1,996	-1,495
alr3980	21,769	14,558	-1,495
all1695	2,657	1,777	-1,495
rpmE	58,380	39,112	-1,493
alr3146	7,252	4,859	-1,493
sigD	14,025	9,396	-1,493
ccmN	22,039	14,784	-1,491
all3391	1,487	0,000	-1,487
alr4914	1,487	0,000	-1,487
all4436	1,486	0,000	-1,486
rpsO	18,163	12,270	-1,480
asl4547	17,963	12,136	-1,480
all4801	17,483	11,811	-1,480
rplF	13,399	9,052	-1,480
alr2475	10,898	7,362	-1,480
rpmC	10,898	7,362	-1,480
alr3737	4,191	2,832	-1,480

all2344	1,858	1,255	-1,480
alr1192	1,728	1,167	-1,480
avaIM	1,692	1,143	-1,480
rpsD	36,236	24,481	-1,480
all1732	13,737	9,280	-1,480
asr5262	13,622	9,203	-1,480
alr1093	8,718	5,890	-1,480
all1660	8,092	5,467	-1,480
all1769	7,046	4,760	-1,480
alr1850	6,645	4,489	-1,480
all2930	6,645	4,489	-1,480
nuiA	6,010	4,060	-1,480
alr3340	5,173	3,495	-1,480
all0186	4,836	3,267	-1,480
all2497	4,406	2,977	-1,480
all0457	3,784	2,556	-1,480
ftsE	3,732	2,521	-1,480
surE_1	3,601	2,432	-1,480
all7309	3,205	2,165	-1,480
natA	3,132	2,116	-1,480
all2124	2,912	1,967	-1,480
all4221	2,838	1,917	-1,480
alr4054	2,809	1,897	-1,480
alr2306	2,671	1,804	-1,480
all0879	2,484	1,678	-1,480
alr3240	2,362	1,596	-1,480
alr1250	2,252	1,521	-1,480
natC	2,162	1,461	-1,480
alr5035	1,870	1,264	-1,480
all3780	1,635	1,104	-1,480
alr0499	1,519	1,026	-1,480
all0364	0,907	0,613	-1,480
ppnK_2	8,013	5,413	-1,480
all0770	5,140	3,473	-1,480
all3323	4,865	3,287	-1,480
all4615	4,279	2,891	-1,480
all2020	2,573	1,738	-1,480
alr1331	2,570	1,736	-1,480
alr1564	2,224	1,503	-1,480
alr4879	2,003	1,353	-1,480
alr7242	1,845	1,246	-1,480
asr1307	1,478	0,000	-1,478

asl2182	1,478	0,000	-1,478
asr2427	1,478	0,000	-1,478
all5169	1,478	0,000	-1,478
all7269	1,469	0,000	-1,469
ileS	12,636	8,619	-1,466
asr1667	1,460	0,000	-1,460
all2609	1,460	0,000	-1,460
hetP_1	1,460	0,000	-1,460
all4327	6,868	4,707	-1,459
pheT	3,311	2,270	-1,459
aspS	4,506	3,088	-1,459
alr7318	3,090	2,118	-1,459
all0040	8,911	6,123	-1,455
alr1805	22,298	15,338	-1,454
clpB_3	98,413	67,707	-1,454
all2089	1,450	0,000	-1,450
all5369	1,450	0,000	-1,450
ftrV	25,246	17,411	-1,450
all1981	17,192	11,856	-1,450
alr3302	7,916	5,459	-1,450
all1751	5,766	3,977	-1,450
alr0300	5,544	3,823	-1,450
alr1376	4,647	3,205	-1,450
all1763	13,297	9,170	-1,450
alr2114	3,782	2,608	-1,450
apcE	99,963	69,042	-1,448
alr2780	12,973	8,973	-1,446
all3227	36,267	25,099	-1,445
rps11	36,267	25,099	-1,445
all3556	10,498	7,265	-1,445
hemE	52,226	36,182	-1,443
clpP_1	14,239	9,876	-1,442
ndhD_2	5,826	4,041	-1,442
all3401	4,293	2,977	-1,442
tldD	11,890	8,247	-1,442
ycf16	11,099	7,698	-1,442
all1984	1,441	0,000	-1,441
asl3777	1,441	0,000	-1,441
alr3958	32,275	22,446	-1,438
alr3057	12,397	8,635	-1,436
all3678	22,363	15,598	-1,434
cphB	30,850	21,545	-1,432

fus_2	42,000	29,396	-1,429
alr7153	1,427	0,000	-1,427
rpmF	18,118	12,694	-1,427
alr4831	6,736	4,719	-1,427
all0567	3,175	2,224	-1,427
alr1081	3,082	2,159	-1,427
alr2335	2,977	2,086	-1,427
all1175	2,386	1,671	-1,427
alr3915	1,684	1,180	-1,427
hetC	1,006	0,705	-1,427
all0262	20,365	14,268	-1,427
all2077	17,227	12,069	-1,427
alr3641	12,079	8,462	-1,427
all0596	9,553	6,693	-1,427
alr4259	6,959	4,876	-1,427
alr0600	6,447	4,517	-1,427
alr4646	6,447	4,517	-1,427
alr1375	6,255	4,382	-1,427
alr8011	5,531	3,875	-1,427
alr1155	5,403	3,785	-1,427
acpD	5,052	3,540	-1,427
alr0248	4,798	3,362	-1,427
all4304	4,520	3,167	-1,427
all0737	4,342	3,042	-1,427
glmM	4,246	2,975	-1,427
alr0180	4,026	2,821	-1,427
all2567	3,970	2,782	-1,427
alr3703	3,892	2,727	-1,427
alr4621	3,863	2,707	-1,427
alr1687	3,562	2,496	-1,427
alr7076	3,064	2,146	-1,427
all3532	2,994	2,098	-1,427
all1975	2,758	1,932	-1,427
xseA	2,544	1,783	-1,427
alr2298	2,299	1,611	-1,427
all5026	2,127	1,490	-1,427
alr7147	2,060	1,444	-1,427
all1316	2,005	1,405	-1,427
all1068	0,890	0,623	-1,427
all0665	1,650	1,156	-1,427
ftsK	1,447	1,014	-1,427
all1780	1,424	0,000	-1,424

alr2587	1,424	0,000	-1,424
all0638	1,422	0,000	-1,422
all3792	41,247	29,009	-1,422
ilvG	27,668	19,482	-1,420
prk	59,252	41,756	-1,419
sigE	43,785	30,863	-1,419
alr7211	1,418	0,000	-1,418
cyaC	3,838	2,707	-1,418
all3024	1,415	0,000	-1,415
alr2309	13,891	9,838	-1,412
all1648	1,471	1,043	-1,410
alr3176	14,874	10,551	-1,410
alr0489	7,836	5,559	-1,410
alr1364	6,578	4,666	-1,410
prfA	12,726	9,027	-1,410
atp1	86,384	61,352	-1,408
hupC	1,407	0,000	-1,407
all0777	1,407	0,000	-1,407
all4445	1,407	0,000	-1,407
asr4747	1,407	0,000	-1,407
alr5027	1,407	0,000	-1,407
alr3442	4,180	2,976	-1,405
all3295	62,949	44,814	-1,405
alr1604	14,595	10,390	-1,405
all3939	11,454	8,154	-1,405
all0174	8,079	5,752	-1,405
ftsH_3	12,973	9,245	-1,403
alr0577	29,542	21,067	-1,402
all1272	7,238	5,167	-1,401
all4035	5,276	3,766	-1,401
alr1222	29,124	20,868	-1,396
alr3281	19,075	13,668	-1,396
alr4308	9,657	6,919	-1,396
zwf	5,037	3,609	-1,396
alr0981	4,538	3,252	-1,396
alr3965	4,491	3,218	-1,396
alr3497	3,638	2,607	-1,396
all2396	2,823	2,023	-1,396
alr4703	2,277	1,632	-1,396
alr1860	10,703	7,669	-1,396
all5046	9,514	6,817	-1,396
alr0264	8,450	6,055	-1,396

alr1966	7,298	5,229	-1,396
all2349	7,149	5,122	-1,396
alr3919	4,225	3,027	-1,396
all5222	1,796	1,287	-1,396
alr4273	1,319	0,945	-1,396
alr1199	1,396	0,000	-1,396
amt1	36,112	25,932	-1,393
asr0358	1,390	0,000	-1,390
alr1013	1,390	0,000	-1,390
asl1840	1,390	0,000	-1,390
asl2733	1,390	0,000	-1,390
asl8505	1,390	0,000	-1,390
all4607	4,153	2,993	-1,388
ssaA	109,348	78,882	-1,386
alr3817	24,342	17,560	-1,386
chlP	33,852	24,420	-1,386
all3745	1,385	0,000	-1,385
purC	11,391	8,230	-1,384
alr2745	9,158	6,616	-1,384
petE	110,923	80,196	-1,383
all3964	15,454	11,214	-1,378
ccmK_3	59,150	42,983	-1,376
all7008	10,468	7,616	-1,374
all3298	9,199	6,693	-1,374
accB	8,294	6,035	-1,374
alr1028	4,114	2,993	-1,374
all4900	1,745	1,269	-1,374
alr3416	8,774	6,383	-1,374
all4575	7,725	5,620	-1,374
all2461	6,761	4,919	-1,374
all1353	5,364	3,902	-1,374
alr3938	4,928	3,586	-1,374
all1154	4,102	2,985	-1,374
sigF	3,882	2,824	-1,374
all3107	3,739	2,720	-1,374
alr7635	2,013	1,465	-1,374
all0361	1,374	0,000	-1,374
asl1679	1,374	0,000	-1,374
asl2101	1,374	0,000	-1,374
asl4860	1,374	0,000	-1,374
alr7357	1,370	0,000	-1,370
alr4672	10,445	7,645	-1,366

alr3351	8,603	6,297	-1,366
desC_1	6,415	4,719	-1,359
all5210	1,123	0,826	-1,359
alr1374	6,659	4,899	-1,359
all4857	4,423	3,254	-1,359
alr1147	1,551	1,141	-1,359
all1252	1,358	0,000	-1,358
asl1650	1,358	0,000	-1,358
asr5183	1,358	0,000	-1,358
asl7156	1,358	0,000	-1,358
alr1285	9,086	6,704	-1,355
all2303	12,472	9,203	-1,355
chlH_1	43,489	32,130	-1,354
alr8019	9,091	6,717	-1,353
alr1278	4,314	3,188	-1,353
all3820	1,350	0,000	-1,350
alr7513	1,350	0,000	-1,350
cobN	19,445	14,408	-1,350
all4420	13,352	9,904	-1,348
ccdA	8,036	5,961	-1,348
all4793	6,321	4,689	-1,348
alr0965	4,118	3,055	-1,348
alr2137	2,100	1,558	-1,348
alr1254	2,030	1,506	-1,348
alr4017	4,737	3,514	-1,348
all4246	3,532	2,620	-1,348
lrtA	67,110	49,885	-1,345
all3292	16,971	12,627	-1,344
asl2794	1,342	0,000	-1,342
alr7573	1,342	0,000	-1,342
rpmB	28,082	20,969	-1,339
all1237	6,468	4,829	-1,339
all1684	5,574	4,162	-1,339
all0594	2,559	1,911	-1,339
alr3165	1,337	0,000	-1,337
all1980	19,460	14,558	-1,337
alr3663	1,334	0,000	-1,334
alr4178	12,804	9,611	-1,332
cysA	14,466	10,859	-1,332
all3746	5,296	3,975	-1,332
alr3443	3,429	2,574	-1,332
alr3311	1,828	1,373	-1,332

all3346	1,716	1,288	-1,332
all7128	0,795	0,597	-1,332
infB	52,542	39,466	-1,331
alr3300	76,431	57,422	-1,331
alr9005	1,331	0,000	-1,331
gap2	38,345	28,861	-1,329
asl1789	1,327	0,000	-1,327
alr5021	11,575	8,727	-1,326
alr1593	21,391	16,158	-1,324
rpsH	21,784	16,483	-1,322
rpsE	16,680	12,621	-1,322
alr1675	13,090	9,904	-1,322
alr2355	8,019	6,068	-1,322
alr0308	2,476	1,873	-1,322
alr1121	1,510	1,143	-1,322
sir	13,810	10,461	-1,320
all5091	20,497	15,579	-1,316
alr0110	18,843	14,321	-1,316
all4545	18,503	14,081	-1,314
gmk	16,930	12,884	-1,314
por	41,043	31,234	-1,314
asl1165	1,312	0,000	-1,312
all3713	1,312	0,000	-1,312
asr8524	1,312	0,000	-1,312
dnaK_4	5,253	4,007	-1,311
alr4176	14,114	10,787	-1,308
ndhG	20,131	15,414	-1,306
alr4058	7,164	5,494	-1,304
ccmK_1	18,118	13,914	-1,302
cpcE	313,189	240,537	-1,302
gyrB	14,460	11,112	-1,301
all1549	6,366	4,895	-1,300
psbV	30,614	23,568	-1,299
all3651	10,041	7,730	-1,299
all3895	9,138	7,042	-1,298
all2754	1,297	0,000	-1,297
all7226	1,297	0,000	-1,297
alr9011	1,297	0,000	-1,297
alr5283	24,661	19,040	-1,295
alr0990	11,466	8,852	-1,295
alr4057	10,650	8,241	-1,292
leuA_2	12,949	10,033	-1,291

alr4267	8,153	6,317	-1,291
all3291	1,290	0,000	-1,290
cpcA	432,870	336,690	-1,286
apcB	376,787	293,588	-1,283
asl8539	1,283	0,000	-1,283
alr3543	19,144	14,940	-1,281
chlB	47,714	37,245	-1,281
trpD_1	1,280	0,000	-1,280
alr2998	1,276	0,000	-1,276
asl3262	22,894	18,045	-1,269
asl3888	16,983	13,386	-1,269
all0646	16,965	13,372	-1,269
pbsY	16,680	13,147	-1,269
all4894	16,445	12,962	-1,269
chlI	13,700	10,798	-1,269
alr4132	12,020	9,474	-1,269
alr0784	11,120	8,765	-1,269
tatA	10,265	8,090	-1,269
all1009	9,267	7,304	-1,269
alr5059	8,860	6,983	-1,269
asl0003	7,413	5,843	-1,269
alr3725	6,487	5,113	-1,269
all3940	6,442	5,077	-1,269
all1762	6,248	4,925	-1,269
all4403	6,135	4,835	-1,269
all3179	6,099	4,807	-1,269
rplR	5,790	4,563	-1,269
all3869	5,575	4,394	-1,269
alr3798	5,307	4,183	-1,269
alr3454	5,283	4,164	-1,269
alr1004	4,891	3,855	-1,269
eno	4,888	3,852	-1,269
all0362	4,272	3,367	-1,269
alr3018	4,097	3,229	-1,269
all3635	4,076	3,213	-1,269
all3913	3,971	3,130	-1,269
asl0163	3,958	3,120	-1,269
asr3783	3,892	3,068	-1,269
all4382	3,742	2,950	-1,269
all0470	3,707	2,922	-1,269
alr0552	3,687	2,906	-1,269
alr7337	3,593	2,832	-1,269

alr9504	3,583	2,824	-1,269
asl3383	3,538	2,789	-1,269
all0844	3,434	2,707	-1,269
asl3438	3,384	2,667	-1,269
chlG	3,384	2,667	-1,269
asr0757	3,336	2,629	-1,269
asl1421	3,289	2,592	-1,269
asl4903	3,243	2,556	-1,269
all2992	3,221	2,539	-1,269
alr0613	2,994	2,360	-1,269
ndhD_5	2,914	2,297	-1,269
all0927	2,857	2,251	-1,269
all3044	2,831	2,231	-1,269
tynA	2,826	2,228	-1,269
all1766	2,797	2,204	-1,269
all1875	2,786	2,196	-1,269
asr1559	2,780	2,191	-1,269
alr1260	2,654	2,092	-1,269
xseB	2,624	2,068	-1,269
alr0480	2,548	2,008	-1,269
asl1394	2,538	2,001	-1,269
asl4654	2,538	2,001	-1,269
asl4565	2,511	1,979	-1,269
all0415	2,506	1,975	-1,269
alr4009	2,502	1,972	-1,269
asr1734	2,484	1,958	-1,269
alr1721	2,445	1,927	-1,269
all2700	2,359	1,859	-1,269
asl4507	2,359	1,859	-1,269
alr0511	2,347	1,850	-1,269
all2423	2,335	1,841	-1,269
alr4864	2,335	1,841	-1,269
all0314	2,312	1,822	-1,269
all0783	2,260	1,781	-1,269
alr1026	2,235	1,761	-1,269
all4981	2,166	1,707	-1,269
alr3917	2,085	1,643	-1,269
alr1349	1,979	1,560	-1,269
alr2746	1,979	1,560	-1,269
alr3015	1,979	1,560	-1,269
all1471	1,954	1,540	-1,269
all1597	1,946	1,534	-1,269

all1151	1,938	1,527	-1,269
all3924	1,928	1,519	-1,269
alr7295	1,901	1,498	-1,269
alr3103	1,893	1,492	-1,269
all7031	1,853	1,461	-1,269
xisF	1,814	1,430	-1,269
all1829	1,769	1,394	-1,269
all3991	1,769	1,394	-1,269
alr7307	1,769	1,394	-1,269
all1059	1,738	1,370	-1,269
alr3052	1,736	1,368	-1,269
alr4435	1,692	1,334	-1,269
all3167	1,664	1,312	-1,269
all1022	1,633	1,287	-1,269
all7109	1,622	1,278	-1,269
all1391	1,610	1,269	-1,269
all7332	1,610	1,269	-1,269
all2068	1,497	1,180	-1,269
alr1337	1,487	1,172	-1,269
all4097	1,450	1,143	-1,269
alr5326	1,450	1,143	-1,269
all7236	1,390	1,096	-1,269
all1820	1,387	1,093	-1,269
alr0074	1,378	1,086	-1,269
alr1727	1,366	1,076	-1,269
all1473	1,327	1,046	-1,269
alr0568	1,312	1,034	-1,269
alr0508	1,242	0,979	-1,269
all3132	1,223	0,964	-1,269
all1523	1,207	0,951	-1,269
all4825	1,204	0,949	-1,269
alr7096	1,204	0,949	-1,269
alr7068	1,168	0,920	-1,269
all3131	1,150	0,907	-1,269
all0071	1,123	0,885	-1,269
alr1712	1,094	0,862	-1,269
alr1999	1,076	0,848	-1,269
alr0447	1,052	0,829	-1,269
all3084	1,047	0,825	-1,269
all7521	1,029	0,811	-1,269
all4998	1,024	0,807	-1,269
alr5095	1,015	0,800	-1,269

all0886	1,009	0,795	-1,269
all1882	0,998	0,787	-1,269
all0032	0,989	0,780	-1,269
all0325	0,953	0,751	-1,269
all0327	0,898	0,708	-1,269
alr4597	0,885	0,697	-1,269
alr2104	0,881	0,695	-1,269
all3630	0,868	0,684	-1,269
ksgA	0,859	0,677	-1,269
all0061	0,840	0,662	-1,269
alr7336	0,811	0,639	-1,269
all0419	0,768	0,605	-1,269
alr0587	0,748	0,590	-1,269
alr3756	0,745	0,587	-1,269
alr4915	0,744	0,586	-1,269
alr3068	0,737	0,581	-1,269
all7011	0,721	0,568	-1,269
all2688	0,710	0,559	-1,269
alr3020	0,706	0,556	-1,269
all3270	0,699	0,551	-1,269
all5345	0,699	0,551	-1,269
alr1583	0,697	0,549	-1,269
all7592	0,644	0,508	-1,269
cyaA	0,635	0,500	-1,269
all3764	0,631	0,497	-1,269
all2477	0,602	0,474	-1,269
all0808	0,600	0,473	-1,269
all5131	0,579	0,457	-1,269
all4832	0,543	0,428	-1,269
alr5236	0,513	0,405	-1,269
alr7647	0,495	0,390	-1,269
cls	0,485	0,383	-1,269
all1186	0,480	0,379	-1,269
alr8530	0,405	0,320	-1,269
all0323	0,387	0,305	-1,269
alr5331	0,285	0,225	-1,269
all3465	0,209	0,164	-1,269
rplQ	21,955	17,305	-1,269
all1542	15,066	11,875	-1,269
asr0855	13,900	10,956	-1,269
alr4661	13,810	10,885	-1,269
alr1892	12,854	10,132	-1,269

asr4648	11,824	9,319	-1,269
alr4100	11,391	8,978	-1,269
rpmH	10,379	8,180	-1,269
trpG	8,836	6,964	-1,269
rbfA	8,714	6,868	-1,269
all3600	8,714	6,868	-1,269
asl4262	8,649	6,817	-1,269
all5176	8,585	6,767	-1,269
recR	8,570	6,754	-1,269
alr1957	8,400	6,621	-1,269
crtQ	8,271	6,519	-1,269
all0298	7,473	5,890	-1,269
all0754	7,298	5,752	-1,269
alr2373	6,311	4,975	-1,269
all2611	6,269	4,941	-1,269
asl3868	5,912	4,660	-1,269
all4804	5,875	4,630	-1,269
alr2083	5,161	4,068	-1,269
fdxB	4,766	3,756	-1,269
alr0252	4,448	3,506	-1,269
alr2925	4,351	3,430	-1,269
alr5068	4,324	3,408	-1,269
all4341	4,133	3,258	-1,269
alr1979	4,050	3,192	-1,269
all3191	4,003	3,155	-1,269
alr1334	3,807	3,001	-1,269
prmA	3,803	2,998	-1,269
alr2376	3,460	2,727	-1,269
nrtA_2	3,274	2,580	-1,269
asr3390	3,199	2,521	-1,269
alr4439	3,177	2,504	-1,269
alr4701	3,156	2,487	-1,269
all5042	3,135	2,471	-1,269
all4789	3,121	2,460	-1,269
asr2468	3,033	2,390	-1,269
alr4129	2,979	2,348	-1,269
asl2197	2,956	2,330	-1,269
all4070	2,956	2,330	-1,269
all1771	2,931	2,310	-1,269
alr7203	2,919	2,301	-1,269
asl0027	2,848	2,245	-1,269
alr0359	2,814	2,218	-1,269

alr4067	2,814	2,218	-1,269
argS	2,775	2,187	-1,269
all4440	2,700	2,128	-1,269
asl4353	2,684	2,116	-1,269
all8564	2,609	2,057	-1,269
alr2773	2,458	1,937	-1,269
alr0279	2,395	1,888	-1,269
all4102	2,383	1,878	-1,269
all1075	2,375	1,872	-1,269
alr5270	2,219	1,749	-1,269
all1719	2,203	1,736	-1,269
all0736	2,142	1,689	-1,269
all5264	2,113	1,666	-1,269
alr3806	2,071	1,632	-1,269
all5022	1,971	1,553	-1,269
alr8563	1,962	1,547	-1,269
alr3066	1,858	1,465	-1,269
all0469	1,845	1,454	-1,269
all3446	1,824	1,438	-1,269
alr0277	1,783	1,405	-1,269
all1806	1,783	1,405	-1,269
alr2132	1,783	1,405	-1,269
all4128	1,766	1,392	-1,269
all4651	1,756	1,384	-1,269
all4478	1,656	1,305	-1,269
all2622	1,627	1,283	-1,269
mazF	1,589	1,252	-1,269
alr4158	1,581	1,246	-1,269
alr3158	1,567	1,235	-1,269
all3022	1,516	1,195	-1,269
all7597	1,478	1,165	-1,269
all1737	1,446	1,140	-1,269
alr1350	1,297	1,023	-1,269
hoxF	1,285	1,013	-1,269
alr1086	1,232	0,971	-1,269
alr1874	1,216	0,959	-1,269
alr1302	1,210	0,954	-1,269
all1932	1,194	0,941	-1,269
alr7350	1,148	0,904	-1,269
alr1852	1,112	0,876	-1,269
all8508	1,104	0,870	-1,269
alr4587	1,091	0,860	-1,269

all4978	1,038	0,818	-1,269
all2912	0,942	0,742	-1,269
all0902	0,938	0,739	-1,269
all3198	0,938	0,739	-1,269
all3011	0,902	0,711	-1,269
all3853	0,902	0,711	-1,269
alr2838	0,891	0,703	-1,269
alr4631	0,875	0,689	-1,269
trpC_2	0,828	0,653	-1,269
all0903	0,814	0,641	-1,269
dmnB	0,794	0,626	-1,269
all2801	0,789	0,622	-1,269
alr2987	0,758	0,598	-1,269
all3047	0,741	0,584	-1,269
alr4629	0,739	0,582	-1,269
all0910	0,679	0,535	-1,269
all7098	0,629	0,496	-1,269
all4423	0,615	0,484	-1,269
alr1448	0,607	0,478	-1,269
all7682	0,597	0,471	-1,269
all3244	0,591	0,466	-1,269
hisZ	0,577	0,454	-1,269
all2950	0,577	0,454	-1,269
alr5319	0,519	0,409	-1,269
all0824	0,466	0,367	-1,269
all7191	0,439	0,346	-1,269
all4413	0,314	0,248	-1,269
hglE_1	0,185	0,146	-1,269
all3289	0,181	0,143	-1,269
all0261	1,265	0,000	-1,265
all4735	1,262	0,000	-1,262
alr7125	1,262	0,000	-1,262
alr7202	1,262	0,000	-1,262
psaC	229,250	181,813	-1,261
atpB	65,270	51,826	-1,259
all3697	1,255	0,000	-1,255
asr5312	1,255	0,000	-1,255
mraY	28,086	22,385	-1,255
psbO	60,511	48,365	-1,251
alr2308	18,364	14,690	-1,250
all0860	15,782	12,650	-1,248
all4464	25,572	20,535	-1,245

all1267	6,524	5,247	-1,243
asl0628	1,242	0,000	-1,242
asr3878	1,242	0,000	-1,242
alr0188	14,636	11,792	-1,241
all0478	2,938	2,369	-1,240
cytA	40,658	32,867	-1,237
trxA	42,164	34,085	-1,237
pecB	486,617	393,649	-1,236
all2480	1,233	0,000	-1,233
ccmK_2	40,780	33,157	-1,230
all7564	1,229	0,000	-1,229
rpoC2	15,384	12,534	-1,227
nusA	7,949	6,481	-1,226
alr4380	20,710	16,886	-1,226
all4110	1,226	0,000	-1,226
all3563	5,036	4,116	-1,223
hisD_1	13,990	11,451	-1,222
all5258	13,268	10,876	-1,220
alr2759	7,409	6,073	-1,220
alr2326	5,366	4,398	-1,220
all3986	56,787	46,585	-1,219
alr5257	13,942	11,446	-1,218
alr0124	5,004	4,108	-1,218
asl1664	1,216	0,000	-1,216
asr7023	1,216	0,000	-1,216
all0333	22,953	18,878	-1,216
all4162	12,608	10,369	-1,216
alr4566	1,213	0,000	-1,213
alr1223	33,847	27,931	-1,212
asl3981	68,111	56,240	-1,211
pyrG	4,491	3,708	-1,211
alr0103	3,144	2,596	-1,211
all8519	2,929	2,419	-1,211
all3558	2,912	2,405	-1,211
all7618	2,315	1,912	-1,211
alr0559	1,210	0,000	-1,210
dmnC	1,210	0,000	-1,210
secA	17,433	14,432	-1,208
all1140	7,065	5,862	-1,205
all4668	3,954	3,281	-1,205
asl0550	30,390	25,213	-1,205
acpP	26,100	21,654	-1,205

alr1566	1,204	0,000	-1,204
alr8556	1,204	0,000	-1,204
all0284	2,455	2,043	-1,202
alr1482	5,251	4,382	-1,198
dnaJ_5	5,943	4,960	-1,198
alr3867	4,269	3,562	-1,198
alr0145	13,233	11,043	-1,198
all0111	18,758	15,690	-1,196
all0909	1,196	0,000	-1,196
alr7640	1,196	0,000	-1,196
all2707	9,353	7,832	-1,194
all2342	21,188	17,766	-1,193
all0462	2,999	2,514	-1,193
alr7228	53,890	45,215	-1,192
alr7019	1,191	0,000	-1,191
all7150	1,191	0,000	-1,191
cpcF	34,854	29,303	-1,189
all2193	8,670	7,289	-1,189
alr2076	6,487	5,454	-1,189
alr0140	6,334	5,325	-1,189
all3118	4,220	3,548	-1,189
all5280	2,272	1,910	-1,189
all1893	16,523	13,891	-1,189
petC_3	37,623	31,699	-1,187
alr1159	14,943	12,599	-1,186
all2651	1,185	0,000	-1,185
chlL	45,250	38,213	-1,184
alr2708	9,476	8,002	-1,184
alr3376	5,838	4,930	-1,184
alr0092	7,623	6,448	-1,182
alr4571	14,362	12,159	-1,181
asr3308	1,179	0,000	-1,179
asl4244	1,179	0,000	-1,179
asl5170	1,179	0,000	-1,179
all8565	1,179	0,000	-1,179
all3680	21,809	18,512	-1,178
all3326	7,589	6,442	-1,178
zam	23,233	19,720	-1,178
asr3294	16,499	14,004	-1,178
aroE_2	5,364	4,553	-1,178
alr7325	3,757	3,189	-1,178
all2654	1,070	0,909	-1,178

all3282	9,730	8,259	-1,178
cpcB	432,821	368,115	-1,176
atpD	23,479	20,006	-1,174
alr5254	1,172	0,000	-1,172
alr4056	50,040	42,728	-1,171
alr0099	17,139	14,635	-1,171
all0144	9,279	7,923	-1,171
hlyB	2,871	2,452	-1,171
clpB_2	3,181	2,716	-1,171
alr7212	1,171	0,000	-1,171
all3143	9,160	7,829	-1,170
all0292	1,168	0,000	-1,168
alr5181	1,168	0,000	-1,168
all7627	1,168	0,000	-1,168
alr1525	109,738	94,104	-1,166
rplE	21,055	18,104	-1,163
smtB	9,375	8,061	-1,163
alr2054	4,014	3,451	-1,163
all3149	4,014	3,451	-1,163
all2343	14,011	12,047	-1,163
alr0116	6,587	5,663	-1,163
alr4714	5,512	4,740	-1,163
alr8535	5,397	4,640	-1,163
alr3824	4,810	4,136	-1,163
alr1052	4,384	3,769	-1,163
alr4907	4,184	3,597	-1,163
alr5279	3,734	3,210	-1,163
all0797	3,538	3,042	-1,163
aroB_2	3,528	3,034	-1,163
aroA	3,015	2,592	-1,163
all1678	2,579	2,218	-1,163
all3313	2,289	1,969	-1,163
all3516	1,740	1,496	-1,163
alr0735	1,162	0,000	-1,162
apcA_1	441,818	380,612	-1,161
all1351	48,261	41,720	-1,157
ureA	1,156	0,000	-1,156
all5337	1,156	0,000	-1,156
alr3115	2,319	2,011	-1,153
asnC	5,033	4,363	-1,153
alr0849	4,561	3,954	-1,153
all4248	2,757	2,390	-1,153

pecE	40,912	35,507	-1,152
all2379	2,518	2,190	-1,150
all7158	13,450	11,729	-1,147
alr4579	7,249	6,322	-1,147
mutS	6,868	5,989	-1,147
alr4745	11,529	10,054	-1,147
alr1744	22,442	19,601	-1,145
alr1571	1,145	0,000	-1,145
asl5128	25,630	22,446	-1,142
alr1746	17,227	15,087	-1,142
asr1494	12,661	11,088	-1,142
all0048	10,404	9,112	-1,142
all2288	9,164	8,026	-1,142
all2977	8,407	7,362	-1,142
alr3172	7,842	6,868	-1,142
alr1906	7,823	6,851	-1,142
alr3339	7,231	6,332	-1,142
alr4373	6,913	6,055	-1,142
alr0517	6,447	5,646	-1,142
dnaJ_2	5,575	4,882	-1,142
all2849	5,307	4,648	-1,142
uvrB	4,734	4,145	-1,142
all2917	3,878	3,396	-1,142
mraW	3,468	3,037	-1,142
alr0309	2,994	2,622	-1,142
alr3210	2,614	2,289	-1,142
alr1096	2,496	2,186	-1,142
alr3473	1,352	1,184	-1,142
alr1137	9,730	8,521	-1,142
alr4288	9,641	8,443	-1,142
alr0507	5,321	4,660	-1,142
rnhB	4,650	4,072	-1,142
all0589	3,128	2,739	-1,142
all2460	2,461	2,155	-1,142
all1872	87,339	76,691	-1,139
nifK	1,138	0,000	-1,138
all1784	1,137	0,000	-1,137
glnA	33,922	29,837	-1,137
alr0101	38,770	34,111	-1,137
all0772	1,134	0,000	-1,134
all0868	1,134	0,000	-1,134
hepK	5,068	4,474	-1,133

all5250	8,064	7,118	-1,133
rpoB	24,033	21,300	-1,128
all3962	1,128	0,000	-1,128
ndhF_3	1,128	0,000	-1,128
all7351	1,128	0,000	-1,128
all7255	7,473	6,626	-1,128
asl3112	22,783	20,201	-1,128
all1761	14,886	13,199	-1,128
ntcA	8,340	7,395	-1,128
cbiM_1	8,158	7,234	-1,128
secF	5,731	5,081	-1,128
alr1605	4,852	4,303	-1,128
alr3871	3,714	3,293	-1,128
alr7649	0,724	0,642	-1,128
alr7231	59,974	53,436	-1,122
atpC_2	19,460	17,339	-1,122
all3971	14,715	13,111	-1,122
rplU	26,739	23,885	-1,119
patN	16,217	14,486	-1,119
all7229	90,713	81,043	-1,119
all3459	18,407	16,457	-1,118
alr8077	12,204	10,919	-1,118
all8024	33,434	30,001	-1,114
gltS	10,778	9,675	-1,114
all4415	1,112	0,000	-1,112
all4519	1,112	0,000	-1,112
all0216	21,893	19,720	-1,110
all2062	13,971	12,585	-1,110
all3748	6,169	5,556	-1,110
alr3640	4,541	4,090	-1,110
all5244	3,132	2,821	-1,110
all2644	0,312	0,281	-1,110
asl7159	8,426	7,590	-1,110
rps1_2	8,013	7,218	-1,110
alr2494	7,201	6,487	-1,110
alr3818	6,361	5,729	-1,110
all4633	6,192	5,577	-1,110
alr4800	6,099	5,494	-1,110
all4673	5,030	4,531	-1,110
alr4216	5,014	4,517	-1,110
alr0198	4,324	3,895	-1,110
all2716	4,170	3,756	-1,110

alr0731	3,929	3,540	-1,110
all1617	3,528	3,178	-1,110
all1007	3,398	3,061	-1,110
alr1271	3,309	2,981	-1,110
alr1259	3,120	2,810	-1,110
alr3285	2,654	2,390	-1,110
obgE	2,383	2,146	-1,110
hupD	2,128	1,917	-1,110
alr3159	2,117	1,907	-1,110
alr1998	1,792	1,615	-1,110
all7631	1,569	1,413	-1,110
all0453	1,421	1,280	-1,110
all7133	1,156	1,041	-1,110
trpE_2	1,142	1,028	-1,110
alr4346	1,005	0,906	-1,110
alr2596	0,996	0,897	-1,110
all3245	0,821	0,739	-1,110
all5040	3,016	2,717	-1,110
all0456	1,115	1,004	-1,110
all4771	1,107	0,000	-1,107
all7148	1,107	0,000	-1,107
engA	10,287	9,324	-1,103
clpX	19,111	17,333	-1,103
rpsL	30,102	27,321	-1,102
orrA	15,988	14,511	-1,102
all0962	1,102	0,000	-1,102
all7658	1,102	0,000	-1,102
pepC	6,708	6,093	-1,101
ndhI	15,568	14,158	-1,100
all1303	6,445	5,862	-1,100
groEL_2	15,211	13,847	-1,099
all4118	12,325	11,248	-1,096
asr3467	25,210	23,007	-1,096
all1445	1,091	0,000	-1,091
alr3312	1,091	0,000	-1,091
alr3583	1,091	0,000	-1,091
all3327	33,274	30,517	-1,090
alr4839	8,868	8,154	-1,087
alr2738	8,029	7,383	-1,087
hrcA	7,656	7,040	-1,087
txlA	7,260	6,676	-1,087
all1225	4,434	4,077	-1,087

alr4943	4,145	3,812	-1,087
dnaN	3,611	3,321	-1,087
alr3370	3,258	2,996	-1,087
all1089	3,121	2,869	-1,087
all3087	2,580	2,373	-1,087
all4225	2,475	2,276	-1,087
glyS	1,954	1,797	-1,087
all3193	8,052	7,405	-1,087
all4690	1,086	0,000	-1,086
carB	8,136	7,496	-1,085
uvsE	1,084	0,000	-1,084
all4254	8,486	7,842	-1,082
all1273	1,081	0,000	-1,081
all1816	1,081	0,000	-1,081
alr2972	1,081	0,000	-1,081
alr7097	1,081	0,000	-1,081
alr7103	1,081	0,000	-1,081
petH	21,181	19,616	-1,080
all1682	4,260	3,950	-1,078
asr3935	27,039	25,187	-1,074
gatB	10,442	9,727	-1,074
all3315	1,923	1,791	-1,074
all4834	1,655	1,542	-1,074
alr4293	15,663	14,590	-1,074
alr2191	8,395	7,819	-1,074
all1748	1,071	0,000	-1,071
alr2215	1,071	0,000	-1,071
alr2488	1,071	0,000	-1,071
ureB	1,071	0,000	-1,071
alr4224	1,071	0,000	-1,071
alr7292	1,071	0,000	-1,071
all3933	4,249	3,969	-1,071
all8083	129,032	120,751	-1,069
alr3816	15,313	14,332	-1,068
alr4104	61,423	57,546	-1,067
alr1520	1,061	0,000	-1,061
alr3063	1,058	0,000	-1,058
glgA_2	11,108	10,506	-1,057
alr4094	5,696	5,387	-1,057
all9031	4,597	4,348	-1,057
all3051	4,261	4,030	-1,057
all5011	3,637	3,440	-1,057

ruvC	3,560	3,367	-1,057
all0376	3,208	3,034	-1,057
rfbC_2	3,208	3,034	-1,057
lysS	3,116	2,948	-1,057
all4350	2,684	2,539	-1,057
alr3201	2,549	2,411	-1,057
alr4521	2,298	2,174	-1,057
alr2683	1,959	1,853	-1,057
alr4099	1,959	1,853	-1,057
all2302	1,493	1,412	-1,057
alr3620	1,204	1,138	-1,057
all1811	1,165	1,102	-1,057
all0872	0,943	0,892	-1,057
all2282	0,327	0,310	-1,057
alr0549	34,854	32,965	-1,057
alr0113	25,383	24,007	-1,057
alr1224	12,100	11,444	-1,057
alr0834	10,262	9,706	-1,057
alr2887	9,429	8,918	-1,057
all5013	7,889	7,462	-1,057
ilvH	6,380	6,035	-1,057
alr3804	6,081	5,752	-1,057
all3410	5,668	5,361	-1,057
alr1352	5,508	5,209	-1,057
all4929	5,300	5,013	-1,057
all3124	5,213	4,930	-1,057
alr4715	4,998	4,727	-1,057
alr1669	4,927	4,660	-1,057
alr1544	4,746	4,489	-1,057
alr4326	4,746	4,489	-1,057
all7630	4,357	4,121	-1,057
all3186	4,324	4,090	-1,057
all1741	4,045	3,826	-1,057
all2623	3,978	3,763	-1,057
hslO	3,866	3,657	-1,057
alr2312	3,626	3,430	-1,057
alr1246	3,355	3,173	-1,057
all4397	3,190	3,017	-1,057
all1823	3,181	3,009	-1,057
alr3445	3,139	2,969	-1,057
exoD	2,767	2,617	-1,057
all3564	2,527	2,390	-1,057

alr3258	2,516	2,380	-1,057
all1219	2,373	2,245	-1,057
all5024	2,308	2,182	-1,057
alr1968	2,304	2,180	-1,057
all4089	2,254	2,132	-1,057
alr2615	2,211	2,092	-1,057
all0178	2,013	1,904	-1,057
alr2785	1,796	1,699	-1,057
alr0304	1,785	1,689	-1,057
all4725	1,732	1,638	-1,057
all7085	1,557	1,472	-1,057
all0809	1,434	1,357	-1,057
alr4168	1,377	1,302	-1,057
alr4360	1,364	1,290	-1,057
all7601	1,318	1,246	-1,057
menE	1,206	1,141	-1,057
all0848	1,114	1,054	-1,057
all1914	1,039	0,983	-1,057
alr0290	0,889	0,840	-1,057
alr7129	0,491	0,464	-1,057
all3719	1,057	0,000	-1,057
all8010	50,992	48,412	-1,053
all9030	1,052	0,000	-1,052
all5192	1,049	0,000	-1,049
nodM	7,109	6,792	-1,047
alr0652	9,158	8,765	-1,045
all7268	6,565	6,283	-1,045
all9003	2,978	2,850	-1,045
all4312	12,721	12,175	-1,045
alr0237	12,103	11,597	-1,044
tig	11,379	10,919	-1,042
all0185	4,839	4,643	-1,042
alr2428	3,299	3,166	-1,042
all2229	1,039	0,000	-1,039
cpcG4	35,396	34,058	-1,039
all4377	50,340	48,494	-1,038
asl2353	21,229	20,451	-1,038
alr2201	12,219	11,771	-1,038
alr4616	12,010	11,569	-1,038
alr2758	5,151	4,962	-1,038
all4127	3,720	3,583	-1,038
dnaB_1	2,388	2,301	-1,038

alr0710	1,168	1,125	-1,038
all2402	1,033	0,000	-1,033
alr3247	1,033	0,000	-1,033
all0028	22,998	22,310	-1,031
all5273	10,468	10,155	-1,031
alr4354	6,837	6,633	-1,031
all1019	5,500	5,335	-1,031
alr0474	2,543	2,466	-1,031
all3173	52,705	51,127	-1,031
asr2016	15,489	15,025	-1,031
all4780	7,368	7,148	-1,031
all2008	3,640	3,531	-1,031
alr2986	3,426	3,324	-1,031
ruvB	6,681	6,520	-1,025
all4656	5,880	5,738	-1,025
alr0548	5,729	5,591	-1,025
all0441	2,933	2,862	-1,025
alr3900	6,130	5,982	-1,025
all1919	4,483	4,374	-1,025
all2022	1,024	0,000	-1,024
chlN	16,466	16,125	-1,021
all2528	1,020	0,000	-1,020
alr4670	15,874	15,570	-1,020
all3259	38,548	37,868	-1,018
alr1562	1,015	0,000	-1,015
all5025	1,015	0,000	-1,015
alr5134	6,720	6,621	-1,015
alr2449	2,518	2,481	-1,015
all3271	2,504	2,467	-1,015
alr3277	15,568	15,338	-1,015
asr0062	13,538	13,337	-1,015
all1163	12,053	11,875	-1,015
accA	11,426	11,257	-1,015
all7183	8,052	7,934	-1,015
lnt	5,297	5,219	-1,015
all3821	4,365	4,300	-1,015
all3435	4,115	4,054	-1,015
all4675	3,992	3,933	-1,015
all3551	2,616	2,578	-1,015
alr0289	2,359	2,324	-1,015
alr3654	2,026	1,996	-1,015
alr3466	1,835	1,808	-1,015

all0993	0,873	0,860	-1,015
alr7190	0,784	0,772	-1,015
glpX	46,232	45,748	-1,011
hemA	14,697	14,587	-1,008
all3418	6,408	6,360	-1,008
alr7566	1,007	0,000	-1,007
all7584	1,007	0,000	-1,007
alr3330	134,509	133,994	-1,004
rbpF	33,046	32,991	-1,002
alr2771	8,488	8,485	-1,000
alr7007	0,998	0,000	-0,998
alr8017	0,998	0,000	-0,998
alr8566	0,998	0,000	-0,998
all1172	0,977	0,000	-0,977
all1006	0,973	0,000	-0,973
alr3061	0,973	0,000	-0,973
alr7254	0,973	0,000	-0,973
all1071	0,965	0,000	-0,965
all1463	0,965	0,000	-0,965
alr2119	0,957	0,000	-0,957
all4719	0,957	0,000	-0,957
alr1577	0,954	0,000	-0,954
all0220	0,953	0,000	-0,953
alr2142	0,953	0,000	-0,953
alr1718	0,949	0,000	-0,949
alr3004	0,949	0,000	-0,949
all4556	0,944	0,000	-0,944
hesB	0,942	0,000	-0,942
all2721	0,942	0,000	-0,942
alr5290	0,942	0,000	-0,942
alr2484	0,938	0,000	-0,938
all7053	0,934	0,000	-0,934
alr7107	0,934	0,000	-0,934
all0823	0,927	0,000	-0,927
gvpG	0,927	0,000	-0,927
all2789	0,927	0,000	-0,927
all3439	0,927	0,000	-0,927
all3000	0,924	0,000	-0,924
all3306	0,923	0,000	-0,923
all0211	0,919	0,000	-0,919
all3040	0,919	0,000	-0,919
alr4455	0,919	0,000	-0,919

all3206	0,918	0,000	-0,918
all0407	0,912	0,000	-0,912
alr1029	0,912	0,000	-0,912
all7002	0,912	0,000	-0,912
alr7534	0,912	0,000	-0,912
alr0080	0,905	0,000	-0,905
alr4305	0,903	0,000	-0,903
all1087	0,898	0,000	-0,898
all2612	0,898	0,000	-0,898
all7186	0,898	0,000	-0,898
all0908	0,895	0,000	-0,895
all7033	0,895	0,000	-0,895
all7578	0,894	0,000	-0,894
alr0554	0,891	0,000	-0,891
all0907	0,891	0,000	-0,891
all7370	0,891	0,000	-0,891
alr4564	0,889	0,000	-0,889
alr2143	0,885	0,000	-0,885
alr3803	0,885	0,000	-0,885
all0468	0,878	0,000	-0,878
phnC	0,878	0,000	-0,878
alr2863	0,871	0,000	-0,871
all3972	0,871	0,000	-0,871
alr4922	0,871	0,000	-0,871
all7624	0,871	0,000	-0,871
all1996	0,859	0,000	-0,859
alr7528	0,855	0,000	-0,855
nifX	0,846	0,000	-0,846
all3629	0,843	0,000	-0,843
alr2966	0,837	0,000	-0,837
alr7580	0,834	0,000	-0,834
alr4222	0,828	0,000	-0,828
all3153	0,823	0,000	-0,823
all1504	0,822	0,000	-0,822
all8553	0,822	0,000	-0,822
alr5182	0,817	0,000	-0,817
all1388	0,811	0,000	-0,811
all3542	0,811	0,000	-0,811
alr1501	0,805	0,000	-0,805
alr2594	0,805	0,000	-0,805
alr1040	0,800	0,000	-0,800
all5171	0,800	0,000	-0,800

alr2802	0,794	0,000	-0,794
alr3336	0,792	0,000	-0,792
alr4880	0,789	0,000	-0,789
all5132	0,789	0,000	-0,789
alr4323	0,781	0,000	-0,781
alr7043	0,778	0,000	-0,778
all1464	0,773	0,000	-0,773
all4475	0,770	0,000	-0,770
alr0377	0,758	0,000	-0,758
alr0614	0,758	0,000	-0,758
all4467	0,758	0,000	-0,758
all8507	0,753	0,000	-0,753
all0707	0,750	0,000	-0,750
ilvB_1	0,741	0,000	-0,741
alr2840	0,739	0,000	-0,739
all3347	0,739	0,000	-0,739
all4927	0,739	0,000	-0,739
all7166	0,739	0,000	-0,739
all7588	0,739	0,000	-0,739
nifB	0,736	0,000	-0,736
alr3070	0,732	0,000	-0,732
all4923	0,730	0,000	-0,730
alr3475	0,728	0,000	-0,728
alr2212	0,714	0,000	-0,714
all4233	0,713	0,000	-0,713
all2289	0,708	0,000	-0,708
alr4250	0,708	0,000	-0,708
all7368	0,708	0,000	-0,708
all8551	0,708	0,000	-0,708
alr3755	0,706	0,000	-0,706
alr2210	0,706	0,000	-0,706
all1100	0,703	0,000	-0,703
alr2174	0,703	0,000	-0,703
alr2178	0,701	0,000	-0,701
alr1568	0,699	0,000	-0,699
all4710	0,699	0,000	-0,699
alr7138	0,699	0,000	-0,699
all8547	0,699	0,000	-0,699
alr3078	0,697	0,000	-0,697
all1193	0,695	0,000	-0,695
alr2184	0,695	0,000	-0,695
all4389	0,695	0,000	-0,695

gap3	0,691	0,000	-0,691
all0833	0,687	0,000	-0,687
all2149	0,687	0,000	-0,687
all2586	0,679	0,000	-0,679
all7113	0,679	0,000	-0,679
all2670	0,678	0,000	-0,678
all0634	0,671	0,000	-0,671
alr2625	0,671	0,000	-0,671
all7054	0,667	0,000	-0,667
alr3242	0,665	0,000	-0,665
all1189	0,663	0,000	-0,663
all1472	0,663	0,000	-0,663
alr4230	0,660	0,000	-0,660
alr7042	0,660	0,000	-0,660
ccmA_1	0,658	0,000	-0,658
alr1166	0,649	0,000	-0,649
alr1631	0,649	0,000	-0,649
all2007	0,649	0,000	-0,649
all0059	0,642	0,000	-0,642
hoxY	0,642	0,000	-0,642
alr7184	0,642	0,000	-0,642
all0615	0,635	0,000	-0,635
all4697	0,635	0,000	-0,635
all5130	0,635	0,000	-0,635
all7679	0,635	0,000	-0,635
alr1580	0,628	0,000	-0,628
alr2140	0,628	0,000	-0,628
all0708	0,624	0,000	-0,624
alr0943	0,624	0,000	-0,624
alr5160	0,624	0,000	-0,624
alr8509	0,617	0,000	-0,617
all1020	0,615	0,000	-0,615
alr2278	0,615	0,000	-0,615
alr2839	0,611	0,000	-0,611
all4644	0,611	0,000	-0,611
alr4854	0,608	0,000	-0,608
alr5243	0,605	0,000	-0,605
all7084	0,605	0,000	-0,605
all0393	0,602	0,000	-0,602
all1573	0,599	0,000	-0,599
alr7684	0,596	0,000	-0,596
alr1567	0,593	0,000	-0,593

alr4229	0,593	0,000	-0,593
aroB_1	0,591	0,000	-0,591
all2618	0,590	0,000	-0,590
alr7530	0,587	0,000	-0,587
all0422	0,584	0,000	-0,584
alr3646	0,581	0,000	-0,581
all7022	0,581	0,000	-0,581
alr2867	0,575	0,000	-0,575
all2442	0,572	0,000	-0,572
all4843	0,570	0,000	-0,570
alr5151	0,565	0,000	-0,565
all7653	0,564	0,000	-0,564
alr3492	0,563	0,000	-0,563
all3750	0,561	0,000	-0,561
all7225	0,561	0,000	-0,561
all0372	0,559	0,000	-0,559
all1888	0,557	0,000	-0,557
alr2138	0,556	0,000	-0,556
alr5370	0,556	0,000	-0,556
alr5329	0,551	0,000	-0,551
all1990	0,546	0,000	-0,546
all3257	0,546	0,000	-0,546
alr5201	0,542	0,000	-0,542
alr1332	0,538	0,000	-0,538
folE_1	0,538	0,000	-0,538
all4593	0,537	0,000	-0,537
all8540	0,536	0,000	-0,536
all0342	0,533	0,000	-0,533
all4635	0,531	0,000	-0,531
all7267	0,530	0,000	-0,530
all4437	0,528	0,000	-0,528
all2633	0,521	0,000	-0,521
alr4769	0,512	0,000	-0,512
alr0280	0,510	0,000	-0,510
all2034	0,509	0,000	-0,509
alr7383	0,501	0,000	-0,501
alr1507	0,499	0,000	-0,499
all3349	0,497	0,000	-0,497
alr5211	0,496	0,000	-0,496
aspA	0,495	0,000	-0,495
alr1200	0,493	0,000	-0,493
all4729	0,493	0,000	-0,493

alr3125	0,491	0,000	-0,491
recQ	0,485	0,000	-0,485
alr3769	0,484	0,000	-0,484
alr7522	0,484	0,000	-0,484
alr7559	0,484	0,000	-0,484
alr1167	0,480	0,000	-0,480
all1608	0,480	0,000	-0,480
all1415	0,479	0,000	-0,479
alr2857	0,479	0,000	-0,479
devA	0,477	0,000	-0,477
alr2226	0,473	0,000	-0,473
all2676	0,472	0,000	-0,472
alr2775	0,467	0,000	-0,467
all7668	0,465	0,000	-0,465
alr1505	0,462	0,000	-0,462
alr2064	0,461	0,000	-0,461
all7525	0,460	0,000	-0,460
all7016	0,456	0,000	-0,456
alr7581	0,454	0,000	-0,454
alr7650	0,454	0,000	-0,454
alr1918	0,453	0,000	-0,453
nagB	0,451	0,000	-0,451
alr7535	0,451	0,000	-0,451
alr4247	0,450	0,000	-0,450
alr0191	0,444	0,000	-0,444
all7353	0,444	0,000	-0,444
alr3216	0,442	0,000	-0,442
alr2628	0,441	0,000	-0,441
alr7209	0,441	0,000	-0,441
alr4619	0,437	0,000	-0,437
alr5296	0,437	0,000	-0,437
alr0228	0,436	0,000	-0,436
alr5318	0,434	0,000	-0,434
alr1198	0,431	0,000	-0,431
alr1306	0,431	0,000	-0,431
all7671	0,425	0,000	-0,425
all7288	0,420	0,000	-0,420
alr2866	0,417	0,000	-0,417
alr7544	0,416	0,000	-0,416
all2658	0,414	0,000	-0,414
all2845	0,414	0,000	-0,414
dapF_2	0,408	0,000	-0,408

alr1634	0,405	0,000	-0,405
alr2154	0,405	0,000	-0,405
all2584	0,403	0,000	-0,403
all0341	0,401	0,000	-0,401
all2619	0,401	0,000	-0,401
all3488	0,400	0,000	-0,400
all2963	0,397	0,000	-0,397
alr0789	0,396	0,000	-0,396
alr7213	0,396	0,000	-0,396
alr7541	0,393	0,000	-0,393
alr4278	0,389	0,000	0,389
alr7132	0,382	0,000	-0,382
alr0722	0,380	0,000	-0,380
all1475	0,378	0,000	-0,378
all3772	0,374	0,000	-0,374
alr3393	0,371	0,000	-0,371
alr7157	0,371	0,000	-0,371
alr4447	0,369	0,000	-0,369
alr4655	0,364	0,000	-0,364
all5310	0,355	0,000	-0,355
alr3627	0,354	0,000	-0,354
alr2590	0,353	0,000	-0,353
alr2595	0,353	0,000	-0,353
alr2627	0,352	0,000	-0,352
alr2593	0,350	0,000	-0,350
all7291	0,349	0,000	-0,349
alr0558	0,345	0,000	-0,345
all0914	0,345	0,000	-0,345
alr2187	0,345	0,000	-0,345
alr2828	0,345	0,000	-0,345
alr2575	0,343	0,000	-0,343
alr2294	0,340	0,000	-0,340
alr4882	0,340	0,000	-0,340
alr3155	0,338	0,000	-0,338
alr5203	0,336	0,000	-0,336
alr1901	0,335	0,000	-0,335
all0426	0,330	0,000	-0,330
alr4032	0,322	0,000	-0,322
alr7089	0,320	0,000	-0,320
alr1680	0,313	0,000	-0,313
pilT_1	0,313	0,000	-0,313
alr8522	0,311	0,000	-0,311

phnM_1	0,308	0,000	-0,308
alr4881	0,305	0,000	-0,305
devC	0,302	0,000	-0,302
all2642	0,301	0,000	-0,301
alr0895	0,299	0,000	-0,299
alr0897	0,299	0,000	-0,299
all4349	0,292	0,000	-0,292
nifS_1	0,291	0,000	-0,291
all7195	0,291	0,000	-0,291
alr2678	0,290	0,000	-0,290
all7663	0,290	0,000	-0,290
all3975	0,289	0,000	-0,289
all2171	0,287	0,000	-0,287
all2656	0,281	0,000	-0,281
alr2256	0,281	0,000	-0,281
alr3072	0,275	0,000	-0,275
all2687	0,273	0,000	-0,273
all0919	0,272	0,000	-0,272
pilT_2	0,270	0,000	-0,270
all0416	0,269	0,000	-0,269
alr2626	0,267	0,000	-0,267
hstK	0,263	0,000	-0,263
all1168	0,259	0,000	-0,259
dnaB_2	0,257	0,000	-0,257
all2796	0,248	0,000	-0,248
alr5075	0,243	0,000	-0,243
all0396	0,236	0,000	-0,236
alr0744	0,236	0,000	-0,236
all0726	0,222	0,000	-0,222
all2649	0,221	0,000	-0,221
alr1527	0,203	0,000	-0,203
alr2663	0,201	0,000	-0,201
all1644	0,199	0,000	-0,199
all5116	0,198	0,000	-0,198
all2146	0,195	0,000	-0,195
all0394	0,192	0,000	-0,192
alr7539	0,192	0,000	-0,192
all2843	0,186	0,000	-0,186
alr2476	0,183	0,000	-0,183
alr1468	0,175	0,000	-0,175
alr3471	0,162	0,000	-0,162
hglE_2	0,151	0,000	-0,151

all2648	0,135	0,000	-0,135
all5111	0,121	0,000	-0,121
all1643	0,073	0,000	-0,073
all7501	0,000	0,003	0,003
all2643	0,000	0,167	0,167
all2674	0,000	0,212	0,212
all2243	0,000	0,300	0,300
alr7064	0,000	0,303	0,303
all7136	0,000	0,338	0,338
alr7046	0,000	0,340	0,340
all4002	0,000	0,386	0,386
all0324	0,000	0,411	0,411
alr2711	0,000	0,431	0,431
alr1180	0,000	0,435	0,435
alr3065	0,000	0,436	0,436
all4126	0,000	0,444	0,444
alr2257	0,000	0,458	0,458
all2032	0,000	0,471	0,471
alr4974	0,000	0,477	0,477
all0721	0,000	0,493	0,493
all2653	0,000	0,516	0,516
all2021	0,000	0,516	0,516
alr1912	0,000	0,535	0,535
all0360	0,000	0,537	0,537
all0932	0,000	0,537	0,537
all2159	0,000	0,540	0,540
alr7574	0,000	0,575	0,575
alr8007	0,000	0,584	0,584
alr7567	0,000	0,586	0,586
thrS_2	0,000	0,601	0,601
alr8532	0,000	0,603	0,603
alr1276	0,000	0,667	0,667
alr1528	0,000	0,669	0,669
alr5200	0,000	0,679	0,679
all7123	0,000	0,687	0,687
all7135	0,000	0,689	0,689
alr3885	0,000	0,700	0,700
phnK	0,000	0,713	0,713
alr2834	0,000	0,719	0,719
all1478	0,000	0,727	0,727
alr1563	0,000	0,727	0,727
all2578	0,000	0,736	0,736

alr7549	0,000	0,745	0,745
all1574	0,000	0,748	0,748
alr7533	0,000	0,754	0,754
purN	0,000	0,764	0,764
all1179	0,000	0,767	0,767
all7270	0,000	0,770	0,770
alr9010	0,000	0,770	0,770
alr0720	0,000	0,777	0,777
all2868	0,000	0,793	0,793
alr3774	0,000	0,793	0,793
alr7029	0,000	0,797	0,797
all5245	0,000	0,822	0,822
alr1579	0,000	0,825	0,825
all7590	0,000	0,844	0,844
alr1698	0,000	0,876	0,876
phnI	0,000	0,885	0,885
alr0765	0,000	0,898	0,898
all4474	0,000	0,898	0,898
all1711	0,000	0,920	0,920
all4243	0,000	0,920	0,920
alr0668	0,000	0,925	0,925
all2792	0,000	0,925	0,925
alr7003	0,000	0,925	0,925
all2652	0,000	0,927	0,927
alr3507	0,000	0,944	0,944
hepB	0,000	0,944	0,944
all3728	0,000	0,954	0,954
all7645	0,000	0,954	0,954
all8068	0,000	0,959	0,959
alr3121	0,000	0,961	0,961
all7523	0,000	0,964	0,964
alr5228	0,000	0,969	0,969
all7675	0,000	0,974	0,974
alr1444	0,000	0,984	0,984
alr7095	0,000	0,984	0,984
petB	40,001	40,050	1,001
all0565	8,737	8,765	1,003
alr7219	5,865	5,883	1,003
alr3562	2,710	2,718	1,003
alr7215	1,046	1,049	1,003
all0604	10,703	10,737	1,003
all1162	9,375	9,404	1,003

all0164	9,307	9,336	1,003
alr3099	9,285	9,314	1,003
alr1074	5,838	5,856	1,003
glcD	2,980	2,989	1,003
rffM	5,117	5,133	1,003
comE	1,607	1,613	1,003
alr2542	4,603	4,618	1,003
alr3364	35,118	35,260	1,004
rrn23Sa	111643,134	112114,414	1,004
all8045	22,478	22,638	1,007
rpoC1	13,429	13,525	1,007
alr0055	0,000	1,008	1,008
rplT	24,530	24,747	1,009
all8076	13,577	13,697	1,009
all0979	13,208	13,325	1,009
alr1128	2,414	2,436	1,009
alr1055	0,000	1,011	1,011
dapA	16,624	16,846	1,013
nrtD	11,760	11,917	1,013
alr2807	11,760	11,917	1,013
alr4898	4,670	4,733	1,013
alr4351	4,087	4,141	1,013
all5104	1,972	1,998	1,013
all5292	0,000	1,017	1,017
alr1834	8,189	8,328	1,017
rrn16Sd	245285,335	249629,196	1,018
all3660	24,799	25,247	1,018
alr0750	32,900	33,557	1,020
all4555	5,655	5,768	1,020
ycf44	4,296	4,382	1,020
alr4526	9,643	9,860	1,023
all4182	16,180	16,544	1,023
alr4232	8,572	8,765	1,023
alr7622	2,654	2,719	1,025
all0184	5,456	5,591	1,025
all2770	6,502	6,685	1,028
alr1600	6,289	6,466	1,028
all2951	10,369	10,660	1,028
glcE	6,216	6,391	1,028
nrtC_1	12,776	13,147	1,029
rplJ	16,680	17,192	1,031
psbZ_1	45,310	46,701	1,031

alr2372	8,929	9,203	1,031
all3314	2,055	2,118	1,031
asr3098	52,625	54,439	1,034
all3953	12,091	12,525	1,036
alr1531	13,858	14,409	1,040
all3568	8,173	8,589	1,051
all4540	2,538	2,667	1,051
all4217	2,245	2,360	1,051
alr1063	1,123	1,180	1,051
alr3625	0,989	1,040	1,051
atpG	23,495	24,691	1,051
all2736	13,112	13,780	1,051
pex	12,079	12,694	1,051
asl4562	9,867	10,369	1,051
alr8510	9,847	10,349	1,051
rplP	9,798	10,297	1,051
ndhE	6,868	7,218	1,051
alr2606	6,868	7,218	1,051
alr4264	6,568	6,902	1,051
alr0655	5,307	5,577	1,051
alr4583	5,062	5,320	1,051
all1590	4,934	5,185	1,051
asl4053	4,934	5,185	1,051
prfB	4,782	5,025	1,051
alr1548	4,476	4,704	1,051
uvrC	4,462	4,689	1,051
all1076	4,259	4,476	1,051
asr8082	3,936	4,136	1,051
alr4777	3,849	4,045	1,051
asl2848	3,538	3,718	1,051
all5247	3,384	3,557	1,051
all3908	3,284	3,451	1,051
alr2472	3,253	3,419	1,051
trmB	3,184	3,346	1,051
all2121	3,175	3,336	1,051
pfkA	2,935	3,085	1,051
alr3085	2,895	3,042	1,051
all3145	2,848	2,993	1,051
alr3027	2,356	2,476	1,051
alr7168	2,060	2,165	1,051
all7611	1,968	2,068	1,051
all1880	1,951	2,051	1,051

alr4965	1,946	2,045	1,051
alr3372	1,868	1,963	1,051
all1515	1,717	1,804	1,051
all2511	1,709	1,796	1,051
all1251	1,629	1,712	1,051
alr2809	1,629	1,712	1,051
alr7207	1,466	1,540	1,051
all5069	1,424	1,496	1,051
alr3863	1,337	1,405	1,051
alr2723	1,168	1,227	1,051
all0412	1,098	1,154	1,051
all0454	1,098	1,154	1,051
alr1255	1,081	1,136	1,051
all2506	1,081	1,136	1,051
all3942	1,033	1,086	1,051
all7134	0,952	1,000	1,051
alr4031	0,820	0,862	1,051
all2956	0,637	0,669	1,051
alr5199	0,569	0,598	1,051
alr7540	0,422	0,444	1,051
all5112	0,420	0,441	1,051
alr1561	0,390	0,410	1,051
efp	13,183	13,854	1,051
all1895	11,644	12,236	1,051
all4074	8,730	9,174	1,051
alr2019	7,349	7,723	1,051
alr0576	6,487	6,817	1,051
alr5253	5,852	6,150	1,051
all4257	5,338	5,609	1,051
all4749	5,189	5,454	1,051
asl4765	5,151	5,413	1,051
asl1243	4,798	5,043	1,051
asr1945	4,734	4,975	1,051
asl3409	3,574	3,756	1,051
alr2106	3,156	3,316	1,051
alr7378	3,118	3,277	1,051
recN	3,004	3,157	1,051
all5172	2,802	2,945	1,051
all7344	2,780	2,922	1,051
all4817	2,737	2,876	1,051
alr1320	2,614	2,747	1,051
all2183	2,484	2,611	1,051

alr2015	2,433	2,556	1,051
alr5158	2,383	2,504	1,051
rfbD	2,375	2,496	1,051
all4424	2,351	2,471	1,051
all3316	2,260	2,375	1,051
all1149	2,149	2,258	1,051
all1122	2,048	2,153	1,051
all0767	1,883	1,979	1,051
alr0685	1,834	1,927	1,051
alr1138	1,820	1,912	1,051
all3255	1,585	1,666	1,051
alr0760	1,578	1,658	1,051
alr7078	1,557	1,636	1,051
all1842	1,472	1,547	1,051
alr3937	1,430	1,503	1,051
alr1404	1,396	1,467	1,051
all1756	1,278	1,343	1,051
all3035	1,220	1,283	1,051
alr4028	1,216	1,278	1,051
alr7049	1,160	1,219	1,051
alr3807	1,158	1,217	1,051
uppP	1,091	1,147	1,051
alr2597	1,001	1,052	1,051
alr0790	0,995	1,046	1,051
all0983	0,970	1,020	1,051
alr2175	0,811	0,852	1,051
all3736	0,791	0,831	1,051
devB_2	0,737	0,775	1,051
all5123	0,695	0,730	1,051
comM	0,687	0,722	1,051
all3787	0,678	0,712	1,051
alr0709	0,390	0,410	1,051
all3500	0,341	0,359	1,051
alr2489	6,609	6,946	1,051
alr0718	4,020	4,224	1,051
all7317	2,836	2,981	1,051
alr1920	1,418	1,490	1,051
alr3395	11,622	12,356	1,063
alr3343	20,720	22,069	1,065
hemF	13,585	14,470	1,065
all5077	12,922	13,822	1,070
all0775	5,124	5,481	1,070

alr2520	0,000	1,070	1,070
alr5030	0,000	1,070	1,070
all4590	4,785	5,129	1,072
alr7628	6,332	6,787	1,072
rpsF	23,566	25,329	1,075
surE_2	8,340	8,995	1,079
all3530	5,282	5,697	1,079
all2004	5,294	5,722	1,081
all0158	7,103	7,698	1,084
secE	25,246	27,360	1,084
asl4317	23,648	25,628	1,084
all1547	6,746	7,362	1,091
all5295	4,531	4,945	1,091
all2500	23,134	25,313	1,094
all0619	0,000	1,096	1,096
rbpA1	26,073	28,591	1,097
pds	11,190	12,270	1,097
rplA	27,358	30,034	1,098
all2285	0,000	1,102	1,102
alr1607	10,963	12,098	1,103
alr3685	7,607	8,393	1,103
alr2543	5,121	5,651	1,103
ribD	2,941	3,245	1,103
cphA_1	1,833	2,023	1,103
infC_2	17,087	18,855	1,103
rps7	7,437	8,206	1,103
alr3833	5,724	6,316	1,103
alr3377	5,406	5,965	1,103
all1670	10,066	11,107	1,103
alr4625	3,730	4,116	1,103
patA	3,073	3,391	1,103
tyrS	2,926	3,229	1,103
all5097	4,990	5,506	1,103
all2507	2,949	3,254	1,103
all7030	2,061	2,274	1,103
all3030	0,000	1,109	1,109
all4983	0,000	1,109	1,109
nusG	29,882	33,148	1,109
alr4331	11,540	12,841	1,113
all3023	0,000	1,115	1,115
nirA	19,351	21,593	1,116
cphA_2	12,427	13,876	1,117

plsX	29,361	32,829	1,118
alr1247	12,612	14,109	1,119
pyrH	14,895	16,664	1,119
alr1859	0,000	1,122	1,122
rbpD_1	8,603	9,687	1,126
all2546	4,491	5,057	1,126
all2108	4,046	4,556	1,126
alr8016	2,566	2,889	1,126
all1483	1,010	1,138	1,126
alr3178	4,347	4,895	1,126
all7375	16,478	18,554	1,126
asl4743	12,771	14,379	1,126
alr2719	9,021	10,158	1,126
all5255	6,287	7,079	1,126
alr3659	5,541	6,239	1,126
alr0816	5,307	5,976	1,126
lpxB	4,246	4,781	1,126
all5196	3,855	4,341	1,126
all4139	3,420	3,851	1,126
phnE_2	3,084	3,473	1,126
murQ	2,654	2,988	1,126
alr0730	6,032	6,792	1,126
vapC_1	6,010	6,767	1,126
all3130	5,108	5,752	1,126
all3835	4,618	5,199	1,126
ftsY	4,483	5,047	1,126
alr2111	3,616	4,072	1,126
all3408	3,493	3,933	1,126
folE_2	3,463	3,900	1,126
alr2467	3,096	3,486	1,126
alr4888	2,848	3,207	1,126
all4688	2,761	3,109	1,126
all1211	1,828	2,059	1,126
all4884	1,599	1,801	1,126
all7187	1,169	1,317	1,126
all8079	5,801	6,565	1,132
alr4150	11,358	12,891	1,135
all3873	11,099	12,597	1,135
alr2695	0,000	1,136	1,136
all1897	29,801	33,885	1,137
all0985	5,215	5,937	1,138
alr1978	3,352	3,816	1,138

alr5307	46,958	53,517	1,140
alr0114	12,313	14,055	1,142
nrtA_1	18,269	20,868	1,142
alr0856	7,057	8,090	1,146
alr4681	4,475	5,131	1,146
alr3350	4,197	4,812	1,146
asl2195	13,810	15,833	1,146
all4481	6,999	8,024	1,146
alr0295	5,512	6,320	1,146
alr2933	3,125	3,583	1,146
glmU	2,842	3,258	1,146
all0182	1,794	2,057	1,146
all8013	9,514	10,907	1,146
all0316	4,339	4,975	1,146
alr7200	2,378	2,727	1,146
all2533	1,861	2,134	1,146
chlH_2	1,045	1,198	1,146
all0746	0,000	1,150	1,150
all3586	0,000	1,150	1,150
alr3524	4,306	4,960	1,152
alr0402	43,128	49,745	1,153
all4502	11,005	12,694	1,153
all2384	3,202	3,701	1,156
all1826	7,328	8,471	1,156
glgC	14,391	16,694	1,160
groEL_1	3,954	4,593	1,162
alr4255	5,065	5,883	1,162
alr3170	2,628	3,062	1,165
all1023	15,705	18,298	1,165
alr2697	0,000	1,172	1,172
alr3535	0,000	1,172	1,172
narB	7,346	8,624	1,174
all3403	5,814	6,874	1,182
ubiA_2	3,210	3,795	1,182
alr3996	2,566	3,034	1,182
all2438	2,347	2,775	1,182
cbiO_2	2,256	2,667	1,182
all4835	2,147	2,539	1,182
all0389	1,705	2,015	1,182
alr5256	1,698	2,008	1,182
alr2532	1,547	1,828	1,182
queA	1,173	1,387	1,182

alr1909	1,123	1,327	1,182
alr1666	0,957	1,131	1,182
all4374	0,805	0,952	1,182
all4052	0,737	0,871	1,182
alr3183	18,197	21,513	1,182
all4107	6,902	8,160	1,182
all0156	2,684	3,173	1,182
alr2974	2,162	2,556	1,182
all1616	2,048	2,422	1,182
all7629	2,013	2,380	1,182
all4402	1,930	2,282	1,182
all3317	1,895	2,241	1,182
all1418	1,680	1,986	1,182
alr3762	1,586	1,875	1,182
alr0247	1,531	1,810	1,182
coxA_3	0,840	0,993	1,182
alr3676	0,719	0,849	1,182
alr3402	7,916	9,359	1,182
all0471	6,802	8,041	1,182
all2681	6,487	7,669	1,182
all3254	6,398	7,564	1,182
all4573	4,702	5,559	1,182
alr2055	3,925	4,640	1,182
alr7349	3,736	4,417	1,182
alr0018	3,538	4,183	1,182
alr7169	3,281	3,879	1,182
alr1056	3,073	3,633	1,182
phnG	3,073	3,633	1,182
alr0214	2,975	3,517	1,182
all4038	2,865	3,388	1,182
all2750	2,700	3,192	1,182
all4759	2,375	2,808	1,182
alr3200	2,312	2,734	1,182
all3578	2,128	2,516	1,182
all0089	2,104	2,487	1,182
all7331	1,987	2,350	1,182
all1253	1,954	2,310	1,182
alr8542	1,954	2,310	1,182
all2013	1,946	2,301	1,182
all0955	1,891	2,236	1,182
alr4072	1,891	2,236	1,182
alr1077	1,876	2,218	1,182

all0464	1,842	2,178	1,182
dapF_1	1,668	1,972	1,182
all3574	1,656	1,958	1,182
all1807	1,639	1,937	1,182
all1228	1,562	1,847	1,182
alr1653	1,478	1,747	1,182
all4705	1,455	1,720	1,182
alr2213	1,433	1,694	1,182
alr1000	1,294	1,530	1,182
alr0719	1,185	1,401	1,182
patB	0,881	1,042	1,182
all5311	0,548	0,648	1,182
alr1927	0,462	0,546	1,182
mutT_1	3,177	3,756	1,182
lspA	2,937	3,473	1,182
alr2060	2,780	3,287	1,182
alr0199	2,624	3,102	1,182
all0580	1,810	2,140	1,182
all1950	1,207	1,427	1,182
alr2188	1,071	1,266	1,182
alr0440	0,831	0,983	1,182
all4220	25,184	30,075	1,194
all0748	24,542	29,308	1,194
all1694	8,397	10,085	1,201
cmpA	0,000	1,203	1,203
all7021	0,000	1,203	1,203
alr3525	4,659	5,617	1,205
all8078	1,334	1,608	1,205
alr2764	5,000	6,027	1,205
all0200	4,649	5,604	1,205
all2275	7,784	9,439	1,213
alr3607	6,515	7,899	1,213
alr3877	3,271	3,967	1,213
alr5049	8,929	10,827	1,213
all2491	6,657	8,073	1,213
all7655	0,000	1,215	1,215
icd	11,824	14,367	1,215
all3687	20,226	24,638	1,218
alr2350	14,874	18,171	1,222
all3325	11,796	14,421	1,222
all0888	4,434	5,436	1,226
alr3529	5,473	6,710	1,226

all2110	5,202	6,378	1,226
alr2535	3,907	4,790	1,226
alr0024	3,849	4,719	1,226
all3274	3,574	4,382	1,226
all4820	1,671	2,048	1,226
alr1031	1,275	1,564	1,226
alr2279	0,808	0,991	1,226
alr2398	2,433	2,982	1,226
all4152	2,330	2,857	1,226
all0889	6,936	8,504	1,226
all4617	6,609	8,103	1,226
all5306	4,026	4,936	1,226
alr1603	3,624	4,443	1,226
desA	2,994	3,671	1,226
bioF	2,715	3,329	1,226
all1484	0,000	1,227	1,227
all2883	6,086	7,479	1,229
rpsM	29,420	36,232	1,232
all2085	8,776	10,827	1,234
alr3606	6,231	7,687	1,234
all4955	2,693	3,335	1,239
alr1308	2,790	3,455	1,239
alr4487	1,640	2,031	1,239
all4441	4,836	5,990	1,239
all2981	8,561	10,624	1,241
all4503	23,904	29,710	1,243
all4872	0,000	1,244	1,244
era	6,563	8,168	1,244
all1873	197,286	247,525	1,255
alr3091	2,402	3,030	1,261
trpE_1	0,793	1,000	1,261
all3797	15,598	19,670	1,261
hemK	9,571	12,069	1,261
asr0798	7,207	9,089	1,261
asl3860	6,415	8,090	1,261
all5036	6,198	7,816	1,261
all2107	4,597	5,797	1,261
all0945	3,496	4,409	1,261
all1508	3,243	4,090	1,261
degT	3,049	3,845	1,261
all1846	2,572	3,243	1,261
alr8029	2,326	2,933	1,261

all0475	2,263	2,854	1,261
alr4807	1,673	2,110	1,261
alr5235	1,673	2,110	1,261
all7222	1,673	2,110	1,261
sqdX	1,540	1,943	1,261
alr4808	1,374	1,732	1,261
alr2831	1,333	1,681	1,261
alr3997	1,175	1,481	1,261
alr3101	10,811	13,634	1,261
all4757	9,980	12,585	1,261
sat	8,913	11,240	1,261
all2473	8,779	11,071	1,261
alr3603	7,733	9,751	1,261
alr0643	7,298	9,203	1,261
alr3901	7,076	8,924	1,261
chlD	6,019	7,590	1,261
all1239	4,200	5,297	1,261
alr2577	4,185	5,278	1,261
all0571	3,971	5,008	1,261
all1765	3,139	3,958	1,261
recF	3,097	3,906	1,261
all4443	2,612	3,294	1,261
all1736	2,516	3,173	1,261
trpA_2	2,187	2,757	1,261
all0400	2,078	2,620	1,261
all0351	1,927	2,430	1,261
alr0786	1,692	2,134	1,261
alr0150	1,608	2,028	1,261
all1325	1,565	1,974	1,261
alr2816	1,450	1,829	1,261
alr3510	1,370	1,728	1,261
all5153	1,168	1,472	1,261
alr4878	0,620	0,782	1,261
all4375	8,714	10,988	1,261
all1141	4,140	5,221	1,261
alr3799	10,267	13,011	1,267
pys	13,516	17,163	1,270
all2952	5,560	7,060	1,270
murD	7,818	9,938	1,271
all1731	4,769	6,062	1,271
alr1217	4,235	5,404	1,276
alr1129	13,252	16,938	1,278

mutT_2	0,000	1,278	1,278
all3324	12,973	16,616	1,281
alr4847	10,544	13,543	1,284
all0268	5,017	6,471	1,290
all0355	3,976	5,129	1,290
alr1621	2,261	2,916	1,290
all3154	2,214	2,856	1,290
all4581	6,145	7,926	1,290
alr2374	9,880	12,742	1,290
hemC	13,512	17,477	1,293
alr1929	0,000	1,301	1,301
all3144	9,288	12,131	1,306
clpS_2	7,453	9,790	1,314
asr1289	19,734	25,924	1,314
ndhH	8,868	11,649	1,314
all8088	7,784	10,225	1,314
alr5045	1,147	1,506	1,314
all2547	17,963	23,597	1,314
all4999	9,372	12,312	1,314
alr2569	5,004	6,573	1,314
all4694	4,549	5,976	1,314
alr4850	3,871	5,084	1,314
alr1024	3,451	4,533	1,314
alr2751	3,199	4,202	1,314
all3016	3,170	4,164	1,314
alr4045	3,086	4,054	1,314
opcA	3,046	4,001	1,314
alr1665	2,968	3,900	1,314
all5017	2,956	3,883	1,314
all0135	2,859	3,756	1,314
alr4786	2,791	3,666	1,314
radA	1,379	1,812	1,314
nadE	1,236	1,623	1,314
all0320	2,399	3,152	1,314
all4289	10,561	13,874	1,314
all0569	5,189	6,817	1,314
alr	3,485	4,579	1,314
all2891	2,705	3,553	1,314
alr3450	2,705	3,553	1,314
all0438	1,033	1,357	1,314
all3691	0,346	0,455	1,314
cmpB	0,000	1,315	1,315

all5006	0,000	1,315	1,315
all0334	47,365	62,510	1,320
clpP_2	29,065	38,707	1,332
alr7637	0,000	1,334	1,334
alr0051	7,824	10,436	1,334
alr0276	0,646	0,862	1,334
all5267	6,246	8,332	1,334
alr4766	3,343	4,460	1,334
alr4238	1,282	1,717	1,340
alr0267	5,710	7,650	1,340
mreB	4,697	6,347	1,351
alr0486	3,665	4,952	1,351
alr3720	6,287	8,495	1,351
all2334	1,685	2,277	1,351
all3237	5,206	7,034	1,351
alr3599	4,738	6,402	1,351
all1758	3,523	4,760	1,351
alr2001	3,005	4,060	1,351
all2177	2,771	3,744	1,351
alr0370	3,478	4,699	1,351
all2776	2,112	2,854	1,351
alr4412	0,996	1,345	1,351
devR	0,000	1,363	1,363
alr7240	0,000	1,363	1,363
alr8014	0,000	1,363	1,363
alr4515	2,968	4,056	1,366
alr2752	8,846	12,085	1,366
all1290	8,714	11,904	1,366
all0839	5,705	7,794	1,366
all4306	0,000	1,374	1,374
all0172	6,868	9,474	1,379
all3782	3,434	4,737	1,379
alr4529	4,345	5,993	1,379
all3509	2,947	4,064	1,379
alr7562	2,301	3,173	1,379
asl0715	65,550	90,414	1,379
coaE	4,742	6,540	1,379
alr1324	2,071	2,857	1,379
alr7508	1,926	2,656	1,379
alr4909	1,895	2,613	1,379
asr1314	10,495	14,476	1,379
alr2044	0,919	1,268	1,379

alr1685	0,000	1,384	1,384
all4465	12,139	16,857	1,389
all0988	4,962	6,902	1,391
alr3142	17,963	25,049	1,394
alr0583	4,064	5,667	1,394
all7178	8,982	12,585	1,401
alr2983	4,397	6,161	1,401
avtA	2,455	3,440	1,401
all1683	13,370	18,734	1,401
alr1555	3,599	5,043	1,401
all3378	3,357	4,704	1,401
alr1619	3,165	4,435	1,401
rca	2,532	3,548	1,401
proA	2,394	3,354	1,401
all0875	1,900	2,663	1,401
all7188	0,000	1,409	1,409
hisH	10,464	14,759	1,410
def	11,800	16,643	1,410
all3977	6,828	9,687	1,419
alr3689	5,406	7,669	1,419
upp	5,381	7,634	1,419
alr1282	3,879	5,503	1,419
all0978	0,984	1,396	1,419
all1730	2,220	3,149	1,419
sdhA	2,027	2,876	1,419
nrtB_1	12,927	18,406	1,424
all1601	6,161	8,787	1,426
alr3644	4,040	5,761	1,426
all2701	0,000	1,427	1,427
rrn23Sc	4945,827	7072,604	1,430
rrn23Sb	9795,288	14031,768	1,433
rrn23Sd	4975,801	7128,056	1,433
all7632	2,285	3,275	1,433
asl7246	18,090	25,924	1,433
alr1136	7,381	10,578	1,433
all7024	2,880	4,127	1,433
all1797	24,060	34,580	1,437
alr2429	0,000	1,438	1,438
alr3477	0,000	1,438	1,438
alr7579	0,000	1,438	1,438
all1587	3,920	5,643	1,439
alr3699	0,000	1,442	1,442

all0215	6,578	9,505	1,445
glnH	5,151	7,443	1,445
argC_1	4,338	6,268	1,445
all2806	5,854	8,480	1,449
alr4537	11,999	17,460	1,455
hetR	15,179	22,087	1,455
thrC_1	12,510	18,203	1,455
alr0240	5,181	7,538	1,455
murF	3,358	4,886	1,455
all1812	0,000	1,461	1,461
all3903	0,000	1,461	1,461
all3802	7,076	10,358	1,464
alr3995	3,874	5,670	1,464
alr5168	5,108	7,477	1,464
alr3140	3,841	5,651	1,471
alr3723	1,920	2,825	1,471
all8520	7,237	10,648	1,471
alr1157	17,630	25,968	1,473
alr1241	9,452	14,023	1,484
all5207	5,077	7,558	1,489
alr2662	0,000	1,490	1,490
hetP_3	0,000	1,496	1,496
alr1905	19,624	29,387	1,498
coxB_1	6,560	9,823	1,498
ftsW	6,470	9,736	1,505
thiG	4,828	7,328	1,518
alr0831	0,000	1,521	1,521
leuC	7,984	12,192	1,527
all5102	0,000	1,527	1,527
alr3276	12,310	18,817	1,529
alr1560	0,000	1,534	1,534
alr4172	11,628	17,947	1,544
all2813	0,000	1,560	1,560
alr3417	7,555	11,910	1,576
all4287	6,769	10,670	1,576
asr4524	5,766	9,089	1,576
all0458	4,541	7,158	1,576
all5304	3,510	5,533	1,576
alr3199	2,985	4,706	1,576
all0675	2,538	4,001	1,576
all2924	2,335	3,681	1,576
alr4695	2,335	3,681	1,576

kaiA	2,267	3,574	1,576
all5133	2,267	3,574	1,576
all4539	2,080	3,279	1,576
alr2984	1,954	3,080	1,576
all0265	1,873	2,953	1,576
asl4805	1,769	2,789	1,576
asr4588	1,692	2,667	1,576
all0807	1,510	2,380	1,576
alr4484	1,447	2,282	1,576
mazE	1,441	2,272	1,576
all0467	1,331	2,098	1,576
all4639	1,188	1,873	1,576
all2962	1,134	1,787	1,576
all5002	1,123	1,770	1,576
alr4553	0,989	1,560	1,576
all1363	0,961	1,515	1,576
all5003	0,855	1,348	1,576
all0938	0,846	1,334	1,576
all2424	0,805	1,269	1,576
alr2582	0,721	1,136	1,576
alr1002	0,699	1,102	1,576
all1256	0,699	1,102	1,576
alr1977	0,642	1,011	1,576
cobU	0,642	1,011	1,576
all2040	0,626	0,987	1,576
all3695	0,600	0,946	1,576
all7220	0,587	0,925	1,576
alr1139	0,567	0,893	1,576
all0563	0,561	0,885	1,576
all0906	0,480	0,757	1,576
all8065	0,442	0,697	1,576
alr4905	0,423	0,667	1,576
alr2510	0,368	0,581	1,576
alr2591	0,355	0,559	1,576
all4722	0,330	0,520	1,576
alr3162	0,316	0,499	1,576
all1012	0,270	0,425	1,576
alr3754	0,269	0,424	1,576
all0390	0,201	0,317	1,576
asl3264	14,371	22,653	1,576
all8063	11,783	18,575	1,576
alr8025	11,512	18,146	1,576

cheW	9,895	15,598	1,576
asl4748	9,218	14,531	1,576
rplW	7,784	12,270	1,576
asr4314	7,185	11,327	1,576
asl3851	7,034	11,088	1,576
all1723	6,895	10,870	1,576
rplV	6,811	10,737	1,576
alr7661	5,413	8,532	1,576
alr7204	5,202	8,201	1,576
petC_1	5,114	8,061	1,576
all5098	4,831	7,616	1,576
alr7507	4,246	6,693	1,576
alr1057	4,097	6,458	1,576
alr8033	3,980	6,275	1,576
alr0957	3,892	6,135	1,576
alr5217	3,871	6,101	1,576
all0155	3,849	6,068	1,576
all0065	2,994	4,719	1,576
asr1899	2,994	4,719	1,576
asl3523	2,994	4,719	1,576
alr3896	2,951	4,651	1,576
all3536	2,780	4,382	1,576
all0398	2,737	4,314	1,576
all2693	2,737	4,314	1,576
asr1775	2,624	4,136	1,576
ntcB	2,611	4,116	1,576
all1367	2,525	3,980	1,576
alr0920	2,499	3,939	1,576
alr7201	2,498	3,937	1,576
all0481	2,484	3,916	1,576
alr3444	2,477	3,904	1,576
all2017	2,450	3,861	1,576
alr4706	2,289	3,609	1,576
all4774	2,275	3,586	1,576
alr5188	2,094	3,301	1,576
asr1836	2,085	3,287	1,576
all4292	2,085	3,287	1,576
alr2482	2,031	3,201	1,576
all2088	2,013	3,173	1,576
alr5129	1,996	3,146	1,576
alr4758	1,796	2,832	1,576
asr0257	1,717	2,707	1,576

mop	1,668	2,629	1,576
asr7343	1,668	2,629	1,576
asr1344	1,645	2,592	1,576
asl2400	1,645	2,592	1,576
cyaB1	1,629	2,568	1,576
asr5313	1,622	2,556	1,576
alr1946	1,536	2,422	1,576
alr0998	1,531	2,414	1,576
asr1213	1,497	2,360	1,576
asr4302	1,497	2,360	1,576
asl5041	1,497	2,360	1,576
asr8513	1,497	2,360	1,576
all0275	1,477	2,328	1,576
alr1174	1,472	2,320	1,576
alr5227	1,450	2,286	1,576
all7510	1,450	2,286	1,576
all0263	1,449	2,284	1,576
alr2300	1,433	2,258	1,576
alr3097	1,390	2,191	1,576
asr7330	1,390	2,191	1,576
all4179	1,376	2,169	1,576
all7316	1,327	2,092	1,576
alr4611	1,322	2,084	1,576
alr3188	1,312	2,068	1,576
asl1839	1,255	1,979	1,576
all2556	1,255	1,979	1,576
alr7321	1,255	1,979	1,576
asr7339	1,255	1,979	1,576
alr1209	1,249	1,969	1,576
alr1803	1,247	1,965	1,576
all7676	1,247	1,965	1,576
all2941	1,223	1,927	1,576
alr3202	1,208	1,904	1,576
all1838	1,204	1,897	1,576
all4541	1,204	1,897	1,576
asl0151	1,179	1,859	1,576
all2059	1,179	1,859	1,576
alr1656	1,128	1,778	1,576
all0385	1,107	1,745	1,576
all1866	1,052	1,658	1,576
petJ_1	1,043	1,643	1,576
alr8531	1,043	1,643	1,576

all9019	1,030	1,624	1,576
alr0758	1,024	1,615	1,576
alr0898	1,024	1,615	1,576
alr2205	1,024	1,615	1,576
alr0303	1,015	1,600	1,576
alr4921	1,007	1,587	1,576
all3039	0,973	1,534	1,576
alr4363	0,973	1,534	1,576
alr4601	0,965	1,521	1,576
all3677	0,927	1,461	1,576
all2685	0,898	1,416	1,576
minD_1	0,888	1,400	1,576
alr2541	0,881	1,389	1,576
all4824	0,834	1,315	1,576
alr0014	0,817	1,287	1,576
alr2859	0,797	1,256	1,576
all2747	0,778	1,227	1,576
all0954	0,768	1,211	1,576
all0984	0,768	1,211	1,576
alr8555	0,768	1,211	1,576
all2661	0,714	1,126	1,576
alr4241	0,708	1,115	1,576
alr7052	0,687	1,083	1,576
alr5332	0,683	1,076	1,576
alr5086	0,667	1,052	1,576
all1642	0,621	0,979	1,576
all7055	0,618	0,974	1,576
alr1499	0,611	0,964	1,576
all2000	0,590	0,930	1,576
alr7334	0,578	0,911	1,576
all7120	0,572	0,902	1,576
alr2296	0,547	0,862	1,576
all2869	0,537	0,846	1,576
all7221	0,499	0,787	1,576
phnL	0,482	0,761	1,576
all2910	0,479	0,754	1,576
all7218	0,477	0,751	1,576
alr7299	0,441	0,695	1,576
alr0922	0,434	0,684	1,576
alr7546	0,422	0,664	1,576
all5246	0,417	0,657	1,576
all4324	0,411	0,648	1,576

alr2837	0,359	0,566	1,576
alr0691	0,354	0,558	1,576
alr3474	0,353	0,556	1,576
all0562	0,316	0,497	1,576
all5322	0,283	0,446	1,576
nifE	0,243	0,383	1,576
hetM	0,230	0,363	1,576
alr0357	0,215	0,338	1,576
alr1584	0,179	0,283	1,576
all4664	11,413	17,991	1,576
alr2766	6,427	10,132	1,576
all3048	6,427	10,132	1,576
tpiA	6,272	9,887	1,576
asl2519	6,066	9,561	1,576
all4101	4,561	7,190	1,576
alr2709	4,429	6,981	1,576
asl4177	4,324	6,817	1,576
all1161	4,230	6,669	1,576
all7259	3,322	5,237	1,576
all3148	3,214	5,066	1,576
all0015	3,177	5,008	1,576
all3462	3,132	4,936	1,576
alr0717	3,100	4,886	1,576
asr1532	3,033	4,781	1,576
alr1360	2,802	4,417	1,576
alr0520	2,684	4,231	1,576
coxC_3	2,324	3,663	1,576
asr5289	2,162	3,408	1,576
all3704	2,025	3,192	1,576
alr4277	2,022	3,187	1,576
leuD	1,726	2,720	1,576
alr1677	1,662	2,620	1,576
alr3611	1,589	2,504	1,576
all4399	1,589	2,504	1,576
asl3928	1,516	2,390	1,576
asl4047	1,516	2,390	1,576
asl1218	1,478	2,330	1,576
all3861	1,478	2,330	1,576
asr4093	1,424	2,245	1,576
alr4486	1,418	2,236	1,576
asl0399	1,407	2,218	1,576
alr5259	1,407	2,218	1,576

alr0944	1,342	2,116	1,576
all0463	1,297	2,045	1,576
all1610	1,297	2,045	1,576
all5278	1,239	1,953	1,576
alr3187	1,163	1,833	1,576
nadD	1,162	1,831	1,576
all5195	1,115	1,757	1,576
all4003	1,021	1,610	1,576
alr0933	1,011	1,594	1,576
alr4167	0,938	1,478	1,576
all0976	0,934	1,472	1,576
all7360	0,865	1,363	1,576
all0997	0,852	1,343	1,576
alr1470	0,852	1,343	1,576
all7373	0,852	1,343	1,576
all2317	0,794	1,252	1,576
alr3623	0,794	1,252	1,576
all0291	0,753	1,187	1,576
alr4115	0,739	1,165	1,576
alr3296	0,675	1,064	1,576
all1188	0,654	1,031	1,576
alr0347	0,649	1,023	1,576
alr2240	0,593	0,934	1,576
all3987	0,593	0,934	1,576
alr4005	0,557	0,879	1,576
all0424	0,555	0,874	1,576
all3359	0,555	0,874	1,576
all1281	0,546	0,860	1,576
alr2841	0,492	0,775	1,576
all0621	0,446	0,703	1,576
all0207	0,438	0,691	1,576
alr3105	0,356	0,561	1,576
all2165	0,336	0,529	1,576
ddl	0,320	0,504	1,576
all3028	0,320	0,504	1,576
all2292	0,297	0,468	1,576
alr1585	0,248	0,392	1,576
alr5353	0,204	0,322	1,576
alr1551	0,193	0,304	1,576
all4299	0,184	0,290	1,576
minE	9,532	15,025	1,576
alr0088	5,838	9,203	1,576

asr5071	3,649	5,752	1,576
alr5275	2,937	4,630	1,576
asr2448	2,715	4,280	1,576
all1814	2,458	3,875	1,576
all1863	2,433	3,835	1,576
alr1941	2,203	3,473	1,576
all3545	2,203	3,473	1,576
alr5220	2,142	3,377	1,576
all0623	2,071	3,265	1,576
alr0913	2,067	3,258	1,576
alr5103	1,984	3,127	1,576
alr0208	1,904	3,001	1,576
asl4865	1,824	2,876	1,576
alr5157	1,824	2,876	1,576
alr1589	1,810	2,854	1,576
all0373	1,743	2,747	1,576
asl4034	1,599	2,521	1,576
alr4166	1,528	2,409	1,576
alr1231	1,505	2,372	1,576
asr2953	1,460	2,301	1,576
all2099	1,363	2,149	1,576
alr2433	1,358	2,140	1,576
asr3387	1,358	2,140	1,576
asr5145	1,358	2,140	1,576
alr5178	1,317	2,076	1,576
alr5242	1,260	1,986	1,576
all8075	1,198	1,889	1,576
alr2614	1,071	1,689	1,576
all7600	1,033	1,629	1,576
ispD	1,020	1,607	1,576
alr0645	0,981	1,547	1,576
all1034	0,942	1,484	1,576
all7262	0,871	1,374	1,576
all0891	0,828	1,305	1,576
all4051	0,828	1,305	1,576
all2598	0,819	1,292	1,576
alr4493	0,789	1,244	1,576
all4709	0,789	1,244	1,576
miaA	0,789	1,244	1,576
nifH2	0,784	1,235	1,576
gvpJ	0,784	1,235	1,576
all2664	0,784	1,235	1,576

alr3394	0,679	1,070	1,576
alr5212	0,679	1,070	1,576
all2720	0,643	1,014	1,576
all2053	0,638	1,006	1,576
alr4105	0,636	1,002	1,576
all2573	0,615	0,969	1,576
rfbC_1	0,615	0,969	1,576
alr2742	0,613	0,966	1,576
all4143	0,608	0,959	1,576
all4444	0,608	0,959	1,576
all4928	0,599	0,944	1,576
all2002	0,596	0,939	1,576
all4844	0,596	0,939	1,576
cbiQ_2	0,528	0,833	1,576
alr7224	0,528	0,833	1,576
alr2822	0,519	0,818	1,576
alr7094	0,517	0,814	1,576
alr1194	0,510	0,804	1,576
alr1008	0,367	0,579	1,576
alr3071	0,366	0,577	1,576
all4859	0,346	0,546	1,576
all2157	0,339	0,535	1,576
all1169	0,319	0,503	1,576
all1831	0,319	0,503	1,576
alr2824	0,319	0,502	1,576
all2290	0,274	0,432	1,576
nifN	0,262	0,414	1,576
all2244	0,184	0,289	1,576
all2129	0,122	0,192	1,576
alr0681	0,000	1,587	1,587
all8004	0,000	1,587	1,587
alr2926	0,000	1,600	1,600
rrn16Sb	3371,091	5428,269	1,610
rrn16Sc	3340,509	5400,086	1,617
all2170	4,217	6,855	1,626
rrn16Sa	3316,278	5433,832	1,639
alr3482	0,000	1,658	1,658
alr2743	5,346	8,870	1,659
all3041	2,131	3,536	1,659
alr3619	0,000	1,668	1,668
all3436	0,000	1,673	1,673
alr5149	0,000	1,673	1,673

recO	6,290	10,535	1,675
all2023	2,587	4,350	1,681
alr3874	6,868	11,600	1,689
alr4848	3,860	6,592	1,708
all0513	6,832	11,748	1,720
all1116	0,000	1,731	1,731
ccmL	11,447	19,849	1,734
alr0484	3,779	6,552	1,734
alr3514	3,879	6,726	1,734
all2631	1,604	2,781	1,734
all3989	1,290	2,237	1,734
alr2346	11,120	19,282	1,734
all3779	0,000	1,736	1,736
all5316	0,000	1,742	1,742
alr7582	0,000	1,747	1,747
alr3297	10,008	17,529	1,752
all2341	9,059	15,867	1,752
all2347	3,780	6,621	1,752
alr4320	3,700	6,481	1,752
bvdR	3,253	5,698	1,752
trmE	2,284	4,001	1,752
all4546	8,783	15,474	1,762
alr0239	11,641	20,574	1,767
all7080	0,000	1,770	1,770
all7177	3,766	6,679	1,773
all2521	2,919	5,177	1,773
alr0367	0,806	1,429	1,773
alr4784	10,564	18,844	1,784
kaiC	3,368	6,017	1,787
all2509	1,817	3,246	1,787
alr5232	0,000	1,796	1,796
alr1942	7,568	13,634	1,802
recJ_2	2,280	4,107	1,802
ycf55	1,388	2,500	1,802
rplK	21,379	38,885	1,819
all7659	2,074	3,772	1,819
all2635	0,969	1,763	1,819
all4237	0,000	1,822	1,822
lpxC	4,986	9,170	1,839
alr1113	1,520	2,795	1,839
alr3813	9,158	16,842	1,839
alr5005	9,098	16,732	1,839

all0173	7,198	13,237	1,839
alr4660	4,520	8,312	1,839
all4869	3,630	6,676	1,839
all0459	5,838	10,737	1,839
all5165	12,184	22,407	1,839
alr5209	5,389	9,911	1,839
alr0159	0,000	1,850	1,850
alr0882	9,456	17,498	1,851
groES	19,086	35,396	1,855
all3838	2,792	5,177	1,855
asl2401	0,000	1,859	1,859
asl8549	0,000	1,859	1,859
all1220	3,916	7,295	1,863
asl2923	0,000	1,878	1,878
asr7252	0,000	1,878	1,878
all4885	0,000	1,891	1,891
alr3897	2,123	4,016	1,892
bioD	2,583	4,886	1,892
all4680	2,041	3,861	1,892
asr2819	6,868	12,992	1,892
all4544	5,406	10,225	1,892
all8030	6,736	12,742	1,892
alr1545	4,561	8,628	1,892
hisA	4,526	8,561	1,892
all3859	1,796	3,398	1,892
alr4046	1,738	3,287	1,892
alr5117	1,707	3,229	1,892
alr1146	1,377	2,605	1,892
all2133	5,260	9,949	1,892
all7105	2,354	4,453	1,892
all7302	2,354	4,453	1,892
rplS	12,478	23,885	1,914
asr7146	0,000	1,917	1,917
all5016	11,506	22,167	1,927
all3788	4,397	8,471	1,927
all1704	4,379	8,436	1,927
alr1295	3,878	7,471	1,927
all1130	0,000	1,937	1,937
menA	4,896	9,500	1,940
all4986	4,834	9,379	1,940
alr8502	0,000	1,948	1,948
alr5124	0,000	1,958	1,958

asl0815	7,413	14,608	1,970
all8031	3,360	6,621	1,970
all5166	2,347	4,625	1,970
all2969	1,610	3,173	1,970
asl3129	4,916	9,687	1,970
all1411	3,766	7,422	1,970
all4892	2,044	4,028	1,970
rimM	1,891	3,726	1,970
alr3906	1,650	3,252	1,970
trpC_1	1,605	3,162	1,970
thrA	1,086	2,140	1,970
all4433	0,903	1,780	1,970
all2444	0,696	1,372	1,970
all4940	6,625	13,054	1,970
alr4534	3,565	7,025	1,970
all7198	3,013	5,937	1,970
all3111	2,273	4,478	1,970
alr0288	2,094	4,127	1,970
alr2559	1,521	2,998	1,970
all4024	0,971	1,913	1,970
all3045	0,923	1,819	1,970
alr2739	0,789	1,555	1,970
alr4939	9,532	18,781	1,970
alr4637	2,780	5,478	1,970
alr4853	6,904	13,721	1,988

Table D. Main genes involved in iron uptake showing ≥2-fold change in expression in the *furA*-turning off strain AGcoaRFurA

ORF	Symbol[a]	Protein description[a]	Fold change
alr0397	*schT*	schizokinen outer membrane transporter SchT	-2.02
all2585	*fecD1*	iron(III) dicitrate transport system permease protein	-2.54
all1101		ferrichrome iron receptor (SchT homologue)	-6.66
all2148		ferrichrome iron receptor	-2.54
all2158		ferrichrome iron receptor	-2.54
alr2185		ferrichrome iron receptor	-2.25
all2236		ferrichrome iron receptor	-5.07
alr2588		ferrichrome iron receptor	-3.81
alr2610		ferrichrome iron receptor	-2.10
all4924		ferrichrome iron receptor	-5.71
alr1382	*futA1*	ABC transporter, ferric iron-binding periplasmic protein FutA1 orthologue)	-3.81
alr1383	*futB*	iron(III) ABC transporter, permease protein (FutB orthologue)	-2.22
alr1384	*futC*	ABC transporter ATP-binding protein	-2.54
alr2209		ferric aerobactin receptor	-4.44
all2158		ferrichrome-iron receptor	-2.54
all3310		TonB-dependent receptor	2.76
all2610		ferrichrome-iron receptor	2.10

[a]Gene symbol and protein description according to the cyanobacteria genome database CyanoBase (http://genome.microbedb.jp/cyanobase).

Table E. Strains and plasmids used in this study

Strain or plasmid	Characteristics	Source or reference
Strains		
E. coli		
DH5α	F^- φ80*lac*ZΔM15 Δ(*lac*ZYA-*arg*F)U169 *recA1 endA1 hsdR17*(rK^-, mK^+) *phoA supE44 thi-1 gyrA96 relA1* λ^-, for cloning and maintaining plasmids	Thermo Fisher Scientific
ED8654	F^- *e14*$^-$ (*mcr*A$^-$) *rec*A 56 *lac-3 o lac*Y1 *gal*K2 *gal*T22 *gln*V44 *sup*F58 *met*B1 *hsd*R514 (rK^- mK^+) *trp*R55, for triparental conjugation	Institute of Plant Biochemistry and Photosynthesis, Seville, Spain
CPB1893	*mcr*A$^-$ *mcr*B$^-$ M.*Eco*K$^+$ R.*Eco*K$^-$, for triparental conjugation	Institute of Plant Biochemistry and Photosynthesis, Seville, Spain
BL21(DE3)	*ompT* $hsdS_B$ (r_B^- m_B^-) *gal dcm* (DE3), for expression and purification of recombinant FurA	EMD Biosciences
Anabaena sp.		
PCC 7120	Wild-type	Pasteur Institute, Paris, France
AG2770FurA	PCC 7120 harboring pAM2770FurA, overexpresses FurA	González et al., 2010
AGcoaRfurA	PCC 7120 with natural promoter of *furA* replaced by locus *coaR*-P_{coaT} from *Synechocystis* sp. PCC 6803, depleted expression of *furA* in BG-$11_{-Co/Zn}$ medium	This study
Plasmids		
pGEM-T	Cloning vector, Amp^r	Promega
pCoaR1	pGEM-T vector harboring the native 2-kb sequence from the upstream region of the *furA* gene	This study
pAM2770FurA	pAM2770 vector containing *furA* expressed from *petE* promoter, Km^r/Nm^r	González et al., 2010
pCoaR2	pAM2770FurA vector harboring the *coaR*-P_{coaT} locus from *Synechocystis* sp. PCC 6803	This study
pCoaR3	pCoaR1 vector harbouring the fusion fragment *coaR*-P_{coaT}:: *furA*	This study

pCoaR4	pGEM-T vector harboring the native 2-kb sequence from the downstream region of the *furA* gene	This study
pCoaR5	pCoaR3 vector harboring the native 2-kb sequence from the downstream region of the *furA* gene	This study
pCoaR6	pCoaR5 vector harboring the Km/Nm-resistance transcription unit from pAM2770FurA	This study
pRL278	Suicide vector; Km^r/Nm^r	Black et al., 1993
pCoaRFurA	Suicide vector containing the fusion fragment *coaR*-P_{coaT}:: *furA* flanked by the 2-kb upstream and downstream regions of the *furA* gene	This study
pRL443	Conjugal plasmid; Amp^r Tc^r; Km^s derivative of RP-4	Elhai et al., 1997
pRL623	Conjugation helper plasmid; Cm^r; Mob_{ColK}, M·*Ava*II, M·*Eco*47II, M·*Eco*T221	Elhai et al., 1997
pET28a	Expression vector, Km^r	EMD Biosciences

Table F. Oligonucleotides used in this study

Primer	Sequence 5´- 3´
Cloning	
2770FurA-coaR-P_up	CCCTCGAGCTAAAGACAAGTGAGATAGC
2770FurAcoaR-P_dw	GGAATTCCATATGGCTTTTTAACTTGGATTTTTAC
PpetEfurA1	AGATCTCTCATCACTACTCATCATCCCC
PpetEfurA2	CTCGAGTAATGTATAAACTCCAATCACAAGG
PpetEfurA3	GGATCCGGTAAGATAACAAGAGGAAGTTACTC
PpetEfurA4	GAGCTCCTATTATCAATTTCAAGGTGGTTTAAC
Km/Nm_up	CCCTCGAGGCTACTGGGCTATCTGGAC
Km/Nm_dw	CCCTCGAGGTCCCGCTCAGAAGAAC
EMSA	
P1_asl2914-up	GTGCGGCTTTTGCCTACGC
P1_asl2914-dw	CAAGCCACTGGACAAGCATCTACG
P2_asl2914-up	GTCAGCGACTCACCCTAAGCG
P2_asl2914-dw	CCAACGGTGAAACACTTGTTGTAAC
Pasl0884-up	CTTCGATCCTCTGGTCGATGTTC
Pasl0884-dw	CCATTGCCTTAAGCCGCAG
Pasl0885-up	GAAAGTGTGAATCAGCATAGGTTTTAGAG
Pasl0885-dw	CGACCAGAGGATCGAAGATTTG
Pall4148-up	GCTGCCTCCGACTCTTAATATAATCC
Pall4148-dw	GGTTGCCATTTCTGTAATCCTCTC
Pall0801-up	GGTTTAGGGGTGTAAGGGTTTAGG

Pall0801-dw	CGGTAATATCTTCAGTAGTGCCTTGG
Pasr0847-46-up	CCAGCCTGGAGCCACTTTACC
Pasr0847-46-dw	GGTCGGATAATTCTCTGCTAGGAGG
P1_asr3848-up	CGATCGCCTCAATGCAGTTTATTC
P1_asr3848-dw	CGACGACCCACAGGGGAATTC
P2_asr3848-46-up	CCGAGATGTAGAAGAAGTGGCAGC
P2_asr3848-46-dw	GGTGACGATATCGGAAAACGG
Pasr1283-up	GAGGTAGGGGTGTGGCAATACG
Pasr1283-dw	GCCGGTATTAGCAACTGGGG
P1_alr3397-up	GCAATATTGATAATCTCAGCTAACGTATG
P1_alr3397-dw	GTCAAGCCAGTAGAGTTTATCCACTC
P2_alr3397-up	CTCAAGCCTGCTGGTTTACGC
P2_alr3397-dw	GCTTCACCATACGTTAGCTGAGATTATC
P1_alr3146-47-up	CCCAAGGTGTGGGCAGAATTG
P1_alr3146-47-dw	CCAAACGTCCCTCCTCTGGTG
P2_alr3146-47-up	GTATCTGTGGGGATACTGGAACAATAG
P2_alr3146-47-dw	CCAGAGAACCAACGATTCCTCTTG
P3_alr3146-47-up	CTCCTTCTACGTCATATATGATATCCG
P3_alr3146-47-dw	CTATTGTTCCAGTATCCCCACAGATAC
Palr3422-up	CAGCGCCCACACATTTGTACTAC
Palr3422-dw	GTAGTAGTTGTGACCCATGCCTTTG
Pall1512-up	GCGTATTGCTGGTGACAGCTTTAG
Pall1512-dw	CGTCTTGATAGGGATGGACTTTCG
Pall1365-up	GCGATACGCTGAATCAGAATTTCAG
Pall1365-dw	GAGGGTGACAACAATCAAACCAAG

P1_alr4685-86-up	CAAGTTGAGGAAACTTCACAATCTATTAG
P1_alr4685-86-dw	CATTGAAGCGCATAGTCTTGTAGAAC
P2_alr4685-86-up	GCGGTTGATACACAGCTTATTTCAC
P2_alr4685-86-dw	GCTAATAGATTGTGAAGTTTCCTCAACTTG
P3_alr4685-86-up	GGATACCTAGTGGCATAGTTAGTACAG
P3_alr4685-86-dw	GTGAAATAAGCTGTGTATCAACCG
Palr2392-93-94-up	CTGTTGAAAAGCTGTAACGTGGC
Palr2392-93-94-dw	GCGCCAATCGGCCAAATC
Pall1291-up	CCTGTAACAGAGTTTGCGTAATTTCC
Pall1291-dw	GTCTTGATGATTGGTATTGGTAAGGG
Pall3735-up	CAGCCCCTGGTAGATGTAATAAGTCTTG
Pall3735-dw	CCTTTAGAGCCAGATTTGCCG
P1_alr1105-04-up	GCTACGCTTTACAAGACAAAGTATGGG
P1_alr1105-04-dw	CCTGCACTAGTCACAGCGATATTATC
P2_alr1105-04-up	GTTCTGGTATCAGTCGATAGTAAATCCAAG
P2_alr1105-04-dw	GGCTACGTGTGTTTTCATAACGG
P1_all0450-up	CAGCATCTGCATTGAGAATCGAC
P1_all0450-dw	GGCACGCAGAAGAATCTCAGC
P2_all0450-up	GGCTGAGATTCTGCGTGC
P2_all0450-dw	GTAGCGGCCTATTAACCTTGTTTC
P1_all3549-up	GCAAGACGTGAAGTTGTTTCTATGG
P1_all3549-dw	CCTCATCTTCTGAAGCTCCCGTC
P2_all3549-up	GGTTGTAGCTGTACTGGCTTGAGTTG
P2_all3549-dw	CATCAATCGCTGAGTTTGCAGAG
Pall3653-up	GCTAAGGTAGCGACGATTCTTGTTC

Pall3653-dw	GTCAGTTGTCAATTGTTCGTTGTGAAG
Palr3814-up	GGCTTTAGGACAGACTGTGGGC
Palr3814-dw	CCGCACGTAGGCGATCGCCC
P1_sufBCDS-up	GGGCTGTTGCCTGTGAGTGTTTC
P1_sufBCDS-dw	GCCGTACTTGTAAGGTTGGTTGAC
P2_sufBCDS-up	GCTACCGATGGAGTTTGCGG
P2_sufBCDS-dw	GCAGGATGTCCCGCCAACAC
P3_sufBCDS-up	GCAACCGTCAGCCAGTTGGAAGATG
P3_sufBCDS-dw	CGATGTCGCCGCGTTATCTAG
Palr4908-up	GCCCATCGTGGTGAAGAGATTAC
Palr4908-dw	GGGCGAATGCTGGTGAATACG
Pall3272-up	CGGTATTGATAGCCATTCCTGC
Pall3272-dw	GAAGGGAGTTTAACTTACAAAGACAGTCAG
Palr3195-up	GCGTAGGTGCAATGTTATATTGAG
Palr3195-dw	CGTACAAATGTAAGTTTTCCATGTAAAG
Palr7354-up	GACAGCGCCATAAGTACCAGCAG
Palr7354-dw	GATGCCTAACTCTTCAAGTAGCCATAGC
Pasl4146-up	GTTGCCACGGGGCAATGG
Pasl4146-dw	CCGATGCGCCTGTCAATCC
Pall0177-up	GGGGCTTGGAATCGCTCATAG
Pall0177-dw	CTGACCGCAGTTCGTCACCG
Palr3090-up	GGACTACTCAGCAGAAGCAGAAGTG
Palr3090-dw	GCTGCTGATAGTTCTCCTGTCGC
Pall5185-up	CTCATCCACTGGGCGGACG
Pall5185-dw	GTCGTGCCACAATTATCAAAATGG

Palr0799-up	CTGAAGCCATCCACGCCTTAG
Palr0799-dw	GGAACACCCAAAGTATTGAGAATCTG
P1_asr0941-up	CCAAACGCTCAGGTTAACACCAG
P1_asr0941-dw	GTACCCCAAGCCAAACTCCAAAG
P2_asr0941-up	CTCATCCGTGCCAACATCGTC
P2_asr0941-dw	GTAGCTTAGATCGATGACTGGTGTTAACC
P1_asr4517-up	GAACAGTTTTGAATAGGTAG
P1_asr4517-dw	CATAACAGACTCCTAAAAGAC
P2_asr4517-up	CTCAGCAATTTGTTCAACCTGAGC
P2_asr4517-dw	CTGGTATATAACCCAAGTCTGCTAGAG
Pall3135-up	GCCTACCTTCTGGATACTTGCAATAG
Pall3135-dw	GGTCATGGGTAATTAGTAATGGGTAACTAG
Palr7386-up	GCGCTCAATTACACCCCGC
Palr7386-dw	CGGCGGCGAAGTAATAGACG
P1_asr7385-up	GCATTCTTGGCTTGTGCCG
P1_asr7385-dw	GCTGAATCTGGTAATAGATGACTTAGCAC
P2_asr7385-up	GCCTCTCACACTCTACGTCTATTACTTCG
P2_asr7385-dw	CGGCACAAGCCAAGAATGC
Palr1010-up	CTCCATTAATTTGGTGTCAATATTATTCAC
Palr1010-dw	CGTCGCGTTCTACACTGGCC
Palr3513-up	CCCATTCCCGATTTTGGCG
Palr3513-dw	CCGGGAGTTTATCAGTTTCTGGG
Palr5007_up	GGTCTAGGGATGATAGTTGTATTCCAG
Palr5007_dw	CACCTTGAGGAGGATTGATAGTC
Pall7348-P1_up	CTGGTGGGAGTTATTGGATACCACTG

Pall7348-P1_dw	GCTGATGATTGCTATGGCAAGG
Pall7348-P2_up	CCTTGCCATAGCAATCATCAGC
Pall7348-P2_dw	GCGATCGCCTAAAACTTTAC
Pall2097_up	GGAACTCTACCACCGCTACTGGATAC
Pall2097_dw	CTAGTATCAAGTGAACTTAGATCATTGGG
Palr3707_up	GACAACTGACAACTGACCATTGATC
Palr3707_dw	GGGAATGGAAGTAAGTGACATATTCTATTC
Palr2945_up	GGCGCGCCTTTACGTTTAGC
Palr2945_dw	CTATAGCGTCCGGGCATCATTTC
Palr0034_up	CTACGACGACATGGTGAAACTGTC
Palr0034_dw	GCTTTGTAGTCATCAGTTGTGGTAAGC
Palr3356-P1_up	CTGTAGGCATTGCCCACCTTACGTA
Palr3356-P1_dw	GAACTAGGATCAAATTCTGCGTGAG
Palr3356-P2_up	GCCATAGCAATACTGTTCCATTTG
Palr3356-P2_dw	GAACAATCCTGTTAGGAATAATCAG
Pall4936_up	GGAAACTGCCGCCAAATTG
Pall4936_dw	CAAAGCTGAGATTATCAGCAACTTGAG
Pall1649-47_up	GCAATAGGTTCCATCTTATAAATCCG
Pall1649-47_dw	CAACTACCCAACTATTGCTCAATTGG
Pall1647_up	CGCTTGCTGAATAATCTGATAATAGGG
Pall1647_dw	CGTCAAGCCCTCCTGAATTGC
Palr2679-80_up	GCAGTGATGGTTTGCTGACACAATC
Palr2679-80_dw	GTCGTTGGCGGTTAATCATTATGC
Palr2680_up	CCCCAGAACCCCAAACCGAAC
Palr2680_dw	CGGGGGTGTCTGCACCTCC

Pall4026-P1_up	CAAGGCTGCCCCACACTCC
Pall4026-P1_dw	GTGCCTGATCTACTTACTTTGCTCTGC
Pall4026-P2_up	GCAGAGCAAAGTAAGTAGATCAGGCAC
Pall4026-P2_dw	GCTGGGGCGATCGCATAACTTAAG
Pall4026-P3_up	GTTATGCGATCGCCCCAGC
Pall4026-P3_dw	GACGCTATGAGATAGTGATCCTCTTTAAG
Pall1847-48_up	GCGGAAGTCATGACCGTAGTATTG
Pall1847-48_dw	GCGCTGGAAGAATTAATCAATTACTAC
Palr1450-49_up	CGTCGGCTCAATAGCTCATGG
Palr1450-49_dw	CTGTAGCCATCTGGCGCTTCC
Palr3100-P1_up	CCAATCCCTGGGGGTATGTCATGG
Palr3100-P1_dw	GCGCTCAACGGTGGTAATTCCC
Palr3100-P2_up	CTAGTTCCACCCTTAAGGCAGACTC
Palr3100-P2_dw	GAAGTCATGTTGATGTGAGCTGCC
Pall2566_up	GCACAAGTCGCCCGATACG
Pall2566_dw	CCCAACACGGCGAAGATATGC
Pall1681_up	GGACGTGGTGACGATTCCAAG
Pall1681_dw	CAATGGTGGCTGTAACACTTGGTAC
Pasr8504_up	CCAGGAATTGCTCAAGCATGAG
Pasr8504_dw	GGTGGTTGATGCCGGAATCC
Pall3033-P1_up	GCACCCATTCCGTCATAACTAGAAAG
Pall3033-P1_dw	CGCTTCGGTTGTGTAAGTACAGAGTTG
Pall3033-P2_up	CGAACACATCAACGAGAAGCAGG
Pall3033-P2_dw	CTCATCCACGCGACAGGTCTG
Palr1358_up	GGTCAGCAATACTGTGTGATTTGC

Palr1358_dw	CTGGGGGTTTTGATTCCTGG
RT-PCR	
Alr2679-RT_up	CAGCCCCCAACGGTACAGC
Alr2679-RT_dw	GGGGTGGGTGTCGTAAAGTTCC
Alr2680-RT_up	CCTTCTACCACAACAACCCCGC
Alr2680-RT_dw	CGCCAAATACAGCCCCTAATGC
Alr0799-RT_up	GGTTTTCATGAAGGGAACCAAATTG
Alr0799-RT_dw	GAAGCTAAAGCAACTTCCACTAATTGC
Asl4146-RT_up	GCTAAGTGCATCTTCAAAACACTACGTG
Asl4146-RT_dw	GGTTAGGGTACAAGAGGTTCCATTAAACC
All5185-RT_up	GTGGGGTAGGGATGAATCACGTTAC
All5185-RT_dw	CGGCATTGAACAAGCTGGATATC
Alr3090-RT_up	GGTGGAGCGACAGGAGAACTATCAG
Alr3090-RT_dw	GCTGGCTCAGAGTTCCAAGGG
Alr3356-RT_up	GTTCTCCAGCGTCTTGTCCAGACAATG
Alr3356-RT_dw	GCTCCTGCCTGCATCAATTCTATG
Asr4517-RT_up	GAACCAACCAATCGAATTGTCATTAG
Asr4517-RT_dw	GCCGGAGTGGAACCAGAATCTAAG
Asr7385-RT_up	GGCGTTCCGCAAGAGCGTAG
Asr7385-RT_dw	GTTAGGTTGAGATTGGAAAGTGAGAAC
Asr7386-RT_up	CTGGAAAATTGCATTCTTGACGAG
Asr7386-RT_dw	GGTCTCTTACATTGGTCAGTCTTTCTG
All3135-RT_up	CCGAGCATCGCTGCTCAGG
All3135-RT_dw	GGAACGCTCATTAACGACGCAC
Alr2495-RT_up	CCCTTGCAGATAAAGTCCGCG

Alr2495-RT_dw	GCGATCGCTTCCCCAATTGC
Alr4686-RT_up	GCGAAGACAACTCCTTATGCCCTC
Alr4686-RT_dw	CCACGTACACCACCACCAAATGG
All7348-RT_up	GGTACTTCCGTAACTCCCTGCCAC
All7348-RT_dw	CTCATGTGTCGCCATCATGGTTG
Alr1010-RT_up	GGCCAGTGTAGAACGCGACG
Alr1010-RT_dw	CCCCTAGCTAACCAATATAACCAATCAG

Table G. Summary of sequencing run statistics

Sample A (*Anabaena* sp. AGcoaRFurA)	Total number of bases [Mbp]	635
	% of Q30 bases	95,9
	Final library reads	4,234,002
Sample B (*Anabaena* sp. PCC 7120)	Total number of bases [Mbp]	1,034
	% of Q30 bases	96,06
	Final library reads	6,890,192
